# GWAS and meta-analysis identifies 49 genetic variants underlying critical COVID-19

**DOI:** 10.1038/s41586-023-06034-3

**Published:** 2023-05-17

**Authors:** Erola Pairo-Castineira, Konrad Rawlik, Andrew D. Bretherick, Ting Qi, Yang Wu, Isar Nassiri, Glenn A. McConkey, Marie Zechner, Lucija Klaric, Fiona Griffiths, Wilna Oosthuyzen, Athanasios Kousathanas, Anne Richmond, Jonathan Millar, Clark D. Russell, Tomas Malinauskas, Ryan Thwaites, Kirstie Morrice, Sean Keating, David Maslove, Alistair Nichol, Malcolm G. Semple, Julian Knight, Manu Shankar-Hari, Charlotte Summers, Charles Hinds, Peter Horby, Lowell Ling, Danny McAuley, Hugh Montgomery, Peter J. M. Openshaw, Colin Begg, Timothy Walsh, Albert Tenesa, Carlos Flores, José A. Riancho, Augusto Rojas-Martinez, Pablo Lapunzina, GenOMICC Investigators, Sara Clohisey, Johnny Millar, Manu Shankar-Hari, Emma Aitkin, Latha Aravindan, Ruth Armstrong, J. Kenneth Baillie, Heather Biggs, Ceilia Boz, Adam Brown, Primmy Chikowore, Richard Clark, Audrey Coutts, Judy Coyle, Louise Cullum, Sukamal Das, Nicky Day, Lorna Donnelly, Esther Duncan, Paul Finernan, Max Head Fourman, Anita Furlong, James Furniss, Bernadette Gallagher, Tammy Gilchrist, Ailsa Golightly, Fiona Griffiths, Katarzyna Hafezi, Debbie Hamilton, Ross Hendry, Naomi Kearns, Dawn Law, Rachel Law, Sarah Law, Rebecca Lidstone-Scott, Christen Lauder, Louise Macgillivray, Alan Maclean, Hanning Mal, Sarah McCafferty, Ellie McMaster, Jen Meikle, Shona C. Moore, Sheena Murphy, Hellen Mybaya, Miranda Odam, Wilna Oosthuyzen, Chenqing Zheng, Jiantao Chen, Nick Parkinson, Trevor Paterson, Petra Tucker, Katherine Schon, Andrew Stenhouse, Mihaela Das, Maaike Swets, Helen Szoor-McElhinney, Filip Taneski, Lance Turtle, Tony Wackett, Mairi Ward, Jane Weaver, Nicola Wrobel, Marie Zechner, Jacqueline Pan, Neus Grau, Tim Owen Jones, Rosario Lim, Martina Marotti, Christopher Whitton, Aneta Bociek, Sara Campos, Gill Arbane, Manu Shankar-Hari, Marlies Ostermann, Mina Cha, Fabiola DAmato, Eirini Kosifidou, Shelley Lorah, Kyma Morera, Sarah Bircham, Laura Brady, Keith Hugill, Jeremy Henning, Stephen Bonner, Evie Headlam, Jessica Jones, Abigail List, Joanne Morley, Amy Welford, Bobette Kamangu, Anitha Ratnakumar, Abiola Shoremekun, Zoe Alldis, Raine Astin-Chamberlain, Fatima Bibi, Jack Biddle, Sarah Blow, Matthew Bolton, Catherine Borra, Ruth Bowles, Maudrian Burton, Yasmin Choudhury, Amber Cox, Amy Easthope, Patrizia Ebano, Stavros Fotiadis, Jana Gurasashvili, Rosslyn Halls, Pippa Hartridge, Delordson Kallon, Jamila Kassam, Ivone Lancoma-Malcolm, Maninderpal Matharu, Peter May, Oliver Mitchelmore, Tabitha Newman, Mital Patel, Jane Pheby, Irene Pinzuti, Zoe Prime, Oleksandra Prysyazhna, Julian Shiel, Melanie Taylor, Carey Tierney, Olivier Zongo, Suzanne Wood, Anne Zak, David Collier, Manuela Mundy, Christopher Thompson, Lisa Pritchard, Minnie Gellamucho, David Cartlidge, Nageswar Bandla, Lucy Bailey, Michelle Davies, Jane Delaney, Leanne Scott, Marwa Abdelrazik, Frater Alasdair, David Carter, Munzir Elhassan, Arunkumar Ganesan, Samuel Jenkins, Zoe Lamond, Dharam Purohit, Kumar Rohit, Malik Saleem, Alanna Wall, Kugan Xavier, Dhanalaksmi Bakthavatsalam, Kirolos Gehad, Pakeerathan Gnanapragasam, Kapil Jain, Swati Jain, Abdul Malik, Naveen Pappachan, Jeronimo Moreno-Cuesta, Anne Haldeos, Rachel Vincent, Maryjane Oziegb, Anna Cavazza, Maeve Cockrell, Eleanor Corcoran, Maria Depante, Clare Finney, Ellen Jerome, Abigail Knighton, Monalisa Nayak, Evita Pappa, Rohit Saha, Sian Saha, Andrew Dodd, Kevin O’Reilly, Mark McPhail, Emma Clarey, Harriet Noble, John Smith, Phoebe Coghlan, Stephen Brett, Anthony Gordon, Maie Templeton, David Antcliffe, Dorota Banach, Sarah Darnell, Ziortza Fernandez, Eleanor Jepson, Amal Mohammed, Roceld Rojo, Sonia Sousa Arias, Anita Tamang Gurung, Jenny Wong, Jaime Fernandez-Roman, David O. Hamilton, Emily Johnson, Brian Johnston, Maria Lopez Martinez, Suleman Mulla, Alicia A. C. Waite, Karen Williams, Victoria Waugh, Ingeborg Welters, Jessica Emblem, Maria Norris, David Shaw, Archana Bashyal, Sally Beer, Paula Hutton, Stuart McKechnie, Neil Davidson, Soya Mathew, Grace Readion, Jung Ryu, Jean Wilson, Shruti Agrawal, Kay Elston, Megan Jones, Eoghan Meaney, Petra Polgarova, Muhammad Elbehery, Charlotte Summers, Esther Daubney, Anthony Ng, Jocelyn Marshall, Nazima Pathan, Katerina Stroud, Deborah White, Angela Andrew, Saima Ashraf, Amy Clark, Martin Dent, Margaret Langley, Cecilia Peters, Lucy Ryan, Julia Sampson, Shuying Wei, Alice Baddeley, Megan Meredith, Lucy Morris, Alexandra Gibbons, Lisa McLoughlin, Carlos Castro Delgado, Victoria Clark, Deborah Dawson, Lijun Ding, Georgia Durrant, Obiageri Ezeobu, Abiola Harrison, William James Hurt, Rebecca Kanu, Ashley Kinch, Susannah Leaver, Ana Lisboa, Jisha Mathew, Kamal Patel, Romina Pepermans Saluzzio, John Rawlins, Tinashe Samakomva, Nirav Shah, Christine Sicat, Joana Texeira, Joana Gomes De Queiroz, Edna Fernandes Da Gloria, Elena Maccacari, Nikki Yun, Soumendu Manna, Sarah Farnell-Ward, Maria Maizcordoba, Maria Thanasi, Hawakin Haji Ali, Janice Hastings, Lina Grauslyte, Musarat Hussain, Bobby Ruge, Sam King, Tatiana Pogreban, Lace Rosaroso, Helen Smith, Mandeep-Kaur Phull, Nikkita Adams, George Franke, Aparna George, Erika Salciute, Joanna Wong, Karen Dunne, Luke Flower, Emma Sharland, Sukhmani Sra, Gillian Andrew, Marie Callaghan, Lucy Barclay, Lucy Marshall, Kenneth Baillie, Maria Amamio, Sophie Birch, Kate Briton, Sarah Clark, Katerine Doverman, Dave Hope, Corrienne Mcculloch, Scott Simpson, Jo Singleton, Rita Fernandez, Meryem Allen, David Baptista, Rebecca Crowe, Jonathan Fox, Jacyntha Khera, Adam Loveridge, India McKenley, Eriko Morino, Andres Naranjo, Denise O’Connor, Richard Simms, Kathryn Sollesta, Andrew Swain, Harish Venkatesh, Rosie Herdman-Grant, Anna Joseph, Angela Nown, Steve Rose, David Pogson, Helen Boxall, Lutece Brimfield, Helen Claridge, Zoe Daly, Shenu George, Andrew Gribbin, Yusuf Cheema, Sean Cutler, Owen Richards, Anna Roynon-Reed, Shiney Cherian, Anne Emma Heron, Gemma Williams, Tamas Szakmany, Abby Waters, Kim Collins, Jill Dunhill, Ffion Jones, Rebecca Morris, Lucy Ship, Amy Cardwell, Syamlan Ali, Ravi Bhatterjee, Rachel Bolton, Srikanth Chukkambotla, Dabheoc Coleman, Jack Dalziel, Joseph Dykes, Christopher Fine, Bethan Gay, Wendy Goddard, Drew Goodchild, Rhiannan Harling, Muhammad Hijazi, Sarah Keith, Meherunnisa Khan, Roseanna Matt, Janet Ryan-Smith, Samuel Saad, Philippa Springle, Jacqueline Thomas, Nick Truman, Aayesha Kazi, Matthew Smith, Heather Collier, Chloe Davison, Stephen Duberley, Jeanette Hargreaves, Janice Hartley, Tahera Patel, Ellen Smith, Alissa Kent, Emma Goodwin, Ahmed Zaki, Clare Tibke, Susan Hopkins, Hywel Gerrard, Matthew Jackson, Sara Bennett, Liane Marsh, Rebecca Mills, Jessica Bell, Helen Campbell, Angela Dawson, Steve Dodds, Stacey Duffy, Lisa Gallagher, Gemma McCafferty, Stacey Short, Tracy Smith, Kirsty Thomas, Claire Walker, Jessica Reynolds, Bryan Yates, Hayley McKie, Maria Panteli, Maria Thompson, Gail Waddell, Sarah De Beger, Azmerelda Abraheem, Charlie Dunmore, Rumanah Girach, Rhianna Jones, Emily London, Imrun Nagra, Farah Nasir, Hannah Sainsbury, Clare Smedley, Stephen Brearey, Caroline Burchett, Kathryn Cawley, Maria Faulkner, Helen Jeffrey, Peter Bamford, Firdaus Shaikh, Lauren Slack, Angela Davies, Hollie Brooke, Jose Cebrian Suarez, Ruth Charlesworth, Karen Hansson, John Norris, Alice Poole, Rajdeep Sandhu, Elizabeth Smithson, Muthu Thirumaran, Veronica Wagstaff, Sarah Buckley, Brendan Sloan, Alastair Rose, Amy Major, Alexandra Metcalfe, Christine Almaden-Boyle, Pauline Austin, Susan Chapman, Alexandre Eros, Louise Cabrelli, Stephen Cole, Clare Whyte, Matt Casey, Vasileios Bafitis, George Tsinaslanidis, Cassandra George, Reena Khade, Christopher Black, Sundar Raj Ashok, Sean Farley, Elaine Brinkworth, Rachel Harford, Carl Murphy, Marie Williams, Luke Newey, Hannah Toghill, Sophie Lewis, Tabitha Rees, Ceri Battle, Mark Baker, Jenny Travers, Karen Chesters, Nicola Baxter, Andrew Arnott, Gordan McCreath, Christopher McParland, Laura Rooney, Malcolm Sim, Steven Henderson, Lynn Abel, Carol Dalton, Sophie Kennedy-Hay, Lynn O’Donohoe, Megan O’Hare, Izabela Orlikowska, Natasha Parker, Fiona McNeela, Amanda Lyle, Alistair Hughes, Jayachandran Radhakrishnan, Sian Gibson, Hollie Bancroft, Mary Bellamy, Jacqueline Daglish, Salma Kadiri, Faye Moore, Joanne Rhodes, Mirriam Sangombe, Zhane Peterkin, James Scriven, Margaret Carmody, Juliet Cottle, Emily Peasgood, Laura Ortiz-Ruiz de Gordoa, Claire Phillips, Denise Skinner, Zoe Cinquina, Kate Howard, Rosie Joy, Samantha Roche, Isobel Birkinshaw, Joseph Carter, Jo Ingham, Nicola Marshall, Harriet Pearson, Zoe Scott, Jo Dasgin, Jaspret Gill, Annette Nilsson, Amy Bamford, Diana Hull, James Scriven, Nafeesah Ahmadhaider, Michelle Bates, Christopher McGhee, Hannah Ellis, Gwenllian Sera Howe, Jayaprakash Singh, Natalie Stroud, Lisa Roche, Ceri Lynch, Bethan Deacon, Carla Pothecary, Justyna Smeaton, Kevin Agravante, Vinodh Krishnamurthy, Cynthia Diaba, Lincy John, Lai Lim, Rajeev Jha, Jasmine Egan, Timothy Felton, Susannah Glasgow, Grace Padden, Ozerah Choudhr, Joanne Bradley-Potts, Stuart Moss, Saejohn Lingeswaran, Peter Alexander, Craig Brandwood, Sofia Fiouni, Luke Ward, Schvearn Allen, Jane Shaw, Christopher Smith, Oluronke Adanini, Rebecca Collins, Maines Msiska, Linda Ofori, Nikhil Bhatia, Hayley Dolan, Mark Brunton, Jess Caterson, Holly Coles, Liza Keating, Emma Tilney, Nicola Jacques, Matthew Frise, Jennifer Armistead, Shauna Bartley, Parminder Bhuie, Sabi Rai, Gabriela Tomkova, Sandra Greer, Karen Shuker, Ascanio Tridente, Emma Dobson, Jodie Hunt, Redmond Tully, Joy Dearden, Andrew Drummond, Prakash Kamath, Emily Bullock, Michelle Mulcahy, Shelia Munt, Grainne O’Connor, Jennifer Philbin, Chloe Rishton, Chloe Scott, Sarah Winnard, Nurkamalia Hasni, Rachel Gascoyne, Joanne Hawes, Kelly Pritchard, Lesley Stevenson, Amanda Whileman, Sarah Beavis, Lauren Bishop, Cindy Cart, Katie Dale, Mary Kelly-Baxter, Adam Mendelski, Emma Moakes, Rheanna Smith, Jan Woodward, Stephanie Wright, Angela Allan, Adriana Botello, Jade Liew, Jasmine Medhora, Erin Trumper, Felicity Savage, Teresa Scott, Marc Place, Callum Kaye, Sarah Benyon, Suzie Marriott, Linda Park, Helen Quinn, Daisy Skyes, Lily Zitter, Kizzy Baines, Elizabeth Gordon, Samantha Keenan, Andrew Pitt, Katharine Duffy, Jane Ireland, Gary Semple, Lynne Turner, Susanne Cathcart, Dominic Rimmer, Alex Puxty, Kathryn Puxty, Andrew Hurst, Jennifer Miller, Susan Speirs, Lauren Walker, Zena Bradshaw, Joanna Brown, Sarah Melling, Stephen Preston, Nicola Slawson, Scott Warden, Alanna Beasley, Emma Stoddard, Leonie Benham, Jason Cupitt, Melanie Caswell, Lisa Elawamy, Ashleigh Wignall, Belinda Roberts, Hannah Golding, Samantha Leggett, Michelle Male, Martyna Marani, Kirsty Prager, Toran Williams, Kim Golder, Oliver Jones, Rebecca Cusack, Clare Bolger, Rachel Burnish, Michael Carter, Susan Jackson, Karen Salmon, Jonathan Biss, Maia Aquino, Maria Croft, Victoria Frost, Ian White, Keshnie Govender, Natasha Webb, Liana Stapleton, Colin Wells, Nikitas Nikitas, Ana Sanchez-Rodriguez, Kayleigh Spencer, Bethan Stowe, Yvonne Izzard, Michelle Poole, Sonja Monnery, Sallyanne Trotman, Valerie Beech, Edward Combes, Teishel Joefield, Patrick Covernton, Sarah Savage, Elizabeth Woodward, Julie Camsooksai, Henrik Reschreiter, Charlotte Barclay, Yasmin DeAth, Judith Dube, Charlotte Humphrey, Sarah Jenkins, Emma Langridge, Rebecca Milne, Beverley Wadams, Megan Woolcock, Michael Brett, Brian Digby, Lisa Gemmell, James Hornsby, Patrick MacGoey, Pauline O’Neil, Richard Price, Radha Sundaram, Lynn Abel, Natalie Rodden, Nicola Thomson, Kevin Rooney, Susan Currie, Natasha Parker, Lauren Walker, Philip Henderson, Bethan Ogg, Simon Whiteley, Liz Wilby, Kate Long, Shailamma Matthew, Sheila Salada, Susan Trott, Sarah Watts, Zoe Friar, Abigail Speight, Victoria Bastion, Humza Chandna, Brice Djeugam, Muhammad Haseeb, Harriet Kent, Gamu Lubimbi, Sophie Murdoch, Alastair Thomas, Beena David, Rachel Lorusso, Ana Vochin, Melchizedek Penacerrada, Retno Wulandari, Charlotte Heath, Srinivas Jakkula, Anna Morris, Ashar Ahmed, Arvind Nune, Claire Buttriss, Emma Whitaker, Miriam Davey, David Golden, Amy Acklery, Fabio Fernandes, Bec Seaman, Victoria Earl, Amy Collins, Waqas Khaliq, Rachel Adam, Estefania Treus, Sarah Holland, Jordan Alfonso, Bethan Blackledge, Michelle Bruce, Laura Jayne Durrans, Ayaa Eltayeb, Jade Harris, Samuel Hey, Martin Hruska, Thomas Lamb, Joanne Rothwell, Adele Fitzgerald, Gabriella Lindergard, Helen T-Michael, Tracey Duncan, Sharon Baxter-Dore, Lisa Cooper, Claire Fox, Jacinta Guerin, Tracey Hodgkiss, Karen Connolly, Paul McAlinden, Victoria Bridgett, Maggie Fearby, A. Gulati, Helen Hanson, Sinead Kelly, Louise McCormack, Rachel Nixon, Philip Robinson, Victoria Slater, Elaine Stephenson, Andrea Webster, K. Webster, Carole Hays, Anne Hudson, Bijal Patel, Ian Clement, John Davis, Sarah Francis, Douglas Jerry, Caroline Abernathy, Louise Foster, Andrew Gratrix, Llucia Cabral-Ortega, Matthew Hines, Victoria Martinson, Elizabeth Stones, Karen Winter, Esther Barrow, Katharine Wylie, Deborah Baines, Katie Birchall, Laurel Kolakaluri, Richard Clark, Anila Sukumaran, Craig Brandwood, Melanie Barker, Deborah Paripoorani, Lara Smith, Charlotte Taylor, Charlotte Downes, Melanie Hayman, Katie Riches, Priya Daniel, Deepak Subramanian, Kathleen Holding, Mary Hilton, Carly McDonald, Georgina Richardson, Georgia Halladay, Peter Harding, Amie Reddy, Ian Turner-Bone, Laura Wilding, Robert Parker, Michaela Lloyd, Leanne Smith, Charlie Kelly, Maria Lazo, Alan Neal, Olivia Walton, Julie Melville, Jay Naisbitt, Emily Bullock, Rosane Joseph, Sara Callam, Lisa Hudig, Jocelyn Keshet-Price, Katie Stammers, Karen Convery, Georgina Randell, Deirdre Fottrell-Gould, Esther Mwaura, Sara-Beth Sutherland, Richard Stewart, Louise Mew, Lynn Wren, Laura Thrasyvoulou, Heather Willis, James Scriven, Bridget Hopkins, Daniel Lenton, Abigail Roberts, Maria Bokhari, Rachael Lucas, Wendy McCormick, Jenny Ritzema, Vanessa Linnett, Amanda Sanderson, Helen Wild, Rebecca Flanagan, Robert Hull, Kat Rhead, Emma McKenna, Gareth Hughes, Jennifer Anderson, Kelly Jones, Scott Latham, Heather Riley, Martina Coulding, Martyn Clark, Jacqueline McCormick, Oliver Mercer, Darsh Potla, Hafiz Rehman, Heather Savill, Victoria Turner, Edward Jude, Susan Kilroy, Elena Apetri, Cathrine Basikolo, Bethan Blackledge, Laura Catlow, Matthew Collis, Reece Doonan, Jade Harris, Alice Harvey, Karen Knowles, Stephanie Lee, Diane Lomas, Chloe Lyons, Liam McMorrow, Angiy Michael, Jessica Pendlebury, Jane Perez, Maria Poulaka, Nicola Proudfoot, Kathryn Slevin, Vicky Thomas, Danielle Walker, Paul Dark, Bethan Charles, Danielle McLaughlan, Melanie Slaughter, Dan Horner, Kathryn Cawley, Tracy Marsden, Joyann Andrews, Emily Beech, Olugbenga Akinkugbe, Alasdair Bamford, Holly Belfield, Gareth A. L. Jones, Tara McHugh, Hamza Meghari, Samiran Ray, Ana Luisa Tomas, Lauran O’Neill, Mark Peters, Michael Bell, Sarah Benkenstein, Catherine Chisholm, Charlene Davies, Klaudia Kupiec, Caroline Payne, Joanna Halls, Hayley Blakemore, Elizabeth Goff, Kati Hayes, Kerry Smith, Deanna Stephens, Ruth Worner, Borislava Borislavova, Beverley Faulkner, Matt Thomas, Ruth Cookson, Emma Gendall, Georgina Larman, Rebecca Pope, Artur Smalira, Victoria Priestley, Tracey Cosier, Gemma Millen, James Rand, Natasha Schumacher, Roxana Sandhar, Heather Weston, Neil Richardson, Lucy Cooper, Cathy Jones, Ya-Wen Jessica Huang, Reni Jacob, Craig Denmade, Lewis McIntyre, Dawn Trodd, Jane Martin, Geoff Watson, Emily Bevan, Caroline Wreybrown, Shereen Bano, Ruth Bellwood, Michael Bentley, Matt Bromley, Lucy Gurr, Camilla Ledgard, Janet McGowan, Kate Pye, Kirsten Sellick, Amelia Stacey, Deborah Warren, Brian Wilkinson, Louise Akeroyd, Huma Shafique, James Morgan, Susan Shorter, Rachel Swinger, Emily Waters, Tom Lawton, Elizabeth Allan, Kate Darlington, Ffyon Davies, Llinos Davies, Jack Easton, Sumit Kumar, Richard Lean, Callum Mackay, Richard Pugh, Xinyi Qiu, Stephanie Rees, Jeremy Scanlon, Joanne Lewis, Daniel Menzies, Annette Bolger, Gwyneth Davies, Jennifer Davies, Esther Garrod, Helen Jones, Rachel Manley, Hannah Williams, Jordan Frankham, Sally Pitts, Nigel White, Debbie Branney, Heather Tiller, Georgia Efford, Zoe Garland, Lisa Grimmer, Bethany Gumbrill, Rebekah Johnson, Katie Sweet, Jeremy Bewley, Christina Coleman, Katie Corcoran, Eva Maria Hernandez Morano, Rachel Shiel, Denise Webster, Josephine Bonnici, Eleanor Daniel, Abbie Dell, Melanie Kent, Ami Wilkinson, Ellen Brown, Andrea Kay, Suzanne Campbell, Amanda Cowton, Mark Birt, Vicki Greenaway, Kathryn Potts, Clare Hutton, Andrew Shepperson, Miranda Forsey, Alice Nicholson, Mark Vertue, Joanne Riches, Agilan Kaliappan, Anne Nicholson, Niall MacCallum, Eamon Raith, Georgia Bercades, Ingrid Hass, David Brealey, Gladys Martir, Anna Reyes, Deborah Smyth, Maria Zapatamartinez, Ana Alvaro, Champa Jetha, Louise Ma, Lauren Booker, Loreta Mostoles, Anezka Pratley, Abdelhakim Altabaibeh, Chetan Parmar, Kayleigh Gilbert, Susie Ferguson, Amy Shepherd, Sheila Morris, Jo Singleton, Rosie Baruah, Maria Amamio, Sophie Birch, Kate Briton, Sarah Clark, Katherine Doverman, Lucy Marshall, Scott Simpson, Georgina Lloyd, Stephanie Bell, Vanessa Rivers, Bally Purewal, Kate Hammerton, Susan Anderson, Janine Birch, Emma Collins, Ryan Oleary, Sarah Cornell, Jordan Jarmain, Kimberley Rogerson, Fiona Wakinshaw, Lindsey Woods, Anthony Rostron, Zeynep Elcioglu, Alistair Roy, Gillian Bell, Holly Dickson, Louise Wilcox, Amro Katary, Katy English, Joanne Hutter, Corinne Pawley, Patricia Doble, Charmaine Shovelton, Marius Vaida, Rebecca Purnell, Ashly Thomas, Lenka Cagova, Adama Fofano, Helen Holcombe, Alice Michael Mitchell, Lucy Mwaura, Krithivasan P. Raman, Lucie Garnr, Sue Mepham, Kitty Paques, Alain Vuylsteke, Jennifer Mackie, Carmen Pearn, Julie Zamikula, Mark Birt, Estefania Treus Gude, Maggie Nyirenda, Lisa Capozzi, Rosie Reece-Anthony, Waqas Khaliq, Hazma Noor, Alfa Cresia Nilo, Michelle Grove, Amelia Daniel, Amy Easthope, Joanne Finn, Nikki White, Rajnish Saha, Bibi Badal, Karen Ixer, Donna Duffin, Ben Player, Helen Hill, Jade Cole, Jenny Brooks, Michelle Davies, Rhys Davies, Lauren Hunt, Emma Thomas, Angharad Williams, Metod Oblak, Mini Thankachen, Jamie Irisari, Amrinder Sayan, Monica Popescu, Cheryl Finch, Andrew Jamieson, Alison Quinn, Joshua Cooper, Sarah Liderth, Natalia Waddington, Iona Burn, Katarina Manso, Ruth Penn, Julie Tebbutt, Danielle Thornton, James Winchester, Geraldine Hambrook, Pradeep Shanmugasundaram, Jayne Craig, Kerry Simpson, Andrew Higham, Louise Sibbett, Sheila Paine, Annabel Reed, Jo-Anna Conyngham, McDonald Mupudzi, Rachel Thomas, Mary Wright, Denise Griffin, Richard Partridge, Maria Alvarez Corral, Nycola Muchenje, Mildred Sitonik, Caroline Wrey Brown, Aaron Butler, Linda Folkes, Heather Fox, Amy Gardner, David Helm, Gillian Hobden, Kirsten King, Jordi Margalef, Michael Margarson, Tim Martindale, Emma Meadows, Dana Raynard, Yvette Thirlwall, Yolanda Baird, Raquel Gomez, Darren Martin, Luke Hodgson, Clinton Corin, Erikka Sidall, Densie Szabo, Sharon Floyd, Hannah Davies, Karen Austin, Olivia Kelsall, Hannah Wood, Peter Anderson, Katie Archer, Andrew Burtenshaw, Sarah Clayton, Naiara Cother, Nicholas Cowley, Caroline Davis, Stephen Digby, Alison Durie, Alison Harrison, Emma Low, Michael McAlindon, Alex McCurdy, Aled Morgan, Tobias Rankin, Jessica Thrush, Helen Tranter, Charlie Vigurs, Laura Wild, Thomas Cornell, Kate Ralph, Sarah Bean, Karen Burt, Michael Spivey, Carol Richards, Rachel Tedstone, Siobhain Carmody, Xiaobei Zhao, Valerie Page, Mark Louie Guanco, Elvira Hoxha, Camilla Zorloni, Charlotte Dean, Emma Jones, Emma Carter, Joshua Dunn, Thomas Kong, Mervin Mahenthran, Chris Marsh, Maureen Holland, Natalie Keenan, Mohamed Mahmoud, Marc Lyons, Joanne Bradley-Potts, Helen Wassall, Meghan Young, Paul Bradley, Dorota Burda, Sinead Donlon, Lesley Harden, Celia Harris, Irving Mayangao, Rugia Montaser, Sheila Mtuwa, Charles Piercy, Eleanor Smith, Sarah Stone, Jerik Verula, Helen Blackman, Cheryl Marriott, Natalia Michalak, Ben Creagh-Brown, Armorel Salberg, Naomi Boyer, Veronika Pristopan, Victoria Maynard, Rachel Walker, Anil Hormis, Dawn Collier, Cheryl Graham, Vicky Maynard, Jake McCormick, Jake Warrington, Denise Cosgrove, Denise McFarland, Judith Ratcliffe, Rob Charnock, Inez Wynter, Mandy Gill, Jill Kirk, Paul Paul, Valli Ratnam, Sarah Shelton, Catherine Jardine, Alasdair Hay, Dewi Williams, Bethan Deacon, Latha Durga, Meg Hibbert, Gareth Kennard-Holden, Chrsitopher Woodford, Carla Pothecary, Lisa Roche, Dariusz Tetla, Kevin Agravante, Justyna Smeaton, Alicia Price, Alice Thomas, Chris Thorpe, Ellen Knights, Donna Ward, Shondipon Laha, Mark Verlander, Alexandra Williams, Rachel Prout, Helen Langton, Malcolm Watters, Charlotte Hunt, Catherine Novis, Sarwat Arif, Amy Cunningham, Claire Hewitt, Julia Hindale, Karen Jackson-Lawrence, Sarah Shepardson, Maryanne Wills, Susie Butler, Silivia Tavares, Russell Barber, Annette Hilldrith, Kelly Hubbard, Dawn Egginton, Michele Clark, Sarah Purvis, Simon Sinclair, Vicky Collins, Bethan Landeg, Craig Sell, Samantha Coetzee, Alistair Gales, Igor Otahal, Becky Icke, Meena Raj, Caroline Williams, Jill Williams, Lucy Hill, Abdul Kayani, Bridgett Masunda, Prisca Gondo, Nigara Atayeva, Carina Cruz, Natalie Pattison, Caroline Burnett, Jonathan Hatton, Elaine Heeney, Maria Newton, Hassan Al-Moasseb, Teresa Behan, Jasmine Player, Rachael Stead, Atideb Mitra, Kirsty Nauyokas, Sally Humphreys, Helen Cockerill, Ruth Tampsett, Evgeniya Postovalova, Tina Coventry, Amanda McGregor, Susan Fowler, Mike Macmahon, Patricia Cochrane, Sandra Pirie, Sarah Hanley, Asifa Ali, Megan Brady, Sam Dale, Annalisa Dance, Lisa Gledhill, Jill Greig, Kathryn Hanson, Kelly Holdroyd, Marie Home, Tahira Ishaq, Diane Kelly, Lear Matapure, Deborah Melia, Samantha Mellor, Ekta Merwaha, Tonicha Nortcliffe, Lisa Shaw, Ryan Shaw, Tracy Wood, Lee-Ann Bayo, Miranda Usher, Alison Wilson, Ross Kitson, Jez Pinnell, Matthew Robinson, Kaitlin Boltwood, Jenny Birch, Laura Bough, Rebecca Tutton, Barbara Winter-Goodwin, Josie Goodsell, Kate Taylor, Patricia Williams, Sarah Williams, Ashleigh Cave, James Rees, Janet Imeson-Wood, Jacqueline Smith, Vishal Amin, Komala Karthik, Rizwana Kausar, Elena Anastasescu, Karen Reid, Vikram Anumakonda, Ella Stoddart, Carrie Demetriou, Charlotte Eckbad, Lucy Howie, Sarah Mitchard, Lidia Ramos, Katie White, Sarah Hierons, Fiona Kelly, Alfredo Serrano-Ruiz, Gabrielle Evans, Liz Nicol, Joy Wilkins, Kim Hulacka, Gabor Debreceni, Alison Brown, Vikki Crickmore, Kay Hill, Thogulava Kannan, Zenaida Dagutao, Kate Beesley, Alison Lewis, Jess Perry, Sherly Antony, Sarah Board, Clare Buckley, Lucy Pippard, Alfonso Tanate, Diane Wood, Agnieska Kubisz-Pudelko, Ayman Gouda, Fiona Auld, Joanne Donnachie, Euan Murdoch, Lynn Prentice, Nikole Runciman, Dhaneesha Senaratne, Abigail Short, Laura Sweeney, Lesley Symon, Anne Todd, Patricia Turner, Erin McCann, Dario Salutous, Ian Edmond, Lesley Whitelaw, Harish Venkatesh, Yvonne Bland, Istvan Kajtor, Lisa Kavanagh, Karen Singler, George Linfield-Brown, Luke Stephen Prockter Moore, Marcela Vizcaychipi, Laura Martins, Luke Moore, Rhian Bull, Jaime Carungcong, Louise Allen, Eva Beranova, Alicia Knight, Carly Price, Sorrell Tilbey, Sharon Turney, Tracy Hazelton, Gabriella Tutt, Mansi Arora, Salah Turki, Emily Sinfield, Joanne Deery, Hazel Ramos, Daniele Cristiano, Natalie Dormand, Zohreh Farzad, Mahitha Gummadi, Sara Salmi, Geraldine Sloane, Mathew Varghese, Vicky Thwaites, Brijesh Patel, Liyanage Kamal, Anelise Catelan Zborowski, Ryan Coe, Madeleine Anderson, Jane Beadle, Charlotte Coates, Katy Collins, Maria Crowley, Laura Johnson, Laura King, Remi Paramsothy, Janet Sargeant, Pedro Silva, Carmel Stuart, June Taylor, David Tyl, Phillipa Wakefield, Charlotte Kamundi, Olumide Olufuwa, Zakaulla Belagodu, Anca Gherman, Naomi Oakley, John Allan, Tim Geary, Alistair Meikle, Peter O’Brien, Stephen Wood, Andrew Clark, Gordon Houston, Karen Black, Michelle Clarkson, Stuart D’Sylva, Alan Morrison, Kathryn Norman, Margaret Taylor, Suzanne Clements, Catriona Cohrane, Nora Gonzalez, Dominic Strachan, Claire Beith, Kirsten Moar, Lorna Murphy, Michelle Smythe, Alistair Nichol, Kathy Brickell, Inthakab Ali Mohamed Ali, Karen Beaumont, Mohamed Elsaadany, Kay Fernandes, Sameena Mohamed Ally, Harini Rangarajan, Varun Sarathy, Sivarupan Selvanayagam, Dave Vedage, Matthew White, Zoe Coton, Aricsa Joshy, Mark Blunt, Hollie Curgenven, Liam Botfield, Catherine Dexter, Aditya Kuravi, Joanne Butler, Robert Chadwick, Poonam Ranga, Lisa Richardson, Emma Virgilio, Maddiha Anwer, Atul Garg, Donna Botfield, Xana Marriott, Keely Stewart, Dee Mullan, Claire Phillips, Jane Gaylard, Justyna Nowak, Denise Skinner, Sian Jones, Rikki Crawley, Abigail Crew, Mishell Cunningham, Allison Daniels, Laura Harrison, Susan Hope, Nicola Lancaster, Jamie Matthews, Gemma Wray, Alice Nicholson, Ken Inweregbu, Sarah Cutts, Katharine Miller, Ailbhe Brady, Rebekah Chan, Shane McIvor, Helena Prady, Bijoy Mathew, Jeff Little, Tim Furniss, Chris Wright, Bernadette King, Christopher Wasson, Aisling O’Neill, Christine Turley, Peter McGuigan, Erin Collins, Stephanie Finn, Jackie Green, Julie McAuley, Abitha Nair, Charlotte Quinn, Suzanne Tauro, Kathryn Ward, Michael McGinlay, Kiran Reddy, Norfaizan Ahmad, Samantha Anderson, Joann Barker, Kris Bauchmuller, Kathryn Birchall, Sarah Bird, Kay Cawthron, Luke Chetam, Joby Cole, Ben Donne, David Foote, Amber Ford, Helena Hanratty, Kate Harrington, Lisa Hesseldon, Kay Housley, Yvonne Jackson, Claire Jarman, Faith Kibutu, Becky Lenagh, Irene Macharia, Shamiso Masuko, Leanne Milner, Helen Newell, Lorenza Nwafor, Simon Oxspring, Patrick Phillips, Ajay Raithatha, Sarah Rowland-Jones, Jacqui Smith, Roger Thompson, Helen Trower, Sara Walker, James Watson, Matthew Wiles, Alison Lye, Jayne Willson, Gary Mills, Sansha Harris, Eleanor Hartill, Anthony Barron, Ciara Collins, Sundeep Kaul, Claire Nolan, Oliver Polgar, Claire Prendergast, Paula Rogers, Rajvinder Shokkar, Meriel Woodruff, Kanta Mahay, Vicky Thwaites, Anna Reed, Hayley Meyrick, Heather Passmore, James Farwell, Alison Brown, Susan O’Connell, Jane Gregory, Luigi Barberis, Rosemary Harper, Tim Smith, Diane Armstrong, Angie Bowey, Anne Cowley, Andrew Corner, Judith Highgate, Claire Rutherfurd, Jo-Anne Taylor, Sarah Goodwin, Claire Rutherford, Beena Eapen, Fiona Trim, Phil Donnison, Lisa Armstrong, Hayley Bates, Emma Dooks, Fiona Farquhar, Amy Kitching, Chantal McParland, Sophie Packham, Brigid Hairsine, Anand Patil, Premetie Andreou, Dawn Hales, Megha Mathews, Rekha Patel, Peter Barry, Neil Flint, Jessica Hailstone, Navneet Ghuman, Bethany Leonard, Rachel Lees, Deborah Butcher, Katy Leng, Nicola Butterworth-Cowin, Susie O’Sullivan, Alison Ghosh, Emma Williams, Colene Adams, Anita Agasou, Tracie Arden, Mandy Beekes, Amy Bowes, Pauline Boyle, Heather Button, Mandy Carnahan, Anne Carter, Danielle Childs, Jane Gaylard, Fran Hurford, Yasmin Hussain, Ayesha Javaid, James Jones, Michael Leigh, Terry Martin, Helen Millward, Nichola Motherwell, Dee Mullan, Julie Newman, Rachel Rikunenko, Jo Stickley, Julie Summers, Louise Ting, Helen Tivenan, Denise Donaldson, Nigel Capps, Emily Cale, Sanal Jose, Wendy Osbourne, Susie Pajak, Jayne Rankin, Louise Tonks, Tracy Baird, Margaret Harkins, Jim Ruddy, Joe West, Joseph Duffield, Lewis Mallon, Oliver Smith, Sara Smuts, Andy Campbell, Cate Davies, Sarah Davies, Rachel Hughes, Lisa Jobes, Victoria Whitehead, Clare Watkins, Fiona Bowman, Barry Milligan, Liane McPherson, Stella Metherell, Nichola Harris, Victoria Lake, Elizabeth Radford, Andy Smallwood, Shameer Gopal, Katherine Vassell, Dina Bell, Rosalind Boyle, Katie Douglas, Lynn Glass, Liz Lennon, Austin Rattray, Claire Beith, Emma Lee, Danielle Jones, Penny Parsons, Ben Attwood, Paul Jefferson, Mohan Ranganathan, Inderjit Atwal, Bridget Campbell, Angela Day, Camilla Stagg, Emma Haynes, Cecilia Ahmed, Sarah Clamp, Julie Colley, Risna Haq, Anne Hayes, Sibet Joseph, Zahira Maqsood, Samia Hussain, Jonathan Hulme, Patience Domingos, Rita Kumar, Manjit Purewal, Becky Taylor, Lara Bunni, Monica Latif, Claire Jennings, Shilu Jose, Rebecca Marshall, Aleksandra Metryka, Gayathri Subramanian, Adam Burgoyne, Susan O’Connell, Amanda Tyler, Joanne Waldron, Paula Hilltout, Jayne Evitts, Geraldine Ward, Pamela Bremmer, Carl Hawkins, Sophie Jackman, Michal Ogorek, Kylie Ashby, Lorraine Thornton, Pauline Mercer, Matthew Halkes, Adam Revill, Bryony Saint, Jo Fletcher, Kimberley Netherton, Manish Chablani, Amy Kirkby, Amanda Roper, Kinga Szymiczek, Isobel Sutherland, Linda O’Brien, Igor Otahal, Joanne Connell, Kim Davies, Tracy Lewis, Zohra Omar, Emma Perkins, Lisa Roche, Sonia Sathe, Ellie Davies, Alex Lyon, Isheunesu Mapfunde, Charlotte Willis, Rachael Hitchcock, Kathryn Hall, Christopher King, Andrew Fagan, Roonak Nazari, Lucy Worsley, Suzanne Allibone, Vidya Kasipandian, Amit Patel, Parisa Cutting, Roman Genetu, Ainhi Mac, Anthony Murphy, Sinead Ward, Fatima Butt, Amanda Ayers, Wendy Harrison, Katherine Mackintosh, Julie North, Lydia Ashton, Rehana Bi, Samantha Owen, Helen Winmill, Barney Scholefield, Hannah Blowing, Erin Williams, Michaela Duskova, Michelle Edwards, Alun Rees, Helen Thomas, Rachel Hughes, Igor Otahal, Jolene Brooks, Janet Phipps, Suzanne Brooks, Catherine Dennis, Vicki Parris, Sinduya Srikaran, Anisha Sukha, Alistair McGregor, Gerlynn Tiongson, Katie Adams, Benedict Andrew, Adam Brayne, Sasha Carter, Louise Findlay, Emma Fisher, Peter Jackson, Duncan Kaye, Juliet Parkin, Victoria Tuckey, Jane Hunt, Nicholas Love, Lynne van Koutrick, Ashley Hanson, Kathy Dent, Elizabeth Horsley, Sandra Pearson, Sue Spencer, Dorothy Hutchinson, Jasmine Player, Dorota Potoczna, Muhammad Nauman Akhtar, Lisa-Jayne Cottam, Kirsty Nauyokas, Jack Sanders, Sara Mingo Garcia, Glykeria Pakou, Cynthia Diaba, Helder Filipe, Lincy John, Amitaa Maharajh, Mark de Neef, Daniel Martin, Christine Eastgate, Poh Choo Teoh, Fiona Barrett, Clare Bradley, Avril Donaldson, Mairi Mascarenhas, Marianne O’Hara, Laura Okeefe, Noreen Clarke, Jonathan Whiteside, Rachael Campbell, Joanna Matheson, Deborah McDonald, Donna Patience, Polly Rice, Tim Smith, Melanie Clapham, Rachel Mutch, Luigi Barberis, Rosemary Harper, Hannah Craig, Una Poultney, Karen Burns, Andrew Higham, Sophie Twiss, Janet Barton, Linsha George, Clare Harrop, Sherly Mathew, David Justin Wright, Rachel Harrison, Jordan Toohie, Ben Chandler, Alison Turnbull, Janine Mallinson, Kerry Elliott, Rebecca Wolf-Roberts, Helen Tench, Igor Otahal, Maria Hobrok, Ronda Loosley, Heather McGuinness, Tanya Sims, Deborah Afolabi, Kathryn Sian Allison, Taya Anderson, Rachael Dore, Dawn Jones, Naomi Rogers, Paula Saunderson, Jennifer Whitbread, Laura O’Malley, Laura Rad, Daniel Hawcutt, Jonathan Aldridge, Melanie Tolson, Sweyn Garrioch, Joanne Tomlinson, Michael Grosdenier, David Loader, Ritoo Kapoor, Gemma Hector, Joslan Scherewode, Chunda Sri-Chandana, Lorraine Stephenson, Sarah Marsh, Arnold Dela Rosa, Shaman Jhanji, Thomas Bemand, Ryan Howle, Ravishankar Rao Baikady, Benjamin Thomas, Ethel Black, Kate Tatham, Sambasivarao Gurram, Ekaterina Watson, Vicki Parris, Sheena Quaid, Alistair McGregor, Anne Saunderson, Rachel O’Brien, Sam Moultrie, Jen Service, Clare Cheyne, Miranda Odam, Alison Wiliams, Nicky Barnes, Peter Csabi, Joana Da Rocha, Louika Glynou, Amy Huffenberger, Jade Bryant, Amy Pickard, Nicholas Roe, Arianna Bellini, Anton Mayer, Amy Burrow, Natalie Colley, Jayne Evans, Alex Howlett, Zeinab Khalifeh, Jerldine Pryce, Claire Gorman, Amy Easthope, Rebecca Brady, Elizabeth Timlick, Pierre Antoine, Abhinhav Gupta, John Hardy, Henry Houlden, Eleanor Moncur, Arianna Tucci, Eamon Raith, Ambreen Tariq, David Brealey, Emma Tagliavini, Becky Ramsay, Katy Fidler, Kevin Donnelly, Rebecca Hollis, Jocelyn Barr, Elizabeth Boyd, Val Irvine, Ben Shelley, Julie Buckley, Charlene Hamilton, Kathryn Valdeavella, SCOURGE Consortium, Javier Abellan, René Acosta-Isaac, Jose María Aguado, Carlos Aguilar, Sergio Aguilera-Albesa, Abdolah Ahmadi Sabbagh, Jorge Alba, Sergiu Albu, Karla A. M. Alcalá-Gallardo, Julia Alcoba-Florez, Sergio Alcolea Batres, Holmes Rafael Algarin-Lara, Virginia Almadana, Julia Almeida, Berta Almoguera, María R. Alonso, Nuria Alvarez, Yady Álvarez-Benítez, Felipe Álvarez-Navia, Rodolfo Alvarez-Sala Walther, Álvaro Andreu-Bernabeu, Maria Rosa Antonijoan, Eunate Arana-Arri, Carlos Aranda, Celso Arango, Carolina Araque, Nathalia K. Araujo, Izabel M. T. Araujo, Ana C. Arcanjo, Ana Arnaiz, Francisco Arnalich Fernández, María J. Arranz, José Ramon Arribas Lopez, Maria-Jesus Artiga, Yubelly Avello-Malaver, Carmen Ayuso, Ana Margarita Baldión-Elorza, Belén Ballina Martín, Raúl C. Baptista-Rosas, Andrea Barranco-Díaz, María Barreda-Sánchez, Viviana Barrera-Penagos, Moncef Belhassen-Garcia, Enrique Bernal, David Bernal-Bello, Joao F. Bezerra, Marcos A. C. Bezerra, Natalia Blanca-López, Rafael Blancas, Lucía Boix-Palop, Alberto Borobia, Elsa Bravo, María Brion, Óscar Brochado-Kith, Ramón Brugada, Matilde Bustos, Alfonso Cabello, Juan J. Caceres-Agra, Esther Calbo, Enrique J. Calderón, Shirley Camacho, Marcela C. Campos, Yolanda Cañadas, Cristina Carbonell, Servando Cardona-Huerta, Antonio Augusto F. Carioca, Maria Sanchez Carpintero, Carlos Carpio Segura, Thássia M. T. Carratto, José Antonio Carrillo-Avila, Maria C. C. Carvalho, Carlos Casasnovas, Luis Castano, Carlos F. Castaño, Jose E. Castelao, Aranzazu Castellano Candalija, María A. Castillo, Francisco C. Ceballos, Jessica G. Chaux, Walter G. Chaves-Santiago, Sylena Chiquillo-Gómez, Marco A. Cid-Lopez, Oscar Cienfuegos-Jimenez, Rosa Conde-Vicente, M. Lourdes Cordero-Lorenzana, Dolores Corella, Almudena Corrales, Jose L. Cortes-Sanchez, Marta Corton, Tatiana X. Costa, Raquel Cruz, Marina S. Cruz, Luisa Cuesta, Gabriela C. R. Cunha, Gabriela V. da Silva, David Dalmau, Raquel C. S. Dantas-Komatsu, M. Teresa Darnaude, Raimundo de Andrés, Jéssica N. G. de Araújo, Carmen de Juan, Juan De la Cruz Troca, Carmen de la Horra, Ana B. de la Hoz, Alba De Martino-Rodríguez, Julianna Lys de Sousa Alves Neri, Victor del Campo-Pérez, Juan Delgado-Cuesta, Covadonga M. Diaz-Caneja, Anderson Díaz-Pérez, Aranzazu Diaz de Bustamante, Beatriz Dietl, Silvia Diz-de Almeida, Manoella do Monte Alves, Elena Domínguez-Garrido, Katiusse A. dos Santos, Alice M. Duarte, Jose Echave-Sustaeta, Rocío Eiros, César O. Enciso-Olivera, Gabriela Escudero, Pedro Pablo España, Gladys Mercedes Estigarribia Sanabria, María Carmen Fariñas, Marianne R. Fernandes, Ramón Fernández, Lidia Fernandez-Caballero, Ana Fernández-Cruz, María J. Fernandez-Nestosa, Uxía Fernández-Robelo, Amanda Fernández-Rodríguez, Marta Fernández-Sampedro, Ruth Fernández-Sánchez, Tania Fernández-Villa, Silvia Fernández Ferrero, Yolanda Fernández Martínez, Carmen Fernéndez Capitán, Patricia Flores-Pérez, Vicente Friaza, Lácides Fuenmayor-Hernández, Marta Fuertes Núñez, Victoria Fumadó, Ignacio Gadea, Lidia Gagliardi, Manuela Gago-Domínguez, Natalia Gallego, Cristina Galoppo, Inés García, Mercedes García, Leticia García, Carlos Garcia-Cerrada, Aitor García-de-Vicuña, Josefina Garcia-García, Irene García-García, Carmen García-Ibarbia, Andrés C. García-Montero, Ana García-Soidán, Elisa García-Vázquez, María Carmen García Torrejón, Emiliano Garza-Frias, Angela Gentile, Belén Gil-Fournier, Javier Gómez-Arrue, Mario Gómez-Duque, Luis Gómez Carrera, María Gómez García, Ángela Gómez Sacristán, Anna González-Neira, Javier González-Peñas, Manuel Gonzalez-Sagrado, Beatriz González Álvarez, Fernan Gonzalez Bernaldo de Quirós, Hugo Gonzalo Benito, Oscar Gorgojo-Galindo, Miguel Górgolas, Florencia Guaragna, Genilson P. Guegel, Beatriz Guillen-Guio, Encarna Guillen-Navarro, Pablo Guisado-Vasco, Juan F. Gutiérrez-Bautista, Luz D. Gutierrez-Castañeda, Sarah Heili-Frades, Estefania Hernandez, Luis D. Hernandez-Ortega, Guillermo Hernández-Pérez, Rebeca Hernández-Vaquero, Cristina Hernández Moro, Belen Herraez, M. Teresa Herranz, María Herrera, María José Herrero, Antonio Herrero-Gonzalez, Juan P. Horcajada, Natale Imaz-Ayo, Maider Intxausti-Urrutibeaskoa, María Íñiguez, Rafael H. Jacomo, Rubén Jara, Perez Maria Jazmin, Ángel Jiménez, Pilar Jiménez, Ignacio Jiménez-Alfaro, María A. Jimenez-Sousa, Iolanda Jordan, Rocío Laguna-Goya, Daniel Laorden, María Lasa-Lazaro, María Claudia Lattig, Ailen Lauriente, Anabel Liger Borja, Lucía Llanos, Amparo López-Bernús, Esther Lopez-Garcia, Rosario Lopez-Rodriguez, Miguel A. López-Ruz, Eduardo López Granados, Leonardo Lorente, José E. Lozano, María Lozano-Espinosa, Andre D. Luchessi, Ignacio Mahillo, Esther Mancebo, Carmen Mar, Cristina Marcelo Calvo, Miguel Marcos, Alba Marcos-Delgado, Alicia Marín Candon, Pablo Mariscal Aguilar, María M. Martín, María Dolores Martín, Vicente Martín, Marta Martin-Fernandez, Caridad Martín-López, José-Ángel Martín-Oterino, Laura Martin-Pedraza, María Martín-Vicente, Amalia Martinez, Ricardo Martínez, Juan José Martínez, Silvia Martínez, Eleno Martínez-Aquino, Óscar Martínez-González, Iciar Martinez-Lopez, Oscar Martinez-Nieto, Pedro Martinez-Paz, Angel Martinez-Perez, Andrea Martínez-Ramas, Michel F. Martinez-Resendez, Violeta Martínez Robles, Laura Marzal, Juliana F. Mazzeu, Jeane F. P. Medeiros, Kelliane A. Medeiros, Francisco J. Medrano, Xose M. Meijome, Natalia Mejuto-Montero, Ana Méndez-Echevarria, Humberto Mendoza Charris, Eleuterio Merayo Macías, Fátima Mercadillo, Arieh R. Mercado-Sesma, Pablo Minguez, Antonio J. J. Molina, Elena Molina-Roldán, Juan José Montoya, Vitor M. S. Moraes, Patricia Moreira-Escriche, Xenia Morelos-Arnedo, Antonio Moreno-Docón, Junior Moreno-Escalante, Victor Moreno Cuerda, Alberto Moreno Fernández, Rubén Morilla, Patricia Muñoz García, Pablo Neira, Julian Nevado, Israel Nieto-Gañán, Joana F. R. Nunes, Rocio Nuñez-Torres, Antònia Obrador-Hevia, J. Gonzalo Ocejo-Vinyals, Virginia Olivar, Silviene F. Oliveira, Lorena Ondo, Alberto Orfao, Luis Ortega, Eva Ortega-Paino, Fernando Ortiz-Flores, Rocio Ortiz-Lopez, José A. Oteo, Harry Pachajoa, Manuel Pacheco, Fredy Javier Pacheco-Miranda, Irene Padilla Conejo, Sonia Panadero-Fajardo, Mara Parellada, Roberto Pariente-Rodríguez, Estela Paz-Artal, Germán Peces-Barba, Miguel S. Pedromingo Kus, Celia Perales, Patricia Perez, César Pérez, Gustavo Perez-de-Nanclares, Felipe Pérez-García, Patricia Pérez-Matute, Alexandra Pérez-Serra, M. Elena Pérez-Tomás, Teresa Perucho, Lisbeth A. Pichardo, Susana M. T. Pinho, Mel·lina Pinsach-Abuin, Luz Adriana Pinzón, Guillermo Pita, Francesc Pla-Junca, Laura Planas-Serra, Ericka N. Pompa-Mera, Gloria L. Porras-Hurtado, Aurora Pujol, María Eugenia Quevedo Chávez, Maria Angeles Quijada, Inés Quintela, Diana Ramirez-Montaño, Soraya Ramiro León, Pedro Rascado Sedes, Delia Recalde, Emma Recio-Fernández, Salvador Resino, Adriana P. Ribeiro, Carlos S. Rivadeneira-Chamorro, Diana Roa-Agudelo, Montserrat Robelo Pardo, Marilyn Johanna Rodriguez, Fernando Rodriguez-Artalejo, Marena Rodríguez-Ferrer, Carlos Rodriguez-Gallego, José A. Rodriguez-Garcia, María A. Rodriguez-Hernandez, Antonio Rodriguez-Nicolas, Agustí Rodriguez-Palmero, Emilio Rodríguez-Ruiz, Paula A. Rodriguez-Urrego, Belén Rodríguez Maya, German Ezequiel Rodriguez Novoa, Federico Rojo, Andrea Romero-Coronado, Filomeno Rondón García, Lidia S. Rosa, Antonio Rosales-Castillo, Cladelis Rubio, María Rubio Olivera, Montserrat Ruiz, Francisco Ruiz-Cabello, Eva Ruiz-Casares, Juan J. Ruiz-Cubillan, Javier Ruiz-Hornillos, Pablo Ryan, Hector D. Salamanca, Lorena Salazar-García, Giorgina Gabriela Salgueiro Origlia, Pedro-Luis Sánchez, Clara Sánchez-Pablo, Olga Sánchez-Pernaute, Antonio J. Sánchez López, María Concepción Sánchez Prados, Javier Sánchez Real, Jorge Sánchez Redondo, Cristina Sancho-Sainz, Anna Sangil, Arnoldo Santos, Ney P. C. Santos, Agatha Schlüter, Sonia Segovia, Alex Serra-Llovich, Fernando Sevil Puras, Marta Sevilla Porras, Miguel A. Sicolo, Vivian N. Silbiger, Nayara S. Silva, Fabiola T. C. Silva, Cristina Silván Fuentes, Jordi Solé-Violán, José Manuel Soria, Jose V. Sorlí, Renata R. Sousa, Juan Carlos Souto, Karla S. C. Souza, Vanessa S. Souza, John J. Sprockel, José Javier Suárez-Rama, David A. Suarez-Zamora, Xiana Taboada-Fraga, Eduardo Tamayo, Alvaro Tamayo-Velasco, Juan Carlos Taracido-Fernandez, Nathali A. C. Tavares, Carlos Tellería, Jair Antonio Tenorio Castaño, Alejandro Teper, Juan Torres-Macho, Lilian Torres-Tobar, Ronald P. Torres Gutiérrez, Jesús Troya, Miguel Urioste, Juan Valencia-Ramos, Agustín Valido, Juan Pablo Vargas Gallo, Belén Varón, Romero H. T. Vasconcelos, Tomas Vega, Santiago Velasco-Quirce, Valentina Vélez-Santamaría, Virginia Víctor, Julia Vidán Estévez, Miriam Vieitez-Santiago, Carlos Vilches, Lavinia Villalobos, Felipe Villar, Judit Villar-Garcia, Cristina Villaverde, Pablo Villoslada-Blanco, Ana Virseda-Berdices, Zuleima Yáñez, Antonio Zapatero-Gaviria, Ruth Zarate, Sandra Zazo, Miguel López de Heredia, Ingrid Mendes, Rocío Moreno, Esther Sande, Pablo Lapunzina, Angel Carracedo, ISARICC Investigators, Beatrice Alex, Petros Andrikopoulos, Benjamin Bach, Wendy S. Barclay, Debby Bogaert, Meera Chand, Kanta Chechi, Graham S. Cooke, Ana da Silva Filipe, Thushan de Silva, Annemarie B. Docherty, Gonçalo dos Santos Correia, Marc-Emmanuel Dumas, Jake Dunning, Tom Fletcher, Christopher A. Green, William Greenhalf, Julian Griffin, Rishi K. Gupta, Ewen M. Harrison, Antonia Y. W. Ho, Karl Holden, Peter W. Horby, Samreen Ijaz, Say Khoo, Paul Klenerman, Andrew Law, Matthew Lewis, Sonia Liggi, Wei Shen Lim, Lynn Maslen, Alexander J. Mentzer, Laura Merson, Alison M. Meynert, Shona C. Moore, Mahdad Noursadeghi, Michael Olanipekun, Anthonia Osagie, Massimo Palmarini, Carlo Palmieri, William A. Paxton, Georgios Pollakis, Nicholas Price, Andrew Rambaut, David L. Robertson, Clark D. Russell, Vanessa Sancho-Shimizu, Caroline Sands, Janet T. Scott, Louise Sigfrid, Tom Solomon, Shiranee Sriskandan, David Stuart, Olivia V. Swann, Zoltan Takats, Panteleimon Takis, Richard S. Tedder, A. A. Roger Thompson, Emma C. Thomson, Ryan S. Thwaites, Lance C. W. Turtle, Maria Zambon, Gail Carson, Thomas M. Drake, Cameron J. Fairfield, Stephen R. Knight, Kenneth A. Mclean, Derek Murphy, Lisa Norman, Riinu Pius, Catherine A. Shaw, Marie Connor, Jo Dalton, Carrol Gamble, Michelle Girvan, Sophie Halpin, Janet Harrison, Clare Jackson, Laura Marsh, Stephanie Roberts, Egle Saviciute, Susan Knight, Eva Lahnsteiner, Gary Leeming, Lucy Norris, James Scott-Brown, Sarah Tait, Murray Wham, James Lee, Daniel Plotkin, Seán Keating, Cara Donegan, Rebecca G. Spencer, Chloe Donohue, Hayley Hardwick, The 23andMe COVID-19 Team, Janie F. Shelton, Anjali J. Shastri, Chelsea Ye, Catherine H. Weldon, Teresa Filshtein-Sonmez, Daniella Coker, Antony Symons, Jorge Esparza-Gordillo, Stella Aslibekyan, Adam Auton, Jian Yang, Chris P. Ponting, James F. Wilson, Veronique Vitart, Malak Abedalthagafi, Andre D. Luchessi, Esteban J. Parra, Raquel Cruz, Angel Carracedo, Angie Fawkes, Lee Murphy, Kathy Rowan, Alexandre C. Pereira, Andy Law, Benjamin Fairfax, Sara Clohisey Hendry, J. Kenneth Baillie

**Affiliations:** 1 Baillie Gifford Pandemic Science Hub, Centre for Inflammation Research, The Queen’s Medical Research Institute, University of Edinburgh, Edinburgh, UK; 2 MRC Human Genetics Unit, Institute of Genetics and Cancer, University of Edinburgh, Western General Hospital, Edinburgh, UK; 3 Roslin Institute, University of Edinburgh, Edinburgh, UK; 4 Pain Service, NHS Tayside, Ninewells Hospital and Medical School, Dundee, UK; 5 School of Life Sciences, Westlake University, Hangzhou, China; 6 Westlake Laboratory of Life Sciences and Biomedicine, Hangzhou, China; 7 Institute for Molecular Bioscience, The University of Queensland, Brisbane, Queensland Australia; 8 Wellcome Centre for Human Genetics, University of Oxford, Oxford, UK; 9 Faculty of Biological Sciences, University of Leeds, Leeds, UK; 10 Genomics England, London, UK; 11 Intensive Care Unit, Royal Infirmary of Edinburgh, Edinburgh, UK; 12 National Heart and Lung Institute, Imperial College London, London, UK; 13 Edinburgh Clinical Research Facility, Western General Hospital, University of Edinburgh, Edinburgh, UK; 14 Department of Critical Care Medicine, Queen’s University and Kingston Health Sciences Centre, Kingston, Ontario Canada; 15 Clinical Research Centre at St Vincent’s University Hospital, University College Dublin, Dublin, Ireland; 16 NIHR Health Protection Research Unit for Emerging and Zoonotic Infections, Institute of Infection, Veterinary and Ecological Sciences University of Liverpool, Liverpool, UK; 17 Respiratory Medicine, Alder Hey Children’s Hospital, Institute in The Park, University of Liverpool, Alder Hey Children’s Hospital, Liverpool, UK; 18 Centre for Inflammation Research, The Queen’s Medical Research Institute, University of Edinburgh, Edinburgh, UK; 19 Department of Medicine, University of Cambridge, Cambridge, UK; 20 William Harvey Research Institute Barts and the London School of Medicine and Dentistry, Queen Mary University of London, London, UK; 21 Centre for Tropical Medicine and Global Health, Nuffield Department of Medicine, University of Oxford, Oxford, UK; 22 Department of Anaesthesia and Intensive Care, The Chinese University of Hong Kong, Prince of Wales Hospital, Hong Kong, China; 23 Wellcome-Wolfson Institute for Experimental Medicine, Queen’s University Belfast, Belfast, UK; 24 Department of Intensive Care Medicine, Royal Victoria Hospital, Belfast, UK; 25 UCL Centre for Human Health and Performance, London, UK; 26 Imperial College Healthcare NHS Trust, London, UK; 27 Royal Hospital for Children, Glasgow, UK; 28 Centre for Global Health Research, Usher Institute of Population Health Sciences and Informatics, Edinburgh, UK; 29 Genomics Division, Instituto Tecnológico y de Energías Renovables, Santa Cruz de Tenerife, Spain; 30 Research Unit, Hospital Universitario N.S. de Candelaria, Santa Cruz de Tenerife, Spain; 31 Centre for Biomedical Network Research on Respiratory Diseases (CIBERES), Instituto de Salud Carlos III, Madrid, Spain; 32 Department of Clinical Sciences, University Fernando Pessoa Canarias, Las Palmas de Gran Canaria, Spain; 33 IDIVAL, Santander, Spain; 34 Universidad de Cantabria, Santander, Spain; 35 Hospital U M Valdecilla, Santander, Spain; 36 Tecnologico de Monterrey, Escuela de Medicina y Ciencias de la Salud and Hospital San Jose TecSalud, Monterrey, Mexico; 37 Centre for Biomedical Network Research on Rare Diseases (CIBERER), Instituto de Salud Carlos III, Madrid, Spain; 38 Instituto de Genética Médica y Molecular (INGEMM), Hospital Universitario La Paz-IDIPAZ, Madrid, Spain; 39 ERN-ITHACA-European Reference Network, Paris, France; 40 Genomic Research Department, King Fahad Medical City, Riyadh, Saudi Arabia; 41 Department of Pathology & Laboratory Medicine, Emory University Hospital, Atlanta, GA USA; 42 Department of Clinical Analysis and Toxicology, Federal University of Rio Grande do Norte, Natal, Brazil; 43 Department of Anthropology, University of Toronto at Mississauga, Mississauga, Ontario Canada; 44 Centro Singular de Investigación en Medicina Molecular y Enfermedades Crónicas (CIMUS), Universidade de Santiago de Compostela, Santiago de Compostela, Spain; 45 Instituto de Investigación Sanitaria de Santiago (IDIS), Santiago de Compostela, Spain; 46 Fundación Pública Galega de Medicina Xenómica, Sistema Galego de Saúde (SERGAS) Santiago de Compostela, Santiago de Compostela, Spain; 47 Intensive Care National Audit & Research Centre, London, UK; 48 Heart Institute, University of Sao Paulo, Butanta, Brazil; 49 NIHR Clinical Research Network (CRN), North West London Core Team, Hammersmith Hospital, London, UK; 50 Cambridge University Hospitals NHS Foundation Trust, Cambridge, UK; 51 Biostatistics Group, State Key Laboratory of Biocontrol, School of Life Sciences, Sun Yat-sen University, Guangzhou, China; 52 Department of Infectious Diseases, Leiden University Medical Center, Leiden, The Netherlands; 53 Guys and St Thomas’ Hospital, London, UK; 54 James Cook University Hospital, Middlesbrough, UK; 55 Barts Health NHS Trust, London, UK; 56 Royal Stoke University Hospital, Stoke-on-Trent, UK; 57 North Middlesex University Hospital NHS Trust, London, UK; 58 King’s College Hospital, London, UK; 59 Charing Cross Hospital, St Mary’s Hospital and Hammersmith Hospital, London, UK; 60 The Royal Liverpool University Hospital, Liverpool, UK; 61 John Radcliffe Hospital, Oxford, UK; 62 Addenbrooke’s Hospital, Cambridge, UK; 63 Nottingham University Hospital, Nottingham, UK; 64 St George’s Hospital, London, UK; 65 BHRUT (Barking Havering)—Queens Hospital and King George Hospital, Ilford, UK; 66 Royal Infirmary of Edinburgh, Edinburgh, UK; 67 Kingston Hospital, London, UK; 68 Queen Alexandra Hospital, Portsmouth, UK; 69 Royal Gwent Hospital, Newport, UK; 70 Royal Blackburn Teaching Hospital, Blackburn, UK; 71 Stepping Hill Hospital, Stockport, UK; 72 Northumbria Healthcare NHS Foundation Trust, North Shields, UK; 73 Countess of Chester Hospital, Chester, UK; 74 Pinderfields General Hospital, Wakefield, UK; 75 Ninewells Hospital, Dundee, UK; 76 Croydon University Hospital, Croydon, UK; 77 Morriston Hospital, Swansea, UK; 78 Queen Elizabeth University Hospital, Glasgow, UK; 79 Broomfield Hospital, Chelmsford, UK; 80 Heartlands Hospital, Birmingham, UK; 81 Royal Sussex County Hospital, Brighton, UK; 82 York Hospital, York, UK; 83 Queen Elizabeth Hospital, Birmingham, UK; 84 Royal Glamorgan Hospital, Pontyclun, UK; 85 Barnet Hospital, London, UK; 86 Wythenshawe Hospital, Manchester, UK; 87 Medway Maritime Hospital, Gillingham, UK; 88 Royal Berkshire NHS Foundation Trust, Reading, UK; 89 Whiston Hospital, Prescot, UK; 90 The Royal Oldham Hospital, Manchester, UK; 91 Chesterfield Royal Hospital Foundation Trust, Chesterfield, UK; 92 Aberdeen Royal Infirmary, Aberdeen, UK; 93 Royal Devon and Exeter Hospital, Exeter, UK; 94 Glasgow Royal Infirmary, Glasgow, UK; 95 Blackpool Victoria Hospital, Blackpool, UK; 96 Southampton General Hospital, Southampton, UK; 97 Ashford and St Peter’s Hospital, Chertsey, UK; 98 Derriford Hospital, Plymouth, UK; 99 East Surrey Hospital, Redhill, UK; 100 Poole Hospital, Poole, UK; 101 Royal Alexandra Hospital, Paisley, UK; 102 St James’s University Hospital and Leeds General Infirmary, Leeds, UK; 103 Bedford Hospital, Bedford, UK; 104 Southport and Formby District General Hospital, Ormskirk, UK; 105 The Tunbridge Wells Hospital and Maidstone Hospital, Kent, UK; 106 Queen Elizabeth Hospital, Woolwich, London, UK; 107 North Manchester General Hospital, Manchester, UK; 108 Royal Victoria Infirmary, Newcastle Upon Tyne, UK; 109 Hull Royal Infirmary, Hull, UK; 110 Manchester Royal Infirmary, Manchester, UK; 111 Royal Derby Hospital, Derby, UK; 112 Aintree University Hospital, Liverpool, UK; 113 Fairfield General Hospital, Bury, UK; 114 Norfolk and Norwich University Hospital (NNUH), Norwich, UK; 115 Milton Keynes University Hospital, Milton Keynes, UK; 116 Good Hope Hospital, Birmingham, UK; 117 Queen Elizabeth Hospital Gateshead, Gateshead, UK; 118 Royal Bolton Hospital, Bolton, UK; 119 Tameside General Hospital, Ashton Under Lyne, UK; 120 Salford Royal Hospital, Manchester, UK; 121 Great Ormond St Hospital and UCL Great Ormond St Institute of Child Health NIHR Biomedical Research Centre, London, UK; 122 Southmead Hospital, Bristol, UK; 123 William Harvey Hospital, Ashford, UK; 124 Arrowe Park Hospital, Wirral, UK; 125 Royal Hampshire County Hospital, Winchester, UK; 126 Bradford Royal Infirmary, Bradford, UK; 127 Glan Clwyd Hospital, Bodelwyddan, UK; 128 Royal Bournemouth Hospital, Bournemouth, UK; 129 Bristol Royal Infirmary, Bristol, UK; 130 University Hospital North Durham, Darlington, UK; 131 Darlington Memorial Hospital, Darlington, UK; 132 Basildon Hospital, Basildon, UK; 133 University College Hospital, London, UK; 134 Whittington Hospital, London, UK; 135 Western General Hospital, Edinburgh, UK; 136 Ipswich Hospital, Ipswich, UK; 137 Hereford County Hospital, Hereford, UK; 138 Sunderland Royal Hospital, Sunderland, UK; 139 Queens Hospital Burton, Burton-On-Trent, UK; 140 Musgrove Park Hospital, Taunton, UK; 141 The Royal Papworth Hospital, Cambridge, UK; 142 University Hospital Lewisham, London, UK; 143 The Princess Alexandra Hospital, Harlow, UK; 144 University Hospital of Wales, Cardiff, UK; 145 West Middlesex Hospital, Isleworth, UK; 146 Royal Albert Edward Infirmary, Wigan, UK; 147 Stoke Mandeville Hospital, Aylesbury, UK; 148 Royal Lancaster Infirmary, Lancaster, UK; 149 Basingstoke and North Hampshire Hospital, Basingstoke, UK; 150 Worthing Hospital, Worthing, UK; 151 St Richard’s Hospital, Chichester, UK; 152 The Alexandra Hospital, Redditch and Worcester Royal Hospital, Worcester, UK; 153 Royal Cornwall Hospital, Truro, UK; 154 Watford General Hospital, Watford, UK; 155 Macclesfield District General Hospital, Macclesfield, UK; 156 Royal Surrey County Hospital, Guildford, UK; 157 Rotherham General Hospital, Rotherham, UK; 158 Craigavon Area Hospital, Portadown, UK; 159 King’s Mill Hospital, Nottingham, UK; 160 Dumfries and Galloway Royal Infirmary, Dumfries, UK; 161 Prince Charles Hospital, Merthyr Tydfil, UK; 162 Ysbyty Gwynedd, Bangor, UK; 163 Royal Preston Hospital, Preston, UK; 164 The Great Western Hospital, Swindon, UK; 165 Lincoln County Hospital, Lincoln, UK; 166 University Hospital of North Tees, Stockton on Tees, UK; 167 Glangwili General Hospital, Camarthen, UK; 168 Southend University Hospital, Westcliff-on-Sea, UK; 169 Lister Hospital, Stevenage, UK; 170 Diana Princess of Wales Hospital, Grimsby, UK; 171 West Suffolk Hospital, Bury St Edmunds, UK; 172 Victoria Hospital, Kirkcaldy, UK; 173 Calderdale Royal Hospital, Halifax, UK; 174 Huddersfield Royal Infirmary, Huddersfield, UK; 175 Dorset County Hospital, Dorchester, UK; 176 Russell’s Hall Hospital, Dudley, UK; 177 Royal United Hospital, Bath, UK; 178 St Mary’s Hospital, Newport, UK; 179 George Eliot Hospital NHS Trust, Nuneaton, UK; 180 Yeovil Hospital, Yeovil, UK; 181 Forth Valley Royal Hospital, Falkirk, UK; 182 Frimley Park Hospital, Camberley, UK; 183 Chelsea & Westminster NHS Foundation Trust, London, UK; 184 Queen Elizabeth the Queen Mother Hospital, Margate, UK; 185 Royal Brompton Hospital, London, UK; 186 Darent Valley Hospital, Dartford, UK; 187 University Hospital Crosshouse, Kilmarnock, UK; 188 University Hospital Wishaw, Wishaw, UK; 189 University College Dublin, St Vincent’s University Hospital, Dublin, Ireland; 190 The Queen Elizabeth Hospital, King’s Lynn, UK; 191 Walsall Manor Hospital, Walsall, UK; 192 Princess Royal Hospital, Brighton, UK; 193 Barnsley Hospital, Barnsley, UK; 194 Warrington General Hospital, Warrington, UK; 195 Royal Victoria Hospital, Belfast, UK; 196 Royal Hallamshire Hospital and Northern General Hospital, Sheffield, UK; 197 Harefield Hospital, London, UK; 198 Cumberland Infirmary, Carlisle, UK; 199 Eastbourne District General Hospital, Eastbourne, UK; 200 Conquest Hospital, Saint Leonards-on-Sea, UK; 201 Salisbury District Hospital, Salisbury, UK; 202 Airedale General Hospital, Keighley, UK; 203 Leicester Royal Infirmary, Leicester, UK; 204 Peterborough City Hospital, Peterborough, UK; 205 Hinchingbrooke Hospital, Huntingdon, UK; 206 Colchester General Hospital, Colchester, UK; 207 Princess Royal Hospital, Telford and Royal Shrewsbury Hospital, Shrewsbury, UK; 208 University Hospital Monklands, Airdrie, UK; 209 Wrexham Maelor Hospital, Wrexham, UK; 210 New Cross Hospital, Wolverhampton, UK; 211 University Hospital Hairmyres, East Kilbride, UK; 212 Warwick Hospital, Warwick, UK; 213 Sandwell General Hospital and City Hospital, Birmingham, UK; 214 Royal Manchester Children’s Hospital, Manchester, UK; 215 Gloucestershire Royal Hospital, Gloucester, UK; 216 University Hospitals Coventry & Warwickshire NHS Trust, Coventry, UK; 217 Torbay Hospital, Torquay, UK; 218 Pilgrim Hospital, Lincoln, UK; 219 Prince Philip Hospital, Lianelli, UK; 220 Princess of Wales Hospital, Llantrisant, UK; 221 Northampton General Hospital NHS Trust, Northampton, UK; 222 The Christie NHS Foundation Trust, Manchester, UK; 223 James Paget University Hospital NHS Trust, Great Yarmouth, UK; 224 Birmingham Children’s Hospital, Birmingham, UK; 225 Withybush General Hospital, Haverfordwest, UK; 226 Northwick Park Hospital, London, UK; 227 North Devon District Hospital, Barnstaple, UK; 228 Scunthorpe General Hospital, Scunthorpe, UK; 229 Royal Free Hospital, London, UK; 230 Raigmore Hospital, Inverness, UK; 231 West Cumberland Hospital, Whitehaven, UK; 232 Furness General Hospital, Barrow-in-Furness, UK; 233 Liverpool Heart and Chest Hospital, Liverpool, UK; 234 Scarborough General Hospital, Scarborough, UK; 235 Bronglais General Hospital, Aberystwyth, UK; 236 Alder Hey Children’s Hospital, Liverpool, UK; 237 Borders General Hospital, Melrose, UK; 238 Leighton Hospital, Crewe, UK; 239 Kent & Canterbury Hospital, Canterbury, UK; 240 Harrogate and District NHS Foundation Trust, Harrogate, UK; 241 The Royal Marsden Hospital, London, UK; 242 Ealing Hospital, Southall, UK; 243 St John’s Hospital Livingston, Livingston, UK; 244 Wexham Park Hospital, Slough, UK; 245 Sheffield Children’s Hospital, Sheffield, UK; 246 Homerton University Hospital Foundation NHS Trust, London, UK; 247 National Hospital for Neurology and Neurosurgery, London, UK; 248 The Royal Alexandra Children’s Hospital, Brighton, UK; 249 Golden Jubilee National Hospital, Clydebank, UK; 250 Hospital Universitario Mostoles, Medicina Interna, Madrid, Spain; 251 Universidad Francisco de Vitoria, Madrid, Spain; 252 Haemostasis and Thrombosis Unit, Hospital de la Santa Creu i Snt Pau, IIB Sant Pau, Barcelona, Spain; 253 Unit of Infectious Diseases, Hospital Universitario 12 de Octubre, Instituto de Investigación Sanitaria Hospital 12 de Octubre (imas12), Madrid, Spain; 254 Spanish Network for Research in Infectious Diseases (REIPI RD16/0016/0002), Instituto de Salud Carlos III, Madrid, Spain; 255 School of Medicine, Universidad Complutense, Madrid, Spain; 256 Centro de Investigación Biomédica en Red de Enfermedades Infecciosas (CIBERINFEC), Instituto de Salud Carlos III, Madrid, Spain; 257 Hospital General Santa Bárbara de Soria, Soria, Spain; 258 Pediatric Neurology Unit, Department of Pediatrics, Navarra Health Service Hospital, Pamplona, Spain; 259 Navarra Health Service, NavarraBioMed Research Group, Pamplona, Spain; 260 Complejo Asistencial Universitario de León, León, Spain; 261 Infectious Diseases Department, Hospital Universitario San Pedro, Logroño, Spain; 262 Fundación Institut Guttmann, Institut Universitari de Neurorehabilitació adscrit a la UAB, Hospital de Neurorehabilitació, Barcelona, Spain; 263 Universitat Autònoma de Barcelona (UAB), Barcelona, Spain; 264 Fundació Institut d’Investigació en Ciències de la Salut Germans Trias i Pujol, Barcelona, Spain; 265 Hospital General de Occidente, Guadalajara, Mexico; 266 Microbiology Unit, Hospital Universitario N.S. de Candelaria, Santa Cruz de Tenerife, Spain; 267 Servicio de Neumología, Hospital Universitario La Paz-IDIPAZ, Madrid, Spain; 268 Camino Universitario Adelita de Char, Mired IPS, Barranquilla, Colombia; 269 Facultad de Ciencias de la Salud, Universidad Simón Bolívar, Barranquilla, Colombia; 270 Neumología, Hospital Universitario Virgen Macarena, Seville, Spain; 271 Departamento de Medicina, Universidad de Salamanca, Salamanca, Spain; 272 Centro de Investigación del Cáncer (IBMCC), Universidad de Salamanca, CSIC, Salamanca, Spain; 273 Biomedical Research Institute of Salamanca (IBSAL), Salamanca, Spain; 274 Centre for Biomedical Network Research on Cancer (CIBERONC), Instituto de Salud Carlos III, Madrid, Spain; 275 Department of Genetics & Genomics, Instituto de Investigación Sanitaria–Fundación Jiménez Díaz University Hospital—Universidad Autónoma de Madrid (IIS-FJD, UAM), Madrid, Spain; 276 Human Genotyping—CEGEN Unit, Spanish National Cancer Research Centre, Madrid, Spain; 277 Servicio de Medicina Interna, Hospital Universitario de Salamanca-IBSAL, Salamanca, Spain; 278 Universidad de Salamanca, Salamanca, Spain; 279 Department of Child and Adolescent Psychiatry, Institute of Psychiatry and Mental Health, Hospital General Universitario Gregorio Marañón (IiSGM), Madrid, Spain; 280 Clinical Pharmacology Service, Hospital de la Santa Creu i Sant Pau, IIB Sant Pau, Barcelona, Spain; 281 Biocruces Bizkai HRI, Barakaldo, Spain; 282 Cruces University Hospital, Osakidetza, Barakaldo, Spain; 283 Hospital Infanta Elena, Madrid, Spain; 284 Instituto de Investigación Sanitaria–Fundación Jiménez Díaz University Hospital, Universidad Autónoma de Madrid (IIS-FJD, UAM), Madrid, Spain; 285 Centre for Biomedical Network Research on Mental Health (CIBERSAM), Instituto de Salud Carlos III, Madrid, Spain; 286 Fundación Hospital Infantil Universitario de San José, Bogotá, Colombia; 287 Fundación Universitaria de Ciencias de la Salud, Bogotá, Colombia; 288 Programa de Pós-graduação em Ciências da Saúde, Universidade Federal do Rio Grande do Norte, Natal, Brazil; 289 Departamento de Medicina Clínica, Universidade Federal do Rio Grande do Norte, Natal, Brazil; 290 Departamento de Genética e Morfologia, Instituto de Ciências Biológicas, Universidade de Brasília, Brasilia, Brazil; 291 Colégio Marista de Brasilia, Brasilia, Brazil; 292 Associação Brasileira de Educação e Cultura, Londrina, Brazil; 293 Servicio de Medicina Interna, Hospital Universitario La Paz-IDIPAZ, Madrid, Spain; 294 Fundació Docència I Recerca Mutua Terrassa, Barcelona, Spain; 295 Spanish National Cancer Research Center, CNIO Biobank, Madrid, Spain; 296 Departamento Patologia y Laboratorios, Fundación Santa Fe de Bogota, Bogotá, Colombia; 297 Hospital General de Occidente, Zapopan, Mexico; 298 Centro Universitario de Tonalá, Universidad de Guadalajara, Tonalá, Mexico; 299 Centro de Investigación Multidisciplinario en Salud, Universidad de Guadalajara, Guadalajara, Mexico; 300 Instituto Murciano de Investigación Biosanitaria (IMIB-Arrixaca), Murcia, Spain; 301 Universidad Católica San Antonio de Murcia (UCAM), Murcia, Spain; 302 Servicio de Medicina Interna-Unidad de Enfermedades Infecciosas, Hospital Universitario de Salamanca-IBSAL, Salamanca, Spain; 303 Department of Internal Medicine, Hospital Universitario de Fuenlabrada, Madrid, Spain; 304 Laboratorio de Vigilancia Molecular Aplicada, Escola Tecnica de Saúde, Pará, Brazil; 305 Genetics Postgraduate Program, Federal University of Pernambuco, Recife, Brazil; 306 Servicio de Alergia, Hospital Universitario Infanta Leonor, Madrid, Spain; 307 Servicio de Medicina Intensiva, Hospital Universitario del Tajo, Toledo, Spain; 308 Hospital Universitario Mutua Terrassa, Barcelona, Spain; 309 Servicio de Farmacología, Hospital Universitario La Paz-IDIPAZ, Madrid, Spain; 310 Alcaldía de Barranquilla, Secretaría de Salud, Barranquilla, Colombia; 311 Xenética Cardiovascular, Instituto de Investigación Sanitaria de Santiago (IDIS), Santiago de Compostela, Spain; 312 Centre for Biomedical Network Research on Cardiovascular Diseases (CIBERCV), Instituto de Salud Carlos III, Madrid, Spain; 313 Unidad de Infección Viral e Inmunidad, Centro Nacional de Microbiología (CNM), Instituto de Salud Carlos III (ISCIII), Madrid, Spain; 314 Cardiovascular Genetics Center, Institut d’Investigació Biomèdica Girona (IDIBGI), Girona, Spain; 315 Medical Science Department, School of Medicine, University of Girona, Girona, Spain; 316 Cardiology Service, Hospital Josep Trueta, Girona, Spain; 317 Institute of Biomedicine of Seville (IBiS), Consejo Superior de Investigaciones Científicas (CSIC), University of Seville, Virgen del Rocio University Hospital, Seville, Spain; 318 Division of Infectious Diseases, Instituto de Investigación Sanitaria–Fundación Jiménez Díaz University Hospital, Universidad Autónoma de Madrid (IIS-FJD, UAM), Madrid, Spain; 319 Intensive Care Unit, Hospital Universitario Insular de Gran Canaria, Las Palmas de Gran Canaria, Spain; 320 Hospital Universitario Mutua Terrassa, Terrassa, Spain; 321 Departemento de Medicina, Hospital Universitario Virgen del Rocío, Universidad de Sevilla, Seville, Spain; 322 Centre for Biomedical Network Research on Epidemiology and Public Health (CIBERESP), Instituto de Salud Carlos III, Madrid, Spain; 323 Instituto de Biomedicina de Sevilla, Seville, Spain; 324 Facultad de Ciencias, Universidad de los Andes, Bogotá, Colombia; 325 Department of Nutrition, University of Fortaleza (UNIFOR), Fortaleza, Brazil; 326 Departamento de Química, Faculdade de Filosofia, Ciências e Letras de Ribeirão Preto, Universidade de São Paulo, São Paulo, Brazil; 327 Andalusian Public Health System Biobank, Granada, Spain; 328 Programa de Pós-Graduação em Ciências Farmacêuticas, Universidade Federal do Rio Grande do Norte, Natal, Brazil; 329 Neuromuscular Unit, Neurology Department, Hospital Universitari de Bellvitge, L’Hospitalet de Llobregat, Barcelona, Spain; 330 Bellvitge Biomedical Research Institute (IDIBELL), Neurometabolic Diseases Laboratory, L’Hospitalet de Llobregat, Barcelona, Spain; 331 Osakidetza, Cruces University Hospital, Barakaldo, Spain; 332 Centre for Biomedical Network Research on Diabetes and Metabolic Associated Diseases (CIBERDEM), Instituto de Salud Carlos III, Madrid, Spain; 333 University of Pais Vasco, UPV/EHU, Bizkaia, Spain; 334 Oncology and Genetics Unit, Instituto de Investigacion Sanitaria Galicia Sur, Xerencia de Xestion Integrada de Vigo-Servizo Galego de Saúde, Vigo, Spain; 335 Hospital Universitario La Paz, Hospital Carlos III, Madrid, Spain; 336 Hospital de San José, Sociedad de Cirugía de Bogota, Bogotá, Colombia; 337 Hospital Universitario Río Hortega, Valladolid, Spain; 338 Servicio de Medicina Intensiva, Complejo Hospitalario Universitario de A Coruña (CHUAC), Sistema Galego de Saúde (SERGAS), A Coruña, Spain; 339 Preventive Medicine Department, Valencia University, Valencia, Spain; 340 Centre for Biomedical Network Research on Physiopatology of Obesity and Nutrition (CIBEROBN), Instituto de Salud Carlos III, Madrid, Spain; 341 Department of Microgravity and Translational Regenerative Medicine, Otto von Guericke University, Magdeburg, Germany; 342 Maternidade Escola Janário Cicco, Natal, Brazil; 343 Centro Nacional de Genotipado (CEGEN), Universidade de Santiago de Compostela, Santiago de Compostela, Spain; 344 Institute of Psychiatry and Mental Health, Hospital General Universitario Gregorio Marañón (IiSGM), Madrid, Spain; 345 Programa de Pós Graduação em Ciências da Saúde, Faculdade de Medicina, Universidade de Brasília, Brasilia, Brazil; 346 Fundació Docència I Recerca Mutua Terrassa, Terrassa, Spain; 347 Unidad de Genética, Hospital Universitario Mostoles, Madrid, Spain; 348 Internal Medicine Department, Instituto de Investigación Sanitaria–Fundación Jiménez Díaz University Hospital, Universidad Autónoma de Madrid (IIS-FJD, UAM), Madrid, Spain; 349 Universidade Federal do Rio Grande do Norte, Pós-graduação em Biotecnologia, Rede de Biotecnologia do Nordeste (Renorbio), Natal, Brazil; 350 Servicio de Medicina Interna, Hospital Universitario Severo Ochoa, Madrid, Spain; 351 Department of Preventive Medicine and Public Health, School of Medicine, Universidad Autónoma de Madrid, Madrid, Spain; 352 IdiPaz (Instituto de Investigación Sanitaria Hospital Universitario La Paz), Madrid, Spain; 353 Instituto Aragonés de Ciencias de la Salud (IACS), Zaragoza, Spain; 354 Instituto Investigación Sanitaria Aragón (IIS-Aragon), Zaragoza, Spain; 355 Programa de Pós Graduação em Nutrição, Universidade Federal do Rio Grande do Norte, Natal, Brazil; 356 Preventive Medicine Department, Instituto de Investigacion Sanitaria Galicia Sur, Xerencia de Xestion Integrada de Vigo-Servizo Galego de Saúde, Vigo, Spain; 357 Servicio de Medicina Interna, Hospital Universitario Virgen del Rocío, Seville, Spain; 358 Departamento de Infectologia, Universidade Federal do Rio Grande do Norte, Natal, Brazil; 359 Hospital de Doenças Infecciosas Giselda Trigueiro, Natal, Brazil; 360 Unidad Diagnóstico Molecular, Fundación Rioja Salud, La Rioja, Spain; 361 Hospital Universitario Quironsalud Madrid, Madrid, Spain; 362 Servicio de Cardiología, Hospital Universitario de Salamanca-IBSAL, Salamanca, Spain; 363 Servicio de Medicina Interna, Hospital Universitario Puerta de Hierro, Majadahonda, Spain; 364 Biocruces Bizkaia Health Research Institute, Galdakao University Hospital, Osakidetza, Bizkaia, Spain; 365 Instituto Regional de Investigación en Salud-Universidad Nacional de Caaguazú, Caaguazú, Paraguay; 366 Núcleo de Pesquisas em Oncologia, Universidade Federal do Pará, Belém, Brazil; 367 Departamento de Ensino e Pesquisa, Hospital Ophir Loyola, Belém, Brazil; 368 Fundación Asilo San Jose, Santander, Spain; 369 Unidad de Enfermedades Infecciosas, Servicio de Medicina Interna, Hospital Universitario Puerta de Hierro, Instituto de Investigación Sanitaria Puerta de Hierro—Segovia de Arana, Madrid, Spain; 370 Universidad Nacional de Asunción, Facultad de Politécnica, Paraguay; 371 Urgencias Hospitalarias, Complejo Hospitalario Universitario de A Coruña (CHUAC), Sistema Galego de Saúde (SERGAS), A Coruña, Spain; 372 Grupo de Investigación en Interacciones Gen-Ambiente y Salud (GIIGAS), Instituto de Biomedicina (IBIOMED), Universidad de León, León, Spain; 373 Pediatrics Department, Hospital Universitario Niño Jesús, Madrid, Spain; 374 Unitat de Malalties Infeccioses i Importades, Servei de Pediatría, Infectious and Imported Diseases, Pediatric Unit, Hospital Universitari Sant Joan de Deú, Barcelona, Spain; 375 Microbiology Department, Instituto de Investigación Sanitaria–Fundación Jiménez Díaz University Hospita, Universidad Autónoma de Madrid (IIS-FJD, UAM), Madrid, Spain; 376 Hospital de Niños Ricardo Gutierrez, Buenos Aires, Argentina; 377 University of Salamanca, Biomedical Research Institute of Salamanca (IBSAL), Salamanca, Spain; 378 Department of Immunology, IRYCIS, Hospital Universitario Ramón y Cajal, Madrid, Spain; 379 Servicio de Medicina Intensiva, Hospital Infanta Elena, Madrid, Spain; 380 Servicio de Genética, Hospital Universitario de Getafe, Madrid, Spain; 381 Pneumology Department, Hospital General Universitario Gregorio Marañón (iiSGM), Madrid, Spain; 382 Ministerio de Salud Ciudad de Buenos Aires, Buenos Aires, Argentina; 383 Unidad de Apoyo a la Investigación, Hospital Clinico Universitario de Valladolid, Valladolid, Spain; 384 Departamento de Cirugía, Universidad de Valladolid, Valladolid, Spain; 385 Secretaria Municipal de Saude de Apodi, Natal, Brazil; 386 Sección Genética Médica, Servicio de Pediatría, Hospital Clínico Universitario Virgen de la Arrixaca, Servicio Murciano de Salud, Murcia, Spain; 387 Departamento Cirugía, Pediatría, Obstetricia y Ginecología, Facultad de Medicina, Universidad de Murcia (UMU), Murcia, Spain; 388 Grupo Clínico Vinculado, Centre for Biomedical Network Research on Rare Diseases (CIBERER), Instituto de Salud Carlos III, Madrid, Spain; 389 Servicio de Análisis Clínicos e Inmunología, Hospital Universitario Virgen de las Nieves, Granada, Spain; 390 Hospital Universitario Centro Dermatológico Federico Lleras Acosta, Bogotá, Colombia; 391 Intermediate Respiratory Care Unit, Department of Pneumology, Instituto de Investigación Sanitaria–Fundación Jiménez Díaz University Hospital, Universidad Autónoma de Madrid (IIS-FJD, UAM), Madrid, Spain; 392 Clinica Comfamiliar Risaralda, Pereira, Colombia; 393 Centro Universitario de Tonalá, Universidad de Guadalajara, Guadalajara, Mexico; 394 Unidad de Cuidados Intensivos, Hospital Clínico Universitario de Santiago (CHUS), Sistema Galego de Saúde (SERGAS), Santiago de Compostela, Spain; 395 Plataforma de Farmacogenética, IIS La Fe, Valencia, Spain; 396 Departamento de Farmacología, Universidad de Valencia, Valencia, Spain; 397 Data Analysis Department, Instituto de Investigación Sanitaria–Fundación Jiménez Díaz University Hospital, Universidad Autónoma de Madrid (IIS-FJD, UAM), Madrid, Spain; 398 Infectious Diseases Service, Hospital del Mar, Barcelona, Spain; 399 Institut Hospital del Mar d’Investigacions Mèdiques (IMIM), Barcelona, Spain; 400 CEXS-Universitat Pompeu Fabra, Spanish Network for Research in Infectious Diseases (REIPI), Barcelona, Spain; 401 Biocruces Bizkaia Health Research Institute, Basurto University Hospital, Osakidetza, Bizkaia, Spain; 402 Infectious Diseases, Microbiota and Metabolism Unit, Center for Biomedical Research of La Rioja (CIBIR), Logroño, Spain; 403 Sabin Medicina Diagnóstica, São Paulo, Brazil; 404 Opthalmology Department, Instituto de Investigación Sanitaria–Fundación Jiménez Díaz University Hospital, Universidad Autónoma de Madrid (IIS-FJD, UAM), Madrid, Spain; 405 Pediatric Critical Care Unit, Hospital Sant Joan de Deu, Barcelona, Spain; 406 Paediatric Intensive Care Unit, Agrupación Hospitalaria Clínic-Sant Joan de Déu, Esplugues de Llobregat, Barcelona, Spain; 407 Department of Immunology, Hospital Universitario 12 de Octubre, Madrid, Spain; 408 Transplant Immunology and Immunodeficiencies Group, Instituto de Investigación Sanitaria Hospital 12 de Octubre (imas12), Madrid, Spain; 409 SIGEN Alianza Universidad de los Andes, Fundación Santa Fe de Bogotá, Bogotá, Colombia; 410 Medicina Intensiva, Hospital General de Segovia, Segovia, Spain; 411 Clinical Trials Unit, Instituto de Investigación Sanitaria–Fundación Jiménez Díaz University Hospital, Universidad Autónoma de Madrid (IIS-FJD, UAM), Madrid, Spain; 412 IMDEA-Food Institute, CEI UAM+CSIC, Madrid, Spain; 413 Servicio de Enfermedades Infecciosas, Hospital Universitario Virgen de las Nieves, Granada, Spain; 414 Instituto de Investigación Biosanitaria de Granada (ibs.GRANADA), Granada, Spain; 415 Departamento de Medicina, Universidad de Granada, Granada, Spain; 416 Servicio de Inmunología, Hospital Universitario La Paz-IDIPAZ, Madrid, Spain; 417 Lymphocyte Pathophysiology in Immunodeficiencies Group, La Paz Institute for Health Research (IdiPAZ), Madrid, Spain; 418 Intensive Care Unit, Hospital Universitario de Canarias, La Laguna, Spain; 419 Dirección General de Salud Pública, Consejería de Sanidad, Junta de Castilla y León, Valladolid, Spain; 420 Departamento de Analises Clinicas e Toxicologicas, Universidade Federal do Rio Grande do Norte, Natal, Brazil; 421 Epidemiology, Fundación Jiménez Díaz, Madrid, Spain; 422 Department of Medicine, Universidad Autónoma de Madrid, Madrid, Spain; 423 Instituto de Biomedicina (IBIOMED), Universidad de León, León, Spain; 424 Intensive Care Unit, Hospital Universitario N. S. de Candelaria, Santa Cruz de Tenerife, Spain; 425 Preventive Medicine Department, Instituto de Investigación Sanitaria–Fundación Jiménez Díaz University Hospital, Universidad Autónoma de Madrid (IIS-FJD, UAM), Madrid, Spain; 426 Departamento de Medicina, Universidad de Valladolid, Valladolid, Spain; 427 Servicio de Medicina Intensiva, Hospital Universitario Infanta Leonor, Madrid, Spain; 428 Servicio de Medicina Interna, Sanatorio Franchin, Buenos Aires, Argentina; 429 Unidad de Genética y Genómica Islas Baleares, Hospital Universitario Son Espases, Islas Baleares, Spain; 430 Unidad de Diagnóstico Molecular y Genética Clínica, Hospital Universitario Son Espases, Islas Baleares, Spain; 431 Genomics of Complex Diseases Unit, Research Institute of Hospital de la Santa Creu i Sant Pau, IIB Sant Pau, Barcelona, Spain; 432 Faculdade de Medicina, Universidade de Brasília, Brasilia, Brazil; 433 Programa de Pós-Graduação em Ciências Médicas, Universidade de Brasília, Brasilia, Brazil; 434 Programa de Pós-Graduação em Ciências da Saúde, Universidade de Brasília, Brasilia, Brazil; 435 Hospital das Forças Armadas, Brasília, Brazil; 436 Exército Brasileiro, Cruzeiro, Brazil; 437 Hospital El Bierzo, Gerencia de Asistencia Sanitaria del Bierzo (GASBI), Gerencia Regional de Salud (SACYL), Ponferrada, Spain; 438 Grupo INVESTEN, Instituto de Salud Carlos III, Madrid, Spain; 439 Unidad de Cuidados Intensivos, Complejo Universitario de A Coruña (CHUAC), Sistema Galego de Saúde (SERGAS), A Coruña, Spain; 440 Unidad Cuidados Intensivos, Hospital El Bierzo, León, Spain; 441 Familial Cancer Clinical Unit, Spanish National Cancer Research Centre, Madrid, Spain; 442 Instituto de Investigación Sanitaria San Carlos (IdISSC), Hospital Clínico San Carlos (HCSC), Madrid, Spain; 443 Departamento de Enfermería, Universidad de Sevilla, Seville, Spain; 444 Hospital General Universitario Gregorio Marañón (IiSGM), Madrid, Spain; 445 ERN-ITHACA-European Reference Network on Rare Congenital Malformations and Rare Intellectual Disability, Brussels, Belgium; 446 Unidad de Genética y Genómica Islas Baleares, Unidad de Diagnóstico Molecular y Genética Clínica, Hospital Universitario Son Espases, Islas Baleares, Spain; 447 Instituto de Investigación Sanitaria Islas Baleares (IdISBa), Islas Baleares, Spain; 448 Programa de Pós-Graduação em Biologia Animal, Universidade de Brasília, Brasília, Brazil; 449 Programa de Pós-Graduação Profissional em Ensino de Biologia, Universidade de Brasília, Brasília, Brazil; 450 Anatomía Patológica, Instituto de Investigación Sanitaria San Carlos (IdISSC), Hospital Clínico San Carlos (HCSC), Madrid, Spain; 451 Tecnológico de Monterrey, Monterrey, Mexico; 452 Centro de Investigación en Anomalías Congénitas y Enfermedades Raras (CIACER), Universidad Icesi, Cali, Colombia; 453 Departamento de Genetica, Fundación Valle del Lili, Cali, Colombia; 454 Department of Immunology, Ophthalmology and ENT, Universidad Complutense de Madrid, Madrid, Spain; 455 Department of Neumology, Instituto de Investigación Sanitaria–Fundación Jiménez Díaz University Hospital, Universidad Autónoma de Madrid (IIS-FJD, UAM), Madrid, Spain; 456 Hospital Nuestra Señora de Sonsoles, Ávila, Spain; 457 Inditex, A Coruña, Spain; 458 Intensive Care Department, Instituto de Investigación Sanitaria–Fundación Jiménez Díaz University Hospital, Universidad Autónoma de Madrid (IIS-FJD, UAM), Madrid, Spain; 459 Servicio de Microbiología Clínica, Hospital Universitario Príncipe de Asturias, Madrid, Spain; 460 Departamento de Biomedicina y Biotecnología, Facultad de Medicina y Ciencias de la Salud, Universidad de Alcalá de Henares, Madrid, Spain; 461 GENYCA, Madrid, Spain; 462 Marinha do Brasil, Brasil, Brazil; 463 Universidade de Brasília, Brasilia, Brazil; 464 Neuromuscular Diseases Unit, Department of Neurology, Hospital de la Santa Creu i Sant Pau, Universitat Autònoma de Barcelona, Barcelona, Spain; 465 Unidad de Investigación Médica en Enfermedades Infecciosas y Parasitarias, Instituto Mexicano del Seguro Social (IMSS), Centro Médico Nacional Siglo XXI, Mexico City, Mexico; 466 Catalan Institution of Research and Advanced Studies (ICREA), Barcelona, Spain; 467 Drug Research Centre, Institut d’Investigació Biomèdica Sant Pau, IIB-Sant Pau, Barcelona, Spain; 468 Departamento de Genetica, Clinica imbanaco, Cali, Colombia; 469 Department of Immunology, Hospital Universitario de Gran Canaria Dr Negrín, Las Palmas de Gran Canaria, Spain; 470 Pediatrics Department, University Hospital Germans Trias i Pujol, Badalona, Spain; 471 Department of Pathology, Biobank, Instituto de Investigación Sanitaria–Fundación Jiménez Díaz University Hospital, Universidad Autónoma de Madrid (IIS-FJD, UAM), Madrid, Spain; 472 Faculdade de Ciências da Saúde, Universidade de Brasília, Brasilia, Brazil; 473 Servicio de Medicina Interna, Hospital Universitario Virgen de las Nieves, Granada, Spain; 474 Grupo de Ciencias Básicas en Salud (CBS), Fundación Universitaria de Ciencias de la Salud, Bogotá, Colombia; 475 Sociedad de Cirugía de Bogotá, Hospital de San José, Bogotá, Colombia; 476 Departamento Bioquímica, Biología Molecular e Inmunología III, Universidad de Granada, Granada, Spain; 477 Allergy Unit, Hospital Infanta Elena, Madrid, Spain; 478 Faculty of Medicine, Universidad Francisco de Vitoria, Madrid, Spain; 479 Hospital Universitario Infanta Leonor, Madrid, Spain; 480 Complutense University of Madrid, Madrid, Spain; 481 Gregorio Marañón Health Research Institute (IiSGM), Madrid, Spain; 482 Reumathology Service, Instituto de Investigación Sanitaria–Fundación Jiménez Díaz University Hospital, Universidad Autónoma de Madrid (IIS-FJD, UAM), Madrid, Spain; 483 Biobank, Puerta de Hierro-Segovia de Arana Health Research Institute, Madrid, Spain; 484 Universidad Rey Juan Carlos, Madrid, Spain; 485 The John Walton Muscular Dystrophy Research Centre, Newcastle University and Newcastle Hospitals NHS Foundation Trust, Newcastle upon Tyne, UK; 486 Neuromuscular Unit, Neuropediatrics Department, Institut de Recerca Sant Joan de Déu, Hospital Sant Joan de Déu, Barcelona, Spain; 487 Casa de Saúde São Lucas, Natal, Brazil; 488 Hospital Rio Grande, Natal, Brazil; 489 Intensive Care Unit, Hospital Universitario de Gran Canaria Dr Negrín, Las Palmas de Gran Canaria, Spain; 490 Universidad Fernando Pessoa Canarias, Las Palmas de Gran Canaria, Spain; 491 Servicio de Anestesiologia y Reanimación, Hospital Clinico Universitario de Valladolid, Valladolid, Spain; 492 Servicio de Hematologia y Hemoterapia, Hospital Clinico Universitario de Valladolid, Valladolid, Spain; 493 Hospital Universitario Lauro Wanderley, João Pessoa, Brazil; 494 Servicio de Medicina Interna, Hospital Universitario Infanta Leonor, Madrid, Spain; 495 University Hospital of Burgos, Burgos, Spain; 496 Universidad de Sevilla, Seville, Spain; 497 Fundación Santa Fe de Bogota, Instituto de Servicios Medicos de Emergencia y Trauma, Bogotá, Colombia; 498 Universidad de los Andes, Bogotá, Colombia; 499 Quironprevención, A Coruña, Spain; 500 Junta de Castilla y León, Consejería de Sanidad, Valladolid, Spain; 501 Gerencia Atención Primaria de Burgos, Burgos, Spain; 502 Immunogenetics–Histocompatibility group, Servicio de Inmunología, Instituto de Investigación Sanitaria Puerta de Hierro, Segovia de Arana, Madrid, Spain; 503 Department of Infectious Diseases, Hospital del Mar, Barcelona, Spain; 504 IMIM—Hospital del Mar Medical Research Institute, Institut Hospital del Mar d’Investigacions Mediques, Barcelona, Spain; 505 Department of Medicine, Universitat Autònoma de Barcelona, Barcelona, Spain; 506 Consejería de Sanidad, Comunidad de Madrid, Madrid, Spain; 507 Centro para el Desarrollo de la Investigación Científica, Asunción, Paraguay; 508 School of Informatics, University of Edinburgh, Edinburgh, UK; 509 Section of Biomolecular Medicine, Division of Systems Medicine, Department of Metabolism, Digestion and Reproduction, Sir Alexander Fleming Building, Imperial College London, London, UK; 510 Section of Genomic and Environmental Medicine, Respiratory Division, National Heart and Lung Institute, London, UK; 511 Section of Molecular Virology, Imperial College London, London, UK; 512 Antimicrobial Resistance and Hospital Acquired Infection Department, Public Health England, London, UK; 513 Department of Epidemiology and Biostatistics, School of Public Health, Faculty of Medicine, Imperial College London, London, UK; 514 Department of Infectious Disease, Imperial College London, London, UK; 515 MRC-University of Glasgow Centre for Virus Research, Glasgow, UK; 516 The Florey Institute for Host-Pathogen Interactions, Department of Infection, Immunity and Cardiovascular Disease, University of Sheffield, Sheffield, UK; 517 Centre for Medical Informatics, The Usher Institute, University of Edinburgh, Edinburgh, UK; 518 National Phenome Centre, Department of Metabolism, Digestion and Reproduction, Imperial College London, London, UK; 519 Section of Bioanalytical Chemistry, Department of Metabolism, Digestion and Reproduction, Imperial College London, London, UK; 520 European Genomic Institute for Diabetes, CNRS UMR 8199, INSERM UMR 1283, Institut Pasteur de Lille, Lille University Hospital, University of Lille, Lille, France; 521 McGill University and Genome Quebec Innovation Centre, Montréal, Quebec Canada; 522 National Infection Service, Public Health England, London, UK; 523 Liverpool School of Tropical Medicine, Liverpool, UK; 524 Institute of Microbiology and Infection, University of Birmingham, Birmingham, UK; 525 Department of Molecular and Clinical Cancer Medicine, University of Liverpool, Liverpool, UK; 526 Institute for Global Health, University College London, London, UK; 527 Department of Infectious Diseases, Queen Elizabeth University Hospital, Glasgow, UK; 528 University of Liverpool, Liverpool, UK; 529 Virology Reference Department, National Infection Service, Public Health England, London, UK; 530 Department of Pharmacology, University of Liverpool, Liverpool, UK; 531 Nuffield Department of Medicine, Peter Medawar Building for Pathogen Research, University of Oxford, Oxford, UK; 532 Translational Gastroenterology Unit, Nuffield Department of Medicine, University of Oxford, Oxford, UK; 533 Nottingham University Hospitals NHS Trust, Nottingham, UK; 534 Nuffield Department of Medicine, John Radcliffe Hospital, Oxford, UK; 535 Department of Microbiology/Infectious Diseases, Oxford University Hospitals NHS Foundation Trust, John Radcliffe Hospital, Oxford, UK; 536 ISARIC Global Support Centre, Centre for Tropical Medicine and Global Health, Nuffield Department of Medicine, University of Oxford, Oxford, UK; 537 Institute of Infection, Veterinary and Ecological Sciences, University of Liverpool, Liverpool, UK; 538 Division of Infection and Immunity, University College London, London, UK; 539 Molecular and Clinical Cancer Medicine, Institute of Systems, Molecular and Integrative Biology, University of Liverpool, Liverpool, UK; 540 Clatterbridge Cancer Centre NHS Foundation Trust, Liverpool, UK; 541 NIHR Health Protection Research Unit in Emerging and Zoonotic Infections, Liverpool, UK; 542 Centre for Clinical Infection and Diagnostics Research, Department of Infectious Diseases, School of Immunology and Microbial Sciences, King’s College London, London, UK; 543 Department of Infectious Diseases, Guy’s and St Thomas’ NHS Foundation Trust, London, UK; 544 Institute of Evolutionary Biology, University of Edinburgh, Edinburgh, UK; 545 Department of Pediatrics and Virology, St Mary’s Medical School Building, Imperial College London, London, UK; 546 NHS Greater Glasgow & Clyde, Glasgow, UK; 547 Walton Centre NHS Foundation Trust, Liverpool, UK; 548 MRC Centre for Molecular Bacteriology and Infection, Imperial College London, London, UK; 549 Department of Child Life and Health, University of Edinburgh, Edinburgh, UK; 550 National Phenome Centre, Division of Systems Medicine, Department of Metabolism, Digestion and Reproduction, Imperial College London, London, UK; 551 Blood Borne Virus Unit, Virus Reference Department, National Infection Service, Public Health England, London, UK; 552 Transfusion Microbiology, National Health Service Blood and Transplant, London, UK; 553 Department of Medicine, Imperial College London, London, UK; 554 Department of Infection, Immunity and Cardiovascular Disease, University of Sheffield, Sheffield, UK; 555 Tropical & Infectious Disease Unit, Royal Liverpool University Hospital, Liverpool, UK; 556 Liverpool Clinical Trials Centre, University of Liverpool, Liverpool, UK; 557 Public Health Scotland, Edinburgh, UK; 558 Centre for Health Informatics, Division of Informatics, Imaging and Data Science, School of Health Sciences, Faculty of Biology, Medicine and Health, University of Manchester, Manchester Academic Health Science Centre, Manchester, UK; 559 EPCC, University of Edinburgh, Edinburgh, UK; 560 ISARIC, Global Support Centre, COVID-19 Clinical Research Resources, Epidemic diseases Research Group, Oxford (ERGO), University of Oxford, Oxford, UK; 561 Institute of Infection, Veterinary and Ecological Sciences, Faculty of Health and Life Sciences, University of Liverpool, Liverpool, UK; 562 23andMe, Sunnyvale, CA USA; 563 Human genetics R&D, GSK Medicines Research Centre, Target Sciences R&D, Stevenage, UK

**Keywords:** Genome-wide association studies, Genetics research, SARS-CoV-2, Viral infection

## Abstract

Critical illness in COVID-19 is an extreme and clinically homogeneous disease phenotype that we have previously shown^1^ to be highly efficient for discovery of genetic associations^2^. Despite the advanced stage of illness at presentation, we have shown that host genetics in patients who are critically ill with COVID-19 can identify immunomodulatory therapies with strong beneficial effects in this group^3^. Here we analyse 24,202 cases of COVID-19 with critical illness comprising a combination of microarray genotype and whole-genome sequencing data from cases of critical illness in the international GenOMICC (11,440 cases) study, combined with other studies recruiting hospitalized patients with a strong focus on severe and critical disease: ISARIC4C (676 cases) and the SCOURGE consortium (5,934 cases). To put these results in the context of existing work, we conduct a meta-analysis of the new GenOMICC genome-wide association study (GWAS) results with previously published data. We find 49 genome-wide significant associations, of which 16 have not been reported previously. To investigate the therapeutic implications of these findings, we infer the structural consequences of protein-coding variants, and combine our GWAS results with gene expression data using a monocyte transcriptome-wide association study (TWAS) model, as well as gene and protein expression using Mendelian randomization. We identify potentially druggable targets in multiple systems, including inflammatory signalling (*JAK1*), monocyte–macrophage activation and endothelial permeability (*PDE4A*), immunometabolism (*SLC2A5* and *AK5*), and host factors required for viral entry and replication (*TMPRSS2* and *RAB2A*).

## Abstract (web summary)

An analysis of 24,202 critical cases of COVID-19 identifies potentially druggable targets in inflammatory signalling (*JAK1*), monocyte–macrophage activation and endothelial permeability (*PDE4A*), immunometabolism (*SLC2A5* and *AK5*), and host factors required for viral entry and replication (*TMPRSS2* and *RAB2A*).

## Main

**Extended Data Fig. 1:**
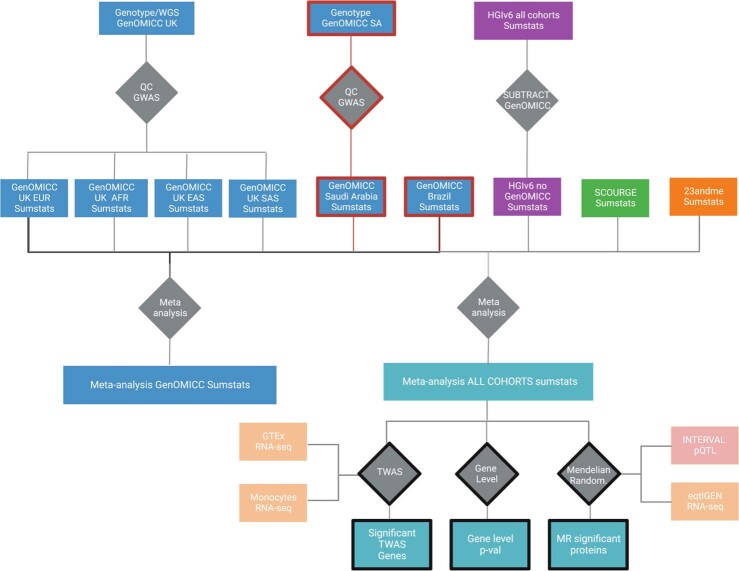
Pipeline of meta-analysis and post-GWAS analyses. Red border indicates that the data is only available for the hospitalized phenotype, while a black border indicates that the analysis was performed for the critical illness phenotype.

**Extended Data Fig. 2:**
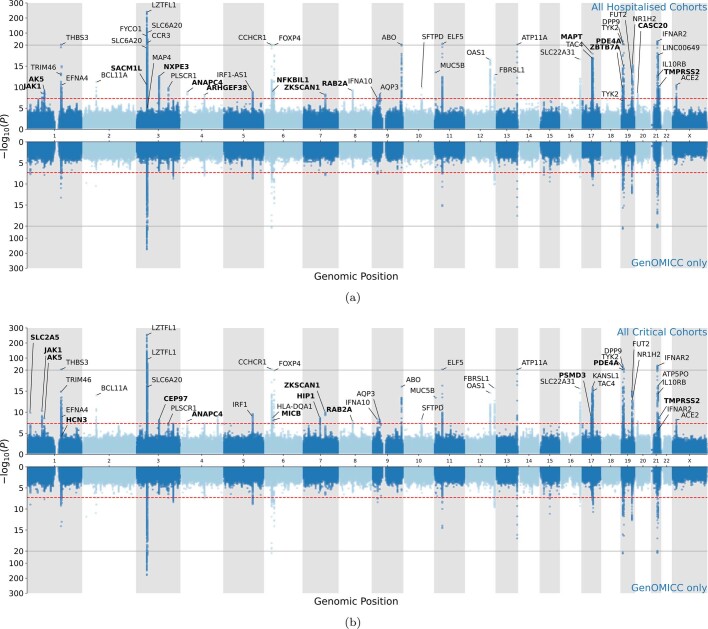
Miami plots. Meta-analysis results are shown for a) critical and b) hospitalized phenotypes. In each plot results obtained using all cohorts are shown at the top and using GenOMICC data only at the bottom. Independent lead variants in the analyses of all cohorts are annotated with associated genes. Genome-wide significant associations that have not been previously reported are indicated in bold.

The design of the GenOMICC study and the rationale for focusing on critical illness has been previously described^1,2^. In brief, patients with confirmed COVID-19 requiring continuous cardiorespiratory monitoring or organ support (a generalizable definition for critical illness) were recruited in 2020–2022. We first performed ancestry-specific GWAS analyses according to the methods that we described previously^1,2^. Using the results of these GWAS analyses, previously reported results obtained using GenOMICC participants with whole-genome sequencing data^2^ and data from GenOMICC Brazil, we performed trans-ancestry and -platform meta-analyses within the GenOMICC study for a critically ill COVID-19 phenotype and a hospitalized COVID-19 phenotype (Extended Data Fig. 1). The results of these GenOMICC-only meta-analyses are presented for both critically ill and hospitalized phenotypes (Table 1 and Extended Data Fig. 2). To put these results into the context of existing knowledge, we performed comprehensive meta-analyses, drawing on further GWAS results, including data shared by the SCOURGE consortium and published data from the COVID-19 Human Genetics Initiative (HGIv6, 2021)^4^. The characteristics of the contributing studies are summarized in Supplementary Tables 13 and 14 for the critically ill and hospitalized phenotypes, with further details on each study provided in the Supplementary Information. We used a mathematical subtraction approach, as done in our previous work^2^, to remove signals of previous GenOMICC releases from HGIv6, yielding an independent dataset.

**Table 1:** Genome-wide significant associations with critical COVID-19, listing independent lead variants

Chr:pos(b38)	rsid	EA	OR	OR_CI_	*P*	*P*_cond_	Nearest gene	Citation
1:9067157	rs2478868	A	0.92	0.9–0.95	1.5 × 10^−10^		*SLC2A5*	GenOMICC^new^
1:64948270	rs12046291	A	1.1	1.07–1.13	5.1 × 10^−11^		*JAK1*	GenOMICC^new^
1:77501822	rs71658797	A	1.1	1.09–1.18	2.8 × 10^−9^		*AK5*	GenOMICC^new^
1:155066988	rs114301457	T	2.4	1.81–3.18	1.5 × 10^−9^		*EFNA4*	GenOMICC^2^
1:155175305	rs7528026	A	1.3	1.25–1.44	1.5 × 10^−15^		*TRIM46*	GenOMICC^2^
1:155197995	rs41264915	A	1.2	1.17–1.26	7.6 × 10^−24^		*THBS3*	HGI^18^
1:155278322	rs11264349	A	0.94	0.92–0.97	7.3 × 10^−5^	3.9 × 10^−13^	*HCN3*	GenOMICC^new^
2:60480453	rs1123573	A	1.1	1.09–1.15	1 × 10^−14^		*BCL11A*	GenOMICC^2^
3:45796521	rs2271616	T	1.2	1.14–1.23	1.1 × 10^−16^		*SLC6A20*	HGI^18^
3:45818159	rs17713054*	A	2	1.96–2.13	7.7 × 10^−254^		*LZTFL1*	SCGG^19^
3:45873093	rs35482426	CTT	0.53	0.5–0.57	6.1 × 10^−91^		*LZTFL1*	SCGG^19^
3:101790631	rs11706494	A	1.1	1.05–1.11	9.4 × 10^−9^		*NXPE3*	GenOMICC^new^
3:146522652	rs343314	T	1.2	1.09–1.21	4.6 × 10^−8^		*PLSCR1*	GenOMICC^2^
4:25446871	rs7664615	A	1.1	1.07–1.14	1.5 × 10^−8^		*ANAPC4*	GenOMICC^new^
4:105673359	rs72670002	A	1.1	1.09–1.21	4.4 × 10^−8^		*ARHGEF38*	GenOMICC^new^
4:167824478	rs1073165	A	1.1	1.05–1.11	1.1 × 10^−9^		−	GenOMICC^new^
5:132422622	rs2269821	A	1.1	1.08–1.16	3 × 10^−10^		*IRF1-AS1*	GenOMICC^2^
6:31153455	rs111837807	T	0.8	0.77–0.84	8.6 × 10^−26^		*CCHCR1*	GenOMICC^1^
6:31571991	rs2071590	A	1.1	1.06–1.11	3.1 × 10^−10^		*LTA*	GenOMICC^new^
6:32702687	rs2858305	T	0.93	0.9–0.95	2.1 × 10^−9^		*HLA-DQA1*	GenOMICC^2^
6:41522644	rs41435745	C	1.4	1.31–1.51	1.5 × 10^−20^		*FOXP4*	HGI^18^
7:75623396	rs1179620	T	0.92	0.9–0.95	2.3 × 10^−9^		*HIP1*	GenOMICC^new^
7:100032719	rs2897075	T	1.1	1.06–1.11	8.9 × 10^−11^		*ZKSCAN1*	GenOMICC^new^
8:60532539	rs13276831	T	1.1	1.05–1.1	1.7 × 10^−8^		*RAB2A*	GenOMICC^new^
9:21206606	rs28368148	C	0.59	0.49–0.7	5.3 × 10^−9^		*IFNA10*	GenOMICC^2^
9:33425186	rs60840586	G	1.1	1.07–1.14	9.7 × 10^−9^		*AQP3*	SCOURGE^20^
9:133271182	rs879055593	T	1.1	1.1–1.16	1 × 10^−16^		*ABO*	SCGG^19^
10:79946568	rs721917	A	0.93	0.9–0.95	7.6 × 10^−9^		*SFTPD*	HGI^4^
11:1219991	rs35705950	T	0.86	0.82–0.89	3.8 × 10^−14^		*MUC5B*	HGI^4^
11:34482745	rs61882275	A	0.88	0.86–0.91	1 × 10^−22^		*ELF5*	GenOMICC^2^
12:112919637	rs2660	A	1.1	1.08–1.13	2.8 × 10^−15^		*OAS1*	GenOMICC^1^
12:132481571	rs11614702	A	1.1	1.08–1.13	2.1 × 10^−16^		*FBRSL1*	GenOMICC^2^
13:112881427	rs12585036	T	1.2	1.12–1.18	9.6 × 10^−22^		*ATP11A*	GenOMICC^2^
16:89196249	rs117169628	A	1.2	1.12–1.2	2.6 × 10^−16^		*SLC22A31*	GenOMICC^2^
17:40003082	rs12941811	T	0.93	0.91–0.95	1.1 × 10^−9^		*PSMD3*	GenOMICC^new^
17:46085231	rs8080583	A	0.89	0.86–0.91	1.8 × 10^−16^		*KANSL1*	^21^
17:49863303	rs77534576	T	1.3	1.24–1.43	8.7 × 10^−16^		*TAC4*	^21^
19:4717660	rs12610495	A	0.8	0.77–0.82	9.1 × 10^−51^		*DPP9*	GenOMICC^1^
19:10352442	rs34536443	C	1.5	1.39–1.61	2.2 × 10^−28^		*TYK2*	GenOMICC^1^
19:10414696	rs142770866	A	1.2	1.19–1.3	9.4 × 10^−21^		*PDE4A*	GenOMICC^new^
19:48702915	rs516246	T	0.9	0.88–0.93	1.4 × 10^−15^		*FUT2*	GenOMICC^2^
19:50374423	rs35463555	A	1.1	1.07–1.13	1.9 × 10^−13^		*NR1H2*	HGI^4^
20:6489447	rs2326788	A	0.93	0.9–0.95	1.5 × 10^−8^		*CASC20*	GenOMICC^new^
21:33229937	rs188401375	C	0.74	0.66–0.84	3.1 × 10^−6^	4.7 × 10^−9^	*IFNAR2*	GenOMICC^1^
21:33237639	rs9636867	A	0.83	0.81–0.85	5.2 × 10^−48^		*IFNAR2*	GenOMICC^1^
21:33287378	rs8178521	T	1.1	1.1–1.17	6.2 × 10^−15^		*IL10RB*	GenOMICC^2^
21:33980963	rs76608815	T	1.2	1.14–1.23	7.4 × 10^−17^		*ATP5PO*	GenOMICC^2^
21:41479527	rs915823	A	1.1	1.06–1.13	2.1 × 10^−9^		*TMPRSS2*	GenOMICC^new^
X:15523993	rs35697037	A	1	1.03–1.06	6.8 × 10^−9^		*ACE2*	HGI^4^

Chr:pos(b38), chromosome and position on human genome build 38; rsid, lead variant rsid; EA, effect allele; OR, odds ratio; OR_CI_, odds ratio confidence interval; *P*, *P* value against null hypothesis of odds ratio of one; *P*_cond_, *P* value in conditional analysis in variants with *P* > 5 × 10^−8^; nearest gene, the nearest or most plausible nearby gene; citation, the first report of genome-wide significant association. ‘GenOMICC^new^’ indicates findings presented in this Article. Additional details are provided in Supplementary Table 15 and full results of conditional analysis are provided in Supplementary Table 16. Supplementary Table 17 contains details of lead variants from the analysis of the hospitalized phenotype.

**Extended Data Fig. 3:**
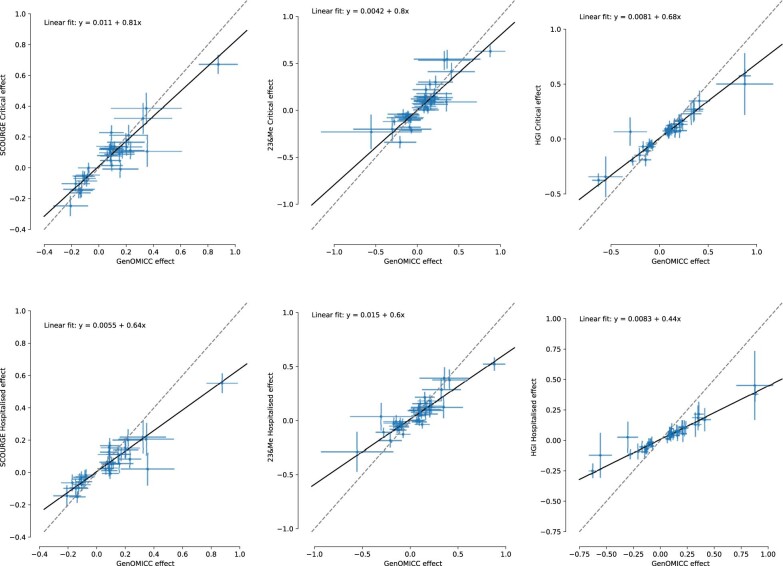
Comparison of effect size estimates. GenOMICC is compared with the critical and hospitalized phenotype definitions in the SCOURGE, 23andMe, and HGI analyses. The black line indicates the best linear fit, given by the equation in each plot, obtained using Orthogonal Distance Regression to account for estimation errors in both sets of effects in the comparison.

**Extended Data Fig. 4:**
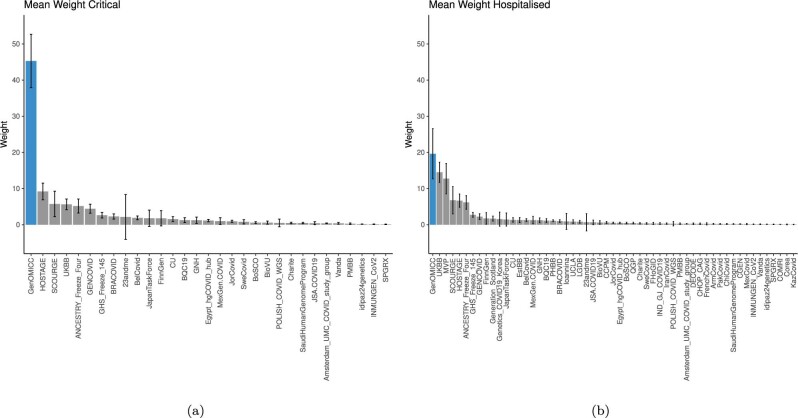
Study weightings for (a) critical and (b) hospitalized COVID-19. Mean +/− standard deviation of weights assigned to each data source in meta-analyses for all significant SNPs.

As no replication cohorts exist for these meta-analyses, we used the heterogeneity across studies to assess the reliability of individual findings (Supplementary Table 15). Owing to the unusually extreme phenotype in the GenOMICC study, some heterogeneity is expected for the strongest associations when compared with studies with more permissive inclusion criteria. Importantly, significant heterogeneity was not detected for any of the findings that we report here (Supplementary Table 15). Comparing effect estimates between studies using a regression approach that takes into account estimation errors (Methods), we detected systematic differences in effect sizes between studies (Extended Data Fig. 3). For example, effects for the HGI critical illness phenotype (which was designed to parallel the GenOMICC inclusion criteria) are smaller than those obtained using prospective recruitment in GenOMICC by a factor of 0.68. As the effect sizes in GenOMICC are consistently larger than other studies, and GenOMICC contributes a disproportionately large signal to meta-analyses of both critical and hospitalized phenotypes (Extended Data Fig. 4), between-study heterogeneity is likely to reflect the careful case ascertainment and extreme phenotype in GenOMICC compared with other studies.

We found 49 common genetic associations with critical COVID-19 meeting our criteria for genome-wide significance in the absence of heterogeneity (Extended Data Fig. 2 and Table 1). Findings from previous reports were consistently replicated (Extended Data Table 2). Conditional analysis revealed two additional lead variants (Table 1) and statistical fine-mapping provided credible sets of putative causal variants for a majority of lead variants (Supplementary Figs. 27–44 and Supplementary Table 5). Gene-level analyses found 196 significantly associated genes at a Bonferroni-corrected threshold (Supplementary Table 10). There were no genome-wide significant differences in the effects between sexes in a sex-stratified meta-analysis using a subset of cohorts (Supplementary Fig. 1).

## Therapeutic implications

Our analysis is limited to common variants that are detectable on genotyping arrays and imputation panels. Although most lead variants are not directly causal, in some cases, they highlight molecular mechanisms that alter clinical outcomes in COVID-19, and may have direct therapeutic relevance. To investigate the disease mechanisms, we first quantified the effect of inferred gene expression on critical illness in three relevant tissue/cell types. Many of the genes that we have found to be implicated in critical COVID-19 (refs. ^1,2^) are highly expressed in the monocyte–macrophage system, which has poor coverage in existing expression quantitative trait loci (eQTL) datasets. For this reason, we constructed a new TWAS model in primary monocytes obtained from 176 individuals (Methods). We found significant associations after Bonferroni correction between critical COVID-19 and predicted gene expression in lung (33), blood (21), monocyte (37) and all-tissue (107) meta-analysis (Supplementary Table 2 and Supplementary Table 11). We extended these findings using generalized summary-level data Mendelian randomization (GSMR) for RNA expression (Fig. 2, Extended Data Table 1, Supplementary Figs. 11–18 and Supplementary Table 4).

**Extended Data Fig. 5:**
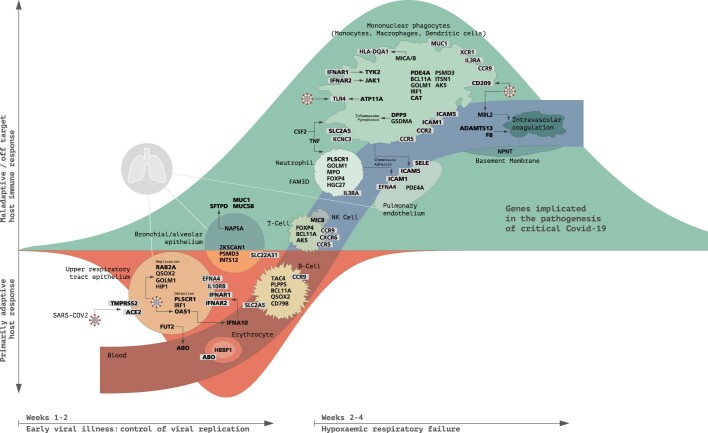
Cartoon showing postulated roles for genes and mediators implicated in the pathogenesis of critical COVID-19 by GenOMICC GWAS, TWAS and Mendelian randomization. Postulated roles for genetic variants are shown in a highly simplified format to illustrate potential roles in pathogenesis, with the shaded background indicating the hypothetical impact of the host immune response over time^17^. Host immune processes are divided into those that are thought to play a role in controlling viral replication early in disease (orange section, showing “adaptive” response), and those implicated in driving hypoxaemic respiratory failure later in disease (green section, showing “maladaptive” response). Bold type gene names indicate a higher level of confidence in both the gene identification and the biological role.

In parallel, we assessed the effect of genetically determined variation in circulating protein levels on the critical illness phenotype using GSMR^5^. We identified 15 unique proteins linked to critical illness, as summarized in Extended Data Table 1 (Supplementary Table 3). Of the significant results, we found causal evidence implicating five new proteins in comparison to our previous GSMR analysis^2^: QSOX2, CREB3L4, myeloperoxidase (MPO), ADAMTS13 and mannose-binding lectin-2 (MBL2) (Supplementary Fig. 10). These include well-studied biomarkers and potential drug targets in sepsis—the innate immune pattern recognition receptor MBL2 and the neutrophil effector enzyme MPO. ADAMTS13 modulates von Willebrand-factor-mediated platelet thrombus formation and may have a role in the hypercoagulable state in critical COVID-19 (Extended Data Fig. 5).

**Fig. 1:**
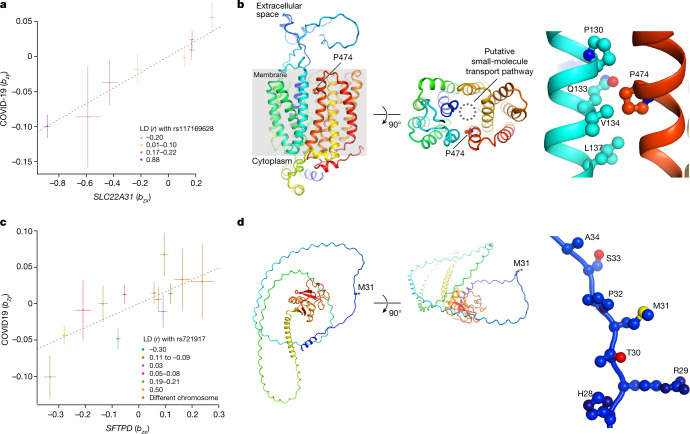
Functional genomics analyses for *SLC22A31* and *SFTPD*. **a**, Effect-size plot for the effect of multiple variants on *SLC22A31* expression (eQTLgen, *x* axis) against increasing susceptibility to critical COVID-19 (*β*_*xy*_ = 0.11; *P*_*xy*_ = 1.3 × 10^−9^). The colour shows linkage disequilibrium (LD) with the missense variant rs117169628. **b**, Three cartoon views of an AlphaFold^22^ model of putative solute carrier family 22 member 31 (SLC22A31; UniProtKB: A6NKX4). The side chains of Pro474 and interacting amino acids are shown as connected spheres. A putative channel for small-molecule transport across the cell membrane is indicated by a dashed circle. Pro474 is predicted to be located in the transmembrane helix and point towards a putative transport pathway of a small molecule. The risk variant, P474L (Ala at rs117169628) would be expected to introduce more flexibility to the transmembrane helix and might therefore affect the transport properties of SLC22A31. Pro474 is predicted to be in a tightly packed environment, and may therefore affect the folding of SLC22A31. **c**, Effect-size plot for effect of multiple variants on SFTPD expression (eQTLgen, *x* axis) against increasing susceptibility to critical COVID-19 (*β*_*xy*_ = 0.16; *P*_*xy*_ = 9.7 × 10^−6^). Colour shows linkage disequilibrium with the missense variant rs721917. **d**, Three cartoon views of an AlphaFold^22^ model of pulmonary surfactant-associated protein D (SFTPD; UniProtKB: P35247). The side chain of the variant Met31 is shown as connected spheres. Met31 is predicted to be located in the secondary-structure-lacking region of SFTPD. In the diagram on the right, oxygen and nitrogen atoms are coloured red and blue respectively, and the sulfur atom is coloured yellow.

**Extended Data Fig. 6:**
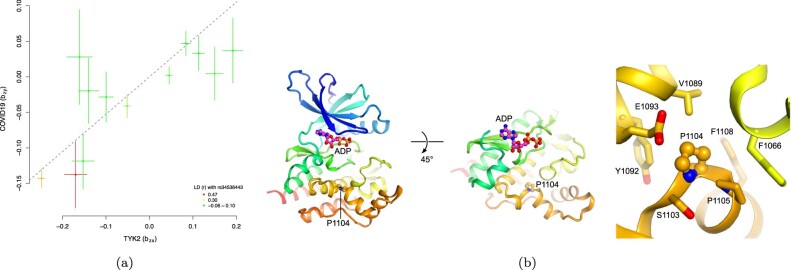
Functional genomics analyses for TYK2. (a) Effect size plot for effect of multiple variants on TYK2 expression (eQTLgen, x-axis) against increasing susceptibility to critical COVID-19 (${\beta }_{{xy}}$ = 0.53; ${P}_{{xy}}=1.2\times {10}^{-23}$). Colour shows linkage disequilibrium (LD) with the missense variant rs34536443. (b) Crystal structure of TYK2 kinase domain (Protein Data Bank ID 4GVJ^39^) in two views that differ by a 45° rotation around a horizontal axis. The side chain of P1104 is shown as connected spheres with a nitrogen atom coloured in blue. Carbon, oxygen, nitrogen and phosphorus atoms of ATP are shown as magenta, red, blue and orange connected spheres, respectively. The N-terminal region of the kinase domain is not shown in the second view for clarity. The right-most panel shows a close view of P1104 and neighbouring residues with their side chains shown as sticks. Numbering of residues corresponds to UniProtKB entry P29597. P1104 is in the catalytic kinase domain and proximal to the ATP-binding site; TYK2 P1104A is catalytically impaired^40^.

Three genes containing non-synonymous protein-coding changes associated with severe disease were also found to have significant effects from differential gene expression: *SLC22A31* (ref. ^2^) (Fig. 1), *SFTPD*^4^ (Fig. 1) and *TKY2* (ref. ^1^) (Extended Data Fig. 6). Further biological and clinical research will be required to dissect the genetic evidence at these loci. In the example of *TYK2*, there is now a therapeutic test of the genetic predictions. Our previous report of association between higher expression and critical illness^1^ led directly to the inclusion of a new drug, baricitinib, in a large clinical trial; the result demonstrated a clear therapeutic benefit^3^. This therapeutic signal is consistent across multiple trials, providing the first proof-of-concept for drug target identification using genetics in critical illness and infectious disease.

**Fig. 2:**
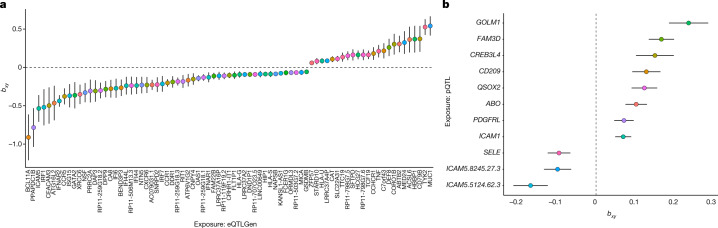
GSMR effect sizes. **a**,**b**, The predicted effect of change in protein concentration (**a**) and gene expression (**b**) on the risk of critical COVID-19 is shown for proteins and genes significantly linked to critical COVID-19 by GSMR (false-discovery rate (FDR) < 0.01). The bars show 95% confidence intervals.

To assess the immediate therapeutic use of our results for repurposing of existing compounds, we considered the drug therapies under consideration by the UK COVID-19 Therapeutic Advisory Panel (UK-CTAP), a national independent review group supported by an expert due-diligence panel^6^. Consistent evidence from gene-level GWAS (Supplementary Table 6 and Supplementary Table 10) and post-GWAS analyses was identified for several licensed compounds (Supplementary Table 12). For example, we found an association in another gene encoding a protein that is inhibited by baricitinib and other JAK inhibitors—the intracellular signalling kinase, *JAK1*, which is stimulated by numerous cytokines including type I interferons and IL-6. Mendelian randomization analysis of RNA expression revealed a significant positive association between the expression of the gene encoding a canonical inflammatory cytokine, tumour necrosis factor (*TNF*), and severe disease (Fig. 2). This suggests that inhibition of TNF signalling may be an effective therapy in severe COVID-19.

Our additional expression data in monocytes reveal a marked tissue-specific effect on expression of *PDE4A*. This phosphodiesterase regulates the production of multiple inflammatory cytokines by myeloid cells. In contrast to the negative correlations seen in the lungs and blood, we show that a genetic tendency for higher expression of *PDE4A* in monocytes is associated with critical COVID-19 (Supplementary Table 11). Inhibition of PDE4A by several existing drugs is under investigation in multiple inflammatory diseases^7^, reduces pulmonary endothelial permeability^8^ and appears to be safe in small clinical trials in patients with COVID-19.

The postulated biological role of genes associated with critical COVID-19 in GWAS, TWAS and GSMR results is shown in Extended Data Fig. 5, which highlights the preponderance of genes with expression or functions in the mononuclear phagocyte system. This includes *SLC2A5*, encoding the GLUT5 fructose transporter, which is strongly inducible in primary macrophages in response to inflammatory stimulation^9^, and *XCR1*, a dendritic cell receptor with a critical role in cytotoxic T cell-mediated antiviral immunity^10^. *NPNT*, a significant meta-TWAS association in the genome-wide significant region on chromosome 4 (chr4:105673359; Supplementary Table 11), encodes a pulmonary basement membrane protein that may have a protective role in acute lung injury^11^.

## Host–pathogen interaction

Our results also demonstrate the capacity of host genetics to reveal core mechanisms of disease. Multiple genes implicated in viral entry are associated with severe disease. In addition to *ACE2*, we detect a genome-wide significant association in *TMPRSS2*, a key host protease that facilitates viral entry that we have previously studied as a candidate gene^12^. This effect may be viral-lineage specific^13^. A strong GWAS association is seen in *RAB2A* (Table 1), with TWAS evidence suggesting that more expression of this gene is associated with worse disease (Supplementary Table 11). *RAB2A* is highly ranked in our previous meta-analysis by information content^14^ study of host genes implicated in SARS-CoV-2 interaction using in vitro and clinical data^15^, and is consistent with CRISPR screen data showing that *RAB2A* is required for viral replication^16^.

**Extended Data Fig. 7:**
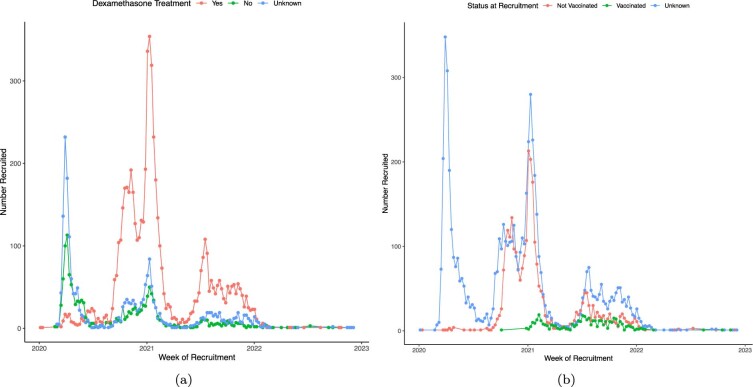
Steroid treatment and vaccination status. Data are shown for a subset of GenOMICC cases who were also recruited to the ISARIC4C study in the UK.

Although our focus on critical illness enhances discovery power (Extended Data Fig. 4), it has the disadvantage of combining genetic signals for multiple stages in disease progression, including viral exposure, infection and replication, and development of inflammatory lung disease. From these data alone we cannot identify when in disease progression the causal effect is mediated, although clinical evidence helps to make some predictions^17^ (Extended Data Fig. 5). As most cases included were recruited before vaccinations and treatments became available (Extended Data Fig. 7), at present, our study does not have sufficient statistical power to dissect the genetic effects of treatments or vaccination. These effects may include the masking of true associations, or the detection of genetic effects mediated by vaccine or drug response, rather than COVID-19 susceptibility. However, the absence of divergent genetic effects between studies (Supplementary Figs. 2–5) or consistent changes in effect allele frequency among cases over time (Supplementary Figs. 45–48) suggests that treatment and vaccination have not substantially affected the association between the specific variants that we report and the risk of critical illness.

As we performed a meta-analysis of multiple studies that may have slightly different definitions of the phenotype, effect sizes differ between studies (Supplementary Figs. 2–5). This, together with ancestry-specific effects^1^, may explain the heterogeneity in strong GWAS signals, such as the LZTFL1 signal in Table 1. Different studies also have sets of variants that are not completely overlapping, so *P* values between variants in high linkage disequilibrium are more different than expected. Although most of the studies contain individuals from multiple ancestries, a large majority of the individuals are of European ancestry. In future research, there is a scientific and moral imperative to include the full diversity of human populations.

Together, these results deepen our understanding of the pathogenesis of critical COVID-19 and highlight new biological mechanisms of disease, several of which have immediate potential for therapeutic targeting.

## Methods

### Hospitalization meta-analysis

The hospitalized phenotype includes patients who were hospitalized with a laboratory-confirmed SARS-Cov2 infection. In this analysis we included GenOMICC, GenOMICC Brazil, GenOMICC Saudi Arabia, ISARIC4C, HGIv6 B2 phenotype with subtraction of GenOMICC data, SCOURGE hospitalized versus population and mild cases, and 23andMe broad respiratory phenotype. A summary description of each analysis is given above, a table with the included studies can be found in Supplementary Table 14 and an extended description can be found in Supplementary Table 1.

### Critical illness meta-analysis

The critically ill COVID-19 group included patients who were hospitalized owing to symptoms associated with laboratory-confirmed SARS-CoV-2 infection and who required respiratory support or whose cause of death was associated with COVID-19. In the critical illness analysis, we included GenOMICC, patients with critical illness from ISARIC4C, HGIv6 phenotype A2 with subtraction of GenOMICC data, SCOURGE severity grades 3 and 4 versus population controls, and 23andMe respiratory support phenotype. A summary description of each analysis can be found above, a table with the included studies can be found in Supplementary Table 13 and an extended description can be found in Supplementary Table 1.

### Meta-analyses

All meta-analyses across studies were performed using a fixed-effect inverse-variance weighting method and control for population stratification in the METAL software^23^. Allele frequency was calculated as the average frequency across studies with the METAL option AVERAGEFREQ. *P* values for heterogeneity in effect sizes between studies were calculated using a Cochran’s *Q*-test implemented in METAL. For variants in the same position with different REF and ALT alleles across studies, the GenoMICC variant in the European population was selected and the rest were removed. Finally, variants with switched ALT and REF alleles between HGIv6 and GenOMICC were also removed on the basis of differences in allele frequency of the alternative allele. Variants were annotated to the closest genes using dbsnp v.b151 GRCh38p7 and bionrRt R package (v.2.46.3)^24^. As each single-nucleotide polymorphism (SNP) of the meta-analysis can be present in different subsets of cohorts, there may be large differences in *P* values in SNPs with a high level of linkage disequilibrium, which may have an effect on downstream analyses. For this reason, variants that were not present in one of the three biggest studies—GenOMICC European ancestry, HGIv6 or SCOURGE—were filtered out from post-GWAS analysis.

### Conditional analysis

We performed a step-wise conditional analysis to find independent signals. As European-specific data are not available in some cohorts but European ancestry is largely predominant (87.2% of cases with critical illness), we performed the conditional analysis using a European reference panel and the meta-analysis results of the whole cohort. To perform the conditional analysis, we used the GCTA (v.1.9.3) --cojo-slct function^25^. The parameters for the function were *P* = 5 × 10^−8^, a distance of 10,000 kb and a co-linear threshold of 0.9 (ref. ^26^), and the reference population for the conditional analysis was individuals of European ancestry with whole-genome sequence available in the GenOMICC study and whole genomes from the 100,000 Genomics England project^2^.

### Credible set fine-mapping

We performed fine-mapping using the SuSiE model^27^ to construct credible sets for the independent signals identified using conditional analysis. As for conditional analysis, we used a European reference panel and the meta-analysis results of the whole critical illness cohort. We performed analyses in 1 Mb windows centred on the lead variants identified through conditional analysis. In cases in which windows for multiple variants overlapped, they were joined into a single window. For each window, we fitted the SuSiE summary statistics model setting the expected number of independent signals to the number of identified though conditional analysis. Models for three windows did not converge in 500 iterations and have been excluded. As a reference, we used the publically available linkage disequilibrium information for non-Finish Europeans from the GNOMAD 2.1.1 release. Full data for all variants included in credible sets are included in Supplementary Table 5.

### Gene-level analysis

We performed an analysis summarizing the genetic associations at the gene level using the mBAT-combo method^28^. We used the COVID ‘all critical cohorts’ meta-analysis (GenOMICC, HGIv6 phenotype A2, SCOURGE and 23andMe) summary statistics. As this is a trans-ethnic meta-analysis, we used a mixed ancestry linkage disequilibrium reference panel, consisting of 3,202 1000 Genomes phase 3 samples. We considered a list of protein-coding genes with unique ensemble gene ID based on the release from GENCODE (v.40) for hg38, which can be found on the mBAT-combo website (https://yanglab.westlake.edu.cn/software/gcta/#mBAT-combo). A gene region was taken to span 50 kb upstream to 50 kb downstream of the gene’s untranslated regions.

### Sex-stratified meta-analysis

To test for differences in genetic effects, we performed sex-stratified GWAS of the COVID-19 critical illness phenotype in the European ancestry GenOMICC WGS and genotyped cohorts and SCOURGE. We then performed a meta-analysis for each sex following the same methods as for the main analysis. We tested for differences in effects between the meta-analyses of the two sexes following previously described methods^29^.

### Mendelian randomization

GSMR^5^ was performed. We used the COVID ‘all critical cohorts’ meta-analysis (GenOMICC, HGIv6 phenotype A2, SCOURGE and 23andMe) as the outcome, protein expression quantitative-trait loci (pQTLs) from ref. ^30^ and RNA expression quantitative-trait loci (eQTLs) from eQTLgen^31^ (2019-12-23 data release) as exposures, and 10,000 individuals of European ancestry randomly sampled from the UK Biobank as the linkage disequilibrium reference cohort (50,000 for linkage disequilibrium to missense variant plots). GSMR was performed for all exposures for which we were able to identify two or more suitable SNPs. SNPs were chosen to meet the following criteria: (1) SNP to exposure association *P* < 5 × 10^−8^; (2) linkage disequilibrium clumping lead SNPs only (±1 Mb, *r*^2^ < 0.05); (3) SNP not removed by HEIDI-outlier filtering (for the removal of SNPs with evidence of horizontal pleiotropy) at the default threshold value of 0.01. eQTLGen effect sizes and standard errors were estimated as described in supplementary note 2 of ref. ^32^. We considered as significant those exposure–outcome pairs with FDR < 0.05.

### TWAS analysis

To perform TWAS analysis in GTExv8 tissues^33^, we used the MetaXcan framework and the GTExv8 eQTL and sQTL MASHR-M models available for download online (http://predictdb.org/) and the ‘all critical cohorts’ meta-analysis. We first calculated individual TWAS for whole blood and lungs using the S-PrediXcan function^34,35^. We next performed a metaTWAS including data from all tissues to increase the statistical power using s-MultiXcan^36^. We applied Bonferroni correction to the results to choose significant genes and introns for each analysis.

### Monocyte gene expression

To detect eQTLs, untreated primary monocytes were prepared from 174 healthy individuals of Northern European (British) ancestry recruited through the Oxford Biobank. Poly(A) RNA was paired-end 100 bp sequenced in the Oxford Genome Centre using the Illumina HiSeq-4000 machines (median = 47,735,438 reads per sample). Reads were aligned to CRGh38/hg38 using HISAT2 with the default parameters. High mapping quality reads were selected on the basis of MAPQ score using bamtools. Duplicate reads were marked and removed using picard (v.1.105). Samtools was used to pass through the mapped reads and calculate statistics. Read count information was generated using HTSeq and normalized using DESeq2. Sample contamination and swaps were detected by comparing the imputed SNP-array genotypes with genotypes called from RNA-seq using verifyBamID. Genotyping was performed with Illumina HumanOmniExpress with coverage of 733,202 separate markers. Genotypes were pre-phased with SHAPEIT2, and missing genotypes were imputed with PBWT. Poly(A) RNA was paired-end sequenced at the Oxford Genome Centre using the Illumina HiSeq-4000 machines. vcftools (v0.1.12b) was applied on genetic variation data in the form of variant call format (VCF) files to filter out indels and SNPs with a minor allele frequency of less than 0.04.

TWAS analysis for monocyte data was performed using genotyping and monocyte RNA-sequencing data from 174 individuals. Using a region of 500 kb around each gene, we calculated gene expression models using the Fusion R package^37^. For each gene, three models were calculated adding as covariates the two first principal components calculated from the genotype: blup, elastic networks and lasso. The model with a better *r*^2^ between predicted and measured expression in a fivefold cross-validation was chosen. Then SNP genetic heritability was calculated for the 500 kb region for each gene and those genes with a nominal significant SNP heritability estimate (*P* ≤ 0.01) were chosen for the TWAS analysis. Summary statistics for the ‘all critical cohorts’ meta-analysis and the best model for each gene were then used to perform the TWAS.

### Colocalization

Significant genes in the TWAS and metaTWAS were selected for a colocalization analysis using the coloc R package. The lead SNPs and a region of 200 Mb around the gene were used to colocalize with significant genes in the TWAS with eQTL summary statistics data on the region from GTExv8 lung, GTExv8 whole blood, eQTLgen or monocyte eqtl. As in our previous analysis^2^, we first performed a sensitivity analysis of the posterior probability of colocalization (PPH4) on the prior probability of colocalization (P12), going from P12 = 10^−8^ to P12 = 10^−4^, with the default threshold being P12 = 10^−5^. eQTL signal and GWAS signals were deemed to colocalize if these two criteria were met: (1) at P12 = 5 × 10^−5^ the probability of colocalization PPH4 > 0.5; and (2) at P12 = 10^−5^ the probability of independent signal (PPH3) was not the main hypothesis (PPH3 < 0.5). These criteria were chosen to allow eQTLs with weaker *P* values, owing to lack of power in GTEx v.8, to be colocalized with the signal when the main hypothesis using small priors was that there was not any signal in the eQTL data.

### Effect comparison

We compared the estimates of effect sizes between the individual GWASs used in the meta-analysis, for all variants that were genome-wide significant in at least one of the individual GWASs. To this end, we regressed the effects obtained using critical illness and hospitalization in the SCOURGE and 23andMe cohorts, as well as the HGI meta-analyses on the effect estimates obtained using the GenOMICC cohort. To account for estimation errors present in both the dependent and independent variables of the regression we used orthogonal distance regression^38^.

### Weight of studies

To calculate the weight of GenOMICC, we downloaded the leave-one-out data of HGIv7. As the meta-analysis is performed using a variance-weighted method, we can recover the variance for each SNP as $v=\frac{1}{{{\rm{s.e.}}}^{2}}$, for the meta-analysis of all of the cohorts and for each one of the leave-one-out analysis. The total weight is ${w}_{{\rm{tot}}}=\frac{1}{v}$ and the weight leaving out a specific study is ${w}_{{\rm{loo}}}=\frac{1}{{v}_{{\rm{loo}}}}$. The weight of a cohort is then ${w}_{{\rm{tot}}}-{w}_{{\rm{loo}}}$. We calculated the weight for each the significant SNPs in our analysis for each study and normalized it using the total weight. Finally, we calculated the mean and s.d. from the significant SNPs for each cohort.

### Forest plots

To compare effects between cohorts, we first performed a trans-ancestry meta-analysis for GenOMICC and 23andMe using METAL^23^. Then, we used the metagen and forest functions of the meta R package to produce forest plots for critical illness and hospitalization separately.

### Reporting summary

Further information on research design is available in the Nature Portfolio Reporting Summary linked to this article.

## Online content

Any methods, additional references, Nature Portfolio reporting summaries, source data, extended data, supplementary information, acknowledgements, peer review information; details of author contributions and competing interests; and statements of data and code availability are available at 10.1038/s41586-023-06034-3.

### Supplementary information

Supplementary Information Supplementary Sections 1–13, including Supplementary Figs. 1–54 and Supplementary Tables 7–17.

Reporting Summary

Peer Review File

Supplementary Table 1 Description of the cohorts used in critical and hospitalized meta-analyses. Cohorts are divided by ancestry and genotyping method (whole-genome sequencing or microarray genotyping). In cohorts in which data are available, the median age with s.d. in parentheses, percentage of female cases, number of female and male cases and controls is shown. NA, data are not available for the cohort. Country of origin indicates the country in which individuals in the cohort were recruited; GenotypingPlatform indicates the array or WGS platform used for genotyping; reference indicates the reference of the publication (if the data have already been published). ‘In GenOMICC v2’ is an indication of whether the dataset was included in a previous GenOMICC paper^1^.

Supplementary Table 2 Full results for colocalization and TWAS analyses in lungs, blood, monocytes and across multiple tissue types (metaTWAS). Colocalization results are reported between significant TWAS genes and eQTLs in GTExv8 in lungs, blood and monocytes, and in eqtlGEN. rsid indicates chromosome, position, reference and alternative alleles; gene.tested is the significant TWAS gene, and ensembl.id is the Ensembl ID corresponding to the significant gene. PP.H3.1e-5 is the posterior probability of independent signals with a prior for colocalization of 5 × 10^−5^, PP.H4.1e-5 is the posterior probability of colocalization with a prior of 5 × 10^−5^, PP.H3.5e-5 is the posterior probability of independent signals with a prior for colocalization of 5 × 10^−5^ and PP.H4.5e-5 is the probability of colocalization with a prior of 5 × 10^−5^. Colocalization was considered to be significant when PP.H3 was lower than 0.5 and PP.H4 was the highest posterior probability. Significant genes after Bonferroni correction in a TWAS meta-analysis of lungs, blood, monocytes and all tissues in GTExv8 and the ‘all critical cohorts’ GWAS. Ensembl ID, gene name, *P* value of the meta-analysis, number of SNPs used in to model gene expression in all tissues, mean *z* score for all tissues and its s.d. are shown.

Supplementary Table 3 The full results from GSMR analysis for protein level. Exposure indicates the protein name used as exposure for the analysis, bxy is the effect size, se is the standard error, p is the *P* value of the analysis, nsnp is the number of SNPs used as instruments and multi_snp_based_heidi_outlier is the *P* value of the Heidi test.

Supplementary Table 4 Full results from GSMR analysis for RNA-seq data from eQTLGEN. Exposure indicates the gene name used as exposure for the analysis, bxy is the effect size, se is the standard error, p is the *P* value of the analysis, nsnp is the number of SNPs used as instruments and multi_snp_based_heidi_outlier is the *P* value of the Heidi test.

Supplementary Table 5 Table of variants in credible sets with 95% probability of containing the causal SNP. Credible set ID is the credible set index to which the variant belongs; posterior is the posterior probability of causality for the variant. Variant indicates chromosome, position, reference and alternative alleles; beta is the effect, beta.se is the error and P is the *P* value.

Supplementary Table 6 The full results of the gene-level analysis performed using the mBAT-combo method. Gene indicates ensembl ID, gene_name indicates name of the gene, gene_p is the *P* value of the gene-level test, nsps is the number of SNPs used for the test, lead_snp is the chromosome, position, reference and alternative alleles for the SNPs with lowest *P* value in the region, and lead_snp_p indicates the *P* value of this lead SNP.

## Figures and Tables

**Extended Data Table 1:**
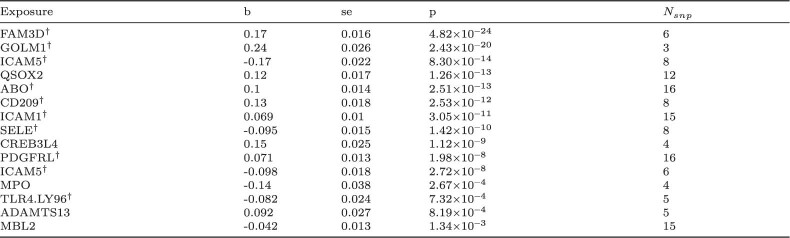
pQTL GSMR results table

Proteins significantly linked to COVID-19 severity (false discovery rate (FDR) $$ < 0.05$$). Exposure: protein name; b: effect-size estimate of the protein on COVID-19 severity from GSMR; se: standard-error of b; p: p-value of the GSMR result; N: Number of independent SNPs included in the analysis. $$\dagger :$$ indicates proteins with GSMR evidence previously presented in Kousathanas et al.^2^.

**Extended Data Table 2:**
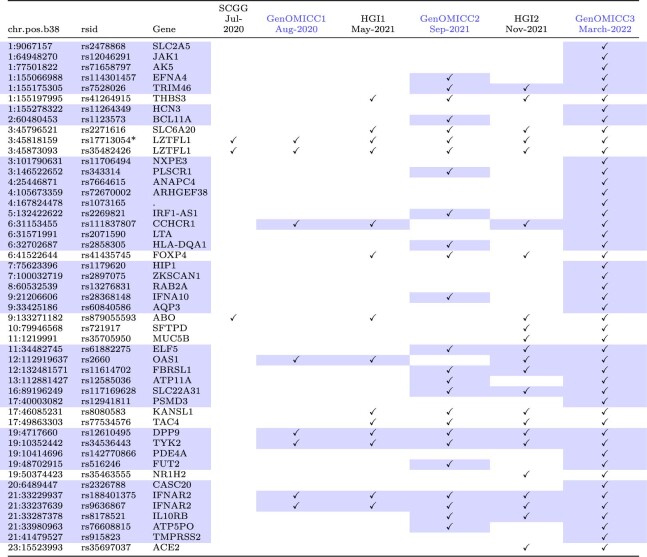
Replication table

Each report of genome-wide significant associations with severe COVID-19 is shown, with associations that were first reported by the GenOMICC consortium are highlighted in blue.

## Data Availability

Downloadable summary data are available through the GenOMICC data site (https://genomicc.org/data). Summary statistics are available, but without the 23andMe summary statistics, except for the 10,000 most significant hits, for which full summary statistics are available. The full GWAS summary statistics for the 23andMe discovery dataset will be made available through 23andMe to qualified researchers under an agreement with 23andMe that protects the privacy of the 23andMe participants. For further information and to apply for access to the data, see the 23andMe website (https://research.23andMe.com/dataset-access/). All individual-level genotype and whole-genome sequencing data (for both academic and commercial uses) can be accessed through the UKRI/HDR UK Outbreak Data Analysis Platform (https://odap.ac.uk). A restricted dataset for a subset of GenOMICC participants is also available through the Genomics England data service. Monocyte RNA-seq data are available under the title ‘Monocyte gene expression data’ within the Oxford University Research Archives (10.5287/ora-ko7q2nq66). Sequencing data will be made freely available to organizations and researchers to conduct research in accordance with the UK Policy Framework for Health and Social Care Research through a data access agreement. Sequencing data have been deposited at the European Genome–Phenome Archive (EGA), which is hosted by the EBI and the CRG, under accession number EGAS00001007111.

## Code availability

Code to calculate the imputation of *P* values on the basis of SNPs in linkage disequilibrium is available at GitHub (https://github.com/baillielab/GenOMICC_GWAS).
