# Author Correction: GWAS and meta-analysis identifies 49 genetic variants underlying critical COVID-19

**DOI:** 10.1038/s41586-023-06383-z

**Published:** 2023-07-11

**Authors:** Erola Pairo-Castineira, Konrad Rawlik, Andrew D. Bretherick, Ting Qi, Yang Wu, Isar Nassiri, Glenn A. McConkey, Marie Zechner, Lucija Klaric, Fiona Griffiths, Wilna Oosthuyzen, Athanasios Kousathanas, Anne Richmond, Jonathan Millar, Clark D. Russell, Tomas Malinauskas, Ryan Thwaites, Kirstie Morrice, Sean Keating, David Maslove, Alistair Nichol, Malcolm G. Semple, Julian Knight, Manu Shankar-Hari, Charlotte Summers, Charles Hinds, Peter Horby, Lowell Ling, Danny McAuley, Hugh Montgomery, Peter J. M. Openshaw, Colin Begg, Timothy Walsh, Albert Tenesa, Carlos Flores, José A. Riancho, Augusto Rojas-Martinez, Pablo Lapunzina, GenOMICC Investigators, Sara Clohisey, Johnny Millar, Manu Shankar-Hari, Emma Aitkin, Latha Aravindan, Ruth Armstrong, J. Kenneth Baillie, Heather Biggs, Ceilia Boz, Adam Brown, Primmy Chikowore, Richard Clark, Audrey Coutts, Judy Coyle, Louise Cullum, Sukamal Das, Nicky Day, Lorna Donnelly, Esther Duncan, Paul Finernan, Max Head Fourman, Anita Furlong, James Furniss, Bernadette Gallagher, Tammy Gilchrist, Ailsa Golightly, Fiona Griffiths, Katarzyna Hafezi, Debbie Hamilton, Ross Hendry, Naomi Kearns, Dawn Law, Rachel Law, Sarah Law, Rebecca Lidstone-Scott, Christen Lauder, Louise Macgillivray, Alan Maclean, Hanning Mal, Sarah McCafferty, Ellie McMaster, Jen Meikle, Shona C. Moore, Sheena Murphy, Hellen Mybaya, Miranda Odam, Wilna Oosthuyzen, Chenqing Zheng, Jiantao Chen, Nick Parkinson, Trevor Paterson, Petra Tucker, Katherine Schon, Andrew Stenhouse, Mihaela Das, Maaike Swets, Helen Szoor-McElhinney, Filip Taneski, Lance Turtle, Tony Wackett, Mairi Ward, Jane Weaver, Nicola Wrobel, Marie Zechner, Jacqueline Pan, Neus Grau, Tim Owen Jones, Rosario Lim, Martina Marotti, Christopher Whitton, Aneta Bociek, Sara Campos, Gill Arbane, Manu Shankar-Hari, Marlies Ostermann, Mina Cha, Fabiola DAmato, Eirini Kosifidou, Shelley Lorah, Kyma Morera, Sarah Bircham, Laura Brady, Keith Hugill, Jeremy Henning, Stephen Bonner, Evie Headlam, Jessica Jones, Abigail List, Joanne Morley, Amy Welford, Bobette Kamangu, Anitha Ratnakumar, Abiola Shoremekun, Zoe Alldis, Raine Astin-Chamberlain, Fatima Bibi, Jack Biddle, Sarah Blow, Matthew Bolton, Catherine Borra, Ruth Bowles, Maudrian Burton, Yasmin Choudhury, Amber Cox, Amy Easthope, Patrizia Ebano, Stavros Fotiadis, Jana Gurasashvili, Rosslyn Halls, Pippa Hartridge, Delordson Kallon, Jamila Kassam, Ivone Lancoma-Malcolm, Maninderpal Matharu, Peter May, Oliver Mitchelmore, Tabitha Newman, Mital Patel, Jane Pheby, Irene Pinzuti, Zoe Prime, Oleksandra Prysyazhna, Julian Shiel, Melanie Taylor, Carey Tierney, Olivier Zongo, Suzanne Wood, Anne Zak, David Collier, Manuela Mundy, Christopher Thompson, Lisa Pritchard, Minnie Gellamucho, David Cartlidge, Nageswar Bandla, Lucy Bailey, Michelle Davies, Jane Delaney, Leanne Scott, Marwa Abdelrazik, Frater Alasdair, David Carter, Munzir Elhassan, Arunkumar Ganesan, Samuel Jenkins, Zoe Lamond, Dharam Purohit, Kumar Rohit, Malik Saleem, Alanna Wall, Kugan Xavier, Dhanalaksmi Bakthavatsalam, Kirolos Gehad, Pakeerathan Gnanapragasam, Kapil Jain, Swati Jain, Abdul Malik, Naveen Pappachan, Jeronimo Moreno-Cuesta, Anne Haldeos, Rachel Vincent, Maryjane Oziegb, Anna Cavazza, Maeve Cockrell, Eleanor Corcoran, Maria Depante, Clare Finney, Ellen Jerome, Abigail Knighton, Monalisa Nayak, Evita Pappa, Rohit Saha, Sian Saha, Andrew Dodd, Kevin O’Reilly, Mark McPhail, Emma Clarey, Harriet Noble, John Smith, Phoebe Coghlan, Stephen Brett, Anthony Gordon, Maie Templeton, David Antcliffe, Dorota Banach, Sarah Darnell, Ziortza Fernandez, Eleanor Jepson, Amal Mohammed, Roceld Rojo, Sonia Sousa Arias, Anita Tamang Gurung, Jenny Wong, Jaime Fernandez-Roman, David O. Hamilton, Emily Johnson, Brian Johnston, Maria Lopez Martinez, Suleman Mulla, Alicia A. C. Waite, Karen Williams, Victoria Waugh, Ingeborg Welters, Jessica Emblem, Maria Norris, David Shaw, Archana Bashyal, Sally Beer, Paula Hutton, Stuart McKechnie, Neil Davidson, Soya Mathew, Grace Readion, Jung Ryu, Jean Wilson, Shruti Agrawal, Kay Elston, Megan Jones, Eoghan Meaney, Petra Polgarova, Muhammad Elbehery, Charlotte Summers, Esther Daubney, Anthony Ng, Jocelyn Marshall, Nazima Pathan, Katerina Stroud, Deborah White, Angela Andrew, Saima Ashraf, Amy Clark, Martin Dent, Margaret Langley, Cecilia Peters, Lucy Ryan, Julia Sampson, Shuying Wei, Alice Baddeley, Megan Meredith, Lucy Morris, Alexandra Gibbons, Lisa McLoughlin, Carlos Castro Delgado, Victoria Clark, Deborah Dawson, Lijun Ding, Georgia Durrant, Obiageri Ezeobu, Abiola Harrison, William James Hurt, Rebecca Kanu, Ashley Kinch, Susannah Leaver, Ana Lisboa, Jisha Mathew, Kamal Patel, Romina Pepermans Saluzzio, John Rawlins, Tinashe Samakomva, Nirav Shah, Christine Sicat, Joana Texeira, Joana Gomes De Queiroz, Edna Fernandes Da Gloria, Elena Maccacari, Nikki Yun, Soumendu Manna, Sarah Farnell-Ward, Maria Maizcordoba, Maria Thanasi, Hawakin Haji Ali, Janice Hastings, Lina Grauslyte, Musarat Hussain, Bobby Ruge, Sam King, Tatiana Pogreban, Lace Rosaroso, Helen Smith, Mandeep-Kaur Phull, Nikkita Adams, George Franke, Aparna George, Erika Salciute, Joanna Wong, Karen Dunne, Luke Flower, Emma Sharland, Sukhmani Sra, Gillian Andrew, Marie Callaghan, Lucy Barclay, Lucy Marshall, Kenneth Baillie, Maria Amamio, Sophie Birch, Kate Briton, Sarah Clark, Katerine Doverman, Dave Hope, Corrienne Mcculloch, Scott Simpson, Jo Singleton, Rita Fernandez, Meryem Allen, David Baptista, Rebecca Crowe, Jonathan Fox, Jacyntha Khera, Adam Loveridge, India McKenley, Eriko Morino, Andres Naranjo, Denise O’Connor, Richard Simms, Kathryn Sollesta, Andrew Swain, Harish Venkatesh, Rosie Herdman-Grant, Anna Joseph, Angela Nown, Steve Rose, David Pogson, Helen Boxall, Lutece Brimfield, Helen Claridge, Zoe Daly, Shenu George, Andrew Gribbin, Yusuf Cheema, Sean Cutler, Owen Richards, Anna Roynon-Reed, Shiney Cherian, Anne Emma Heron, Gemma Williams, Tamas Szakmany, Abby Waters, Kim Collins, Jill Dunhill, Ffion Jones, Rebecca Morris, Lucy Ship, Amy Cardwell, Syamlan Ali, Ravi Bhatterjee, Rachel Bolton, Srikanth Chukkambotla, Dabheoc Coleman, Jack Dalziel, Joseph Dykes, Christopher Fine, Bethan Gay, Wendy Goddard, Drew Goodchild, Rhiannan Harling, Muhammad Hijazi, Sarah Keith, Meherunnisa Khan, Roseanna Matt, Janet Ryan-Smith, Samuel Saad, Philippa Springle, Jacqueline Thomas, Nick Truman, Aayesha Kazi, Matthew Smith, Heather Collier, Chloe Davison, Stephen Duberley, Jeanette Hargreaves, Janice Hartley, Tahera Patel, Ellen Smith, Alissa Kent, Emma Goodwin, Ahmed Zaki, Clare Tibke, Susan Hopkins, Hywel Gerrard, Matthew Jackson, Sara Bennett, Liane Marsh, Rebecca Mills, Jessica Bell, Helen Campbell, Angela Dawson, Steve Dodds, Stacey Duffy, Lisa Gallagher, Gemma McCafferty, Stacey Short, Tracy Smith, Kirsty Thomas, Claire Walker, Jessica Reynolds, Bryan Yates, Hayley McKie, Maria Panteli, Maria Thompson, Gail Waddell, Sarah De Beger, Azmerelda Abraheem, Charlie Dunmore, Rumanah Girach, Rhianna Jones, Emily London, Imrun Nagra, Farah Nasir, Hannah Sainsbury, Clare Smedley, Stephen Brearey, Caroline Burchett, Kathryn Cawley, Maria Faulkner, Helen Jeffrey, Peter Bamford, Firdaus Shaikh, Lauren Slack, Angela Davies, Hollie Brooke, Jose Cebrian Suarez, Ruth Charlesworth, Karen Hansson, John Norris, Alice Poole, Rajdeep Sandhu, Elizabeth Smithson, Muthu Thirumaran, Veronica Wagstaff, Sarah Buckley, Brendan Sloan, Alastair Rose, Amy Major, Alexandra Metcalfe, Christine Almaden-Boyle, Pauline Austin, Susan Chapman, Alexandre Eros, Louise Cabrelli, Stephen Cole, Clare Whyte, Matt Casey, Vasileios Bafitis, George Tsinaslanidis, Cassandra George, Reena Khade, Christopher Black, Sundar Raj Ashok, Sean Farley, Elaine Brinkworth, Rachel Harford, Carl Murphy, Marie Williams, Luke Newey, Hannah Toghill, Sophie Lewis, Tabitha Rees, Ceri Battle, Mark Baker, Jenny Travers, Karen Chesters, Nicola Baxter, Andrew Arnott, Gordan McCreath, Christopher McParland, Laura Rooney, Malcolm Sim, Steven Henderson, Lynn Abel, Carol Dalton, Sophie Kennedy-Hay, Lynn O’Donohoe, Megan O’Hare, Izabela Orlikowska, Natasha Parker, Fiona McNeela, Amanda Lyle, Alistair Hughes, Jayachandran Radhakrishnan, Sian Gibson, Hollie Bancroft, Mary Bellamy, Jacqueline Daglish, Salma Kadiri, Faye Moore, Joanne Rhodes, Mirriam Sangombe, Zhane Peterkin, James Scriven, Margaret Carmody, Juliet Cottle, Emily Peasgood, Laura Ortiz-Ruiz de Gordoa, Claire Phillips, Denise Skinner, Zoe Cinquina, Kate Howard, Rosie Joy, Samantha Roche, Isobel Birkinshaw, Joseph Carter, Jo Ingham, Nicola Marshall, Harriet Pearson, Zoe Scott, Jo Dasgin, Jaspret Gill, Annette Nilsson, Amy Bamford, Diana Hull, James Scriven, Nafeesah Ahmadhaider, Michelle Bates, Christopher McGhee, Hannah Ellis, Gwenllian Sera Howe, Jayaprakash Singh, Natalie Stroud, Lisa Roche, Ceri Lynch, Bethan Deacon, Carla Pothecary, Justyna Smeaton, Kevin Agravante, Vinodh Krishnamurthy, Cynthia Diaba, Lincy John, Lai Lim, Rajeev Jha, Jasmine Egan, Timothy Felton, Susannah Glasgow, Grace Padden, Ozerah Choudhr, Joanne Bradley-Potts, Stuart Moss, Saejohn Lingeswaran, Peter Alexander, Craig Brandwood, Sofia Fiouni, Luke Ward, Schvearn Allen, Jane Shaw, Christopher Smith, Oluronke Adanini, Rebecca Collins, Maines Msiska, Linda Ofori, Nikhil Bhatia, Hayley Dolan, Mark Brunton, Jess Caterson, Holly Coles, Liza Keating, Emma Tilney, Nicola Jacques, Matthew Frise, Jennifer Armistead, Shauna Bartley, Parminder Bhuie, Sabi Rai, Gabriela Tomkova, Sandra Greer, Karen Shuker, Ascanio Tridente, Emma Dobson, Jodie Hunt, Redmond Tully, Joy Dearden, Andrew Drummond, Prakash Kamath, Emily Bullock, Michelle Mulcahy, Shelia Munt, Grainne O’Connor, Jennifer Philbin, Chloe Rishton, Chloe Scott, Sarah Winnard, Nurkamalia Hasni, Rachel Gascoyne, Joanne Hawes, Kelly Pritchard, Lesley Stevenson, Amanda Whileman, Sarah Beavis, Lauren Bishop, Cindy Cart, Katie Dale, Mary Kelly-Baxter, Adam Mendelski, Emma Moakes, Rheanna Smith, Jan Woodward, Stephanie Wright, Angela Allan, Adriana Botello, Jade Liew, Jasmine Medhora, Erin Trumper, Felicity Savage, Teresa Scott, Marc Place, Callum Kaye, Sarah Benyon, Suzie Marriott, Linda Park, Helen Quinn, Daisy Skyes, Lily Zitter, Kizzy Baines, Elizabeth Gordon, Samantha Keenan, Andrew Pitt, Katharine Duffy, Jane Ireland, Gary Semple, Lynne Turner, Susanne Cathcart, Dominic Rimmer, Alex Puxty, Kathryn Puxty, Andrew Hurst, Jennifer Miller, Susan Speirs, Lauren Walker, Zena Bradshaw, Joanna Brown, Sarah Melling, Stephen Preston, Nicola Slawson, Scott Warden, Alanna Beasley, Emma Stoddard, Leonie Benham, Jason Cupitt, Melanie Caswell, Lisa Elawamy, Ashleigh Wignall, Belinda Roberts, Hannah Golding, Samantha Leggett, Michelle Male, Martyna Marani, Kirsty Prager, Toran Williams, Kim Golder, Oliver Jones, Rebecca Cusack, Clare Bolger, Rachel Burnish, Michael Carter, Susan Jackson, Karen Salmon, Jonathan Biss, Maia Aquino, Maria Croft, Victoria Frost, Ian White, Keshnie Govender, Natasha Webb, Liana Stapleton, Colin Wells, Nikitas Nikitas, Ana Sanchez-Rodriguez, Kayleigh Spencer, Bethan Stowe, Yvonne Izzard, Michelle Poole, Sonja Monnery, Sallyanne Trotman, Valerie Beech, Edward Combes, Teishel Joefield, Patrick Covernton, Sarah Savage, Elizabeth Woodward, Julie Camsooksai, Henrik Reschreiter, Charlotte Barclay, Yasmin DeAth, Judith Dube, Charlotte Humphrey, Sarah Jenkins, Emma Langridge, Rebecca Milne, Beverley Wadams, Megan Woolcock, Michael Brett, Brian Digby, Lisa Gemmell, James Hornsby, Patrick MacGoey, Pauline O’Neil, Richard Price, Radha Sundaram, Lynn Abel, Natalie Rodden, Nicola Thomson, Kevin Rooney, Susan Currie, Natasha Parker, Lauren Walker, Philip Henderson, Bethan Ogg, Simon Whiteley, Liz Wilby, Kate Long, Shailamma Matthew, Sheila Salada, Susan Trott, Sarah Watts, Zoe Friar, Abigail Speight, Victoria Bastion, Humza Chandna, Brice Djeugam, Muhammad Haseeb, Harriet Kent, Gamu Lubimbi, Sophie Murdoch, Alastair Thomas, Beena David, Rachel Lorusso, Ana Vochin, Melchizedek Penacerrada, Retno Wulandari, Charlotte Heath, Srinivas Jakkula, Anna Morris, Ashar Ahmed, Arvind Nune, Claire Buttriss, Emma Whitaker, Miriam Davey, David Golden, Amy Acklery, Fabio Fernandes, Bec Seaman, Victoria Earl, Amy Collins, Waqas Khaliq, Rachel Adam, Estefania Treus, Sarah Holland, Jordan Alfonso, Bethan Blackledge, Michelle Bruce, Laura Jayne Durrans, Ayaa Eltayeb, Jade Harris, Samuel Hey, Martin Hruska, Thomas Lamb, Joanne Rothwell, Adele Fitzgerald, Gabriella Lindergard, Helen T-Michael, Tracey Duncan, Sharon Baxter-Dore, Lisa Cooper, Claire Fox, Jacinta Guerin, Tracey Hodgkiss, Karen Connolly, Paul McAlinden, Victoria Bridgett, Maggie Fearby, A. Gulati, Helen Hanson, Sinead Kelly, Louise McCormack, Rachel Nixon, Philip Robinson, Victoria Slater, Elaine Stephenson, Andrea Webster, K. Webster, Carole Hays, Anne Hudson, Bijal Patel, Ian Clement, John Davis, Sarah Francis, Douglas Jerry, Caroline Abernathy, Louise Foster, Andrew Gratrix, Llucia Cabral-Ortega, Matthew Hines, Victoria Martinson, Elizabeth Stones, Karen Winter, Esther Barrow, Katharine Wylie, Deborah Baines, Katie Birchall, Laurel Kolakaluri, Richard Clark, Anila Sukumaran, Craig Brandwood, Melanie Barker, Deborah Paripoorani, Lara Smith, Charlotte Taylor, Charlotte Downes, Melanie Hayman, Katie Riches, Priya Daniel, Deepak Subramanian, Kathleen Holding, Mary Hilton, Carly McDonald, Georgina Richardson, Georgia Halladay, Peter Harding, Amie Reddy, Ian Turner-Bone, Laura Wilding, Robert Parker, Michaela Lloyd, Leanne Smith, Charlie Kelly, Maria Lazo, Alan Neal, Olivia Walton, Julie Melville, Jay Naisbitt, Emily Bullock, Rosane Joseph, Sara Callam, Lisa Hudig, Jocelyn Keshet-Price, Katie Stammers, Karen Convery, Georgina Randell, Deirdre Fottrell-Gould, Esther Mwaura, Sara-Beth Sutherland, Richard Stewart, Louise Mew, Lynn Wren, Laura Thrasyvoulou, Heather Willis, James Scriven, Bridget Hopkins, Daniel Lenton, Abigail Roberts, Maria Bokhari, Rachael Lucas, Wendy McCormick, Jenny Ritzema, Vanessa Linnett, Amanda Sanderson, Helen Wild, Rebecca Flanagan, Robert Hull, Kat Rhead, Emma McKenna, Gareth Hughes, Jennifer Anderson, Kelly Jones, Scott Latham, Heather Riley, Martina Coulding, Martyn Clark, Jacqueline McCormick, Oliver Mercer, Darsh Potla, Hafiz Rehman, Heather Savill, Victoria Turner, Edward Jude, Susan Kilroy, Elena Apetri, Cathrine Basikolo, Bethan Blackledge, Laura Catlow, Matthew Collis, Reece Doonan, Jade Harris, Alice Harvey, Karen Knowles, Stephanie Lee, Diane Lomas, Chloe Lyons, Liam McMorrow, Angiy Michael, Jessica Pendlebury, Jane Perez, Maria Poulaka, Nicola Proudfoot, Kathryn Slevin, Vicky Thomas, Danielle Walker, Paul Dark, Bethan Charles, Danielle McLaughlan, Melanie Slaughter, Dan Horner, Kathryn Cawley, Tracy Marsden, Joyann Andrews, Emily Beech, Olugbenga Akinkugbe, Alasdair Bamford, Holly Belfield, Gareth A. L. Jones, Tara McHugh, Hamza Meghari, Samiran Ray, Ana Luisa Tomas, Lauran O’Neill, Mark Peters, Michael Bell, Sarah Benkenstein, Catherine Chisholm, Charlene Davies, Klaudia Kupiec, Caroline Payne, Joanna Halls, Hayley Blakemore, Elizabeth Goff, Kati Hayes, Kerry Smith, Deanna Stephens, Ruth Worner, Borislava Borislavova, Beverley Faulkner, Matt Thomas, Ruth Cookson, Emma Gendall, Georgina Larman, Rebecca Pope, Artur Smalira, Victoria Priestley, Tracey Cosier, Gemma Millen, James Rand, Natasha Schumacher, Roxana Sandhar, Heather Weston, Neil Richardson, Lucy Cooper, Cathy Jones, Ya-Wen Jessica Huang, Reni Jacob, Craig Denmade, Lewis McIntyre, Dawn Trodd, Jane Martin, Geoff Watson, Emily Bevan, Caroline Wreybrown, Shereen Bano, Ruth Bellwood, Michael Bentley, Matt Bromley, Lucy Gurr, Camilla Ledgard, Janet McGowan, Kate Pye, Kirsten Sellick, Amelia Stacey, Deborah Warren, Brian Wilkinson, Louise Akeroyd, Huma Shafique, James Morgan, Susan Shorter, Rachel Swinger, Emily Waters, Tom Lawton, Elizabeth Allan, Kate Darlington, Ffyon Davies, Llinos Davies, Jack Easton, Sumit Kumar, Richard Lean, Callum Mackay, Richard Pugh, Xinyi Qiu, Stephanie Rees, Jeremy Scanlon, Joanne Lewis, Daniel Menzies, Annette Bolger, Gwyneth Davies, Jennifer Davies, Esther Garrod, Helen Jones, Rachel Manley, Hannah Williams, Jordan Frankham, Sally Pitts, Nigel White, Debbie Branney, Heather Tiller, Georgia Efford, Zoe Garland, Lisa Grimmer, Bethany Gumbrill, Rebekah Johnson, Katie Sweet, Jeremy Bewley, Christina Coleman, Katie Corcoran, Eva Maria Hernandez Morano, Rachel Shiel, Denise Webster, Josephine Bonnici, Eleanor Daniel, Abbie Dell, Melanie Kent, Ami Wilkinson, Ellen Brown, Andrea Kay, Suzanne Campbell, Amanda Cowton, Mark Birt, Vicki Greenaway, Kathryn Potts, Clare Hutton, Andrew Shepperson, Miranda Forsey, Alice Nicholson, Mark Vertue, Joanne Riches, Agilan Kaliappan, Anne Nicholson, Niall MacCallum, Eamon Raith, Georgia Bercades, Ingrid Hass, David Brealey, Gladys Martir, Anna Reyes, Deborah Smyth, Maria Zapatamartinez, Ana Alvaro, Champa Jetha, Louise Ma, Lauren Booker, Loreta Mostoles, Anezka Pratley, Abdelhakim Altabaibeh, Chetan Parmar, Kayleigh Gilbert, Susie Ferguson, Amy Shepherd, Sheila Morris, Jo Singleton, Rosie Baruah, Maria Amamio, Sophie Birch, Kate Briton, Sarah Clark, Katherine Doverman, Lucy Marshall, Scott Simpson, Georgina Lloyd, Stephanie Bell, Vanessa Rivers, Bally Purewal, Kate Hammerton, Susan Anderson, Janine Birch, Emma Collins, Ryan Oleary, Sarah Cornell, Jordan Jarmain, Kimberley Rogerson, Fiona Wakinshaw, Lindsey Woods, Anthony Rostron, Zeynep Elcioglu, Alistair Roy, Gillian Bell, Holly Dickson, Louise Wilcox, Amro Katary, Katy English, Joanne Hutter, Corinne Pawley, Patricia Doble, Charmaine Shovelton, Marius Vaida, Rebecca Purnell, Ashly Thomas, Lenka Cagova, Adama Fofano, Helen Holcombe, Alice Michael Mitchell, Lucy Mwaura, Krithivasan P. Raman, Lucie Garnr, Sue Mepham, Kitty Paques, Alain Vuylsteke, Jennifer Mackie, Carmen Pearn, Julie Zamikula, Mark Birt, Estefania Treus Gude, Maggie Nyirenda, Lisa Capozzi, Rosie Reece-Anthony, Waqas Khaliq, Hazma Noor, Alfa Cresia Nilo, Michelle Grove, Amelia Daniel, Amy Easthope, Joanne Finn, Nikki White, Rajnish Saha, Bibi Badal, Karen Ixer, Donna Duffin, Ben Player, Helen Hill, Jade Cole, Jenny Brooks, Michelle Davies, Rhys Davies, Lauren Hunt, Emma Thomas, Angharad Williams, Metod Oblak, Mini Thankachen, Jamie Irisari, Amrinder Sayan, Monica Popescu, Cheryl Finch, Andrew Jamieson, Alison Quinn, Joshua Cooper, Sarah Liderth, Natalia Waddington, Iona Burn, Katarina Manso, Ruth Penn, Julie Tebbutt, Danielle Thornton, James Winchester, Geraldine Hambrook, Pradeep Shanmugasundaram, Jayne Craig, Kerry Simpson, Andrew Higham, Louise Sibbett, Sheila Paine, Annabel Reed, Jo-Anna Conyngham, McDonald Mupudzi, Rachel Thomas, Mary Wright, Denise Griffin, Richard Partridge, Maria Alvarez Corral, Nycola Muchenje, Mildred Sitonik, Caroline Wrey Brown, Aaron Butler, Linda Folkes, Heather Fox, Amy Gardner, David Helm, Gillian Hobden, Kirsten King, Jordi Margalef, Michael Margarson, Tim Martindale, Emma Meadows, Dana Raynard, Yvette Thirlwall, Yolanda Baird, Raquel Gomez, Darren Martin, Luke Hodgson, Clinton Corin, Erikka Sidall, Densie Szabo, Sharon Floyd, Hannah Davies, Karen Austin, Olivia Kelsall, Hannah Wood, Peter Anderson, Katie Archer, Andrew Burtenshaw, Sarah Clayton, Naiara Cother, Nicholas Cowley, Caroline Davis, Stephen Digby, Alison Durie, Alison Harrison, Emma Low, Michael McAlindon, Alex McCurdy, Aled Morgan, Tobias Rankin, Jessica Thrush, Helen Tranter, Charlie Vigurs, Laura Wild, Thomas Cornell, Kate Ralph, Sarah Bean, Karen Burt, Michael Spivey, Carol Richards, Rachel Tedstone, Siobhain Carmody, Xiaobei Zhao, Valerie Page, Mark Louie Guanco, Elvira Hoxha, Camilla Zorloni, Charlotte Dean, Emma Jones, Emma Carter, Joshua Dunn, Thomas Kong, Mervin Mahenthran, Chris Marsh, Maureen Holland, Natalie Keenan, Mohamed Mahmoud, Marc Lyons, Joanne Bradley-Potts, Helen Wassall, Meghan Young, Paul Bradley, Dorota Burda, Sinead Donlon, Lesley Harden, Celia Harris, Irving Mayangao, Rugia Montaser, Sheila Mtuwa, Charles Piercy, Eleanor Smith, Sarah Stone, Jerik Verula, Helen Blackman, Cheryl Marriott, Natalia Michalak, Ben Creagh-Brown, Armorel Salberg, Naomi Boyer, Veronika Pristopan, Victoria Maynard, Rachel Walker, Anil Hormis, Dawn Collier, Cheryl Graham, Vicky Maynard, Jake McCormick, Jake Warrington, Denise Cosgrove, Denise McFarland, Judith Ratcliffe, Rob Charnock, Inez Wynter, Mandy Gill, Jill Kirk, Paul Paul, Valli Ratnam, Sarah Shelton, Catherine Jardine, Alasdair Hay, Dewi Williams, Bethan Deacon, Latha Durga, Meg Hibbert, Gareth Kennard-Holden, Chrsitopher Woodford, Carla Pothecary, Lisa Roche, Dariusz Tetla, Kevin Agravante, Justyna Smeaton, Alicia Price, Alice Thomas, Chris Thorpe, Ellen Knights, Donna Ward, Shondipon Laha, Mark Verlander, Alexandra Williams, Rachel Prout, Helen Langton, Malcolm Watters, Charlotte Hunt, Catherine Novis, Sarwat Arif, Amy Cunningham, Claire Hewitt, Julia Hindale, Karen Jackson-Lawrence, Sarah Shepardson, Maryanne Wills, Susie Butler, Silivia Tavares, Russell Barber, Annette Hilldrith, Kelly Hubbard, Dawn Egginton, Michele Clark, Sarah Purvis, Simon Sinclair, Vicky Collins, Bethan Landeg, Craig Sell, Samantha Coetzee, Alistair Gales, Igor Otahal, Becky Icke, Meena Raj, Caroline Williams, Jill Williams, Lucy Hill, Abdul Kayani, Bridgett Masunda, Prisca Gondo, Nigara Atayeva, Carina Cruz, Natalie Pattison, Caroline Burnett, Jonathan Hatton, Elaine Heeney, Maria Newton, Hassan Al-Moasseb, Teresa Behan, Jasmine Player, Rachael Stead, Atideb Mitra, Kirsty Nauyokas, Sally Humphreys, Helen Cockerill, Ruth Tampsett, Evgeniya Postovalova, Tina Coventry, Amanda McGregor, Susan Fowler, Mike Macmahon, Patricia Cochrane, Sandra Pirie, Sarah Hanley, Asifa Ali, Megan Brady, Sam Dale, Annalisa Dance, Lisa Gledhill, Jill Greig, Kathryn Hanson, Kelly Holdroyd, Marie Home, Tahira Ishaq, Diane Kelly, Lear Matapure, Deborah Melia, Samantha Mellor, Ekta Merwaha, Tonicha Nortcliffe, Lisa Shaw, Ryan Shaw, Tracy Wood, Lee-Ann Bayo, Miranda Usher, Alison Wilson, Ross Kitson, Jez Pinnell, Matthew Robinson, Kaitlin Boltwood, Jenny Birch, Laura Bough, Rebecca Tutton, Barbara Winter-Goodwin, Josie Goodsell, Kate Taylor, Patricia Williams, Sarah Williams, Ashleigh Cave, James Rees, Janet Imeson-Wood, Jacqueline Smith, Vishal Amin, Komala Karthik, Rizwana Kausar, Elena Anastasescu, Karen Reid, Vikram Anumakonda, Ella Stoddart, Carrie Demetriou, Charlotte Eckbad, Lucy Howie, Sarah Mitchard, Lidia Ramos, Katie White, Sarah Hierons, Fiona Kelly, Alfredo Serrano-Ruiz, Gabrielle Evans, Liz Nicol, Joy Wilkins, Kim Hulacka, Gabor Debreceni, Alison Brown, Vikki Crickmore, Kay Hill, Thogulava Kannan, Zenaida Dagutao, Kate Beesley, Alison Lewis, Jess Perry, Sherly Antony, Sarah Board, Clare Buckley, Lucy Pippard, Alfonso Tanate, Diane Wood, Agnieska Kubisz-Pudelko, Ayman Gouda, Fiona Auld, Joanne Donnachie, Euan Murdoch, Lynn Prentice, Nikole Runciman, Dhaneesha Senaratne, Abigail Short, Laura Sweeney, Lesley Symon, Anne Todd, Patricia Turner, Erin McCann, Dario Salutous, Ian Edmond, Lesley Whitelaw, Harish Venkatesh, Yvonne Bland, Istvan Kajtor, Lisa Kavanagh, Karen Singler, George Linfield-Brown, Luke Stephen Prockter Moore, Marcela Vizcaychipi, Laura Martins, Luke Moore, Rhian Bull, Jaime Carungcong, Louise Allen, Eva Beranova, Alicia Knight, Carly Price, Sorrell Tilbey, Sharon Turney, Tracy Hazelton, Gabriella Tutt, Mansi Arora, Salah Turki, Emily Sinfield, Joanne Deery, Hazel Ramos, Daniele Cristiano, Natalie Dormand, Zohreh Farzad, Mahitha Gummadi, Sara Salmi, Geraldine Sloane, Mathew Varghese, Vicky Thwaites, Brijesh Patel, Liyanage Kamal, Anelise Catelan Zborowski, Ryan Coe, Madeleine Anderson, Jane Beadle, Charlotte Coates, Katy Collins, Maria Crowley, Laura Johnson, Laura King, Remi Paramsothy, Janet Sargeant, Pedro Silva, Carmel Stuart, June Taylor, David Tyl, Phillipa Wakefield, Charlotte Kamundi, Olumide Olufuwa, Zakaulla Belagodu, Anca Gherman, Naomi Oakley, John Allan, Tim Geary, Alistair Meikle, Peter O’Brien, Stephen Wood, Andrew Clark, Gordon Houston, Karen Black, Michelle Clarkson, Stuart D’Sylva, Alan Morrison, Kathryn Norman, Margaret Taylor, Suzanne Clements, Catriona Cohrane, Nora Gonzalez, Dominic Strachan, Claire Beith, Kirsten Moar, Lorna Murphy, Michelle Smythe, Alistair Nichol, Kathy Brickell, Inthakab Ali Mohamed Ali, Karen Beaumont, Mohamed Elsaadany, Kay Fernandes, Sameena Mohamed Ally, Harini Rangarajan, Varun Sarathy, Sivarupan Selvanayagam, Dave Vedage, Matthew White, Zoe Coton, Aricsa Joshy, Mark Blunt, Hollie Curgenven, Liam Botfield, Catherine Dexter, Aditya Kuravi, Joanne Butler, Robert Chadwick, Poonam Ranga, Lisa Richardson, Emma Virgilio, Maddiha Anwer, Atul Garg, Donna Botfield, Xana Marriott, Keely Stewart, Dee Mullan, Claire Phillips, Jane Gaylard, Justyna Nowak, Denise Skinner, Sian Jones, Rikki Crawley, Abigail Crew, Mishell Cunningham, Allison Daniels, Laura Harrison, Susan Hope, Nicola Lancaster, Jamie Matthews, Gemma Wray, Alice Nicholson, Ken Inweregbu, Sarah Cutts, Katharine Miller, Ailbhe Brady, Rebekah Chan, Shane McIvor, Helena Prady, Bijoy Mathew, Jeff Little, Tim Furniss, Chris Wright, Bernadette King, Christopher Wasson, Aisling O’Neill, Christine Turley, Peter McGuigan, Erin Collins, Stephanie Finn, Jackie Green, Julie McAuley, Abitha Nair, Charlotte Quinn, Suzanne Tauro, Kathryn Ward, Michael McGinlay, Kiran Reddy, Norfaizan Ahmad, Samantha Anderson, Joann Barker, Kris Bauchmuller, Kathryn Birchall, Sarah Bird, Kay Cawthron, Luke Chetam, Joby Cole, Ben Donne, David Foote, Amber Ford, Helena Hanratty, Kate Harrington, Lisa Hesseldon, Kay Housley, Yvonne Jackson, Claire Jarman, Faith Kibutu, Becky Lenagh, Irene Macharia, Shamiso Masuko, Leanne Milner, Helen Newell, Lorenza Nwafor, Simon Oxspring, Patrick Phillips, Ajay Raithatha, Sarah Rowland-Jones, Jacqui Smith, Roger Thompson, Helen Trower, Sara Walker, James Watson, Matthew Wiles, Alison Lye, Jayne Willson, Gary Mills, Sansha Harris, Eleanor Hartill, Anthony Barron, Ciara Collins, Sundeep Kaul, Claire Nolan, Oliver Polgar, Claire Prendergast, Paula Rogers, Rajvinder Shokkar, Meriel Woodruff, Kanta Mahay, Vicky Thwaites, Anna Reed, Hayley Meyrick, Heather Passmore, James Farwell, Alison Brown, Susan O’Connell, Jane Gregory, Luigi Barberis, Rosemary Harper, Tim Smith, Diane Armstrong, Angie Bowey, Anne Cowley, Andrew Corner, Judith Highgate, Claire Rutherfurd, Jo-Anne Taylor, Sarah Goodwin, Claire Rutherford, Beena Eapen, Fiona Trim, Phil Donnison, Lisa Armstrong, Hayley Bates, Emma Dooks, Fiona Farquhar, Amy Kitching, Chantal McParland, Sophie Packham, Brigid Hairsine, Anand Patil, Premetie Andreou, Dawn Hales, Megha Mathews, Rekha Patel, Peter Barry, Neil Flint, Jessica Hailstone, Navneet Ghuman, Bethany Leonard, Rachel Lees, Deborah Butcher, Katy Leng, Nicola Butterworth-Cowin, Susie O’Sullivan, Alison Ghosh, Emma Williams, Colene Adams, Anita Agasou, Tracie Arden, Mandy Beekes, Amy Bowes, Pauline Boyle, Heather Button, Mandy Carnahan, Anne Carter, Danielle Childs, Jane Gaylard, Fran Hurford, Yasmin Hussain, Ayesha Javaid, James Jones, Michael Leigh, Terry Martin, Helen Millward, Nichola Motherwell, Dee Mullan, Julie Newman, Rachel Rikunenko, Jo Stickley, Julie Summers, Louise Ting, Helen Tivenan, Denise Donaldson, Nigel Capps, Emily Cale, Sanal Jose, Wendy Osbourne, Susie Pajak, Jayne Rankin, Louise Tonks, Tracy Baird, Margaret Harkins, Jim Ruddy, Joe West, Joseph Duffield, Lewis Mallon, Oliver Smith, Sara Smuts, Andy Campbell, Cate Davies, Sarah Davies, Rachel Hughes, Lisa Jobes, Victoria Whitehead, Clare Watkins, Fiona Bowman, Barry Milligan, Liane McPherson, Stella Metherell, Nichola Harris, Victoria Lake, Elizabeth Radford, Andy Smallwood, Shameer Gopal, Katherine Vassell, Dina Bell, Rosalind Boyle, Katie Douglas, Lynn Glass, Liz Lennon, Austin Rattray, Claire Beith, Emma Lee, Danielle Jones, Penny Parsons, Ben Attwood, Paul Jefferson, Mohan Ranganathan, Inderjit Atwal, Bridget Campbell, Angela Day, Camilla Stagg, Emma Haynes, Cecilia Ahmed, Sarah Clamp, Julie Colley, Risna Haq, Anne Hayes, Sibet Joseph, Zahira Maqsood, Samia Hussain, Jonathan Hulme, Patience Domingos, Rita Kumar, Manjit Purewal, Becky Taylor, Lara Bunni, Monica Latif, Claire Jennings, Shilu Jose, Rebecca Marshall, Aleksandra Metryka, Gayathri Subramanian, Adam Burgoyne, Susan O’Connell, Amanda Tyler, Joanne Waldron, Paula Hilltout, Jayne Evitts, Geraldine Ward, Pamela Bremmer, Carl Hawkins, Sophie Jackman, Michal Ogorek, Kylie Ashby, Lorraine Thornton, Pauline Mercer, Matthew Halkes, Adam Revill, Bryony Saint, Jo Fletcher, Kimberley Netherton, Manish Chablani, Amy Kirkby, Amanda Roper, Kinga Szymiczek, Isobel Sutherland, Linda O’Brien, Igor Otahal, Joanne Connell, Kim Davies, Tracy Lewis, Zohra Omar, Emma Perkins, Lisa Roche, Sonia Sathe, Ellie Davies, Alex Lyon, Isheunesu Mapfunde, Charlotte Willis, Rachael Hitchcock, Kathryn Hall, Christopher King, Andrew Fagan, Roonak Nazari, Lucy Worsley, Suzanne Allibone, Vidya Kasipandian, Amit Patel, Parisa Cutting, Roman Genetu, Ainhi Mac, Anthony Murphy, Sinead Ward, Fatima Butt, Amanda Ayers, Wendy Harrison, Katherine Mackintosh, Julie North, Lydia Ashton, Rehana Bi, Samantha Owen, Helen Winmill, Barney Scholefield, Hannah Blowing, Erin Williams, Michaela Duskova, Michelle Edwards, Alun Rees, Helen Thomas, Rachel Hughes, Igor Otahal, Jolene Brooks, Janet Phipps, Suzanne Brooks, Catherine Dennis, Vicki Parris, Sinduya Srikaran, Anisha Sukha, Alistair McGregor, Gerlynn Tiongson, Katie Adams, Benedict Andrew, Adam Brayne, Sasha Carter, Louise Findlay, Emma Fisher, Peter Jackson, Duncan Kaye, Juliet Parkin, Victoria Tuckey, Jane Hunt, Nicholas Love, Lynne van Koutrick, Ashley Hanson, Kathy Dent, Elizabeth Horsley, Sandra Pearson, Sue Spencer, Dorothy Hutchinson, Jasmine Player, Dorota Potoczna, Muhammad Nauman Akhtar, Lisa-Jayne Cottam, Kirsty Nauyokas, Jack Sanders, Sara Mingo Garcia, Glykeria Pakou, Cynthia Diaba, Helder Filipe, Lincy John, Amitaa Maharajh, Mark de Neef, Daniel Martin, Christine Eastgate, Poh Choo Teoh, Fiona Barrett, Clare Bradley, Avril Donaldson, Mairi Mascarenhas, Marianne O’Hara, Laura Okeefe, Noreen Clarke, Jonathan Whiteside, Rachael Campbell, Joanna Matheson, Deborah McDonald, Donna Patience, Polly Rice, Tim Smith, Melanie Clapham, Rachel Mutch, Luigi Barberis, Rosemary Harper, Hannah Craig, Una Poultney, Karen Burns, Andrew Higham, Sophie Twiss, Janet Barton, Linsha George, Clare Harrop, Sherly Mathew, David Justin Wright, Rachel Harrison, Jordan Toohie, Ben Chandler, Alison Turnbull, Janine Mallinson, Kerry Elliott, Rebecca Wolf-Roberts, Helen Tench, Igor Otahal, Maria Hobrok, Ronda Loosley, Heather McGuinness, Tanya Sims, Deborah Afolabi, Kathryn Sian Allison, Taya Anderson, Rachael Dore, Dawn Jones, Naomi Rogers, Paula Saunderson, Jennifer Whitbread, Laura O’Malley, Laura Rad, Daniel Hawcutt, Jonathan Aldridge, Melanie Tolson, Sweyn Garrioch, Joanne Tomlinson, Michael Grosdenier, David Loader, Ritoo Kapoor, Gemma Hector, Joslan Scherewode, Chunda Sri-Chandana, Lorraine Stephenson, Sarah Marsh, Arnold Dela Rosa, Shaman Jhanji, Thomas Bemand, Ryan Howle, Ravishankar Rao Baikady, Benjamin Thomas, Ethel Black, Kate Tatham, Sambasivarao Gurram, Ekaterina Watson, Vicki Parris, Sheena Quaid, Alistair McGregor, Anne Saunderson, Rachel O’Brien, Sam Moultrie, Jen Service, Clare Cheyne, Miranda Odam, Alison Wiliams, Nicky Barnes, Peter Csabi, Joana Da Rocha, Louika Glynou, Amy Huffenberger, Jade Bryant, Amy Pickard, Nicholas Roe, Arianna Bellini, Anton Mayer, Amy Burrow, Natalie Colley, Jayne Evans, Alex Howlett, Zeinab Khalifeh, Jerldine Pryce, Claire Gorman, Amy Easthope, Rebecca Brady, Elizabeth Timlick, Pierre Antoine, Abhinhav Gupta, John Hardy, Henry Houlden, Eleanor Moncur, Arianna Tucci, Eamon Raith, Ambreen Tariq, David Brealey, Emma Tagliavini, Becky Ramsay, Katy Fidler, Kevin Donnelly, Rebecca Hollis, Jocelyn Barr, Elizabeth Boyd, Val Irvine, Ben Shelley, Julie Buckley, Charlene Hamilton, Kathryn Valdeavella, SCOURGE Consortium, Javier Abellan, René Acosta-Isaac, Jose María Aguado, Carlos Aguilar, Sergio Aguilera-Albesa, Abdolah Ahmadi Sabbagh, Jorge Alba, Sergiu Albu, Karla A. M. Alcalá-Gallardo, Julia Alcoba-Florez, Sergio Alcolea Batres, Holmes Rafael Algarin-Lara, Virginia Almadana, Julia Almeida, Berta Almoguera, María R. Alonso, Nuria Alvarez, Yady Álvarez-Benítez, Felipe Álvarez-Navia, Rodolfo Alvarez-Sala Walther, Álvaro Andreu-Bernabeu, Maria Rosa Antonijoan, Eunate Arana-Arri, Carlos Aranda, Celso Arango, Carolina Araque, Nathalia K. Araujo, Izabel M. T. Araujo, Ana C. Arcanjo, Ana Arnaiz, Francisco Arnalich Fernández, María J. Arranz, José Ramon Arribas Lopez, Maria-Jesus Artiga, Yubelly Avello-Malaver, Carmen Ayuso, Ana Margarita Baldión-Elorza, Belén Ballina Martín, Raúl C. Baptista-Rosas, Andrea Barranco-Díaz, María Barreda-Sánchez, Viviana Barrera-Penagos, Moncef Belhassen-Garcia, Enrique Bernal, David Bernal-Bello, Joao F. Bezerra, Marcos A. C. Bezerra, Natalia Blanca-López, Rafael Blancas, Lucía Boix-Palop, Alberto Borobia, Elsa Bravo, María Brion, Óscar Brochado-Kith, Ramón Brugada, Matilde Bustos, Alfonso Cabello, Juan J. Caceres-Agra, Esther Calbo, Enrique J. Calderón, Shirley Camacho, Marcela C. Campos, Yolanda Cañadas, Cristina Carbonell, Servando Cardona-Huerta, Antonio Augusto F. Carioca, Maria Sanchez Carpintero, Carlos Carpio Segura, Thássia M. T. Carratto, José Antonio Carrillo-Avila, Maria C. C. Carvalho, Carlos Casasnovas, Luis Castano, Carlos F. Castaño, Jose E. Castelao, Aranzazu Castellano Candalija, María A. Castillo, Francisco C. Ceballos, Jessica G. Chaux, Walter G. Chaves-Santiago, Sylena Chiquillo-Gómez, Marco A. Cid-Lopez, Oscar Cienfuegos-Jimenez, Rosa Conde-Vicente, M. Lourdes Cordero-Lorenzana, Dolores Corella, Almudena Corrales, Jose L. Cortes-Sanchez, Marta Corton, Tatiana X. Costa, Raquel Cruz, Marina S. Cruz, Luisa Cuesta, Gabriela C. R. Cunha, Gabriela V. da Silva, David Dalmau, Raquel C. S. Dantas-Komatsu, M. Teresa Darnaude, Raimundo de Andrés, Jéssica N. G. de Araújo, Carmen de Juan, Juan De la Cruz Troca, Carmen de la Horra, Ana B. de la Hoz, Alba De Martino-Rodríguez, Julianna Lys de Sousa Alves Neri, Victor del Campo-Pérez, Juan Delgado-Cuesta, Covadonga M. Diaz-Caneja, Anderson Díaz-Pérez, Aranzazu Diaz de Bustamante, Beatriz Dietl, Silvia Diz-de Almeida, Manoella do Monte Alves, Elena Domínguez-Garrido, Katiusse A. dos Santos, Alice M. Duarte, Jose Echave-Sustaeta, Rocío Eiros, César O. Enciso-Olivera, Gabriela Escudero, Pedro Pablo España, Gladys Mercedes Estigarribia Sanabria, María Carmen Fariñas, Marianne R. Fernandes, Ramón Fernández, Lidia Fernandez-Caballero, Ana Fernández-Cruz, María J. Fernandez-Nestosa, Uxía Fernández-Robelo, Amanda Fernández-Rodríguez, Marta Fernández-Sampedro, Ruth Fernández-Sánchez, Tania Fernández-Villa, Silvia Fernández Ferrero, Yolanda Fernández Martínez, Carmen Fernéndez Capitán, Patricia Flores-Pérez, Vicente Friaza, Lácides Fuenmayor-Hernández, Marta Fuertes Núñez, Victoria Fumadó, Ignacio Gadea, Lidia Gagliardi, Manuela Gago-Domínguez, Natalia Gallego, Cristina Galoppo, Inés García, Mercedes García, Leticia García, Carlos Garcia-Cerrada, Aitor García-de-Vicuña, Josefina Garcia-García, Irene García-García, Carmen García-Ibarbia, Andrés C. García-Montero, Ana García-Soidán, Elisa García-Vázquez, María Carmen García Torrejón, Emiliano Garza-Frias, Angela Gentile, Belén Gil-Fournier, Javier Gómez-Arrue, Mario Gómez-Duque, Luis Gómez Carrera, María Gómez García, Ángela Gómez Sacristán, Anna González-Neira, Javier González-Peñas, Manuel Gonzalez-Sagrado, Beatriz González Álvarez, Fernan Gonzalez Bernaldo de Quirós, Hugo Gonzalo Benito, Oscar Gorgojo-Galindo, Miguel Górgolas, Florencia Guaragna, Genilson P. Guegel, Beatriz Guillen-Guio, Encarna Guillen-Navarro, Pablo Guisado-Vasco, Juan F. Gutiérrez-Bautista, Luz D. Gutierrez-Castañeda, Sarah Heili-Frades, Estefania Hernandez, Luis D. Hernandez-Ortega, Guillermo Hernández-Pérez, Rebeca Hernández-Vaquero, Cristina Hernández Moro, Belen Herraez, M. Teresa Herranz, María Herrera, María José Herrero, Antonio Herrero-Gonzalez, Juan P. Horcajada, Natale Imaz-Ayo, Maider Intxausti-Urrutibeaskoa, María Íñiguez, Rafael H. Jacomo, Rubén Jara, Perez Maria Jazmin, Ángel Jiménez, Pilar Jiménez, Ignacio Jiménez-Alfaro, María A. Jimenez-Sousa, Iolanda Jordan, Rocío Laguna-Goya, Daniel Laorden, María Lasa-Lazaro, María Claudia Lattig, Ailen Lauriente, Anabel Liger Borja, Lucía Llanos, Amparo López-Bernús, Esther Lopez-Garcia, Rosario Lopez-Rodriguez, Miguel A. López-Ruz, Eduardo López Granados, Leonardo Lorente, José E. Lozano, María Lozano-Espinosa, Andre D. Luchessi, Ignacio Mahillo, Esther Mancebo, Carmen Mar, Cristina Marcelo Calvo, Miguel Marcos, Alba Marcos-Delgado, Alicia Marín Candon, Pablo Mariscal Aguilar, María M. Martín, María Dolores Martín, Vicente Martín, Marta Martin-Fernandez, Caridad Martín-López, José-Ángel Martín-Oterino, Laura Martin-Pedraza, María Martín-Vicente, Amalia Martinez, Ricardo Martínez, Juan José Martínez, Silvia Martínez, Eleno Martínez-Aquino, Óscar Martínez-González, Iciar Martinez-Lopez, Oscar Martinez-Nieto, Pedro Martinez-Paz, Angel Martinez-Perez, Andrea Martínez-Ramas, Michel F. Martinez-Resendez, Violeta Martínez Robles, Laura Marzal, Juliana F. Mazzeu, Jeane F. P. Medeiros, Kelliane A. Medeiros, Francisco J. Medrano, Xose M. Meijome, Natalia Mejuto-Montero, Ana Méndez-Echevarria, Humberto Mendoza Charris, Eleuterio Merayo Macías, Fátima Mercadillo, Arieh R. Mercado-Sesma, Pablo Minguez, Antonio J. J. Molina, Elena Molina-Roldán, Juan José Montoya, Vitor M. S. Moraes, Patricia Moreira-Escriche, Xenia Morelos-Arnedo, Antonio Moreno-Docón, Junior Moreno-Escalante, Victor Moreno Cuerda, Alberto Moreno Fernández, Rubén Morilla, Patricia Muñoz García, Pablo Neira, Julian Nevado, Israel Nieto-Gañán, Joana F. R. Nunes, Rocio Nuñez-Torres, Antònia Obrador-Hevia, J. Gonzalo Ocejo-Vinyals, Virginia Olivar, Silviene F. Oliveira, Lorena Ondo, Alberto Orfao, Luis Ortega, Eva Ortega-Paino, Fernando Ortiz-Flores, Rocio Ortiz-Lopez, José A. Oteo, Harry Pachajoa, Manuel Pacheco, Fredy Javier Pacheco-Miranda, Irene Padilla Conejo, Sonia Panadero-Fajardo, Mara Parellada, Roberto Pariente-Rodríguez, Estela Paz-Artal, Germán Peces-Barba, Miguel S. Pedromingo Kus, Celia Perales, Patricia Perez, César Pérez, Gustavo Perez-de-Nanclares, Felipe Pérez-García, Patricia Pérez-Matute, Alexandra Pérez-Serra, M. Elena Pérez-Tomás, Teresa Perucho, Lisbeth A. Pichardo, Susana M. T. Pinho, Mel·lina Pinsach-Abuin, Luz Adriana Pinzón, Guillermo Pita, Francesc Pla-Junca, Laura Planas-Serra, Ericka N. Pompa-Mera, Gloria L. Porras-Hurtado, Aurora Pujol, María Eugenia Quevedo Chávez, Maria Angeles Quijada, Inés Quintela, Diana Ramirez-Montaño, Soraya Ramiro León, Pedro Rascado Sedes, Delia Recalde, Emma Recio-Fernández, Salvador Resino, Adriana P. Ribeiro, Carlos S. Rivadeneira-Chamorro, Diana Roa-Agudelo, Montserrat Robelo Pardo, Marilyn Johanna Rodriguez, Fernando Rodriguez-Artalejo, Marena Rodríguez-Ferrer, Carlos Rodriguez-Gallego, José A. Rodriguez-Garcia, María A. Rodriguez-Hernandez, Antonio Rodriguez-Nicolas, Agustí Rodriguez-Palmero, Emilio Rodríguez-Ruiz, Paula A. Rodriguez-Urrego, Belén Rodríguez Maya, German Ezequiel Rodriguez Novoa, Federico Rojo, Andrea Romero-Coronado, Filomeno Rondón García, Lidia S. Rosa, Antonio Rosales-Castillo, Cladelis Rubio, María Rubio Olivera, Montserrat Ruiz, Francisco Ruiz-Cabello, Eva Ruiz-Casares, Juan J. Ruiz-Cubillan, Javier Ruiz-Hornillos, Pablo Ryan, Hector D. Salamanca, Lorena Salazar-García, Giorgina Gabriela Salgueiro Origlia, Pedro-Luis Sánchez, Clara Sánchez-Pablo, Olga Sánchez-Pernaute, Antonio J. Sánchez López, María Concepción Sánchez Prados, Javier Sánchez Real, Jorge Sánchez Redondo, Cristina Sancho-Sainz, Anna Sangil, Arnoldo Santos, Ney P. C. Santos, Agatha Schlüter, Sonia Segovia, Alex Serra-Llovich, Fernando Sevil Puras, Marta Sevilla Porras, Miguel A. Sicolo, Vivian N. Silbiger, Nayara S. Silva, Fabiola T. C. Silva, Cristina Silván Fuentes, Jordi Solé-Violán, José Manuel Soria, Jose V. Sorlí, Renata R. Sousa, Juan Carlos Souto, Karla S. C. Souza, Vanessa S. Souza, John J. Sprockel, José Javier Suárez-Rama, David A. Suarez-Zamora, Xiana Taboada-Fraga, Eduardo Tamayo, Alvaro Tamayo-Velasco, Juan Carlos Taracido-Fernandez, Nathali A. C. Tavares, Carlos Tellería, Jair Antonio Tenorio Castaño, Alejandro Teper, Juan Torres-Macho, Lilian Torres-Tobar, Ronald P. Torres Gutiérrez, Jesús Troya, Miguel Urioste, Juan Valencia-Ramos, Agustín Valido, Juan Pablo Vargas Gallo, Belén Varón, Romero H. T. Vasconcelos, Tomas Vega, Santiago Velasco-Quirce, Valentina Vélez-Santamaría, Virginia Víctor, Julia Vidán Estévez, Miriam Vieitez-Santiago, Carlos Vilches, Lavinia Villalobos, Felipe Villar, Judit Villar-Garcia, Cristina Villaverde, Pablo Villoslada-Blanco, Ana Virseda-Berdices, Zuleima Yáñez, Antonio Zapatero-Gaviria, Ruth Zarate, Sandra Zazo, Miguel López de Heredia, Ingrid Mendes, Rocío Moreno, Esther Sande, Pablo Lapunzina, Angel Carracedo, ISARICC Investigators, Beatrice Alex, Petros Andrikopoulos, Benjamin Bach, Wendy S. Barclay, Debby Bogaert, Meera Chand, Kanta Chechi, Graham S. Cooke, Ana da Silva Filipe, Thushan de Silva, Annemarie B. Docherty, Gonçalo dos Santos Correia, Marc-Emmanuel Dumas, Jake Dunning, Tom Fletcher, Christopher A. Green, William Greenhalf, Julian Griffin, Rishi K. Gupta, Ewen M. Harrison, Antonia Y. W. Ho, Karl Holden, Peter W. Horby, Samreen Ijaz, Say Khoo, Paul Klenerman, Andrew Law, Matthew Lewis, Sonia Liggi, Wei Shen Lim, Lynn Maslen, Alexander J. Mentzer, Laura Merson, Alison M. Meynert, Shona C. Moore, Mahdad Noursadeghi, Michael Olanipekun, Anthonia Osagie, Massimo Palmarini, Carlo Palmieri, William A. Paxton, Georgios Pollakis, Nicholas Price, Andrew Rambaut, David L. Robertson, Clark D. Russell, Vanessa Sancho-Shimizu, Caroline Sands, Janet T. Scott, Louise Sigfrid, Tom Solomon, Shiranee Sriskandan, David Stuart, Olivia V. Swann, Zoltan Takats, Panteleimon Takis, Richard S. Tedder, A. A. Roger Thompson, Emma C. Thomson, Ryan S. Thwaites, Lance C. W. Turtle, Maria Zambon, Gail Carson, Thomas M. Drake, Cameron J. Fairfield, Stephen R. Knight, Kenneth A. Mclean, Derek Murphy, Lisa Norman, Riinu Pius, Catherine A. Shaw, Marie Connor, Jo Dalton, Carrol Gamble, Michelle Girvan, Sophie Halpin, Janet Harrison, Clare Jackson, Laura Marsh, Stephanie Roberts, Egle Saviciute, Susan Knight, Eva Lahnsteiner, Gary Leeming, Lucy Norris, James Scott-Brown, Sarah Tait, Murray Wham, James Lee, Daniel Plotkin, Seán Keating, Cara Donegan, Rebecca G. Spencer, Chloe Donohue, Hayley Hardwick, The 23andMe COVID-19 Team, Janie F. Shelton, Anjali J. Shastri, Chelsea Ye, Catherine H. Weldon, Teresa Filshtein-Sonmez, Daniella Coker, Antony Symons, Jorge Esparza-Gordillo, Stella Aslibekyan, Adam Auton, Jian Yang, Chris P. Ponting, James F. Wilson, Veronique Vitart, Malak Abedalthagafi, Andre D. Luchessi, Esteban J. Parra, Raquel Cruz, Angel Carracedo, Angie Fawkes, Lee Murphy, Kathy Rowan, Alexandre C. Pereira, Andy Law, Benjamin Fairfax, Sara Clohisey Hendry, J. Kenneth Baillie

**Affiliations:** 1 Baillie Gifford Pandemic Science Hub, Centre for Inflammation Research, The Queen’s Medical Research Institute, University of Edinburgh, Edinburgh, UK; 2 MRC Human Genetics Unit, Institute of Genetics and Cancer, University of Edinburgh, Western General Hospital, Edinburgh, UK; 3 Roslin Institute, University of Edinburgh, Edinburgh, UK; 4 Pain Service, NHS Tayside, Ninewells Hospital and Medical School, Dundee, UK; 5 School of Life Sciences, Westlake University, Hangzhou, China; 6 Westlake Laboratory of Life Sciences and Biomedicine, Hangzhou, China; 7 Institute for Molecular Bioscience, The University of Queensland, Brisbane, Queensland Australia; 8 Wellcome Centre for Human Genetics, University of Oxford, Oxford, UK; 9 Faculty of Biological Sciences, University of Leeds, Leeds, UK; 10 Genomics England, London, UK; 11 Intensive Care Unit, Royal Infirmary of Edinburgh, Edinburgh, UK; 12 National Heart and Lung Institute, Imperial College London, London, UK; 13 Edinburgh Clinical Research Facility, Western General Hospital, University of Edinburgh, Edinburgh, UK; 14 Department of Critical Care Medicine, Queen’s University and Kingston Health Sciences Centre, Kingston, Ontario Canada; 15 Clinical Research Centre at St Vincent’s University Hospital, University College Dublin, Dublin, Ireland; 16 NIHR Health Protection Research Unit for Emerging and Zoonotic Infections, Institute of Infection, Veterinary and Ecological Sciences University of Liverpool, Liverpool, UK; 17 Respiratory Medicine, Alder Hey Children’s Hospital, Institute in The Park, University of Liverpool, Alder Hey Children’s Hospital, Liverpool, UK; 18 Centre for Inflammation Research, The Queen’s Medical Research Institute, University of Edinburgh, Edinburgh, UK; 19 Department of Medicine, University of Cambridge, Cambridge, UK; 20 William Harvey Research Institute Barts and the London School of Medicine and Dentistry, Queen Mary University of London, London, UK; 21 Centre for Tropical Medicine and Global Health, Nuffield Department of Medicine, University of Oxford, Oxford, UK; 22 Department of Anaesthesia and Intensive Care, The Chinese University of Hong Kong, Prince of Wales Hospital, Hong Kong, China; 23 Wellcome-Wolfson Institute for Experimental Medicine, Queen’s University Belfast, Belfast, UK; 24 Department of Intensive Care Medicine, Royal Victoria Hospital, Belfast, UK; 25 UCL Centre for Human Health and Performance, London, UK; 26 Imperial College Healthcare NHS Trust, London, UK; 27 Royal Hospital for Children, Glasgow, UK; 28 Centre for Global Health Research, Usher Institute of Population Health Sciences and Informatics, Edinburgh, UK; 29 Genomics Division, Instituto Tecnológico y de Energías Renovables, Santa Cruz de Tenerife, Spain; 30 Research Unit, Hospital Universitario N.S. de Candelaria, Santa Cruz de Tenerife, Spain; 31 Centre for Biomedical Network Research on Respiratory Diseases (CIBERES), Instituto de Salud Carlos III, Madrid, Spain; 32 Department of Clinical Sciences, University Fernando Pessoa Canarias, Las Palmas de Gran Canaria, Spain; 33 IDIVAL, Santander, Spain; 34 Universidad de Cantabria, Santander, Spain; 35 Hospital U M Valdecilla, Santander, Spain; 36 Tecnologico de Monterrey, Escuela de Medicina y Ciencias de la Salud and Hospital San Jose TecSalud, Monterrey, Mexico; 37 Centre for Biomedical Network Research on Rare Diseases (CIBERER), Instituto de Salud Carlos III, Madrid, Spain; 38 Instituto de Genética Médica y Molecular (INGEMM), Hospital Universitario La Paz-IDIPAZ, Madrid, Spain; 39 ERN-ITHACA-European Reference Network, Paris, France; 40 Genomic Research Department, King Fahad Medical City, Riyadh, Saudi Arabia; 41 Department of Pathology & Laboratory Medicine, Emory University Hospital, Atlanta, GA USA; 42 Department of Clinical Analysis and Toxicology, Federal University of Rio Grande do Norte, Natal, Brazil; 43 Department of Anthropology, University of Toronto at Mississauga, Mississauga, Ontario Canada; 44 Centro Singular de Investigación en Medicina Molecular y Enfermedades Crónicas (CIMUS), Universidade de Santiago de Compostela, Santiago de Compostela, Spain; 45 Instituto de Investigación Sanitaria de Santiago (IDIS), Santiago de Compostela, Spain; 46 Fundación Pública Galega de Medicina Xenómica, Sistema Galego de Saúde (SERGAS) Santiago de Compostela, Santiago de Compostela, Spain; 47 Intensive Care National Audit & Research Centre, London, UK; 48 Heart Institute, University of Sao Paulo, Butanta, Brazil; 49 NIHR Clinical Research Network (CRN), North West London Core Team, Hammersmith Hospital, London, UK; 50 Cambridge University Hospitals NHS Foundation Trust, Cambridge, UK; 51 Biostatistics Group, State Key Laboratory of Biocontrol, School of Life Sciences, Sun Yat-sen University, Guangzhou, China; 52 Department of Infectious Diseases, Leiden University Medical Center, Leiden, The Netherlands; 53 Guys and St Thomas’ Hospital, London, UK; 54 James Cook University Hospital, Middlesbrough, UK; 55 Barts Health NHS Trust, London, UK; 56 Royal Stoke University Hospital, Stoke-on-Trent, UK; 57 North Middlesex University Hospital NHS Trust, London, UK; 58 King’s College Hospital, London, UK; 59 Charing Cross Hospital, St Mary’s Hospital and Hammersmith Hospital, London, UK; 60 The Royal Liverpool University Hospital, Liverpool, UK; 61 John Radcliffe Hospital, Oxford, UK; 62 Addenbrooke’s Hospital, Cambridge, UK; 63 Nottingham University Hospital, Nottingham, UK; 64 St George’s Hospital, London, UK; 65 BHRUT (Barking Havering)—Queens Hospital and King George Hospital, Ilford, UK; 66 Royal Infirmary of Edinburgh, Edinburgh, UK; 67 Kingston Hospital, London, UK; 68 Queen Alexandra Hospital, Portsmouth, UK; 69 Royal Gwent Hospital, Newport, UK; 70 Royal Blackburn Teaching Hospital, Blackburn, UK; 71 Stepping Hill Hospital, Stockport, UK; 72 Northumbria Healthcare NHS Foundation Trust, North Shields, UK; 73 Countess of Chester Hospital, Chester, UK; 74 Pinderfields General Hospital, Wakefield, UK; 75 Ninewells Hospital, Dundee, UK; 76 Croydon University Hospital, Croydon, UK; 77 Morriston Hospital, Swansea, UK; 78 Queen Elizabeth University Hospital, Glasgow, UK; 79 Broomfield Hospital, Chelmsford, UK; 80 Heartlands Hospital, Birmingham, UK; 81 Royal Sussex County Hospital, Brighton, UK; 82 York Hospital, York, UK; 83 Queen Elizabeth Hospital, Birmingham, UK; 84 Royal Glamorgan Hospital, Pontyclun, UK; 85 Barnet Hospital, London, UK; 86 Wythenshawe Hospital, Manchester, UK; 87 Medway Maritime Hospital, Gillingham, UK; 88 Royal Berkshire NHS Foundation Trust, Reading, UK; 89 Whiston Hospital, Prescot, UK; 90 The Royal Oldham Hospital, Manchester, UK; 91 Chesterfield Royal Hospital Foundation Trust, Chesterfield, UK; 92 Aberdeen Royal Infirmary, Aberdeen, UK; 93 Royal Devon and Exeter Hospital, Exeter, UK; 94 Glasgow Royal Infirmary, Glasgow, UK; 95 Blackpool Victoria Hospital, Blackpool, UK; 96 Southampton General Hospital, Southampton, UK; 97 Ashford and St Peter’s Hospital, Chertsey, UK; 98 Derriford Hospital, Plymouth, UK; 99 East Surrey Hospital, Redhill, UK; 100 Poole Hospital, Poole, UK; 101 Royal Alexandra Hospital, Paisley, UK; 102 St James’s University Hospital and Leeds General Infirmary, Leeds, UK; 103 Bedford Hospital, Bedford, UK; 104 Southport and Formby District General Hospital, Ormskirk, UK; 105 The Tunbridge Wells Hospital and Maidstone Hospital, Kent, UK; 106 Queen Elizabeth Hospital, Woolwich, London, UK; 107 North Manchester General Hospital, Manchester, UK; 108 Royal Victoria Infirmary, Newcastle Upon Tyne, UK; 109 Hull Royal Infirmary, Hull, UK; 110 Manchester Royal Infirmary, Manchester, UK; 111 Royal Derby Hospital, Derby, UK; 112 Aintree University Hospital, Liverpool, UK; 113 Fairfield General Hospital, Bury, UK; 114 Norfolk and Norwich University Hospital (NNUH), Norwich, UK; 115 Milton Keynes University Hospital, Milton Keynes, UK; 116 Good Hope Hospital, Birmingham, UK; 117 Queen Elizabeth Hospital Gateshead, Gateshead, UK; 118 Royal Bolton Hospital, Bolton, UK; 119 Tameside General Hospital, Ashton Under Lyne, UK; 120 Salford Royal Hospital, Manchester, UK; 121 Great Ormond St Hospital and UCL Great Ormond St Institute of Child Health NIHR Biomedical Research Centre, London, UK; 122 Southmead Hospital, Bristol, UK; 123 William Harvey Hospital, Ashford, UK; 124 Arrowe Park Hospital, Wirral, UK; 125 Royal Hampshire County Hospital, Winchester, UK; 126 Bradford Royal Infirmary, Bradford, UK; 127 Glan Clwyd Hospital, Bodelwyddan, UK; 128 Royal Bournemouth Hospital, Bournemouth, UK; 129 Bristol Royal Infirmary, Bristol, UK; 130 University Hospital North Durham, Darlington, UK; 131 Darlington Memorial Hospital, Darlington, UK; 132 Basildon Hospital, Basildon, UK; 133 University College Hospital, London, UK; 134 Whittington Hospital, London, UK; 135 Western General Hospital, Edinburgh, UK; 136 Ipswich Hospital, Ipswich, UK; 137 Hereford County Hospital, Hereford, UK; 138 Sunderland Royal Hospital, Sunderland, UK; 139 Queens Hospital Burton, Burton-On-Trent, UK; 140 Musgrove Park Hospital, Taunton, UK; 141 The Royal Papworth Hospital, Cambridge, UK; 142 University Hospital Lewisham, London, UK; 143 The Princess Alexandra Hospital, Harlow, UK; 144 University Hospital of Wales, Cardiff, UK; 145 West Middlesex Hospital, Isleworth, UK; 146 Royal Albert Edward Infirmary, Wigan, UK; 147 Stoke Mandeville Hospital, Aylesbury, UK; 148 Royal Lancaster Infirmary, Lancaster, UK; 149 Basingstoke and North Hampshire Hospital, Basingstoke, UK; 150 Worthing Hospital, Worthing, UK; 151 St Richard’s Hospital, Chichester, UK; 152 The Alexandra Hospital, Redditch and Worcester Royal Hospital, Worcester, UK; 153 Royal Cornwall Hospital, Truro, UK; 154 Watford General Hospital, Watford, UK; 155 Macclesfield District General Hospital, Macclesfield, UK; 156 Royal Surrey County Hospital, Guildford, UK; 157 Rotherham General Hospital, Rotherham, UK; 158 Craigavon Area Hospital, Portadown, UK; 159 King’s Mill Hospital, Nottingham, UK; 160 Dumfries and Galloway Royal Infirmary, Dumfries, UK; 161 Prince Charles Hospital, Merthyr Tydfil, UK; 162 Ysbyty Gwynedd, Bangor, UK; 163 Royal Preston Hospital, Preston, UK; 164 The Great Western Hospital, Swindon, UK; 165 Lincoln County Hospital, Lincoln, UK; 166 University Hospital of North Tees, Stockton on Tees, UK; 167 Glangwili General Hospital, Camarthen, UK; 168 Southend University Hospital, Westcliff-on-Sea, UK; 169 Lister Hospital, Stevenage, UK; 170 Diana Princess of Wales Hospital, Grimsby, UK; 171 West Suffolk Hospital, Bury St Edmunds, UK; 172 Victoria Hospital, Kirkcaldy, UK; 173 Calderdale Royal Hospital, Halifax, UK; 174 Huddersfield Royal Infirmary, Huddersfield, UK; 175 Dorset County Hospital, Dorchester, UK; 176 Russell’s Hall Hospital, Dudley, UK; 177 Royal United Hospital, Bath, UK; 178 St Mary’s Hospital, Newport, UK; 179 George Eliot Hospital NHS Trust, Nuneaton, UK; 180 Yeovil Hospital, Yeovil, UK; 181 Forth Valley Royal Hospital, Falkirk, UK; 182 Frimley Park Hospital, Camberley, UK; 183 Chelsea & Westminster NHS Foundation Trust, London, UK; 184 Queen Elizabeth the Queen Mother Hospital, Margate, UK; 185 Royal Brompton Hospital, London, UK; 186 Darent Valley Hospital, Dartford, UK; 187 University Hospital Crosshouse, Kilmarnock, UK; 188 University Hospital Wishaw, Wishaw, UK; 189 University College Dublin, St Vincent’s University Hospital, Dublin, Ireland; 190 The Queen Elizabeth Hospital, King’s Lynn, UK; 191 Walsall Manor Hospital, Walsall, UK; 192 Princess Royal Hospital, Brighton, UK; 193 Barnsley Hospital, Barnsley, UK; 194 Warrington General Hospital, Warrington, UK; 195 Royal Victoria Hospital, Belfast, UK; 196 Royal Hallamshire Hospital and Northern General Hospital, Sheffield, UK; 197 Harefield Hospital, London, UK; 198 Cumberland Infirmary, Carlisle, UK; 199 Eastbourne District General Hospital, Eastbourne, UK; 200 Conquest Hospital, Saint Leonards-on-Sea, UK; 201 Salisbury District Hospital, Salisbury, UK; 202 Airedale General Hospital, Keighley, UK; 203 Leicester Royal Infirmary, Leicester, UK; 204 Peterborough City Hospital, Peterborough, UK; 205 Hinchingbrooke Hospital, Huntingdon, UK; 206 Colchester General Hospital, Colchester, UK; 207 Princess Royal Hospital, Telford and Royal Shrewsbury Hospital, Shrewsbury, UK; 208 University Hospital Monklands, Airdrie, UK; 209 Wrexham Maelor Hospital, Wrexham, UK; 210 New Cross Hospital, Wolverhampton, UK; 211 University Hospital Hairmyres, East Kilbride, UK; 212 Warwick Hospital, Warwick, UK; 213 Sandwell General Hospital and City Hospital, Birmingham, UK; 214 Royal Manchester Children’s Hospital, Manchester, UK; 215 Gloucestershire Royal Hospital, Gloucester, UK; 216 University Hospitals Coventry & Warwickshire NHS Trust, Coventry, UK; 217 Torbay Hospital, Torquay, UK; 218 Pilgrim Hospital, Lincoln, UK; 219 Prince Philip Hospital, Lianelli, UK; 220 Princess of Wales Hospital, Llantrisant, UK; 221 Northampton General Hospital NHS Trust, Northampton, UK; 222 The Christie NHS Foundation Trust, Manchester, UK; 223 James Paget University Hospital NHS Trust, Great Yarmouth, UK; 224 Birmingham Children’s Hospital, Birmingham, UK; 225 Withybush General Hospital, Haverfordwest, UK; 226 Northwick Park Hospital, London, UK; 227 North Devon District Hospital, Barnstaple, UK; 228 Scunthorpe General Hospital, Scunthorpe, UK; 229 Royal Free Hospital, London, UK; 230 Raigmore Hospital, Inverness, UK; 231 West Cumberland Hospital, Whitehaven, UK; 232 Furness General Hospital, Barrow-in-Furness, UK; 233 Liverpool Heart and Chest Hospital, Liverpool, UK; 234 Scarborough General Hospital, Scarborough, UK; 235 Bronglais General Hospital, Aberystwyth, UK; 236 Alder Hey Children’s Hospital, Liverpool, UK; 237 Borders General Hospital, Melrose, UK; 238 Leighton Hospital, Crewe, UK; 239 Kent & Canterbury Hospital, Canterbury, UK; 240 Harrogate and District NHS Foundation Trust, Harrogate, UK; 241 The Royal Marsden Hospital, London, UK; 242 Ealing Hospital, Southall, UK; 243 St John’s Hospital Livingston, Livingston, UK; 244 Wexham Park Hospital, Slough, UK; 245 Sheffield Children’s Hospital, Sheffield, UK; 246 Homerton University Hospital Foundation NHS Trust, London, UK; 247 National Hospital for Neurology and Neurosurgery, London, UK; 248 The Royal Alexandra Children’s Hospital, Brighton, UK; 249 Golden Jubilee National Hospital, Clydebank, UK; 250 Hospital Universitario Mostoles, Medicina Interna, Madrid, Spain; 251 Universidad Francisco de Vitoria, Madrid, Spain; 252 Haemostasis and Thrombosis Unit, Hospital de la Santa Creu i Snt Pau, IIB Sant Pau, Barcelona, Spain; 253 Unit of Infectious Diseases, Hospital Universitario 12 de Octubre, Instituto de Investigación Sanitaria Hospital 12 de Octubre (imas12), Madrid, Spain; 254 Spanish Network for Research in Infectious Diseases (REIPI RD16/0016/0002), Instituto de Salud Carlos III, Madrid, Spain; 255 School of Medicine, Universidad Complutense, Madrid, Spain; 256 Centro de Investigación Biomédica en Red de Enfermedades Infecciosas (CIBERINFEC), Instituto de Salud Carlos III, Madrid, Spain; 257 Hospital General Santa Bárbara de Soria, Soria, Spain; 258 Pediatric Neurology Unit, Department of Pediatrics, Navarra Health Service Hospital, Pamplona, Spain; 259 Navarra Health Service, NavarraBioMed Research Group, Pamplona, Spain; 260 Complejo Asistencial Universitario de León, León, Spain; 261 Infectious Diseases Department, Hospital Universitario San Pedro, Logroño, Spain; 262 Fundación Institut Guttmann, Institut Universitari de Neurorehabilitació adscrit a la UAB, Hospital de Neurorehabilitació, Barcelona, Spain; 263 Universitat Autònoma de Barcelona (UAB), Barcelona, Spain; 264 Fundació Institut d’Investigació en Ciències de la Salut Germans Trias i Pujol, Barcelona, Spain; 265 Hospital General de Occidente, Guadalajara, Mexico; 266 Microbiology Unit, Hospital Universitario N.S. de Candelaria, Santa Cruz de Tenerife, Spain; 267 Servicio de Neumología, Hospital Universitario La Paz-IDIPAZ, Madrid, Spain; 268 Camino Universitario Adelita de Char, Mired IPS, Barranquilla, Colombia; 269 Facultad de Ciencias de la Salud, Universidad Simón Bolívar, Barranquilla, Colombia; 270 Neumología, Hospital Universitario Virgen Macarena, Seville, Spain; 271 Departamento de Medicina, Universidad de Salamanca, Salamanca, Spain; 272 Centro de Investigación del Cáncer (IBMCC), Universidad de Salamanca, CSIC, Salamanca, Spain; 273 Biomedical Research Institute of Salamanca (IBSAL), Salamanca, Spain; 274 Centre for Biomedical Network Research on Cancer (CIBERONC), Instituto de Salud Carlos III, Madrid, Spain; 275 Department of Genetics & Genomics, Instituto de Investigación Sanitaria–Fundación Jiménez Díaz University Hospital—Universidad Autónoma de Madrid (IIS-FJD, UAM), Madrid, Spain; 276 Human Genotyping—CEGEN Unit, Spanish National Cancer Research Centre, Madrid, Spain; 277 Servicio de Medicina Interna, Hospital Universitario de Salamanca-IBSAL, Salamanca, Spain; 278 Universidad de Salamanca, Salamanca, Spain; 279 Department of Child and Adolescent Psychiatry, Institute of Psychiatry and Mental Health, Hospital General Universitario Gregorio Marañón (IiSGM), Madrid, Spain; 280 Clinical Pharmacology Service, Hospital de la Santa Creu i Sant Pau, IIB Sant Pau, Barcelona, Spain; 281 Biocruces Bizkai HRI, Barakaldo, Spain; 282 Cruces University Hospital, Osakidetza, Barakaldo, Spain; 283 Hospital Infanta Elena, Madrid, Spain; 284 Instituto de Investigación Sanitaria–Fundación Jiménez Díaz University Hospital, Universidad Autónoma de Madrid (IIS-FJD, UAM), Madrid, Spain; 285 Centre for Biomedical Network Research on Mental Health (CIBERSAM), Instituto de Salud Carlos III, Madrid, Spain; 286 Fundación Hospital Infantil Universitario de San José, Bogotá, Colombia; 287 Fundación Universitaria de Ciencias de la Salud, Bogotá, Colombia; 288 Programa de Pós-graduação em Ciências da Saúde, Universidade Federal do Rio Grande do Norte, Natal, Brazil; 289 Departamento de Medicina Clínica, Universidade Federal do Rio Grande do Norte, Natal, Brazil; 290 Departamento de Genética e Morfologia, Instituto de Ciências Biológicas, Universidade de Brasília, Brasilia, Brazil; 291 Colégio Marista de Brasilia, Brasilia, Brazil; 292 Associação Brasileira de Educação e Cultura, Londrina, Brazil; 293 Servicio de Medicina Interna, Hospital Universitario La Paz-IDIPAZ, Madrid, Spain; 294 Fundació Docència I Recerca Mutua Terrassa, Barcelona, Spain; 295 Spanish National Cancer Research Center, CNIO Biobank, Madrid, Spain; 296 Departamento Patologia y Laboratorios, Fundación Santa Fe de Bogota, Bogotá, Colombia; 297 Hospital General de Occidente, Zapopan, Mexico; 298 Centro Universitario de Tonalá, Universidad de Guadalajara, Tonalá, Mexico; 299 Centro de Investigación Multidisciplinario en Salud, Universidad de Guadalajara, Guadalajara, Mexico; 300 Instituto Murciano de Investigación Biosanitaria (IMIB-Arrixaca), Murcia, Spain; 301 Universidad Católica San Antonio de Murcia (UCAM), Murcia, Spain; 302 Servicio de Medicina Interna-Unidad de Enfermedades Infecciosas, Hospital Universitario de Salamanca-IBSAL, Salamanca, Spain; 303 Department of Internal Medicine, Hospital Universitario de Fuenlabrada, Madrid, Spain; 304 Laboratorio de Vigilancia Molecular Aplicada, Escola Tecnica de Saúde, Pará, Brazil; 305 Genetics Postgraduate Program, Federal University of Pernambuco, Recife, Brazil; 306 Servicio de Alergia, Hospital Universitario Infanta Leonor, Madrid, Spain; 307 Servicio de Medicina Intensiva, Hospital Universitario del Tajo, Toledo, Spain; 308 Hospital Universitario Mutua Terrassa, Barcelona, Spain; 309 Servicio de Farmacología, Hospital Universitario La Paz-IDIPAZ, Madrid, Spain; 310 Alcaldía de Barranquilla, Secretaría de Salud, Barranquilla, Colombia; 311 Xenética Cardiovascular, Instituto de Investigación Sanitaria de Santiago (IDIS), Santiago de Compostela, Spain; 312 Centre for Biomedical Network Research on Cardiovascular Diseases (CIBERCV), Instituto de Salud Carlos III, Madrid, Spain; 313 Unidad de Infección Viral e Inmunidad, Centro Nacional de Microbiología (CNM), Instituto de Salud Carlos III (ISCIII), Madrid, Spain; 314 Cardiovascular Genetics Center, Institut d’Investigació Biomèdica Girona (IDIBGI), Girona, Spain; 315 Medical Science Department, School of Medicine, University of Girona, Girona, Spain; 316 Cardiology Service, Hospital Josep Trueta, Girona, Spain; 317 Institute of Biomedicine of Seville (IBiS), Consejo Superior de Investigaciones Científicas (CSIC), University of Seville, Virgen del Rocio University Hospital, Seville, Spain; 318 Division of Infectious Diseases, Instituto de Investigación Sanitaria–Fundación Jiménez Díaz University Hospital, Universidad Autónoma de Madrid (IIS-FJD, UAM), Madrid, Spain; 319 Intensive Care Unit, Hospital Universitario Insular de Gran Canaria, Las Palmas de Gran Canaria, Spain; 320 Hospital Universitario Mutua Terrassa, Terrassa, Spain; 321 Departemento de Medicina, Hospital Universitario Virgen del Rocío, Universidad de Sevilla, Seville, Spain; 322 Centre for Biomedical Network Research on Epidemiology and Public Health (CIBERESP), Instituto de Salud Carlos III, Madrid, Spain; 323 Instituto de Biomedicina de Sevilla, Seville, Spain; 324 Facultad de Ciencias, Universidad de los Andes, Bogotá, Colombia; 325 Department of Nutrition, University of Fortaleza (UNIFOR), Fortaleza, Brazil; 326 Departamento de Química, Faculdade de Filosofia, Ciências e Letras de Ribeirão Preto, Universidade de São Paulo, São Paulo, Brazil; 327 Andalusian Public Health System Biobank, Granada, Spain; 328 Programa de Pós-Graduação em Ciências Farmacêuticas, Universidade Federal do Rio Grande do Norte, Natal, Brazil; 329 Neuromuscular Unit, Neurology Department, Hospital Universitari de Bellvitge, L’Hospitalet de Llobregat, Barcelona, Spain; 330 Bellvitge Biomedical Research Institute (IDIBELL), Neurometabolic Diseases Laboratory, L’Hospitalet de Llobregat, Barcelona, Spain; 331 Osakidetza, Cruces University Hospital, Barakaldo, Spain; 332 Centre for Biomedical Network Research on Diabetes and Metabolic Associated Diseases (CIBERDEM), Instituto de Salud Carlos III, Madrid, Spain; 333 University of Pais Vasco, UPV/EHU, Bizkaia, Spain; 334 Oncology and Genetics Unit, Instituto de Investigacion Sanitaria Galicia Sur, Xerencia de Xestion Integrada de Vigo-Servizo Galego de Saúde, Vigo, Spain; 335 Hospital Universitario La Paz, Hospital Carlos III, Madrid, Spain; 336 Hospital de San José, Sociedad de Cirugía de Bogota, Bogotá, Colombia; 337 Hospital Universitario Río Hortega, Valladolid, Spain; 338 Servicio de Medicina Intensiva, Complejo Hospitalario Universitario de A Coruña (CHUAC), Sistema Galego de Saúde (SERGAS), A Coruña, Spain; 339 Preventive Medicine Department, Valencia University, Valencia, Spain; 340 Centre for Biomedical Network Research on Physiopatology of Obesity and Nutrition (CIBEROBN), Instituto de Salud Carlos III, Madrid, Spain; 341 Department of Microgravity and Translational Regenerative Medicine, Otto von Guericke University, Magdeburg, Germany; 342 Maternidade Escola Janário Cicco, Natal, Brazil; 343 Centro Nacional de Genotipado (CEGEN), Universidade de Santiago de Compostela, Santiago de Compostela, Spain; 344 Institute of Psychiatry and Mental Health, Hospital General Universitario Gregorio Marañón (IiSGM), Madrid, Spain; 345 Programa de Pós Graduação em Ciências da Saúde, Faculdade de Medicina, Universidade de Brasília, Brasilia, Brazil; 346 Fundació Docència I Recerca Mutua Terrassa, Terrassa, Spain; 347 Unidad de Genética, Hospital Universitario Mostoles, Madrid, Spain; 348 Internal Medicine Department, Instituto de Investigación Sanitaria–Fundación Jiménez Díaz University Hospital, Universidad Autónoma de Madrid (IIS-FJD, UAM), Madrid, Spain; 349 Universidade Federal do Rio Grande do Norte, Pós-graduação em Biotecnologia, Rede de Biotecnologia do Nordeste (Renorbio), Natal, Brazil; 350 Servicio de Medicina Interna, Hospital Universitario Severo Ochoa, Madrid, Spain; 351 Department of Preventive Medicine and Public Health, School of Medicine, Universidad Autónoma de Madrid, Madrid, Spain; 352 IdiPaz (Instituto de Investigación Sanitaria Hospital Universitario La Paz), Madrid, Spain; 353 Instituto Aragonés de Ciencias de la Salud (IACS), Zaragoza, Spain; 354 Instituto Investigación Sanitaria Aragón (IIS-Aragon), Zaragoza, Spain; 355 Programa de Pós Graduação em Nutrição, Universidade Federal do Rio Grande do Norte, Natal, Brazil; 356 Preventive Medicine Department, Instituto de Investigacion Sanitaria Galicia Sur, Xerencia de Xestion Integrada de Vigo-Servizo Galego de Saúde, Vigo, Spain; 357 Servicio de Medicina Interna, Hospital Universitario Virgen del Rocío, Seville, Spain; 358 Departamento de Infectologia, Universidade Federal do Rio Grande do Norte, Natal, Brazil; 359 Hospital de Doenças Infecciosas Giselda Trigueiro, Natal, Brazil; 360 Unidad Diagnóstico Molecular, Fundación Rioja Salud, La Rioja, Spain; 361 Hospital Universitario Quironsalud Madrid, Madrid, Spain; 362 Servicio de Cardiología, Hospital Universitario de Salamanca-IBSAL, Salamanca, Spain; 363 Servicio de Medicina Interna, Hospital Universitario Puerta de Hierro, Majadahonda, Spain; 364 Biocruces Bizkaia Health Research Institute, Galdakao University Hospital, Osakidetza, Bizkaia, Spain; 365 Instituto Regional de Investigación en Salud-Universidad Nacional de Caaguazú, Caaguazú, Paraguay; 366 Núcleo de Pesquisas em Oncologia, Universidade Federal do Pará, Belém, Brazil; 367 Departamento de Ensino e Pesquisa, Hospital Ophir Loyola, Belém, Brazil; 368 Fundación Asilo San Jose, Santander, Spain; 369 Unidad de Enfermedades Infecciosas, Servicio de Medicina Interna, Hospital Universitario Puerta de Hierro, Instituto de Investigación Sanitaria Puerta de Hierro—Segovia de Arana, Madrid, Spain; 370 Universidad Nacional de Asunción, Facultad de Politécnica, Paraguay; 371 Urgencias Hospitalarias, Complejo Hospitalario Universitario de A Coruña (CHUAC), Sistema Galego de Saúde (SERGAS), A Coruña, Spain; 372 Grupo de Investigación en Interacciones Gen-Ambiente y Salud (GIIGAS), Instituto de Biomedicina (IBIOMED), Universidad de León, León, Spain; 373 Pediatrics Department, Hospital Universitario Niño Jesús, Madrid, Spain; 374 Unitat de Malalties Infeccioses i Importades, Servei de Pediatría, Infectious and Imported Diseases, Pediatric Unit, Hospital Universitari Sant Joan de Deú, Barcelona, Spain; 375 Microbiology Department, Instituto de Investigación Sanitaria–Fundación Jiménez Díaz University Hospita, Universidad Autónoma de Madrid (IIS-FJD, UAM), Madrid, Spain; 376 Hospital de Niños Ricardo Gutierrez, Buenos Aires, Argentina; 377 University of Salamanca, Biomedical Research Institute of Salamanca (IBSAL), Salamanca, Spain; 378 Department of Immunology, IRYCIS, Hospital Universitario Ramón y Cajal, Madrid, Spain; 379 Servicio de Medicina Intensiva, Hospital Infanta Elena, Madrid, Spain; 380 Servicio de Genética, Hospital Universitario de Getafe, Madrid, Spain; 381 Pneumology Department, Hospital General Universitario Gregorio Marañón (iiSGM), Madrid, Spain; 382 Ministerio de Salud Ciudad de Buenos Aires, Buenos Aires, Argentina; 383 Unidad de Apoyo a la Investigación, Hospital Clinico Universitario de Valladolid, Valladolid, Spain; 384 Departamento de Cirugía, Universidad de Valladolid, Valladolid, Spain; 385 Secretaria Municipal de Saude de Apodi, Natal, Brazil; 386 Sección Genética Médica, Servicio de Pediatría, Hospital Clínico Universitario Virgen de la Arrixaca, Servicio Murciano de Salud, Murcia, Spain; 387 Departamento Cirugía, Pediatría, Obstetricia y Ginecología, Facultad de Medicina, Universidad de Murcia (UMU), Murcia, Spain; 388 Grupo Clínico Vinculado, Centre for Biomedical Network Research on Rare Diseases (CIBERER), Instituto de Salud Carlos III, Madrid, Spain; 389 Servicio de Análisis Clínicos e Inmunología, Hospital Universitario Virgen de las Nieves, Granada, Spain; 390 Hospital Universitario Centro Dermatológico Federico Lleras Acosta, Bogotá, Colombia; 391 Intermediate Respiratory Care Unit, Department of Pneumology, Instituto de Investigación Sanitaria–Fundación Jiménez Díaz University Hospital, Universidad Autónoma de Madrid (IIS-FJD, UAM), Madrid, Spain; 392 Clinica Comfamiliar Risaralda, Pereira, Colombia; 393 Centro Universitario de Tonalá, Universidad de Guadalajara, Guadalajara, Mexico; 394 Unidad de Cuidados Intensivos, Hospital Clínico Universitario de Santiago (CHUS), Sistema Galego de Saúde (SERGAS), Santiago de Compostela, Spain; 395 Plataforma de Farmacogenética, IIS La Fe, Valencia, Spain; 396 Departamento de Farmacología, Universidad de Valencia, Valencia, Spain; 397 Data Analysis Department, Instituto de Investigación Sanitaria–Fundación Jiménez Díaz University Hospital, Universidad Autónoma de Madrid (IIS-FJD, UAM), Madrid, Spain; 398 Infectious Diseases Service, Hospital del Mar, Barcelona, Spain; 399 Institut Hospital del Mar d’Investigacions Mèdiques (IMIM), Barcelona, Spain; 400 CEXS-Universitat Pompeu Fabra, Spanish Network for Research in Infectious Diseases (REIPI), Barcelona, Spain; 401 Biocruces Bizkaia Health Research Institute, Basurto University Hospital, Osakidetza, Bizkaia, Spain; 402 Infectious Diseases, Microbiota and Metabolism Unit, Center for Biomedical Research of La Rioja (CIBIR), Logroño, Spain; 403 Sabin Medicina Diagnóstica, São Paulo, Brazil; 404 Opthalmology Department, Instituto de Investigación Sanitaria–Fundación Jiménez Díaz University Hospital, Universidad Autónoma de Madrid (IIS-FJD, UAM), Madrid, Spain; 405 Pediatric Critical Care Unit, Hospital Sant Joan de Deu, Barcelona, Spain; 406 Paediatric Intensive Care Unit, Agrupación Hospitalaria Clínic-Sant Joan de Déu, Esplugues de Llobregat, Barcelona, Spain; 407 Department of Immunology, Hospital Universitario 12 de Octubre, Madrid, Spain; 408 Transplant Immunology and Immunodeficiencies Group, Instituto de Investigación Sanitaria Hospital 12 de Octubre (imas12), Madrid, Spain; 409 SIGEN Alianza Universidad de los Andes, Fundación Santa Fe de Bogotá, Bogotá, Colombia; 410 Medicina Intensiva, Hospital General de Segovia, Segovia, Spain; 411 Clinical Trials Unit, Instituto de Investigación Sanitaria–Fundación Jiménez Díaz University Hospital, Universidad Autónoma de Madrid (IIS-FJD, UAM), Madrid, Spain; 412 IMDEA-Food Institute, CEI UAM+CSIC, Madrid, Spain; 413 Servicio de Enfermedades Infecciosas, Hospital Universitario Virgen de las Nieves, Granada, Spain; 414 Instituto de Investigación Biosanitaria de Granada (ibs.GRANADA), Granada, Spain; 415 Departamento de Medicina, Universidad de Granada, Granada, Spain; 416 Servicio de Inmunología, Hospital Universitario La Paz-IDIPAZ, Madrid, Spain; 417 Lymphocyte Pathophysiology in Immunodeficiencies Group, La Paz Institute for Health Research (IdiPAZ), Madrid, Spain; 418 Intensive Care Unit, Hospital Universitario de Canarias, La Laguna, Spain; 419 Dirección General de Salud Pública, Consejería de Sanidad, Junta de Castilla y León, Valladolid, Spain; 420 Departamento de Analises Clinicas e Toxicologicas, Universidade Federal do Rio Grande do Norte, Natal, Brazil; 421 Epidemiology, Fundación Jiménez Díaz, Madrid, Spain; 422 Department of Medicine, Universidad Autónoma de Madrid, Madrid, Spain; 423 Instituto de Biomedicina (IBIOMED), Universidad de León, León, Spain; 424 Intensive Care Unit, Hospital Universitario N. S. de Candelaria, Santa Cruz de Tenerife, Spain; 425 Preventive Medicine Department, Instituto de Investigación Sanitaria–Fundación Jiménez Díaz University Hospital, Universidad Autónoma de Madrid (IIS-FJD, UAM), Madrid, Spain; 426 Departamento de Medicina, Universidad de Valladolid, Valladolid, Spain; 427 Servicio de Medicina Intensiva, Hospital Universitario Infanta Leonor, Madrid, Spain; 428 Servicio de Medicina Interna, Sanatorio Franchin, Buenos Aires, Argentina; 429 Unidad de Genética y Genómica Islas Baleares, Hospital Universitario Son Espases, Islas Baleares, Spain; 430 Unidad de Diagnóstico Molecular y Genética Clínica, Hospital Universitario Son Espases, Islas Baleares, Spain; 431 Genomics of Complex Diseases Unit, Research Institute of Hospital de la Santa Creu i Sant Pau, IIB Sant Pau, Barcelona, Spain; 432 Faculdade de Medicina, Universidade de Brasília, Brasilia, Brazil; 433 Programa de Pós-Graduação em Ciências Médicas, Universidade de Brasília, Brasilia, Brazil; 434 Programa de Pós-Graduação em Ciências da Saúde, Universidade de Brasília, Brasilia, Brazil; 435 Hospital das Forças Armadas, Brasília, Brazil; 436 Exército Brasileiro, Cruzeiro, Brazil; 437 Hospital El Bierzo, Gerencia de Asistencia Sanitaria del Bierzo (GASBI), Gerencia Regional de Salud (SACYL), Ponferrada, Spain; 438 Grupo INVESTEN, Instituto de Salud Carlos III, Madrid, Spain; 439 Unidad de Cuidados Intensivos, Complejo Universitario de A Coruña (CHUAC), Sistema Galego de Saúde (SERGAS), A Coruña, Spain; 440 Unidad Cuidados Intensivos, Hospital El Bierzo, León, Spain; 441 Familial Cancer Clinical Unit, Spanish National Cancer Research Centre, Madrid, Spain; 442 Instituto de Investigación Sanitaria San Carlos (IdISSC), Hospital Clínico San Carlos (HCSC), Madrid, Spain; 443 Departamento de Enfermería, Universidad de Sevilla, Seville, Spain; 444 Hospital General Universitario Gregorio Marañón (IiSGM), Madrid, Spain; 445 ERN-ITHACA-European Reference Network on Rare Congenital Malformations and Rare Intellectual Disability, Brussels, Belgium; 446 Unidad de Genética y Genómica Islas Baleares, Unidad de Diagnóstico Molecular y Genética Clínica, Hospital Universitario Son Espases, Islas Baleares, Spain; 447 Instituto de Investigación Sanitaria Islas Baleares (IdISBa), Islas Baleares, Spain; 448 Programa de Pós-Graduação em Biologia Animal, Universidade de Brasília, Brasília, Brazil; 449 Programa de Pós-Graduação Profissional em Ensino de Biologia, Universidade de Brasília, Brasília, Brazil; 450 Anatomía Patológica, Instituto de Investigación Sanitaria San Carlos (IdISSC), Hospital Clínico San Carlos (HCSC), Madrid, Spain; 451 Tecnológico de Monterrey, Monterrey, Mexico; 452 Centro de Investigación en Anomalías Congénitas y Enfermedades Raras (CIACER), Universidad Icesi, Cali, Colombia; 453 Departamento de Genetica, Fundación Valle del Lili, Cali, Colombia; 454 Department of Immunology, Ophthalmology and ENT, Universidad Complutense de Madrid, Madrid, Spain; 455 Department of Neumology, Instituto de Investigación Sanitaria–Fundación Jiménez Díaz University Hospital, Universidad Autónoma de Madrid (IIS-FJD, UAM), Madrid, Spain; 456 Hospital Nuestra Señora de Sonsoles, Ávila, Spain; 457 Inditex, A Coruña, Spain; 458 Intensive Care Department, Instituto de Investigación Sanitaria–Fundación Jiménez Díaz University Hospital, Universidad Autónoma de Madrid (IIS-FJD, UAM), Madrid, Spain; 459 Servicio de Microbiología Clínica, Hospital Universitario Príncipe de Asturias, Madrid, Spain; 460 Departamento de Biomedicina y Biotecnología, Facultad de Medicina y Ciencias de la Salud, Universidad de Alcalá de Henares, Madrid, Spain; 461 GENYCA, Madrid, Spain; 462 Marinha do Brasil, Brasil, Brazil; 463 Universidade de Brasília, Brasilia, Brazil; 464 Neuromuscular Diseases Unit, Department of Neurology, Hospital de la Santa Creu i Sant Pau, Universitat Autònoma de Barcelona, Barcelona, Spain; 465 Unidad de Investigación Médica en Enfermedades Infecciosas y Parasitarias, Instituto Mexicano del Seguro Social (IMSS), Centro Médico Nacional Siglo XXI, Mexico City, Mexico; 466 Catalan Institution of Research and Advanced Studies (ICREA), Barcelona, Spain; 467 Drug Research Centre, Institut d’Investigació Biomèdica Sant Pau, IIB-Sant Pau, Barcelona, Spain; 468 Departamento de Genetica, Clinica imbanaco, Cali, Colombia; 469 Department of Immunology, Hospital Universitario de Gran Canaria Dr Negrín, Las Palmas de Gran Canaria, Spain; 470 Pediatrics Department, University Hospital Germans Trias i Pujol, Badalona, Spain; 471 Department of Pathology, Biobank, Instituto de Investigación Sanitaria–Fundación Jiménez Díaz University Hospital, Universidad Autónoma de Madrid (IIS-FJD, UAM), Madrid, Spain; 472 Faculdade de Ciências da Saúde, Universidade de Brasília, Brasilia, Brazil; 473 Servicio de Medicina Interna, Hospital Universitario Virgen de las Nieves, Granada, Spain; 474 Grupo de Ciencias Básicas en Salud (CBS), Fundación Universitaria de Ciencias de la Salud, Bogotá, Colombia; 475 Sociedad de Cirugía de Bogotá, Hospital de San José, Bogotá, Colombia; 476 Departamento Bioquímica, Biología Molecular e Inmunología III, Universidad de Granada, Granada, Spain; 477 Allergy Unit, Hospital Infanta Elena, Madrid, Spain; 478 Faculty of Medicine, Universidad Francisco de Vitoria, Madrid, Spain; 479 Hospital Universitario Infanta Leonor, Madrid, Spain; 480 Complutense University of Madrid, Madrid, Spain; 481 Gregorio Marañón Health Research Institute (IiSGM), Madrid, Spain; 482 Reumathology Service, Instituto de Investigación Sanitaria–Fundación Jiménez Díaz University Hospital, Universidad Autónoma de Madrid (IIS-FJD, UAM), Madrid, Spain; 483 Biobank, Puerta de Hierro-Segovia de Arana Health Research Institute, Madrid, Spain; 484 Universidad Rey Juan Carlos, Madrid, Spain; 485 The John Walton Muscular Dystrophy Research Centre, Newcastle University and Newcastle Hospitals NHS Foundation Trust, Newcastle upon Tyne, UK; 486 Neuromuscular Unit, Neuropediatrics Department, Institut de Recerca Sant Joan de Déu, Hospital Sant Joan de Déu, Barcelona, Spain; 487 Casa de Saúde São Lucas, Natal, Brazil; 488 Hospital Rio Grande, Natal, Brazil; 489 Intensive Care Unit, Hospital Universitario de Gran Canaria Dr Negrín, Las Palmas de Gran Canaria, Spain; 490 Universidad Fernando Pessoa Canarias, Las Palmas de Gran Canaria, Spain; 491 Servicio de Anestesiologia y Reanimación, Hospital Clinico Universitario de Valladolid, Valladolid, Spain; 492 Servicio de Hematologia y Hemoterapia, Hospital Clinico Universitario de Valladolid, Valladolid, Spain; 493 Hospital Universitario Lauro Wanderley, João Pessoa, Brazil; 494 Servicio de Medicina Interna, Hospital Universitario Infanta Leonor, Madrid, Spain; 495 University Hospital of Burgos, Burgos, Spain; 496 Universidad de Sevilla, Seville, Spain; 497 Fundación Santa Fe de Bogota, Instituto de Servicios Medicos de Emergencia y Trauma, Bogotá, Colombia; 498 Universidad de los Andes, Bogotá, Colombia; 499 Quironprevención, A Coruña, Spain; 500 Junta de Castilla y León, Consejería de Sanidad, Valladolid, Spain; 501 Gerencia Atención Primaria de Burgos, Burgos, Spain; 502 Immunogenetics–Histocompatibility group, Servicio de Inmunología, Instituto de Investigación Sanitaria Puerta de Hierro, Segovia de Arana, Madrid, Spain; 503 Department of Infectious Diseases, Hospital del Mar, Barcelona, Spain; 504 IMIM—Hospital del Mar Medical Research Institute, Institut Hospital del Mar d’Investigacions Mediques, Barcelona, Spain; 505 Department of Medicine, Universitat Autònoma de Barcelona, Barcelona, Spain; 506 Consejería de Sanidad, Comunidad de Madrid, Madrid, Spain; 507 Centro para el Desarrollo de la Investigación Científica, Asunción, Paraguay; 508 School of Informatics, University of Edinburgh, Edinburgh, UK; 509 Section of Biomolecular Medicine, Division of Systems Medicine, Department of Metabolism, Digestion and Reproduction, Sir Alexander Fleming Building, Imperial College London, London, UK; 510 Section of Genomic and Environmental Medicine, Respiratory Division, National Heart and Lung Institute, London, UK; 511 Section of Molecular Virology, Imperial College London, London, UK; 512 Antimicrobial Resistance and Hospital Acquired Infection Department, Public Health England, London, UK; 513 Department of Epidemiology and Biostatistics, School of Public Health, Faculty of Medicine, Imperial College London, London, UK; 514 Department of Infectious Disease, Imperial College London, London, UK; 515 MRC-University of Glasgow Centre for Virus Research, Glasgow, UK; 516 The Florey Institute for Host-Pathogen Interactions, Department of Infection, Immunity and Cardiovascular Disease, University of Sheffield, Sheffield, UK; 517 Centre for Medical Informatics, The Usher Institute, University of Edinburgh, Edinburgh, UK; 518 National Phenome Centre, Department of Metabolism, Digestion and Reproduction, Imperial College London, London, UK; 519 Section of Bioanalytical Chemistry, Department of Metabolism, Digestion and Reproduction, Imperial College London, London, UK; 520 European Genomic Institute for Diabetes, CNRS UMR 8199, INSERM UMR 1283, Institut Pasteur de Lille, Lille University Hospital, University of Lille, Lille, France; 521 McGill University and Genome Quebec Innovation Centre, Montréal, Quebec Canada; 522 National Infection Service, Public Health England, London, UK; 523 Liverpool School of Tropical Medicine, Liverpool, UK; 524 Institute of Microbiology and Infection, University of Birmingham, Birmingham, UK; 525 Department of Molecular and Clinical Cancer Medicine, University of Liverpool, Liverpool, UK; 526 Institute for Global Health, University College London, London, UK; 527 Department of Infectious Diseases, Queen Elizabeth University Hospital, Glasgow, UK; 528 University of Liverpool, Liverpool, UK; 529 Virology Reference Department, National Infection Service, Public Health England, London, UK; 530 Department of Pharmacology, University of Liverpool, Liverpool, UK; 531 Nuffield Department of Medicine, Peter Medawar Building for Pathogen Research, University of Oxford, Oxford, UK; 532 Translational Gastroenterology Unit, Nuffield Department of Medicine, University of Oxford, Oxford, UK; 533 Nottingham University Hospitals NHS Trust, Nottingham, UK; 534 Nuffield Department of Medicine, John Radcliffe Hospital, Oxford, UK; 535 Department of Microbiology/Infectious Diseases, Oxford University Hospitals NHS Foundation Trust, John Radcliffe Hospital, Oxford, UK; 536 ISARIC Global Support Centre, Centre for Tropical Medicine and Global Health, Nuffield Department of Medicine, University of Oxford, Oxford, UK; 537 Institute of Infection, Veterinary and Ecological Sciences, University of Liverpool, Liverpool, UK; 538 Division of Infection and Immunity, University College London, London, UK; 539 Molecular and Clinical Cancer Medicine, Institute of Systems, Molecular and Integrative Biology, University of Liverpool, Liverpool, UK; 540 Clatterbridge Cancer Centre NHS Foundation Trust, Liverpool, UK; 541 NIHR Health Protection Research Unit in Emerging and Zoonotic Infections, Liverpool, UK; 542 Centre for Clinical Infection and Diagnostics Research, Department of Infectious Diseases, School of Immunology and Microbial Sciences, King’s College London, London, UK; 543 Department of Infectious Diseases, Guy’s and St Thomas’ NHS Foundation Trust, London, UK; 544 Institute of Evolutionary Biology, University of Edinburgh, Edinburgh, UK; 545 Department of Pediatrics and Virology, St Mary’s Medical School Building, Imperial College London, London, UK; 546 NHS Greater Glasgow & Clyde, Glasgow, UK; 547 Walton Centre NHS Foundation Trust, Liverpool, UK; 548 MRC Centre for Molecular Bacteriology and Infection, Imperial College London, London, UK; 549 Department of Child Life and Health, University of Edinburgh, Edinburgh, UK; 550 National Phenome Centre, Division of Systems Medicine, Department of Metabolism, Digestion and Reproduction, Imperial College London, London, UK; 551 Blood Borne Virus Unit, Virus Reference Department, National Infection Service, Public Health England, London, UK; 552 Transfusion Microbiology, National Health Service Blood and Transplant, London, UK; 553 Department of Medicine, Imperial College London, London, UK; 554 Department of Infection, Immunity and Cardiovascular Disease, University of Sheffield, Sheffield, UK; 555 Tropical & Infectious Disease Unit, Royal Liverpool University Hospital, Liverpool, UK; 556 Liverpool Clinical Trials Centre, University of Liverpool, Liverpool, UK; 557 Public Health Scotland, Edinburgh, UK; 558 Centre for Health Informatics, Division of Informatics, Imaging and Data Science, School of Health Sciences, Faculty of Biology, Medicine and Health, University of Manchester, Manchester Academic Health Science Centre, Manchester, UK; 559 EPCC, University of Edinburgh, Edinburgh, UK; 560 ISARIC, Global Support Centre, COVID-19 Clinical Research Resources, Epidemic diseases Research Group, Oxford (ERGO), University of Oxford, Oxford, UK; 561 Institute of Infection, Veterinary and Ecological Sciences, Faculty of Health and Life Sciences, University of Liverpool, Liverpool, UK; 562 23andMe, Sunnyvale, CA USA; 563 Human genetics R&D, GSK Medicines Research Centre, Target Sciences R&D, Stevenage, UK

**Keywords:** Genome-wide association studies, Genetics research, SARS-CoV-2, Viral infection

Correction to: *Nature* 10.1038/s41586-023-06034-3 Published online 17 May 2023

In the version of this article initially published, the name of Ana Margarita Baldión-Elorza, of the SCOURGE Consortium, appeared incorrectly (as Ana María Baldion) and has now been amended in the HTML and PDF versions of the article.

