# Supplementary material for: GWAS and meta-analysis identifies 49 genetic variants underlying critical COVID-19
Source: Nature. 2023 May 17;617(7962):764–8. doi: 10.1038/s41586-023-06034-3 (PMC10208981; doi:10.1038/s41586-023-06034-3)
Supplement: Supplementary file 1 — Supplementary Sections 1–13, including Supplementary Figs. 1–54 and Supplementary Tables 7–17. [file 41586_2023_6034_MOESM1_ESM.pdf]

nature portfolio

https://doi.org/10.1038/s41586-023-06034-3

Supplementary information

GWAS and meta-analysis identifies 49 genetic variants underlying critical COVID-19

In the format provided by the authors and unedited

Supplementary material - GWAS and meta-analysis identifies 49 genetic variants underlying critical Covid-19.

Contents

1 Comorbidity **2**

2 Gene level analysis **2**

3 Sex Stratified GWAS **5**

4 TWAS **6**

5 Drugs in consideration by UK-CTAP **8**

6 Description of cohorts **9**
6.1 Critical and hospitalisation meta-analysis cohorts 9

7 Full GWAS results tables **11**
7.1 Forest plots 16

8 Mendelian randomisation for Protein expression (INTERVAL) **25**

9 Mendelian randomisation for RNA expression (eQTLgen) **25**

10 Fine mapping **42**

11 Change in allele frequency over time **60**

12 Contributing Studies **65**
12.1 Ethical approval 65
12.2 GenOMICC and ISARIC4C 65
12.3 SCOURGE 69
12.4 HGI release 6 69
12.5 23andMe 71

13 Contributors **71**
13.1 GenOMICC Investigators 71
13.2 SCOURGE Consortium 89
13.3 23andMe Investigators 95
13.4 ISARIC4C Investigators 95

References **98**

1 Comorbidity

Supplementary Table 7: Comorbities in a subset of GenOMICC cases who were also recruited to the ISARIC4C study in the UK providing electronic health care record linkage.

Name	Cases	N	Percentage
Chronic Cardiac Disease	1009	7573	13
Hypertension	2962	6933	43
Chronic Pulmonary Disease (not Asthma)	655	7579	9
Asthma (physician diagnosed)	1443	7641	18
Chronic Kidney Disease	582	7585	8
Chronic Neurological Disease	322	7583	4
Malignent Neoplasm	346	7572	5
Chronic Hematological Disease	184	7569	2
AIDS/HIV	48	7406	<1
Obesity	2377	7105	33
Diabetes	988	7163	14
Rheumatologic Disorder	633	7572	8
Dementia	30	7604	<1
Malnutrition	49	7356	<1

Supplementary Table 8: Comorbities in 3533 hospitaized cases in the GenOMICC Brazil cohort. This information has been previously presented.[1]

Comorbidity	N	%
Not Available	1514	43
Hypertension	1937	55
Cardiovascular D	996	30
ACEi users	395	14
Asthma	172	4.9
Tabagism	833	24
COPD	213	6.1
Diabetes mellitus type 2	1238	35
HIV	33	0.9
CKD	441	13
BMI status:		
Obese (BMI of 30kg/m2or greater)	735	36
Overweight (BMI of 25–29.9kg/m2)	683	34
Normal (BMI of 20–24.9kg/m2)	489	24
BMI of 20kg/m2or lower	113	5.6
Oncologic	170	4.9
Autoimmune diseases	101	2.9

Supplementary Table 9: Comorbidities in 9371 individuals in the SCOURGE cohort. This information has been previously presented.[2]

Comorbidity	N	%
Vascular/endocrinological	4099	44
Cardiac	1057	11
Nervous	773	8.3
Digestive	264	2.8
Onco-hematological	647	6.9
Respiratory	905	9.7

2 Gene level analysis

Supplementary Table 10: Gene level association results, showing genes with significant association after Bonferroni correction, with full results in Supplementary Material (Supplementary Table 6). Chr:pos$_{start}$ (b38): chromosome and start BP position on human genome build 38; P_{gene}: mBAT-combo gene level association P value; N_{snps}: number of SNPs used in association test for gene; Lead SNP: lead variant amongst variants used in test for gene level association; $P_{Lead\,SNP}$: association P value for lead variant.

Gene Symbol	Chr:pos$_{start}$ (b38)	P_{gene}	N_{snps}	Lead SNP	$P_{Lead\,SNP}$
PMVK	1:154924740	1.3×10^{-14}	212	1:154945075:C:T	1×10^{-8}
PBXIP1	1:154944076	7.3×10^{-16}	207	1:155003661:G:T	1.1×10^{-10}
PYGO2	1:154957026	4.3×10^{-19}	191	1:155003661:G:T	1.1×10^{-10}
SHC1	1:154962298	1.7×10^{-19}	203	1:155003661:G:T	1.1×10^{-10}
CKS1B	1:154974653	1×10^{-19}	188	1:155003661:G:T	1.1×10^{-10}
FLAD1	1:154983338	2.6×10^{-23}	198	1:155003661:G:T	1.1×10^{-10}
LENEP	1:154993586	4.3×10^{-23}	165	1:155003661:G:T	1.1×10^{-10}
ZBTB7B	1:155002630	2.2×10^{-29}	185	1:155068178:C:T	2×10^{-13}
DCST2	1:155018520	1.7×10^{-29}	199	1:155068178:C:T	2×10^{-13}
DCST1	1:155033824	4.6×10^{-40}	219	1:155090454:C:G	5.1×10^{-14}
ADAM15	1:155050566	4.9×10^{-45}	216	1:155090454:C:G	5.1×10^{-14}
EFNA4	1:155063737	4.9×10^{-47}	226	1:155090454:C:G	5.1×10^{-14}
ENSG00000251246	1:155063748	1.1×10^{-48}	271	1:155090454:C:G	5.1×10^{-14}
EFNA3	1:155078837	1.9×10^{-49}	237	1:155090454:C:G	5.1×10^{-14}
EFNA1	1:155127876	5.4×10^{-39}	254	1:155175305:G:A	1.6×10^{-15}
SLC50A1	1:155135344	2.3×10^{-37}	246	1:155175305:G:A	1.6×10^{-15}
DPM3	1:155139891	8.7×10^{-36}	235	1:155175305:G:A	1.6×10^{-15}
KRTCAP2	1:155169408	2.1×10^{-38}	201	1:155197995:A:G	6.6×10^{-24}
ENSG00000273088	1:155169409	5.5×10^{-37}	229	1:155197995:A:G	6.6×10^{-24}
TRIM46	1:155173787	3.1×10^{-36}	207	1:155197995:A:G	6.6×10^{-24}
MUC1	1:155185824	2.7×10^{-32}	196	1:155197995:A:G	6.6×10^{-24}
THBS3	1:155195588	1×10^{-33}	219	1:155197995:A:G	6.6×10^{-24}
MTX1	1:155208695	1.4×10^{-33}	194	1:155197995:A:G	6.6×10^{-24}
GBA	1:155234452	4.2×10^{-30}	196	1:155197995:A:G	6.6×10^{-24}
FAM189B	1:155247205	3×10^{-29}	202	1:155197995:A:G	6.6×10^{-24}
SCAMP3	1:155255979	1.2×10^{-18}	196	1:155212198:G:A	3.6×10^{-14}
CLK2	1:155262868	9.2×10^{-17}	206	1:155237942:C:T	1.7×10^{-13}
HCN3	1:155277463	3.3×10^{-18}	208	1:155237942:C:T	1.7×10^{-13}
PKLR	1:155289293	4.4×10^{-18}	203	1:155296105:G:A	2×10^{-8}
FDPS	1:155308748	3.4×10^{-15}	186	1:155355915:G:GT	2×10^{-9}
RUSC1	1:155320894	9.1×10^{-15}	171	1:155355915:G:GT	2×10^{-9}
ASH1L	1:155335268	1.1×10^{-21}	419	1:155419726:T:C	7.3×10^{-10}
MSTO1	1:155610205	5.4×10^{-17}	120	1:155623087:G:A	1.5×10^{-10}
YY1AP1	1:155659443	2.6×10^{-23}	164	1:155706701:A:G	9.3×10^{-12}
DAP3	1:155687960	1.3×10^{-19}	226	1:155706701:A:G	9.3×10^{-12}
GON4L	1:155749659	3.2×10^{-17}	379	1:155863616:T:C	1.7×10^{-12}
SYT11	1:155859567	3.5×10^{-18}	236	1:155863616:T:C	1.7×10^{-12}
RIT1	1:155897808	4.1×10^{-17}	214	1:155863616:T:C	1.7×10^{-12}
KHDC4	1:155913045	3×10^{-16}	208	1:155863616:T:C	1.7×10^{-12}
RXFP4	1:155941638	3.5×10^{-8}	171	1:155927586:C:G	8.4×10^{-8}
ARHGEF2	1:155946851	1.3×10^{-15}	292	1:156014716:A:G	2.3×10^{-11}
SSR2	1:156009048	2.3×10^{-13}	199	1:156014716:A:G	2.3×10^{-11}
UBQLN4	1:156035299	6.2×10^{-13}	221	1:156014716:A:G	2.3×10^{-11}
LAMTOR2	1:156054782	1.7×10^{-11}	184	1:156014716:A:G	2.3×10^{-11}
RAB25	1:156061160	1×10^{-8}	206	1:156014716:A:G	2.3×10^{-11}
MEX3A	1:156072013	5.1×10^{-8}	248	1:156075211:G:T	1.8×10^{-9}
LMNA	1:156082573	5.5×10^{-7}	372	1:156075211:G:T	1.8×10^{-9}
SEMA4A	1:156147366	3.1×10^{-7}	367	1:156107888:C:T	1.8×10^{-8}
SLC25A44	1:156193932	2×10^{-7}	307	1:156155844:A:G	7×10^{-6}
PMF1-BGLAP	1:156212982	1.6×10^{-6}	357	1:156225891:G:A	4×10^{-5}
PMF1	1:156212993	9.4×10^{-7}	347	1:156225891:G:A	4×10^{-5}
BCL11A	2:60450520	5.8×10^{-7}	537	2:60480453:A:G	9.2×10^{-15}
LARS2	3:45388561	2.6×10^{-15}	854	3:45587795:G:A	1×10^{-32}
LIMD1	3:45555394	2.4×10^{-32}	896	3:45632867:A:G	4.8×10^{-38}
SACM1L	3:45689056	1.5×10^{-69}	611	3:45756734:C:T	1.4×10^{-42}
SLC6A20	3:45755449	3.5×10^{-217}	412	3:45818159:G:A	4.4×10^{-254}
LZTFL1	3:45823316	1.8×10^{-209}	544	3:45818159:G:A	4.4×10^{-254}
CCR9	3:45886509	7.3×10^{-197}	341	3:45847198:A:G	1.3×10^{-252}
FYCO1	3:45917903	3.2×10^{-112}	629	3:45868152:C:A	1.9×10^{-181}
CXCR6	3:45940933	3.1×10^{-114}	355	3:45912847:C:T	1.9×10^{-139}
XCR1	3:46016990	4×10^{-96}	758	3:45981341:G:A	1.2×10^{-136}
CCR3	3:46130890	5.9×10^{-92}	974	3:46081551:A:G	1.2×10^{-124}
CCR1	3:46201711	2.5×10^{-44}	416	3:46237659:C:T	5.3×10^{-66}
CCR2	3:46353864	2.7×10^{-46}	411	3:46347209:C:T	1.5×10^{-61}
CCR5	3:46370946	5.3×10^{-45}	422	3:46347209:C:T	1.5×10^{-61}
CCRL2	3:46407166	7.5×10^{-43}	477	3:46366689:C:A	1.7×10^{-46}
LTF	3:46435645	9×10^{-27}	586	3:46422383:G:A	7.1×10^{-34}
RTP3	3:46494611	1.7×10^{-13}	367	3:46444801:G:A	2.1×10^{-25}
LRRC2	3:46515385	9.3×10^{-12}	792	3:46522300:A:G	5.3×10^{-25}
TDGF1	3:46574534	2.3×10^{-12}	548	3:46532185:A:G	1.7×10^{-20}
ENSG00000283877	3:46581150	1.1×10^{-10}	644	3:46532185:A:G	1.7×10^{-20}
FAM240A	3:46612525	2×10^{-7}	530	3:46610496:A:G	2.1×10^{-19}
MECOM	3:169083499	8.3×10^{-9}	2630	3:169079990:C:T	5.8×10^{-8}
P4HA2	5:132190147	5.2×10^{-7}	684	5:132282906:C:T	2.4×10^{-6}
SLC22A4	5:132294394	1.3×10^{-6}	591	5:132282906:C:T	2.4×10^{-6}
SLC22A5	5:132369710	2.8×10^{-7}	465	5:132422622:A:G	2.7×10^{-10}
C6orf15	6:31111223	4.8×10^{-10}	1342	6:31153604:G:C	9.8×10^{-27}
PSORS1C1	6:31114750	2.1×10^{-11}	1681	6:31153604:G:C	9.8×10^{-27}
CDSN	6:31115087	2.3×10^{-10}	1379	6:31153604:G:C	9.8×10^{-27}

Gene Symbol	Chr:pos$_{start}$ (b38)	P$_{gene}$	N$_{snps}$	Lead SNP	P$_{Lead SNP}$
PSORS1C2	6:31137534	2×10^{-12}	1419	6:31153604:G:C	9.8×10^{-27}
CCHCR1	6:31142439	4×10^{-12}	1713	6:31153604:G:C	9.8×10^{-27}
TCF19	6:31158547	5.4×10^{-12}	1636	6:31153604:G:C	9.8×10^{-27}
POU5F1	6:31164337	2.4×10^{-11}	1759	6:31153604:G:C	9.8×10^{-27}
HLA-C	6:31268749	6.5×10^{-9}	2593	6:31276554:C:T	6×10^{-15}
HLA-B	6:31353872	1.8×10^{-8}	2952	6:31306250:C:T	2.3×10^{-13}
MICA	6:31399784	9.8×10^{-10}	2400	6:31461190:G:A	1.7×10^{-14}
MICB	6:31494881	2×10^{-10}	1314	6:31461190:G:A	1.7×10^{-14}
MCCD1	6:31528962	7.7×10^{-9}	955	6:31539985:CCT:C	1.2×10^{-12}
DDX39B	6:31530219	3.4×10^{-9}	1000	6:31539985:CCT:C	1.2×10^{-12}
ATP6V1G2-DDX39B	6:31530219	2.5×10^{-9}	1032	6:31539985:CCT:C	1.2×10^{-12}
ATP6V1G2	6:31544444	2×10^{-10}	852	6:31539985:CCT:C	1.2×10^{-12}
NFKBIL1	6:31546870	1.6×10^{-10}	860	6:31539985:CCT:C	1.2×10^{-12}
LTA	6:31572054	1.8×10^{-10}	628	6:31539985:CCT:C	1.2×10^{-12}
TNF	6:31575565	2.1×10^{-10}	610	6:31539985:CCT:C	1.2×10^{-12}
LTB	6:31580525	4.9×10^{-11}	592	6:31539985:CCT:C	1.2×10^{-12}
LST1	6:31586124	3.8×10^{-11}	600	6:31539985:CCT:C	1.2×10^{-12}
NCR3	6:31588895	7.5×10^{-11}	593	6:31539985:CCT:C	1.2×10^{-12}
AIF1	6:31615217	1.3×10^{-9}	514	6:31571991:A:G	3.1×10^{-10}
PRRC2A	6:31620715	1×10^{-9}	559	6:31571991:A:G	3.1×10^{-10}
ENSG00000289282	6:31623874	5.9×10^{-9}	508	6:31587353:A:G	3.6×10^{-9}
BAG6	6:31639028	1.6×10^{-8}	524	6:31593434:T:C	8.3×10^{-9}
APOM	6:31652416	8×10^{-8}	450	6:31638615:G:A	1.9×10^{-7}
C6orf47	6:31658298	1.2×10^{-6}	427	6:31638615:G:A	1.9×10^{-7}
GPANK1	6:31661228	1×10^{-6}	437	6:31638615:G:A	1.9×10^{-7}
CSNK2B	6:31665227	1.7×10^{-6}	437	6:31638615:G:A	1.9×10^{-7}
ENSG00000263020	6:31666102	1.1×10^{-6}	439	6:31638615:G:A	1.9×10^{-7}
LY6G5B	6:31670167	5.4×10^{-7}	417	6:31638615:G:A	1.9×10^{-7}
LY6G5C	6:31676684	3.2×10^{-8}	416	6:31638615:G:A	1.9×10^{-7}
ENSG00000204422	6:31686962	1.7×10^{-6}	474	6:31638615:G:A	1.9×10^{-7}
LY6G6F	6:31706866	4.7×10^{-7}	378	6:31720741:T:A	2.7×10^{-6}
LY6G6F-LY6G6D	6:31706904	5.3×10^{-7}	407	6:31720741:T:A	2.7×10^{-6}
MSH5	6:31739677	9.9×10^{-7}	433	6:31720741:T:A	2.7×10^{-6}
MSH5-SAPCD1	6:31740020	7.1×10^{-7}	438	6:31720741:T:A	2.7×10^{-6}
SAPCD1	6:31762996	3.5×10^{-7}	366	6:31720741:T:A	2.7×10^{-6}
VWA7	6:31765590	8×10^{-7}	387	6:31720741:T:A	2.7×10^{-6}
VARS1	6:31777518	1.4×10^{-6}	434	6:31842089:T:G	9.8×10^{-6}
LSM2	6:31797396	1.9×10^{-6}	405	6:31842089:T:G	9.8×10^{-6}
NEU1	6:31857659	1.1×10^{-6}	420	6:31872678:A:G	3.4×10^{-6}
SLC44A4	6:31863192	1.2×10^{-6}	449	6:31872678:A:G	3.4×10^{-6}
EHMT2	6:31879759	2.6×10^{-7}	465	6:31872678:A:G	3.4×10^{-6}
C2	6:31897785	5.3×10^{-7}	515	6:31872678:A:G	3.4×10^{-6}
ZBTB12	6:31899613	3.1×10^{-7}	392	6:31872678:A:G	3.4×10^{-6}
ENSG00000244255	6:31927698	2.9×10^{-7}	385	6:31926167:G:A	5.1×10^{-6}
CFB	6:31945650	1.9×10^{-6}	309	6:31926167:G:A	5.1×10^{-6}
NELFE	6:31952087	2.1×10^{-6}	288	6:31926167:G:A	5.1×10^{-6}
SKIV2L	6:31959117	1.4×10^{-6}	266	6:31926167:G:A	5.1×10^{-6}
DXO	6:31969810	1.8×10^{-6}	224	6:31926167:G:A	5.1×10^{-6}
ZKSCAN1	7:100015572	2.4×10^{-6}	267	7:100032719:C:T	8.2×10^{-11}
ABO	9:133233278	1×10^{-6}	968	9:133271182:T:C	1.2×10^{-16}
ABTB2	11:34150987	1.4×10^{-6}	1159	11:34396323:T:C	2.4×10^{-12}
CAT	11:34438934	1.9×10^{-12}	684	11:34482745:G:A	6.9×10^{-23}
ELF5	11:34478791	5.3×10^{-12}	530	11:34482745:G:A	6.9×10^{-23}
OAS1	12:112905856	2.2×10^{-7}	423	12:112919637:G:A	2.2×10^{-15}
OAS3	12:112938051	2.8×10^{-7}	537	12:112919637:G:A	2.2×10^{-15}
FBRSL1	12:132489551	8.7×10^{-9}	1006	12:132481571:G:A	2.2×10^{-16}
ATP11A	13:112690038	3.2×10^{-7}	1191	13:112881427:C:T	7.8×10^{-22}
ACSF3	16:89088375	1.8×10^{-9}	935	16:89196249:G:A	3×10^{-16}
CDH15	16:89171748	6.3×10^{-9}	663	16:89196249:G:A	3×10^{-16}
SLC22A31	16:89195761	1.3×10^{-7}	579	16:89196249:G:A	3×10^{-16}
ZNF778	16:89217703	1.5×10^{-7}	588	16:89196249:G:A	3×10^{-16}
LRRC3C	17:39927732	9.8×10^{-7}	439	17:39989575:A:G	1.5×10^{-9}
GSDMA	17:39953263	3.2×10^{-7}	521	17:40003082:T:C	1.1×10^{-9}
PSMD3	17:39980807	4.4×10^{-7}	477	17:40003082:T:C	1.1×10^{-9}
CSF3	17:40015361	5.5×10^{-7}	370	17:40003082:T:C	1.1×10^{-9}
MED24	17:40019097	1.5×10^{-6}	442	17:40003082:T:C	1.1×10^{-9}
ARHGAP27	17:45393902	2.4×10^{-6}	422	17:45473955:G:C	1.5×10^{-11}
PLEKHM1	17:45435900	1.1×10^{-6}	339	17:45495053:C:T	8.8×10^{-15}
LINC02210-CRHR1	17:45620344	1.2×10^{-10}	1573	17:45826476:C:T	2.1×10^{-15}
CRHR1	17:45784280	6×10^{-11}	928	17:45826476:C:T	2.1×10^{-15}
SPPL2C	17:45844881	9.4×10^{-12}	665	17:45826476:C:T	2.1×10^{-15}
MAPT	17:45894527	5×10^{-11}	1497	17:46048999:C:T	2.6×10^{-16}
STH	17:45999250	5.3×10^{-10}	605	17:46048999:C:T	2.6×10^{-16}
KANSL1	17:46029916	7.8×10^{-8}	1398	17:46085231:C:A	1.8×10^{-16}
NSF	17:46590669	9.4×10^{-8}	219	17:46748690:C:G	1×10^{-14}
WNT3	17:46762506	2.2×10^{-6}	406	17:46748690:C:G	1×10^{-14}
KAT7	17:49788648	2.4×10^{-8}	266	17:49863303:C:T	8.6×10^{-16}
TAC4	17:49838300	8.1×10^{-7}	261	17:49863303:C:T	8.6×10^{-16}
SEMA6B	19:4542593	4.5×10^{-7}	582	19:4617960:C:T	4×10^{-12}
TNFAIP8L1	19:4639516	8.2×10^{-12}	480	19:4686976:G:T	8×10^{-24}
MYDGF	19:4641374	1.9×10^{-21}	522	19:4717660:A:G	8.9×10^{-51}
DPP9	19:4675224	1.8×10^{-24}	574	19:4717660:A:G	8.9×10^{-51}
DNMT1	19:10133342	1.9×10^{-6}	526	19:10271861:G:A	7.2×10^{-7}
MRPL4	19:10251901	8.3×10^{-16}	322	19:10305768:G:A	8×10^{-17}

Gene Symbol	Chr:pos_{start} (b38)	P_{gene}	N_{snps}	Lead SNP	$P_{Lead\,SNP}$
ICAM1	19:10271093	1.3×10^{-18}	363	19:10305768:G:A	8×10^{-17}
ICAM4	19:10286955	3.1×10^{-20}	339	19:10305768:G:A	8×10^{-17}
ICAM5	19:10289952	4.7×10^{-19}	361	19:10305768:G:A	8×10^{-17}
ZGLP1	19:10304803	6.2×10^{-24}	355	19:10352442:G:C	2.1×10^{-28}
ENSG00000167807	19:10305427	1.6×10^{-24}	373	19:10352442:G:C	2.1×10^{-28}
FDX2	19:10310045	3.6×10^{-25}	359	19:10352442:G:C	2.1×10^{-28}
ENSG00000267303	19:10315471	2.4×10^{-26}	363	19:10352442:G:C	2.1×10^{-28}
RAVER1	19:10316212	4×10^{-27}	407	19:10352442:G:C	2.1×10^{-28}
ICAM3	19:10333776	1.2×10^{-27}	369	19:10352442:G:C	2.1×10^{-28}
TYK2	19:10350529	4.8×10^{-30}	392	19:10352442:G:C	2.1×10^{-28}
CDC37	19:10391133	2.6×10^{-32}	331	19:10352442:G:C	2.1×10^{-28}
PDE4A	19:10416773	1.1×10^{-24}	414	19:10381598:C:T	4.4×10^{-28}
KEAP1	19:10486125	1.9×10^{-15}	370	19:10486312:C:A	3.7×10^{-20}
S1PR5	19:10512742	6.6×10^{-10}	371	19:10486312:C:A	3.7×10^{-20}
ATG4D	19:10543895	8.5×10^{-7}	435	19:10523588:C:T	1.9×10^{-9}
KRI1	19:10553085	4.7×10^{-7}	457	19:10523588:C:T	1.9×10^{-9}
CDKN2D	19:10566460	2.9×10^{-7}	423	19:10523588:C:T	1.9×10^{-9}
SLC44A2	19:10602457	9.3×10^{-7}	474	19:10583892:G:A	3.6×10^{-9}
NTN5	19:48661407	2.8×10^{-8}	319	19:48702915:C:T	1.2×10^{-15}
FUT2	19:48695971	1.5×10^{-9}	339	19:48702915:C:T	1.2×10^{-15}
MAMSTR	19:48712725	6.4×10^{-9}	361	19:48702915:C:T	1.2×10^{-15}
RASIP1	19:48720585	5.1×10^{-9}	411	19:48702915:C:T	1.2×10^{-15}
IZUMO1	19:48740852	6.4×10^{-9}	385	19:48702915:C:T	1.2×10^{-15}
FUT1	19:48748011	4.9×10^{-9}	402	19:48702915:C:T	1.2×10^{-15}
FGF21	19:48755524	3.8×10^{-8}	368	19:48705608:G:A	6×10^{-15}
BCAT2	19:48795062	1.9×10^{-6}	460	19:48745473:G:A	9×10^{-12}
IFNAR2	21:33205282	1.8×10^{-31}	711	21:33237639:A:G	6.2×10^{-48}
ENSG00000249624	21:33229892	3.1×10^{-34}	721	21:33237639:A:G	6.2×10^{-48}
IL10RB	21:33266367	7.8×10^{-34}	517	21:33237639:A:G	6.2×10^{-48}
IFNAR1	21:33324429	1.7×10^{-8}	459	21:33287378:C:T	7.2×10^{-15}
ENSG00000249209	21:33584687	2.1×10^{-7}	1067	21:33949755:C:T	6.4×10^{-17}
ITSN1	21:33642400	9.5×10^{-7}	872	21:33949755:C:T	6.4×10^{-17}
ATP5PO	21:33903453	1.2×10^{-7}	425	21:33949755:C:T	6.4×10^{-17}

3 Sex Stratified GWAS

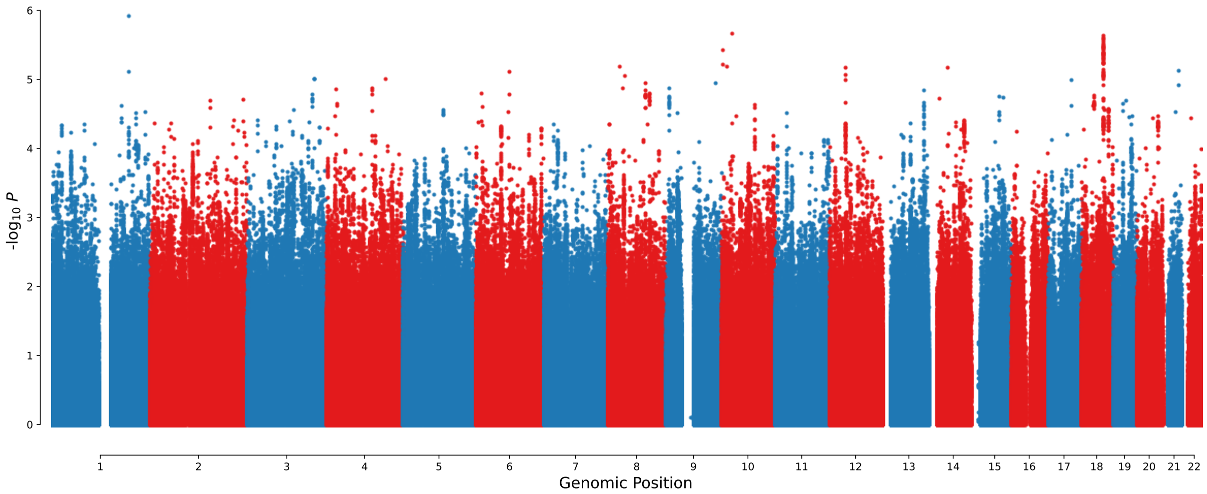

Supplementary Figure 1: Manhattan plot for tests for differences in effects between sexes at each genetic variant. We tested for differences between effects of meta analyses of sex specific GWAS from GenOMICC and SCOURGE.

4 TWAS

Supplementary Table 11: Selected TWAS results for lung, whole blood, monocytes, and metaTWAS with $P_{meta} < 1\times10^{-5}$ (See Supplementary Table 2 for full results). Gene: Gene symbol; Z_{lung}: TWAS Z-score in lung; Z_{blood}: TWAS Z-score in blood; Z_{mono}: TWAS Z-score in monocytes; Z_{meta}: metaTWAS mean Z-score; Z_{SD}: metaTWAS Z-score standard deviation, n_{meta}: number of SNPs included in metaTWAS; P_{meta}: metaTWAS P-value.

Gene	Z_{lung}	Z_{blood}	Z_{mono}	Z_{meta}	Z_{SD}	n_{meta}	P_{meta}
FYCO1	8.9	3.9		4.2	4.2	47	9.5×10^{-46}
IL10RB	8.9	-3.1		7.4	4	46	2.3×10^{-34}
FOXP4	8.7			0.12	2	28	1×10^{-15}
ATP5O	7	2.8		3.7	1.7	47	3.2×10^{-13}
FUT2	6.6			0.37	5.4	28	4.7×10^{-13}
TCF19	6.1	4.8		4.7	2.9	49	1.5×10^{-12}
GSDMA	5.9	0.22	5.8	3.8	2.2	38	1.7×10^{-6}
TYK2	5.8	7.7		2.7	3.8	45	9.5×10^{-18}
ELF5	5.8			3.7	1.7	9	2.5×10^{-8}
SLC6A20	5.7			9.3	6.3	33	3.4×10^{-229}
MUC1	5.5	7.1		5	2.9	42	2.2×10^{-25}
ABO	5.1	-1.5	-6.7	3.7	1.9	48	4.7×10^{-7}
RAB2A	5	3.7	4.9	4.7	0.84	45	1.3×10^{-6}
TOMM7	4.7	3.4	4.6	4.2	0.85	49	2.3×10^{-6}
HLA-C	4.5	6.3		2.8	2.6	48	2.6×10^{-15}
RP11-387H17.6	4.4			2.6	3.1	5	5.3×10^{-6}
CAT	4.2	2.9		2.9	2.2	49	9.1×10^{-14}
ZNF778	4.1	0.19		0.76	2.2	49	6.4×10^{-7}
CDH15	4.1			1.9	2.9	34	2.2×10^{-7}
HIP1	4.1	-5.8		0.94	1.7	42	1.8×10^{-7}
ACSF3	3.8	1.1	4.7	1.8	2	48	1.4×10^{-9}
CCHCR1	3.7	3.9		3.3	2.3	49	9.7×10^{-14}
FBRSL1	3.4	1.9		0.87	3.5	45	5.8×10^{-10}
ACSL6	3	4.6		-0.38	3.6	44	4.8×10^{-7}
CCR1	3	-1.5		3.4	7.6	47	3.9×10^{-53}
HLA-DPA1	3	1.1		-0.81	1.5	37	1.1×10^{-6}
RP11-11C20.3	3			3.2	0.51	32	9.6×10^{-6}
MTX1	3	-0.85		1.1	3.9	36	2.9×10^{-30}
IRF7	2.7	0.075		-1.7	1.9	44	9.6×10^{-7}
HLA-A	2.7	-0.96		1.4	2.3	46	8.4×10^{-6}
FAM215B	2.7			-1.2	4	7	5.7×10^{-11}
HLA-F	2.6	2.8		1.1	1.9	45	6.7×10^{-6}
FUT1	2.1			2.8	2.5	27	2.6×10^{-7}
SLC50A1	2.1	1.6		0.7	1.8	49	3×10^{-15}
FAM83H	2.1	-0.89		0.99	1.4	48	2.3×10^{-7}
DDX39B	1.8	3.3		0.25	2.4	41	7.1×10^{-13}
FDX2	1.8	-1.2		-2.8	2.4	48	1.3×10^{-12}
CCR2	1.7	0.88	-10	6	7.8	35	4.6×10^{-44}
HLA-DRA	1.5			0.89	1.9	31	2.6×10^{-6}
SACM1L	1.4	1		1.9	2.7	44	5.7×10^{-10}
SEMA6B	0.7	2.1		0.23	2	26	6.7×10^{-6}
PSMD3	0.47	0.48	4.7	0.23	2.1	40	2.3×10^{-8}
OAS3	0.24	4		0.32	4.2	37	4.2×10^{-9}
SFTPD	0.17			3	1.9	47	1.5×10^{-6}
BTNL2	0.1			-0.5	2.4	21	2.4×10^{-7}
LZTFL1	-0.071	-2.6		-2.6	8	45	2.5×10^{-119}
MCM6	-0.094			0.79	2.3	44	3.9×10^{-6}
PSORS1C1	-0.28			-0.61	2.3	48	1.2×10^{-9}
SLC5A3	-0.3			0.22	2.1	21	3.7×10^{-7}
SLC35B1	-0.31	-0.31		1.4	3	46	3.5×10^{-11}
NAPSA	-0.56	-6.8		-6.2	1.9	39	7.1×10^{-11}
LRRC37A2	-0.59		-5.9	-0.15	3.6	6	3.4×10^{-13}
KCNC3	-0.73		-6.2	2.1	1.9	45	4.1×10^{-6}
C5orf56	-0.92	-2.5		-1.7	1.9	38	1.6×10^{-10}
MED24	-0.95	5	5.3	2.4	3.4	46	1.3×10^{-7}
MICB	-1	-1.8		-0.86	1.5	49	2.4×10^{-10}
LTF	-1.1			-0.93	2.2	37	2.9×10^{-16}
RIT1	-1.2	-4.2		-2.3	1.4	48	3.7×10^{-7}
DCST2	-1.2	-3.3		-0.88	1.6	49	7.2×10^{-11}
DPP9	-1.2	-5.5		-3.8	4	45	9.9×10^{-41}
ATP11A	-1.2	-3.5		-1.7	3.2	42	2.8×10^{-11}
PMVK	-1.5	-0.9		-1.4	2.4	48	8.9×10^{-6}
MAMSTR	-1.7	5.7		-1.9	3.6	45	4×10^{-9}
ZKSCAN1	-1.9	-1.9		-1.1	2.1	43	1.6×10^{-6}
DDAH2	-2.1	2.7		-0.25	2.4	44	1.9×10^{-6}
TNFAIP8L1	-2.2	-1.5		-1	2.1	36	1.7×10^{-8}
XCR1	-2.4	5.8	7.7	-0.49	6.1	23	9.9×10^{-117}
XXbac-BPG181B23.7	-2.5	-4.2		-1.1	1.6	38	1.8×10^{-8}
EFNA1	-2.5	-6.6		0.044	2.6	48	2.3×10^{-7}
IZUMO1	-2.6			-2.2	2.1	43	2.8×10^{-7}
CCRL2	-2.9			2.8	6	30	2.8×10^{-33}
PDE4A	-3.2	-0.49	4.6	-2.7	3	47	1×10^{-9}
ICAM3	-3.2	-2		-1.5	1.7	34	3.3×10^{-9}
RAVER1	-3.4	6.1		-0.53	2.2	45	9.1×10^{-9}
NXPE3	-3.5	-4.4		-2.3	2	47	9.6×10^{-7}

Gene	Z_{lung}	Z_{blood}	Z_{mono}	Z_{meta}	Z_{SD}	n_{meta}	P_{meta}
NPNT	-3.9	-0.48		1.6	2.6	48	1.5×10^{-8}
ZGLP1	-4	4		-4	2.6	45	6.3×10^{-14}
ICAM5	-4.1	1.7	-6.4	-3.6	3.5	47	4.2×10^{-10}
PSORS1C2	-4.2			-2.5	1.9	39	1.6×10^{-12}
IFNAR1	-4.4	-2.4		-2.4	2.4	49	3.9×10^{-6}
OAS1	-4.7	-3.3		-2.9	1.8	41	2.7×10^{-9}
STK19	-4.9	-3.3		-3.2	1.1	43	2.7×10^{-6}
NTN5	-5	3.6		-3.7	3.2	47	4.3×10^{-10}
LINC01301	-5			-4.3	1.1	38	5.6×10^{-6}
JAK1	-5.3	1.3		-1.7	3.4	47	1.8×10^{-6}
RASIP1	-5.3	-5.3		-4.8	1.9	32	2.9×10^{-8}
IFNAR2	-5.3	-3.8		-4.4	6.9	48	5×10^{-51}
CEP97	-5.5	0.36		-1.7	2.8	17	8.2×10^{-7}
CSF3	-5.7			-4	2	38	4.7×10^{-7}
CCR3	-6.1	-1.8	-6.9	-9	5.9	6	2.4×10^{-64}
ADAM15	-6.4	3		2.4	2.1	46	2.3×10^{-15}
ARHGAP27	-6.4	3.6		0.11	5.1	44	2.4×10^{-9}
WNT3	-7.6	-7		-4.7	2.3	47	1.3×10^{-10}
CCR5	-16	2.4	-6.9	-3.5	3.7	46	1.5×10^{-51}
CXCR6	-21		-6.9	-8	9.6	38	1.1×10^{-134}
CCR9		31		13	11	7	2.6×10^{-223}
HLA-B		2.8		-0.36	2.2	44	2×10^{-8}
ICAM1		2.7		2.6	1.2	7	6.6×10^{-6}
GPC2		2.7		-0.87	1.8	43	5.1×10^{-6}
LINC02009		2.5		1.7	3.8	35	5.2×10^{-16}
FNIP1		1.4		-1	2.4	11	1.1×10^{-6}
MRPS6		0.97		-1.2	2.5	13	3.4×10^{-10}
ZBTB7B		0.59		1.4	1.8	23	2.7×10^{-6}
PBXIP1		-0.95		0.58	1.3	16	1.8×10^{-7}
LTA		-1.5		-2.4	3.2	38	7.4×10^{-8}
TRIM46				5.8	5.1	18	5.4×10^{-22}
CDC37				4.6	0.95	27	1.2×10^{-6}
ZBTB11				2.5	3.9	33	1.4×10^{-6}
CFB				1.9	1.2	31	5.5×10^{-8}
ASH1L				1.9	2	4	4×10^{-22}
EFNA4				1.8	4	6	3.1×10^{-12}
HLA-DRB5				1.4	2.4	5	3.1×10^{-8}
NFKBIZ				0.94	4.7	10	5.6×10^{-6}
LINC01377				0.45	1.7	5	1.7×10^{-7}
CACNG8				0.28	1.7	16	8.7×10^{-7}
GON4L				-0.53	4.7	32	4.5×10^{-14}
ANKRD45				-0.89	1.4	21	4.1×10^{-6}
RTP3				-1.3	2.1	5	8.4×10^{-7}
C6orf15				-1.5	4	6	6.4×10^{-14}
KRTCAP2				-1.8	3	4	5.9×10^{-10}
LINC01149				-1.9	4.5	2	2.7×10^{-6}
PLEKHM1				-1.9	3.1	6	1.2×10^{-17}
AP000569.9				-2.1	2.4	3	9.5×10^{-13}
CDSN				-2.4	1.6	2	8.5×10^{-6}
RP4-535B20.1				-2.7	2.1	5	8.2×10^{-6}
RP11-798G7.6				-3.4	5.4	3	2.3×10^{-10}

5 Drugs in consideration by UK-CTAP

Supplementary Table 12: Drugs under consideration for use in severe Covid-19 by the independent UK COVID-19 Therapeutics Advisory Panel (UK-CTAP). Genes with potential relevance to the assessment of each drug were identified by a UK-CTAP investigator (GM) without prior knowledge of the GenOMICC results. Significant P-values in TWAS, GSMR (RNA), and Gene-level GWAS are shown.

Gene Symbol	Target drug	Taget pathway	In trial	$P_{metaTWAS}$	$P_{GSMR-RNA}$	$P_{Gene-level}$
NLRP3	Colchicine	Inflammasome	✓			
NLRP1	Colchicine	Inflammasome	✓			
NLRC5	Colchicine	Inflammasome	✓			
AIM2	Colchicine	Inflammasome	✓			
C5	Nomacopan, Zilucoplan	Inflammatory response and terminal complement pathway	✓			
CSF1	Edicotinib, Cabiralizumab	A macrophage stimulating factor	✓			
F10	Apixaban	Prevents thrombin generation	✓			
FGA	Alteplase	Thrombolytic	✓			
FGB	Alteplase	Thrombolytic	✓			
FGG	Alteplase	Thrombolytic	✓			
CSF2	Namilumab	Monocyte/macrophage stimulating factor	✓			
IFN	PEG-IFN beta and lambda	innate immune response	✓			
ERN1	Fluvoxamine	Inflammation	✓			
SIGMAR1	Fluvoxamine	Inflammation - sigma-1 receptor	✓			
TMEM97	Fluvoxamine	Inflammation - sigma-2 receptor	✓			
SLC5A2	Gliflozin	Sodium-glucose co-transporter-2	✓			
JAK1	Baricitinib, nezulcitinib	Cytokine and interferon activation	✓	1.8e-06		3.4e-06
STAT1	Baricitinib		✓		0.0004	
STAT2	Baricitinib		✓			
TYK2	Nezulcitinib, baricitinib	IL-6R and type I interferon (IFN) signalling		9.5e-18	1.2e-23	4.8e-30
ANO1	Niclosamide	Calcium-activated ion channel involved in viral-induced syncytia	✓			
TMPRSS2	Camostat, Nafamostat	Core viral entry mechanism	✓			4.4e-06
TNF	Adalimumab		✓		2.5e-07	2.1e-10
TRPM2	Artesunate	Neutrophil activation	✓			
CSNK2A1	Silmitasertib	Casein kinase 2 in inflammation	✓			
CSNK2A2	Silmitasertib	Casein kinase 2 in inflammation	✓			
CSNK2B	Silmitasertib	Casein kinase 2 in inflammation	✓		0.00017	1.7e-06
ACE2	Ursodeoxycholic acid, recombinant ACE2	Core viral entry mechanism				
ATP2A1	Thapsigargin	SERCA pump				
ATP2A2	Thapsigargin	SERCA pump				
ATP2A3	Thapsigargin	SERCA pump				
AXL	Bemcentinib	Cell surface receptor for viral entry				
CSF1R	Edicotinib, Cabiralizumab	Macrophage activation				
CTSL	SLV213	Core viral entry mechanism - cysteine protease cathepsin L				
DHFR	Pralatrexate, methotrexate	Folate pathway				
DHODH	Lefluonamide, IMU-838					
GBA	Ambroxol	Glucosylceramidase to lower glycosphingolipids				4.2e-30
GBA2	Ambroxol	Glucosylceramidase to lower glycosphingolipids				
GBA3	Ambroxol	Glucosylceramidase to lower glycosphingolipids				
GPX1	Ebselen	Glutathione peroxidase induced oxidant stress				
GPX2	Ebselen	Glutathione peroxidase induced oxidant stress				
GPX3	Ebselen	Glutathione peroxidase induced oxidant stress				
GPX4	Ebselen	Glutathione peroxidase induced oxidant stress				
GPX5	Ebselen	Glutathione peroxidase induced oxidant stress				
GPX6	Ebselen	Glutathione peroxidase induced oxidant stress				
GPX7	Ebselen	Glutathione peroxidase induced oxidant stress				
HIF1A	2-deoxy-D-glucose	glucose metabolism - hypoxia-inducible factor-1α				
HRH2	Famotidine	Histamine response in inflammation				
HTR1A	Cyproheptadine	Serotonin and histamine receptor binding				
HTR2A	Cyproheptadine	Serotonin and histamine receptor binding				
HTR3A	Cyproheptadine	Serotonin and histamine receptor binding				
HTR2C	Cyproheptadine	Serotonin and histamine receptor binding				

Gene Symbol	Target drug	Taget pathway	In trial	$P_{metaTWAS}$	$P_{GSMR-RNA}$	$P_{Gene-level}$
IL1a	Anakinra	Inflammatory cytokine				
IL1b	Anakinra	Inflammatory cytokine				
IL5RA	Fasenra	Inflammation				
KEAP1	Ebselen	Oxidant stress regulation				1.9e-15
LDHA	2-deoxy-D-glucose	Glucose metabolism				
IFIH1	Hiltonol	Innate immune response				
MTOR	Metformin	Glucose regulation				
NFKB1	Metformin, APPA	Immune regulation				
NFKB2	Metformin, APPA	Immune regulation				
NOS2	NO	Antiviral action of NO				
NFE2L2	APPA	Oxidant resistance				
NUP genes	Ivermectin	Nuclear pore complex				
OAS1	Hiltonol	Innate immune response		2.7e-09	7e-13	2.2e-07
OAS2	Hiltonol	Innate immune response			0.00042	
OAS3	Hiltonol	Innate immune response		4.2e-09		2.9e-07
SLC22A8	Probenecid	Organic anion transporter				
PIKFYVE	Apilimod	Phosphoinositide Kinase				
SPHK2	Opaganib	Sphingosine kinase 2 in inflammation				
SOD3	N-acetylcysteine	Oxidant stress regulation				
TGM2	Nylexa	Transglutaminase-2 regulating inflammation				
VIP	Aviptadil	Bronchodilator				

6 Description of cohorts

6.1 Critical and hospitalisation meta-analysis cohorts

Supplementary Table 13: Description of cohorts included in the critical meta-analysis with ancestry information, median age, and percentage of females for the cases. Complete details of all cohorts can be found in Supplementary Table 1. "Critical" refers to the initiation of continuous cardiorespiratory monitoring, which in adults is undertaken in intensive care units (ICU) or high dependency units (HDU) in most health care systems, or admission to ICU/HDU. This is the definition used by the GenOMICC study since 2015 and because of this, was included by HGI as the "A2" phenotype for use in registry studies. In the SCOURGE study the "severe" cohort corresponds to levels 3-4 in the SCOURGE severity grading system.[2] WGS: whole-genome sequencing. NA: information not available for this cohort. **data was available for the hospitalisation cohort but not for the critically-ill patients.

Study	Dataset	Ancestry	Case definition	Control definition	Median age(IQR)	Female(%)	N cases	N controls
GenOMICC	WGS	EUR	Critical	Population/ mild	60(11.87)	32.17%	5989	42981
GenOMICC	WGS	EAS	Critical	Population/ mild	54(11.27)	40.87%	274	366
GenOMICC	WGS	SAS	Critical	Population/ mild	57(13.22)	25.63%	788	3793
GenOMICC	WGS	AFR	Critical	Population/ mild	57(13.06)	35%	440	1350
GenOMICC	Array	EUR	Critical	Population	58(13.86)	32.5%	3029	15215
GenOMICC	Array	EAS	Critical	Population	53(12.09)	37.5%	112	323
GenOMICC	Array	SAS	Critical	Population	58(16.10)	32.5%	324	1451
GenOMICC	Array	AFR	Critical	Population	55(16.47)	43.1%	218	1090
ISARIC4C	Array	EUR	ICU/ CPAP/ NIV	Population	63(14.92)	30%	184	920
ISARIC4C	Array	EAS	ICU/ CPAP/ NIV	Population	51(9.92)	26.67%	15	70
ISARIC4C	Array	SAS	ICU/ CPAP/ NIV	Population	59(17.79)	41.3%	29	140
ISARIC4C	Array	AFR	ICU/ CPAP/ NIV	Population	58(14.98)	25%	24	100
HGiv6	Array	ALL	Critical	Population			8779	1001875
SCOURGE	Array	EUR	Severe	Population	70(23)	27.5%	3502	5455
23andMe	Array	EUR	Respiratory Support	Population	53(17.5)**	50.7% **	495	680440
ALL_studies							24202	1755569

Supplementary Table 14: Cohorts including patients hospitalised with Covid-19. Description of cohorts included in the critical meta-analysis with ancestry information, median age and percentage of females for the cases. NA: information not available for this cohort.

Study	Dataset	Ancestry	Case definition	Control definition	Median age(IQR)	Female(%)	N cases	N controls
GenOMICC	WGS	EUR	Critical	Population/ mild	60(11.87)	32.17%	5989	42981
GenOMICC	WGS	EAS	Critical	Population/ mild	54(11.27)	40.87%	274	366

Study	Dataset	Ancestry	Case definition	Control definition	Median age(IQR)	Female(%)	N cases	N controls
GenOMICC	WGS	SAS	Critical	Population/ mild	57(13.22)	25.63%	788	3793
GenOMICC	WGS	AFR	Critical	Population/ mild	57(13.06)	35%	440	1350
GenOMICC	Array	EUR	Critical	Population	58(13.86)	32.5%	3029	15145
GenOMICC	Array	EAS	Critical	Population	53(12.09)	37.5%	112	323
GenOMICC	Array	SAS	Critical	Population	58(16.10)	32.5%	324	1451
GenOMICC	Array	AFR	Critical	Population	55(16.47)	43.1%	218	1090
GenOMICC Saudi Arabia	Array	ADMIX	Hospitalised	Mild cases	61(16.88)	33.3%	93	645
GenOMICC Brazil	Array	ALL	Hospitalised	Mild cases			3533	1700
ISARIC4C	Array	EUR	Hospitalised	Population	64(16.69)	37.81%	548	2740
ISARIC4C	Array	EAS	Hospitalised	Population	48(9.67)	39.28%	28	127
ISARIC4C	Array	SAS	Hospitalised	Population	57(16.65)	35.55	%	59
ISARIC4C	Array	AFR	Hospitalised	Population	57(14.74)	36.58%	41	28
HGiv6	Array	ALL	Hospitalised	Population			22637	2052436
SCOURGE	Array	EUR	Hospitalised	Population	70.66(22.1)	27.5%	5933	8810
23andMe	Array	EUR	Respiratory Support or pneumonia	Population			1128	679531
23andMe	Array	AMR	Respiratory Support or pneumonia	Population			218	94318
23andMe	Array	AFR	Respiratory Support or pneumonia	Population			64	22382
ALL							45456	2929461

7 Full GWAS results tables

Supplementary Table 15: Full results for critical covid GWAS. Chr:Pos(b38): chromosome and position on human genome build 38; rsid: lead variant rsid; EA: Effect Allele; EAF: Effect Allele Frequency; Non-EA: Non effect allele; OR: odds ratio; OR_{CI}: odds ratio confidence interval; P: P-value against null hypothesis of odds ratio of one; P_{cond}: P-value in conditional analysis in variants with $P > 5 \times 10^{-8}$; P_{Het}: Heterogeneity P-value Gene: nearest or most plausible nearby gene; Citation: first demonstration of association. GenOMICC^{new} indicates the new findings in the present manuscript.

Chr:pos(b38)	rsid	EA	Non-EA	EAF	OR	OR_{CI}	P	P_{cond}	P_{Het}	N	Nearest Gene	Citation
1:9067157	rs2478868	A	C	0.64	0.92	0.9-0.95	1.5×10^{-10}		0.018	1766590	SLC2A5	GenOMICC^{new}
1:64948270	rs12046291	A	G	0.33	1.1	1.07-1.13	5.1×10^{-11}		0.69	1731600	JAK1	GenOMICC^{new}
1:77501822	rs71658797	A	T	0.12	1.1	1.09-1.18	2.8×10^{-9}		0.25	1759210	AK5	GenOMICC^{new}
1:155066988	rs114301457	T	C	0.0058	2.4	1.81-3.18	1.5×10^{-9}		1	48877	EFNA4	GenOMICC[3]
1:155175305	rs7528026	A	G	0.03	1.3	1.25-1.44	1.5×10^{-15}		0.46	1756890	TRIM46	GenOMICC[3]
1:155197995	rs41264915	A	G	0.89	1.2	1.17-1.26	7.6×10^{-24}		0.73	1765280	THBS3	HGI[4]
1:155278322	rs11264349	A	T	0.73	0.94	0.92-0.97	7.3×10^{-5}	3.9×10^{-13}	0.36	1743390	HCN3	GenOMICC^{new}
2:60480453	rs1123573	A	G	0.63	1.1	1.09-1.15	1×10^{-14}		0.24	1725960	BCL11A	GenOMICC[3]
3:45796521	rs2271616	T	G	0.13	1.2	1.14-1.23	1.1×10^{-16}		0.00013	1016720	SLC6A20	HGI[4]
3:45818159	rs17713054*	A	G	0.1	2	1.96-2.13	7.7×10^{-254}		8×10^{-15}	1764290	LZTFL1	SCGG[5]
3:45873093	rs35482426	CTT	C	0.83	0.53	0.5-0.57	6.1×10^{-91}		0.2	55882	LZTFL1	SCGG[5]
3:101790631	rs11706494	A	T	0.66	1.1	1.05-1.11	9.4×10^{-9}		0.58	1755710	NXPE3	GenOMICC^{new}
3:146522652	rs343314	T	C	0.076	1.2	1.09-1.21	4.6×10^{-8}		0.12	1723090	PLSCR1	GenOMICC[3]
4:25446871	rs7664615	A	G	0.21	1.1	1.07-1.14	1.5×10^{-8}		0.28	1725240	ANAPC4	GenOMICC^{new}
4:105673359	rs72670002	A	G	0.06	1.1	1.09-1.21	4.4×10^{-8}		0.037	1757770	ARHGEF38	GenOMICC^{new}
4:167824478	rs1073165	A	G	0.61	1.1	1.05-1.11	1.1×10^{-9}		0.78	1766650	.	GenOMICC^{new}
5:132422622	rs2269821	A	G	0.14	1.1	1.08-1.16	3×10^{-10}		0.15	1743980	IRF1-AS1	GenOMICC[3]
6:31153455	rs111837807	T	C	0.91	0.8	0.77-0.84	8.6×10^{-26}		2.3×10^{-15}	1766690	CCHCR1	GenOMICC[6]
6:31571991	rs2071590	A	G	0.34	1.1	1.06-1.11	3.1×10^{-10}		0.34	1766590	LTA	GenOMICC^{new}
6:32702687	rs2858305	T	G	0.62	0.93	0.9-0.95	2.1×10^{-9}		0.02	1766220	HLA-DQA1	GenOMICC[3]
6:41522644	rs41435745	C	G	0.077	1.4	1.31-1.51	1.5×10^{-20}		0.45	1725810	FOXP4	HGI[4]
7:75623396	rs1179620	T	C	0.57	0.92	0.9-0.95	2.3×10^{-9}		0.39	1725980	HIP1	GenOMICC^{new}
7:100032719	rs2897075	T	C	0.37	1.1	1.06-1.11	8.9×10^{-11}		0.9	1766100	ZKSCAN1	GenOMICC^{new}
8:60532539	rs13276831	T	C	0.39	1.1	1.05-1.1	1.7×10^{-8}		0.64	1766140	RAB2A	GenOMICC^{new}
9:21206606	rs28368148	C	G	0.99	0.59	0.49-0.7	5.3×10^{-9}		0.31	729815	IFNA10	GenOMICC[3]
9:33425186	rs60840586	G	GTAAC	0.2	1.1	1.07-1.14	9.7×10^{-9}		0.035	936386	AQP3	SCOURGE[2]
9:133271182	rs879055593	T	C	0.26	1.1	1.1-1.16	1×10^{-16}		0.96	1732540	ABO	SCGG[5]
10:79946568	rs721917	A	G	0.57	0.93	0.9-0.95	7.6×10^{-9}		0.07	1743510	SFTPD	HGI[7]
11:1219991	rs35705950	T	G	0.11	0.86	0.82-0.89	3.8×10^{-14}		0.82	1751430	MUC5B	HGI[7]
11:34482745	rs61882275	A	G	0.37	0.88	0.86-0.91	1×10^{-22}		0.54	1765170	ELF5	GenOMICC[3]
12:112919637	rs2660	A	G	0.67	1.1	1.08-1.13	2.8×10^{-15}		0.45	1765230	OAS1	GenOMICC[6]
12:132481571	rs11614702	A	G	0.49	1.1	1.08-1.13	2.1×10^{-16}		0.74	1766600	FBRSL1	GenOMICC[3]
13:112881427	rs12585036	T	C	0.21	1.2	1.12-1.18	9.6×10^{-22}		0.18	1766450	ATP11A	GenOMICC[3]
16:89196249	rs117169628	A	G	0.14	1.2	1.12-1.2	2.6×10^{-16}		0.42	1763760	SLC22A31	GenOMICC[3]
17:40003082	rs12941811	T	C	0.41	0.93	0.91-0.95	1.1×10^{-9}		0.42	1766110	PSMD3	GenOMICC^{new}
17:46085231	rs8080583	A	C	0.22	0.89	0.86-0.91	1.8×10^{-16}		0.4	1762770	KANSL1	[8]
17:49863303	rs77534576	T	C	0.033	1.3	1.24-1.43	8.7×10^{-16}		0.3	1759300	TAC4	[8]
19:4717660	rs12610495	A	G	0.7	0.8	0.77-0.82	9.1×10^{-51}		0.0019	1691560	DPP9	GenOMICC[6]
19:10352442	rs34536443	C	G	0.045	1.5	1.39-1.61	2.2×10^{-28}		0.97	1683680	TYK2	GenOMICC[6]
19:10414696	rs142770866	A	G	0.083	1.2	1.19-1.3	9.4×10^{-21}		0.65	1759600	PDE4A	GenOMICC^{new}
19:48702915	rs516246	T	C	0.47	0.9	0.88-0.93	1.4×10^{-15}		0.4	1752670	FUT2	GenOMICC[3]
19:50374423	rs35463555	A	G	0.32	1.1	1.07-1.13	1.9×10^{-13}		0.44	1086190	NR1H2	HGI[7]
20:6489447	rs2326788	A	G	0.38	0.93	0.9-0.95	1.5×10^{-8}		0.99	1760070	CASC20	GenOMICC^{new}
21:33229937	rs188401375	C	G	0.98	0.74	0.66-0.84	3.1×10^{-6}	4.7×10^{-9}	0.4	734303	IFNAR2	GenOMICC[6]
21:33237639	rs9636867	A	G	0.67	0.83	0.81-0.85	5.2×10^{-48}		0.03	1766650	IFNAR2	GenOMICC[6]
21:33287378	rs8178521	T	C	0.25	1.1	1.1-1.17	6.2×10^{-15}		0.31	1726400	IL10RB	GenOMICC[3]

Chr:pos(b38)	rsid	EA	Non-EA	EAF	OR	OR_{CI}	P	P_{cond}	P_{Het}	N	Nearest Gene	Citation
21:33980963	rs76608815	T	C	0.09	1.2	1.14-1.23	7.4×10^{-17}		0.19	1765700	ATP5PO	GenOMICC[3]
21:41479527	rs915823	A	C	0.79	1.1	1.06-1.13	2.1×10^{-9}		0.95	1767130	TMPRSS2	GenOMICC[new]
X:15523993	rs35697037	A	G	0.55	1	1.03-1.06	6.8×10^{-9}		0.38	1723360	ACE2	HGI[7]

Supplementary Table 16: Conditional analysis for critical illness phenotype. Conditional analysis calculated using EUR reference panel as most of the population from the analysis is from European ancestry. Chr: chromosome; Pos(b38): position on human genome build 38; EA: effect allele; beta: effect; SE: standard error; P: P-value; $beta_{cond}$: beta in conditional analysis; SE_{cond}: standard error in conditional analysis; P_{cond}: P-value in conditional analysis; LD_r: LD between SNP_i and SNP_{i+1} significant in conditional analysis; Gene: nearest or most plausible nearby gene.

Chr	Pos(b38)	rsid	Eff.Allele	Beta	SE	P	$Beta_{cond}$	SE_{cond}	P_{cond}	LD_r	Gene
1	155066988	rs114301457	T	0.87	0.14	1.5e-09	0.89	0.14	7.3e-10	-0.013	EFNA4
1	155175305	rs7528026	A	0.29	0.036	1.6e-15	0.29	0.037	1.1e-15	0.062	TRIM46
1	155197995	rs41264915	A	0.2	0.019	6.6e-24	0.22	0.02	7.1e-28	0.25	THBS3
1	155278322	rs11264349	A	-0.059	0.015	7.3e-05	-0.11	0.015	4e-13	0	HCN3
1	64948270	rs12046291	A	0.094	0.014	4.7e-11	0.094	0.014	4.7e-11	0	JAK1
1	77501822	rs71658797	A	0.12	0.021	2.7e-09	0.12	0.021	2.7e-09	0	AK5
1	9067157	rs2478868	A	-0.081	0.013	1.5e-10	-0.081	0.013	1.5e-10	0	SLC2A5
10	79946568	rs721917	A	-0.076	0.013	8.2e-09	-0.076	0.013	8.2e-09	0	SFTPD
11	1219991	rs35705950	T	-0.15	0.02	3.8e-14	-0.15	0.02	3.8e-14	0	MUC5B
11	34482745	rs61882275	A	-0.12	0.013	6.9e-23	-0.12	0.013	6.9e-23	0	ELF5
12	112919637	rs2660	A	0.1	0.013	2.2e-15	0.1	0.013	2.2e-15	0	OAS1
12	132481571	rs11614702	A	0.1	0.012	2.2e-16	0.1	0.012	2.2e-16	0	FBRSL1
13	112881427	rs12585036	TRUE	0.14	0.015	7.8e-22	0.14	0.015	7.8e-22	0	ATP11A
16	89196249	rs117169628	A	0.15	0.018	3e-16	0.15	0.018	3e-16	0	SLC22A31
17	40003082	rs12941811	T	-0.075	0.012	1.1e-09	-0.075	0.012	9.3e-10	-0.0039	PSMD3
17	46085231	rs8080583	A	-0.12	0.015	1.8e-16	-0.12	0.015	1.2e-16	0.0031	KANSL1
17	49863303	rs77534576	T	0.29	0.036	8.6e-16	0.29	0.036	7.1e-16	0	TAC4
19	10352442	rs34536443	C	0.4	0.036	2.1e-28	0.42	0.037	2.7e-30	-0.064	TYK2
19	10414696	rs142770866	A	0.22	0.023	1e-20	0.23	0.023	6.7e-23	0	PDE4A
19	4717660	rs12610495	A	-0.23	0.015	8.9e-51	-0.23	0.015	9.1e-50	-0.0092	DPP9
19	48702915	rs516246	T	-0.1	0.013	1.2e-15	-0.1	0.013	2.1e-15	-0.0089	FUT2
19	50374423	rs35463555	A	0.097	0.013	2.1e-13	0.096	0.013	3.5e-13	0	NR1H2
2	60480453	rs1123573	A	0.11	0.014	9.2e-15	0.11	0.014	1.2e-14	0	BCL11A
20	6489447	rs2326788	A	-0.076	0.013	1.5e-08	-0.076	0.013	1.5e-08	0	CASC20
21	33229937	rs188401375	C	-0.3	0.064	3.1e-06	-0.37	0.064	4.7e-09	-0.11	IFNAR2
21	33237639	rs9636867	A	-0.19	0.013	6.2e-48	-0.18	0.013	3.2e-44	-0.13	IFNAR2
21	33287378	rs8178521	T	0.12	0.016	7.2e-15	0.11	0.016	6.3e-11	-0.023	IL10RB
21	33980963	rs76608815	T	0.17	0.02	7.9e-17	0.17	0.02	1.8e-16	0.002	ATP5PO
21	41479527	rs915823	A	0.093	0.016	2.3e-09	0.091	0.016	5.9e-09	0	TMPRSS2
3	101790631	rs11706494	A	0.075	0.013	9e-09	0.075	0.013	9e-09	0	NXPE3
3	146522652	rs343314	T	0.14	0.026	4.6e-08	0.14	0.026	4.6e-08	0	PLSCR1
3	45796521	rs2271616	T	0.17	0.02	9.8e-17	0.24	0.02	4.9e-31	-0.094	SLC6A20
3	45818159	rs17713054*	A	0.71	0.021	4.4e-254	0.67	0.023	1.2e-186	-0.72	LZTFL1
3	45873093	rs35482426	CTT	-0.63	0.031	9.4e-91	-0.26	0.034	2.3e-14	0	LZTFL1
4	105673359	rs72670002	A	0.14	0.025	4.5e-08	0.14	0.025	4.5e-08	0	ARHGEF38
4	167824478	rs1073165	A	0.076	0.013	1.1e-09	0.076	0.013	1.1e-09	0	.
4	25446871	rs7664615	A	0.099	0.017	1.5e-08	0.099	0.017	1.5e-08	0	ANAPC4
5	132422622	rs2269821	A	0.11	0.018	2.7e-10	0.11	0.018	2.7e-10	0	IRF1-AS1
6	31153455	rs111837807	T	-0.22	0.021	8.6e-26	-0.26	0.021	2.7e-33	0.12	CCHCR1
6	31571991	rs2071590	A	0.08	0.013	3.1e-10	0.094	0.013	2.1e-13	-0.044	LTA

Chr	Pos(b38)	rsid	Eff.Allele	Beta	SE	P	$Beta_{cond}$	SE_{cond}	P_{cond}	LD_r	Gene
6	32702687	rs2858305	T	-0.076	0.013	2e-09	-0.088	0.013	3.3e-12	-0.007	HLA-DQA1
6	41522644	rs41435745	C	0.34	0.037	1.4e-20	0.34	0.037	2.8e-20	0	FOXP4
7	100032719	rs2897075	TRUE	0.081	0.013	8.2e-11	0.081	0.013	8.3e-11	0	ZKSCAN1
7	75623396	rs1179620	TRUE	-0.082	0.014	2.4e-09	-0.082	0.014	2.4e-09	0	HIP1
8	60532539	rs13276831	TRUE	0.069	0.012	1.9e-08	0.069	0.012	1.9e-08	0	RAB2A
9	133271182	rs879055593	T	0.12	0.015	1.2e-16	0.12	0.015	1.2e-16	0	ABO
9	21206606	rs28368148	C	-0.53	0.09	5.3e-09	-0.53	0.091	5.3e-09	0	IFNA10
9	33425186	rs60840586	G	0.098	0.017	1e-08	0.098	0.017	1e-08	0	AQP3

Supplementary Table 17: Full results for lead variants in severe (hospitalised) covid GWAS. Chr:Pos(b38): chromosome, Chr:Pos(b38): chromosome and position on human genome build 38; rsid: lead variant rsid; EA: effect allele; Non-EA: non-effect allele (the full insertion at rs71288014 has the sequence GCCTTACCCACAGACTCCTTCAC); OR: odds ratio; OR_{CI}: odds ratio confidence interval; P: P-value against null hypothesis of odds ratio of one; P_{cond}: P-value in conditional analysis in variants with $P > 5 \times 10^{-8}$; P_{Het}: Heterogeneity P-value; N: number of individuals in the analysis; Gene: nearest or most plausible nearby gene; Citation: first demonstration of association. GenOMICC^{new} indicates the new findings in the present manuscript.

Chr:pos(b38)	rsid	EA	Non-EA	OR	OR_{CI}	P	P_{cond}	P_{Het}	N	Nearest Gene	Citation
1:64948270	rs12046291	A	G	1.1	1.04-1.08	3.2×10^{-9}		0.39	2818420	JAK1	GenOMICC^{new}
1:77488712	rs12034334	C	G	1.1	1.05-1.1	9.3×10^{-10}		0.24	2964540	AK5	GenOMICC^{new}
1:155066988	rs114301457	T	C	1.7	1.46-2.01	3.7×10^{-11}		0.0051	1557660	EFNA4	GenOMICC[3]
1:155172916	rs41264911	A	G	1.2	1.16-1.28	1×10^{-13}		0.065	2955380	TRIM46	GenOMICC[3]
1:155203736	rs67579710	A	G	0.87	0.85-0.9	1.7×10^{-22}		0.22	2971380	THBS3	HGI[4]
2:60480759	rs7579014	A	G	0.93	0.92-0.95	7×10^{-12}		0.015	2822150	BCL11A	GenOMICC[3]
3:45696221	rs149853133	T	C	0.71	0.64-0.79	3.4×10^{-11}		0.055	2499890	SACM1L	GenOMICC^{new}
3:45796521	rs2271616	T	G	1.1	1.11-1.17	4.8×10^{-20}		7×10^{-6}	2090810	SLC6A20	HGI[4]
3:45804256	rs17078346	A	C	0.72	0.7-0.74	3.9×10^{-103}		4.5×10^{-55}	2234270	SLC6A20	HGI[4]
3:45818159	rs17713054*	A	G	1.7	1.62-1.73	2.8×10^{-239}		3.9×10^{-42}	2956580	LZTFL1	SCGG[5]
3:45991675	rs71288014	G	GCCTTACC...	0.56	0.52-0.59	2.7×10^{-80}		0.14	55870	FYCO1	SCGG[5]
3:46227281	rs6775046	A	T	0.84	0.82-0.87	1.2×10^{-35}		4.3×10^{-22}	2970910	CCR3	SCGG[5]
3:47920114	rs12631365	C	G	1	1.02-1.06	1.3×10^{-5}	2.9×10^{-8}	0.06	2909630	MAP4	GenOMICC^{new}
3:101800253	rs11712309	T	C	1.1	1.05-1.09	2.8×10^{-13}		0.35	2953910	NXPE3	GenOMICC^{new}
3:146520241	rs186910	A	G	0.91	0.88-0.93	1.5×10^{-10}		0.11	2958740	PLSCR1	GenOMICC[3]
4:25446871	rs7664615	A	G	1.1	1.05-1.1	1.6×10^{-9}		0.14	2912790	ANAPC4	GenOMICC^{new}
4:105669137	rs115599607	T	G	0.9	0.87-0.93	7×10^{-9}		0.0034	2951240	ARHGEF38	GenOMICC^{new}
5:132427482	rs4143335	T	C	1.1	1.05-1.1	1.3×10^{-9}		0.0086	2948530	IRF1-AS1	GenOMICC[3]
6:31153649	rs143334143*	A	G	1.2	1.13-1.19	7.5×10^{-23}		1.7×10^{-21}	2959380	CCHCR1	GenOMICC[6]
6:31556227	rs3838684	G	GC	0.93	0.9-0.95	1.5×10^{-9}		0.81	947977	NFKBIL1	GenOMICC^{new}
6:41522644	rs41435745	C	G	1.2	1.18-1.29	1.2×10^{-20}		0.019	2834490	FOXP4	HGI[4]
7:100032719	rs2897075	T	C	1.1	1.04-1.07	5.8×10^{-9}		0.28	2971270	ZKSCAN1	GenOMICC^{new}
8:60507169	rs2875974	A	G	1.1	1.04-1.07	6.8×10^{-10}		0.1	2971690	RAB2A	GenOMICC^{new}
9:21206606	rs28368148	C	G	0.75	0.68-0.83	4×10^{-8}		0.01	2326800	IFNA10	GenOMICC[3]
9:33425570	rs60639666	A	G	1.1	1.04-1.09	5×10^{-9}		0.037	2971830	AQP3	SCOURGE[2]
9:133274084	rs529565	T	C	0.91	0.9-0.93	9.9×10^{-24}		0.36	2936170	ABO	SCOURGE[2]
10:79946568	rs721917	A	G	0.94	0.93-0.96	1.5×10^{-10}		0.13	2947310	SFTPD	HGI[7]
11:1219991	rs35705950	T	G	0.89	0.87-0.92	3.6×10^{-14}		0.48	2674960	MUC5B	HGI[7]
11:34480495	rs7949972	T	C	0.91	0.89-0.92	2.9×10^{-28}		0.029	2964100	ELF5	GenOMICC[3]
12:112919637	rs2660	A	G	1.1	1.06-1.1	3.1×10^{-17}		0.00038	2948600	OAS1	GenOMICC[6]
12:132565387	rs5023077	T	C	1.1	1.05-1.09	1.2×10^{-13}		0.24	2829560	FBRSL1	GenOMICC[3]
13:112881427	rs12585036	T	C	1.1	1.09-1.13	1.1×10^{-22}		0.0021	2746150	ATP11A	GenOMICC[3]

Chr:pos(b38)	rsid	EA	Non-EA	OR	OR_{CI}	P	P_{cond}	P_{Het}	N	Nearest Gene	Citation
16:89196249	rs117169628	A	G	1.1	1.09-1.14	2.6×10^{-17}		0.23	2962620	SLC22A31	GenOMICC[3]
17:45983409	rs63750417	T	C	0.91	0.89-0.93	1.2×10^{-18}		0.27	2948600	MAPT	GenOMICC[new]
17:49863303	rs77534576	T	C	1.2	1.19-1.31	2×10^{-17}		0.11	2865730	TAC4	[8]
19:4063488	rs66833742	T	C	0.93	0.91-0.95	2.1×10^{-10}		0.082	2918950	ZBTB7A	GenOMICC[new]
19:4723658	rs2277732	A	C	1.2	1.14-1.19	1.2×10^{-45}		1.7×10^{-8}	2897900	DPP9	GenOMICC[6]
19:10352632	rs149329233	A	G	1.3	1.16-1.39	2.4×10^{-7}	3.6×10^{-8}	0.91	2788380	TYK2	GenOMICC[6]
19:10381598	rs144309607	T	C	1.3	1.21-1.32	4.4×10^{-23}		0.00046	2909050	TYK2	GenOMICC[6]
19:10414696	rs142770866	A	G	1.1	1.11-1.18	2.6×10^{-15}		0.041	2950950	PDE4A	GenOMICC[new]
19:48702851	rs679574	C	G	1.1	1.05-1.09	1.7×10^{-13}		0.037	2960610	FUT2	GenOMICC[3]
19:50379362	rs1405655	T	C	0.93	0.91-0.95	5.7×10^{-16}		0.02	2973250	NR1H2	HGI[7]
20:6517380	rs6117308	A	G	0.95	0.93-0.97	3.8×10^{-9}		0.57	2973250	CASC20	GenOMICC[new]
21:33237639	rs9636867	A	G	0.88	0.86-0.89	1.9×10^{-46}		9.6×10^{-8}	2972780	IFNAR2	GenOMICC[6]
21:33287378	rs8178521	T	C	1.1	1.06-1.11	2.6×10^{-13}		0.0039	2914120	IL10RB	GenOMICC[3]
21:33954962	rs78258279	A	G	0.88	0.85-0.9	2.3×10^{-18}		0.002	2964540	LINC00649	GenOMICC[3]
21:41479527	rs915823	A	C	1.1	1.05-1.1	6.5×10^{-11}		0.84	2966090	TMPRSS2	GenOMICC[new]
X:15497196		C	G	0.96	0.95-0.97	2.9×10^{-11}		0.45	2377330	ACE2	HGI[7]

7.1 Forest plots

Forest plots are shown here, plotting odds ratio and confidence interval of each variant per cohort for critical and hospitalised phenotypes, respectively. GenOMICC (meta-analysis of European, South Asian, East Asian and African ancestries recruited in UK and Ireland), GenOMICC Saudi Arabia, GenOMICC Brazil, HGI v6, SCOURGE and 23andMe (meta-analysis of Latino, European and African ancestries for 23andMe summary statistics). GenOMICC cohorts are represented in blue, HGI in purple, SCOURGE in green and 23andMe in orange. Size of the square represents weight of the cohort in a fixed-effect meta-analysis.

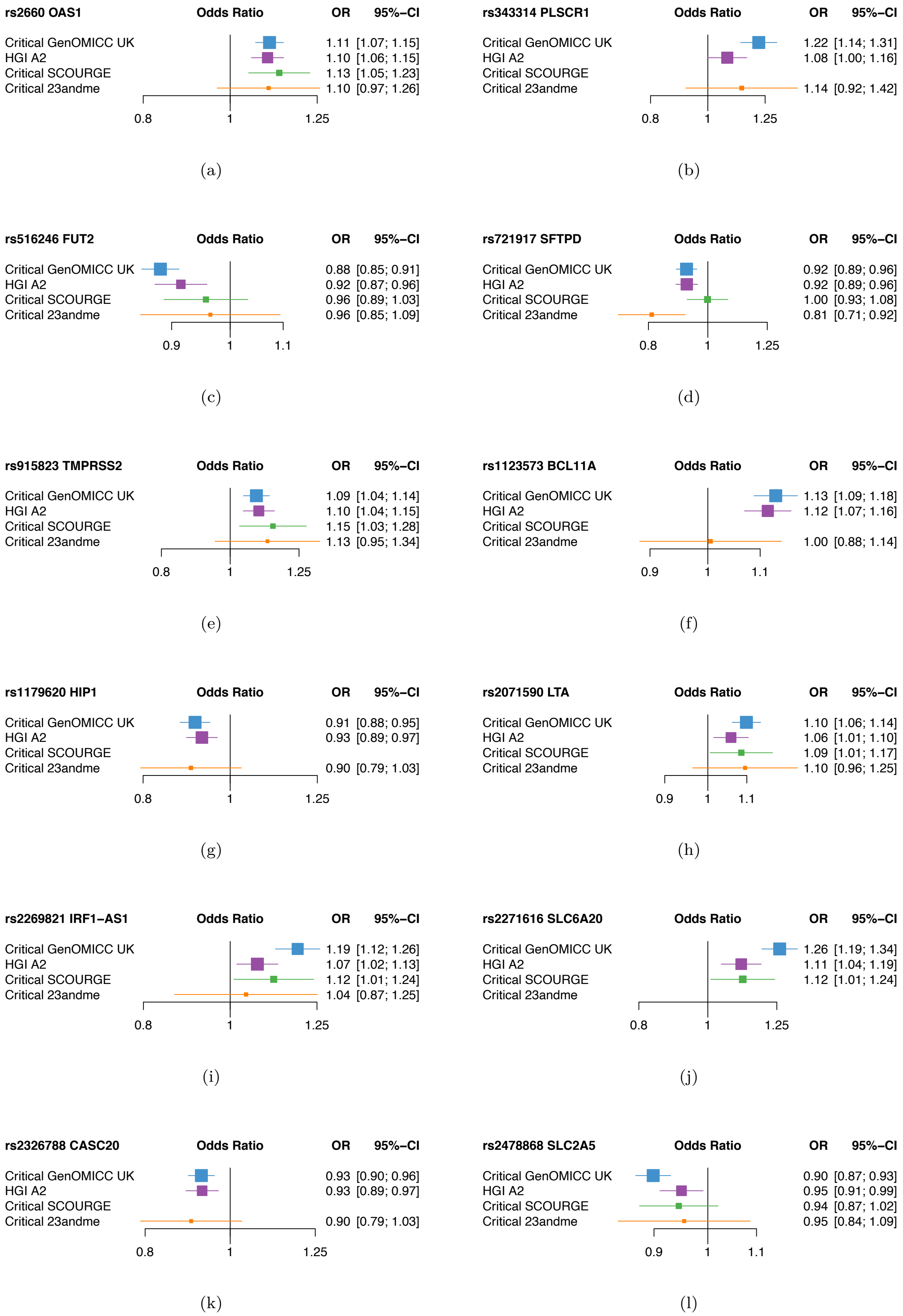

Supplementary Figure 2: Forest plots (critical phenotype)

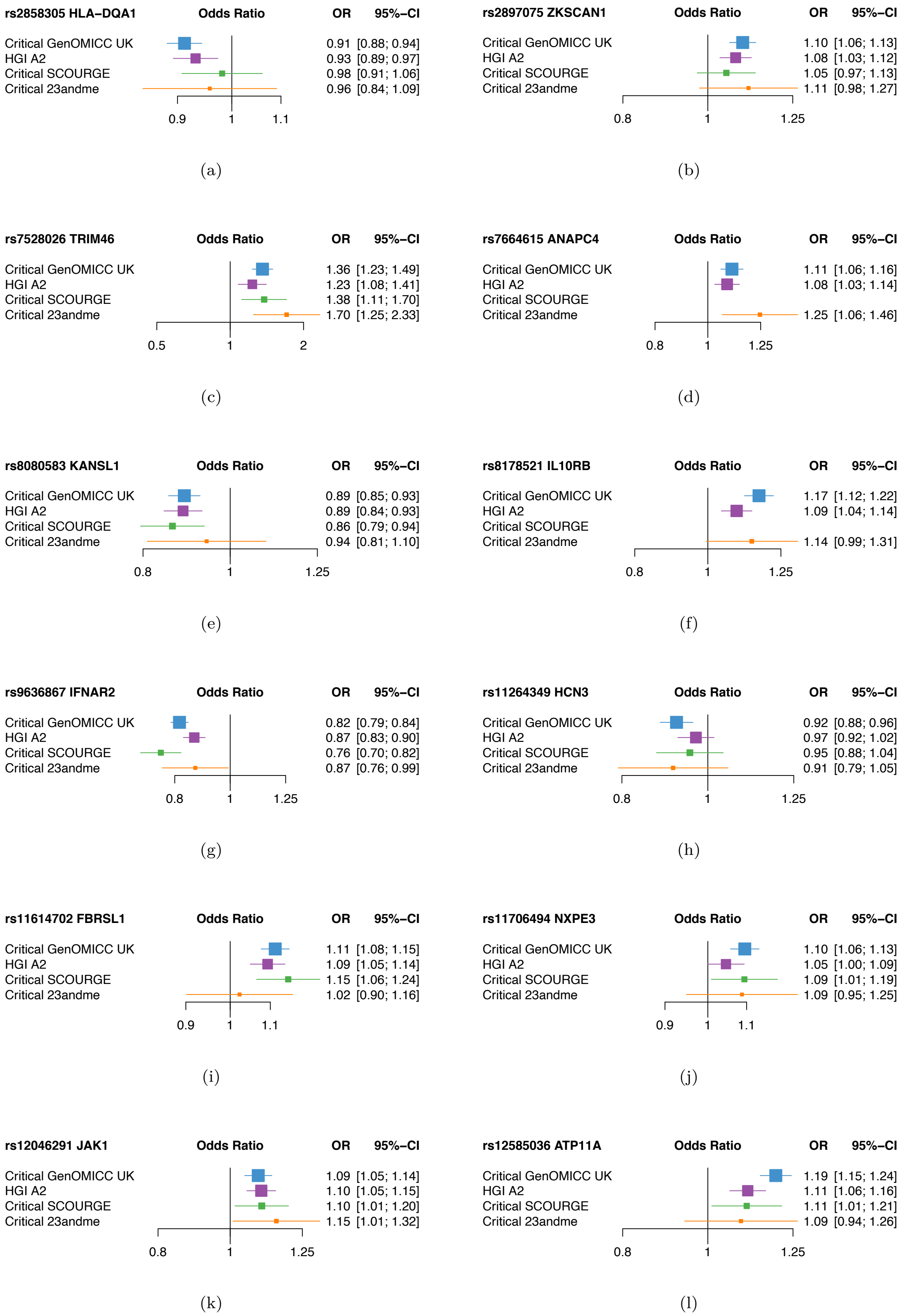

Supplementary Figure 3: Forest plots (critical phenotype)

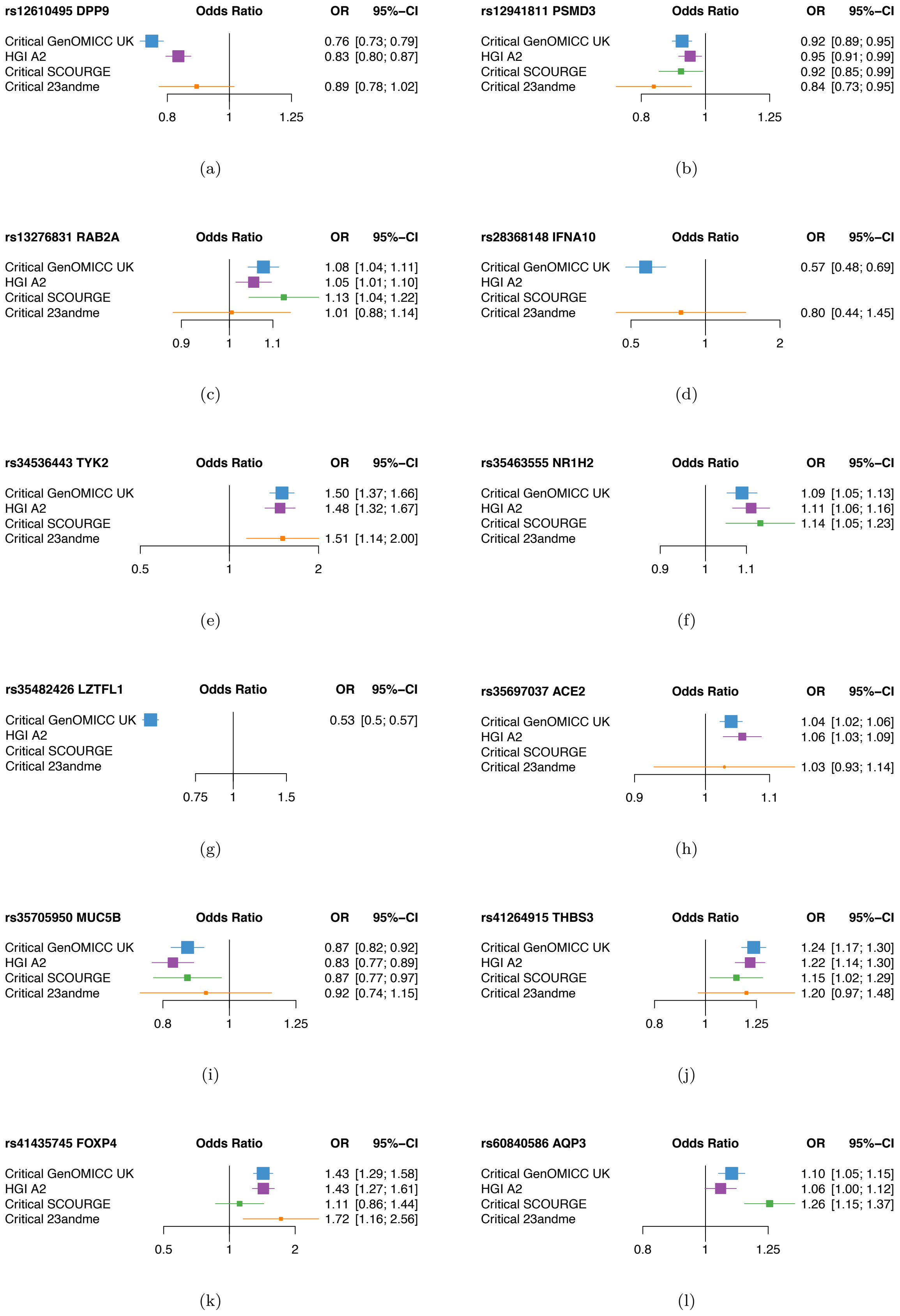

Supplementary Figure 4: Forest plots (critical phenotype)

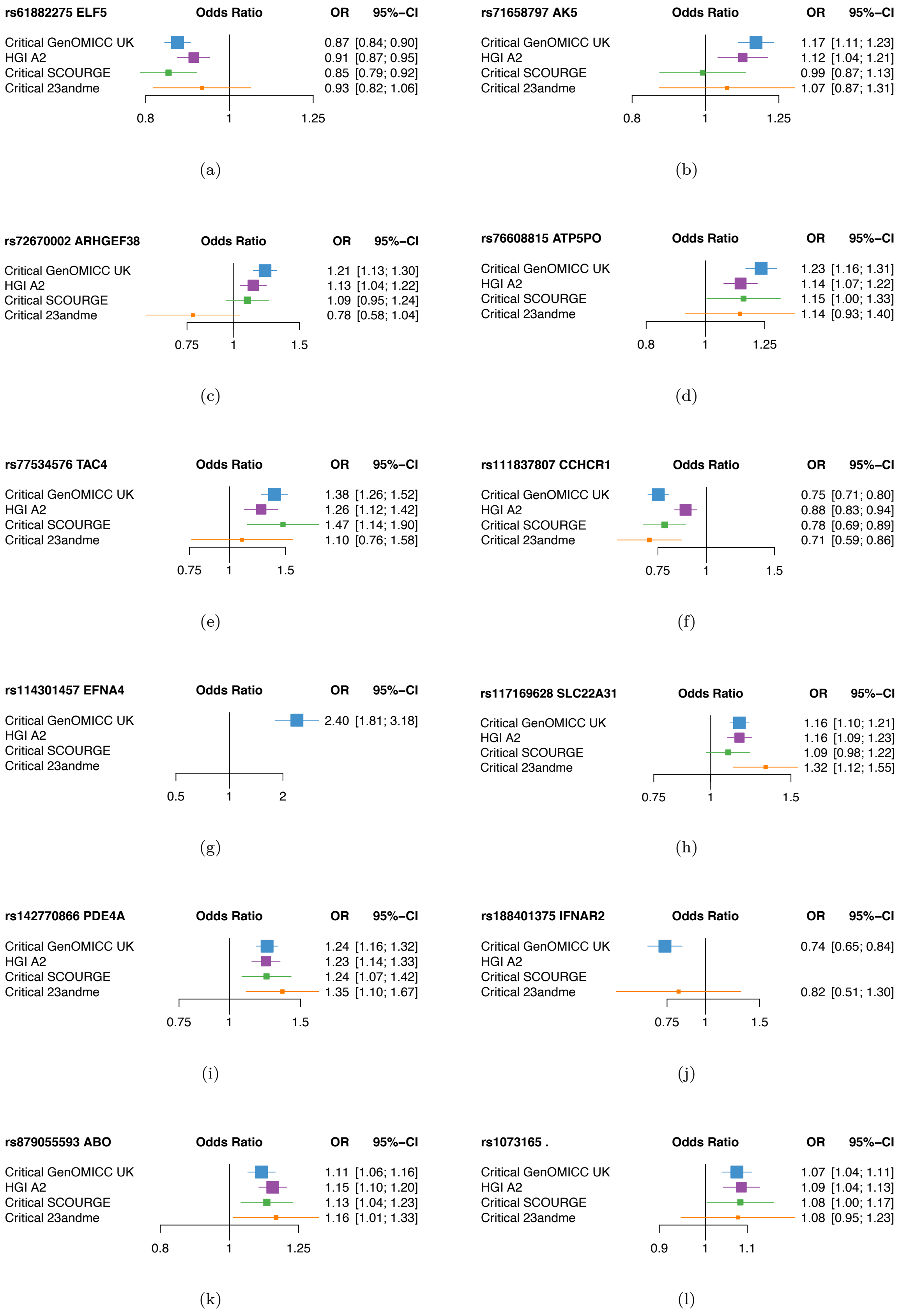

Supplementary Figure 5: Forest plots (critical phenotype)

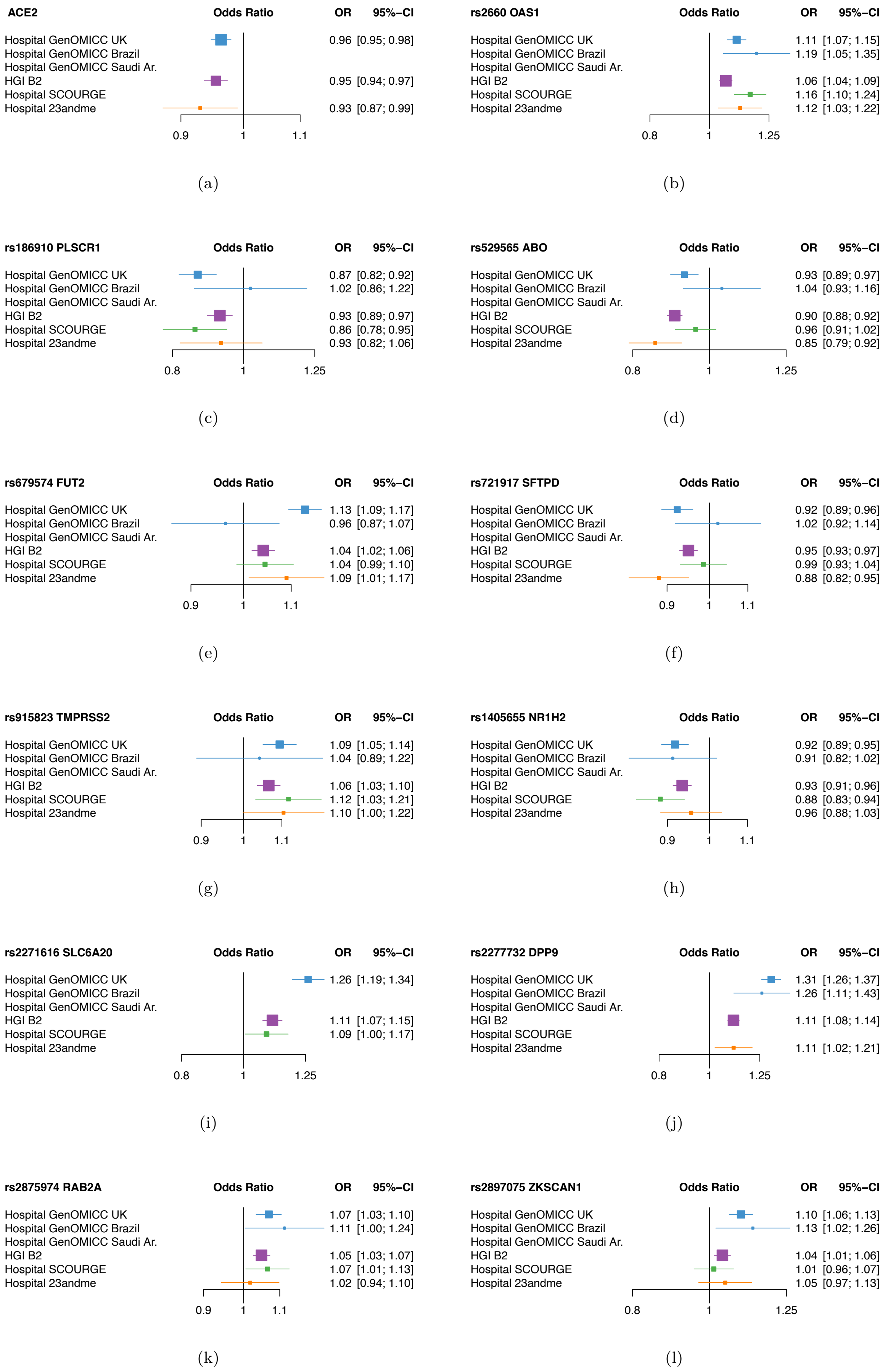

Supplementary Figure 6: Forest plots (hospitalised phenotype)

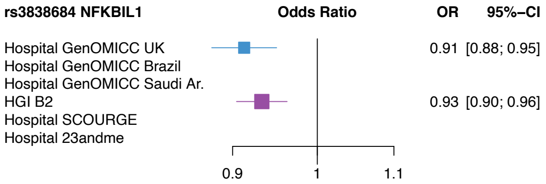

(a)

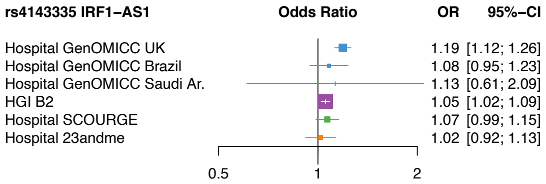

(b)

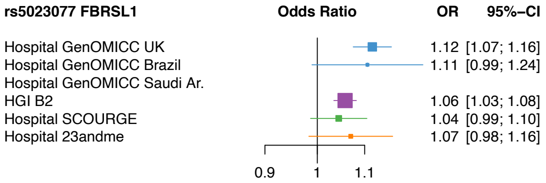

(c)

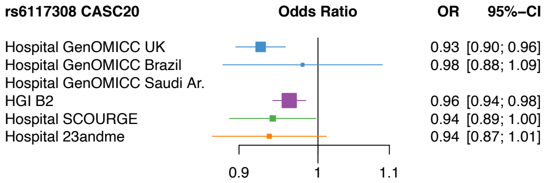

(d)

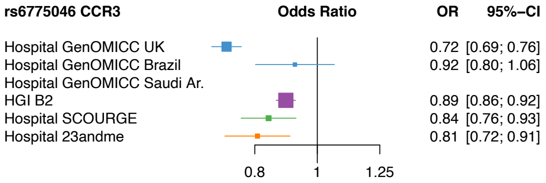

(e)

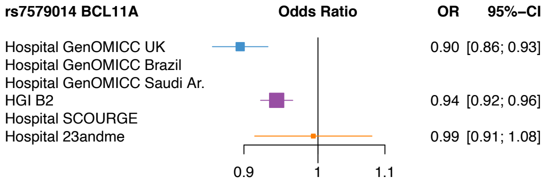

(f)

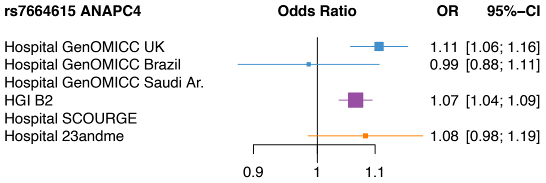

(g)

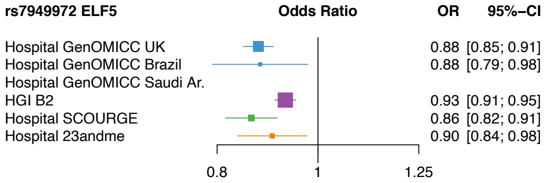

(h)

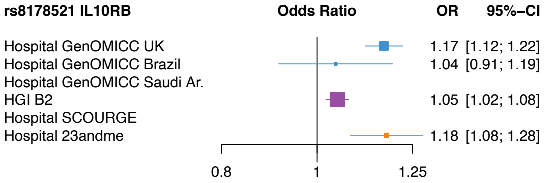

(i)

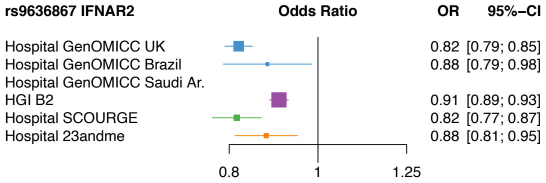

(j)

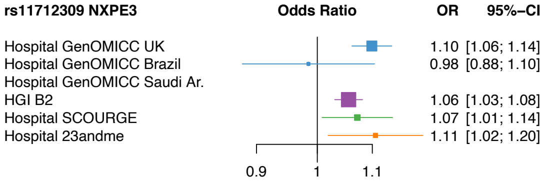

(k)

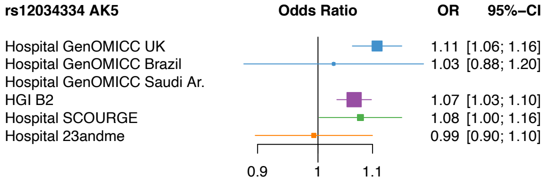

(l)

Supplementary Figure 7: Forest plots (hospitalised phenotype)

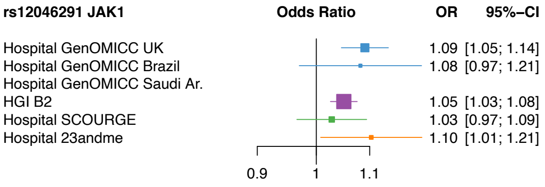

(a)

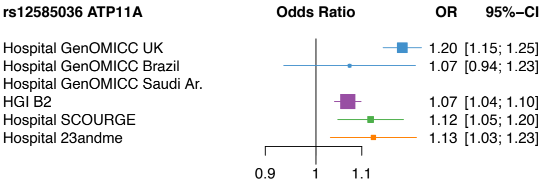

(b)

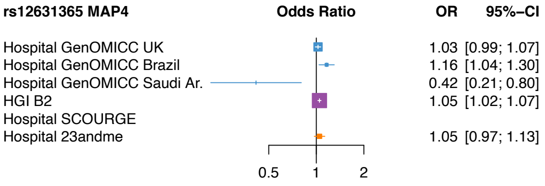

(c)

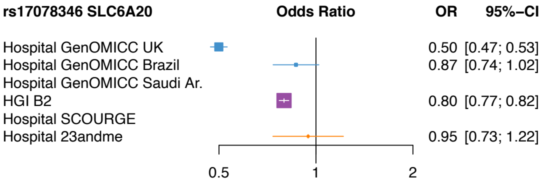

(d)

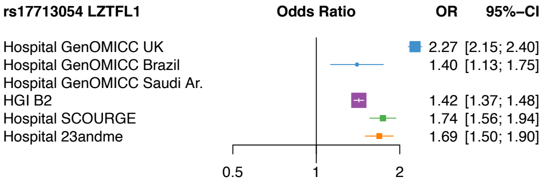

(e)

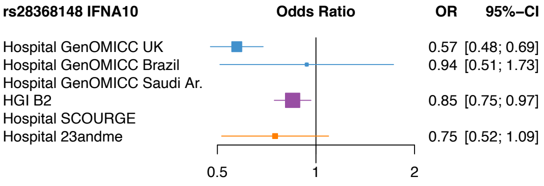

(f)

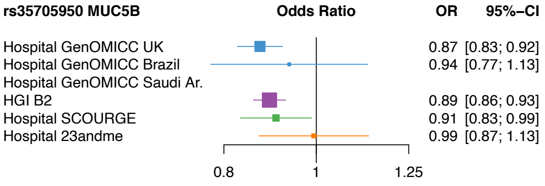

(g)

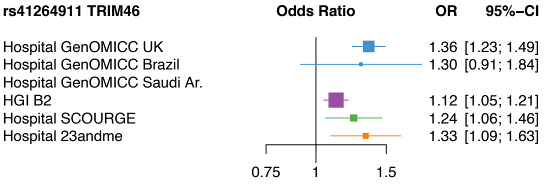

(h)

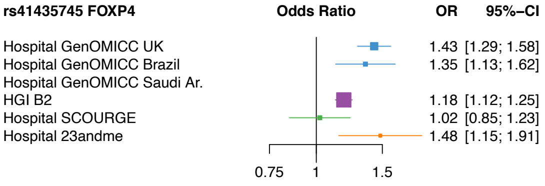

(i)

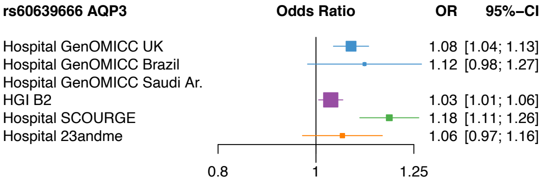

(j)

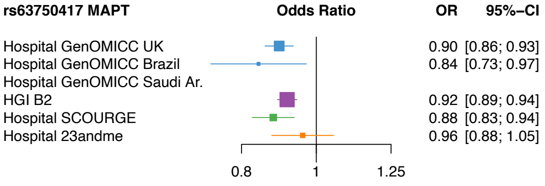

(k)

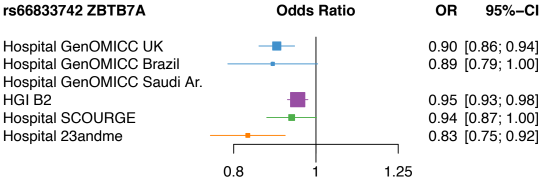

(l)

Supplementary Figure 8: Forest plots (hospitalised phenotype)

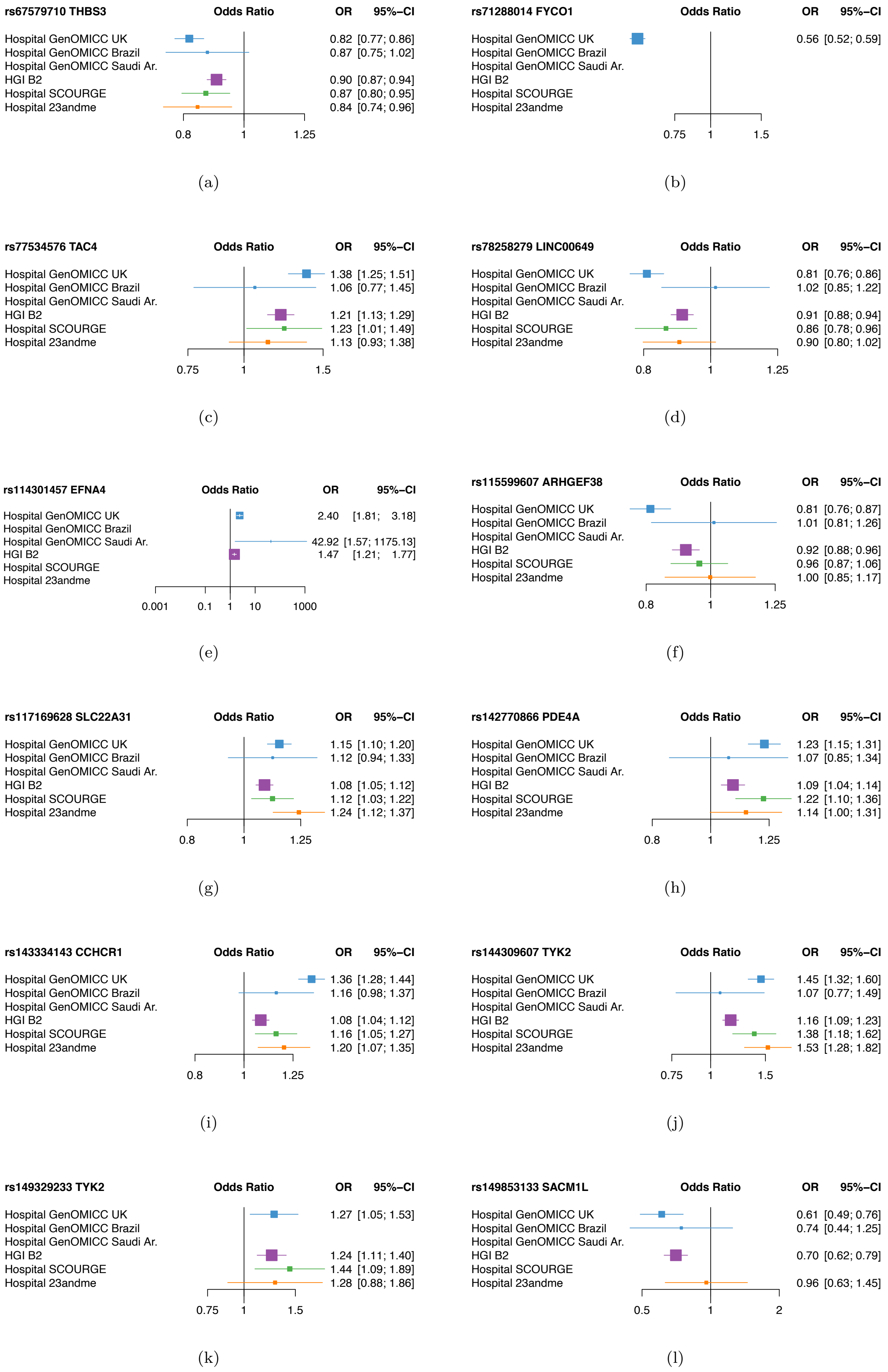

Supplementary Figure 9: Forest plots (hospitalised phenotype)

8 Mendelian randomisation for Protein expression (INTERVAL)

Full tabular results from GSMR analyses are available in Supplementary Table 4 and Supplementary Table 3.

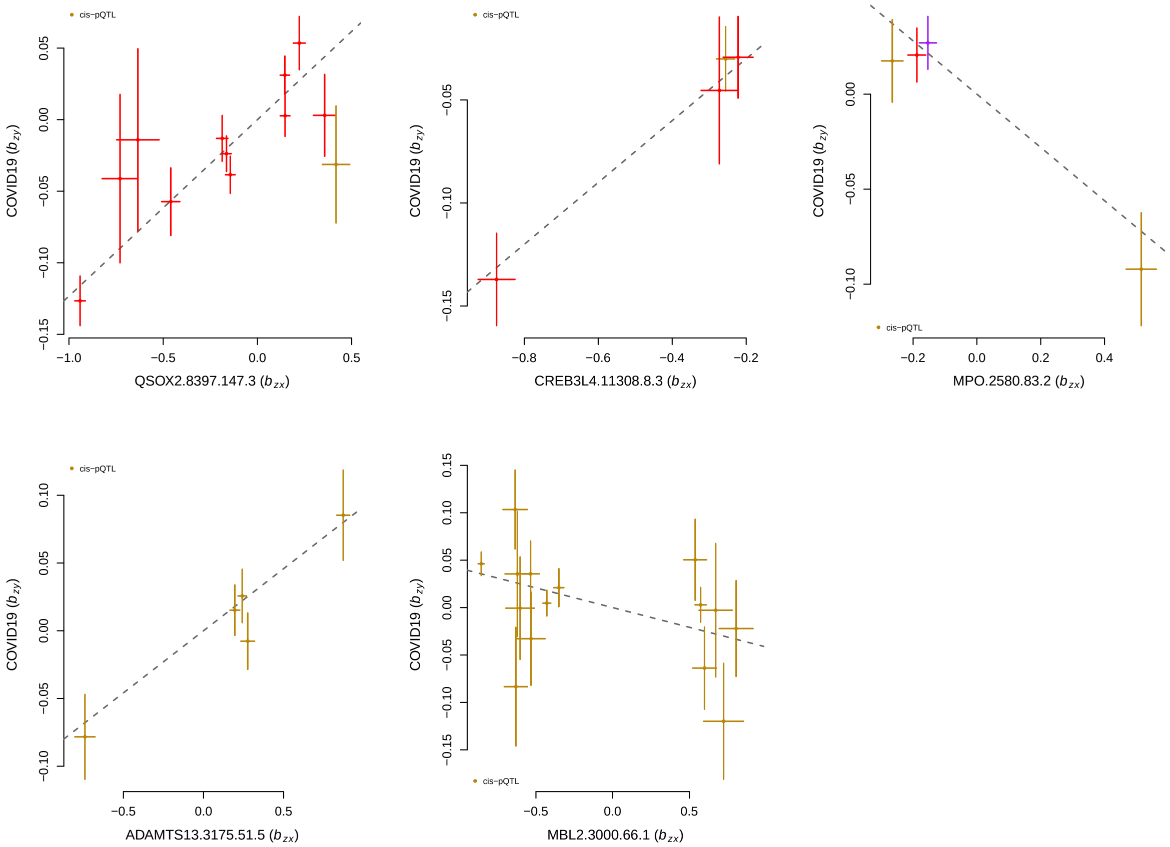

Supplementary Figure 10: GSMR effect-size plots. SNP to protein expression and SNP to Covid-19 severity effect-size are shown for QSOX2, CREB3L4, MPO, ADAMTS13, and MBL2. These protein GSMR results are novel with respect a similar analysis performed using the most recent GenOMICC Covid-19 GWAS.[3] Each point on the graph represents a single SNP. cis-pQTL (SNP located within ±2Mb of the protein's locus) are coloured gold. Other colours represent distant loci (each locus greater then 5Mb distant from each other). x-axis: the effect of that SNP on the exposure (protein expression), y-axis: the effect of that SNP on the outcome (Covid-19 severity). Bars are standard errors. The dashed grey line is the GSMR effect-size estimate of exposure on outcome.

9 Mendelian randomisation for RNA expression (eQTLgen)

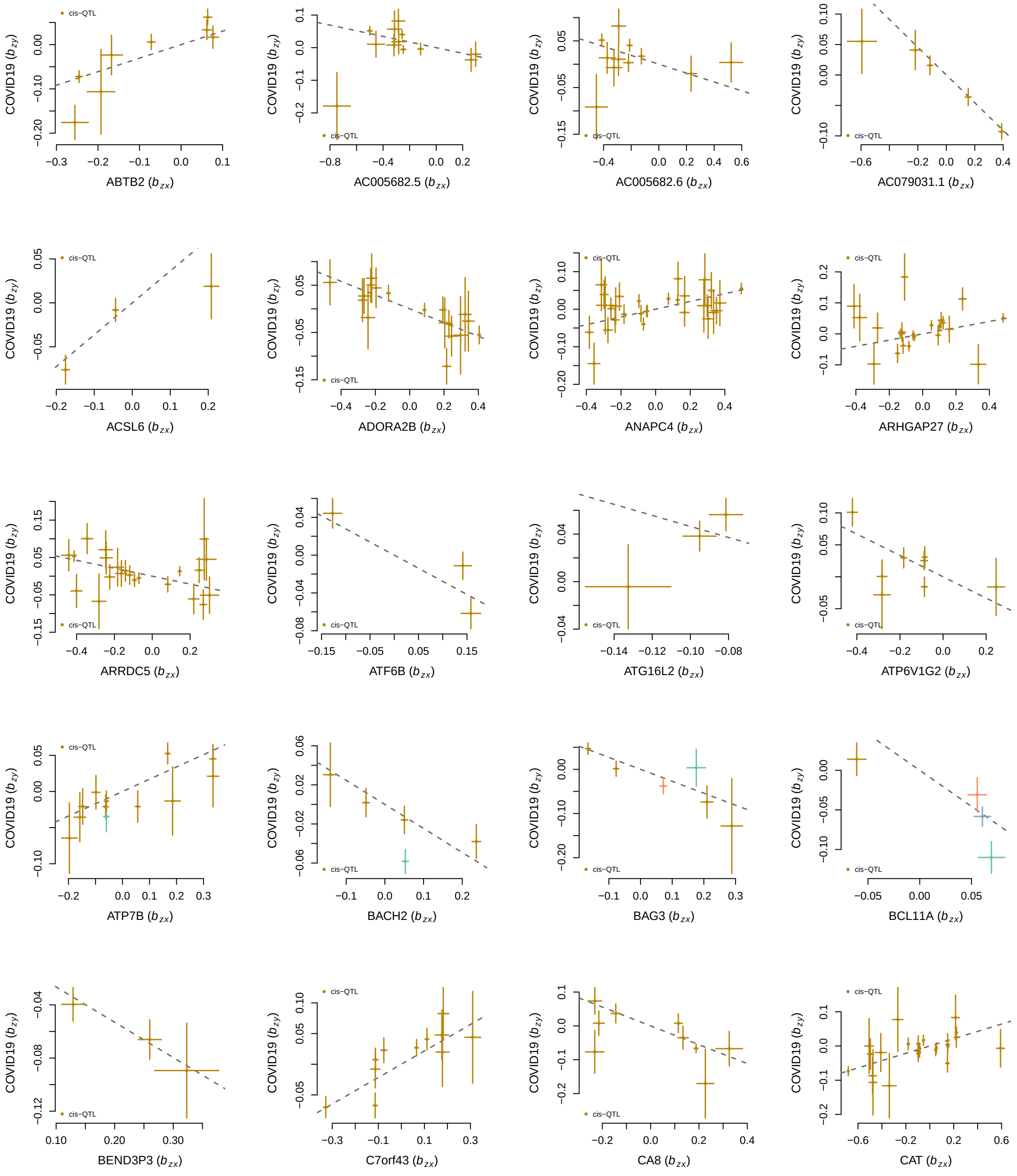

Supplementary Figure 11: Effect size plots for significant RNA expression (eQTLgen) to GenOMICC GSMR (FDR < 0.05; n $=$ 156).

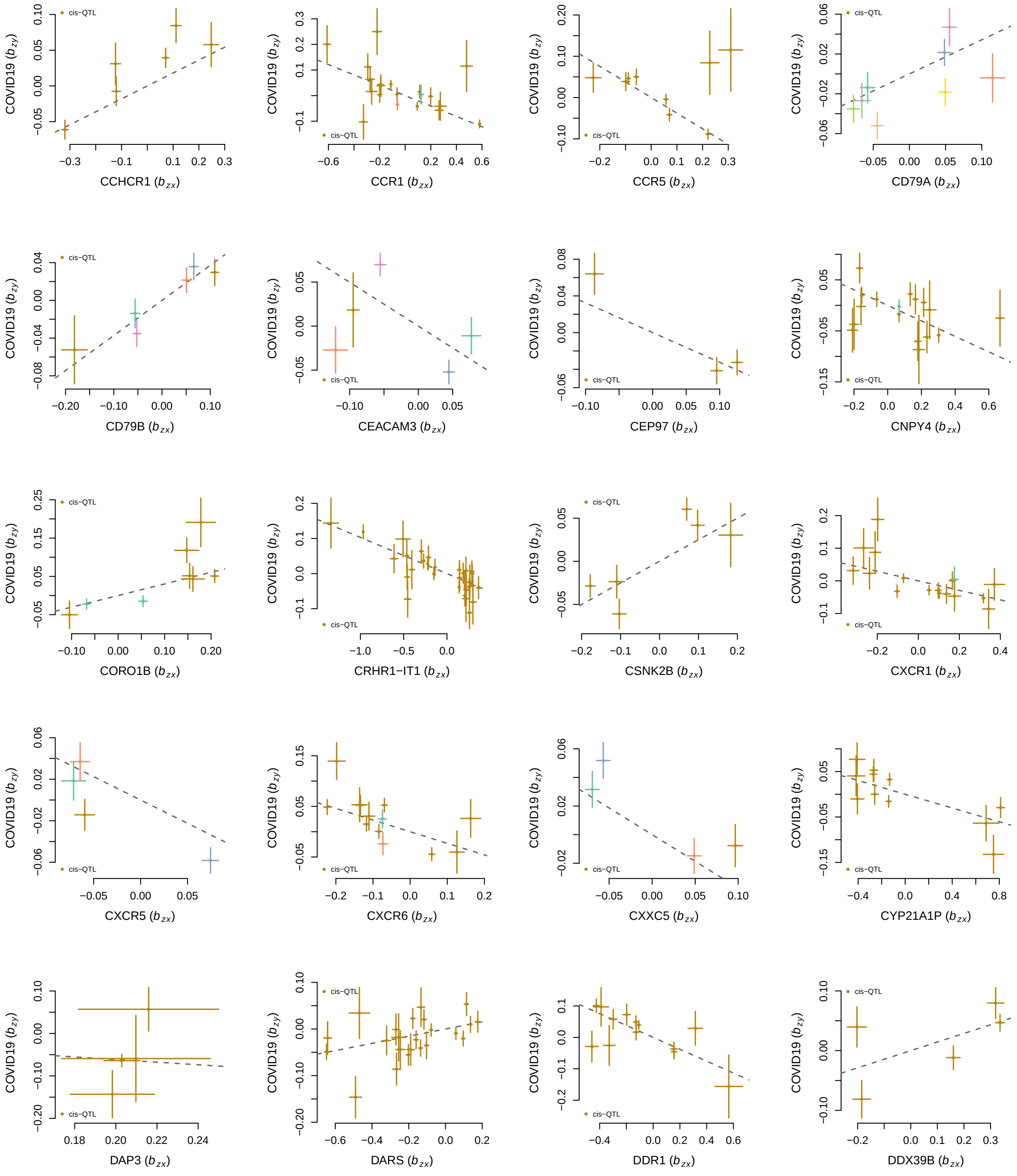

Supplementary Figure 12: Effect size plots for significant RNA expression (eQTLgen) to GenOMICC GSMR (FDR < 0.05; n $= 156$).

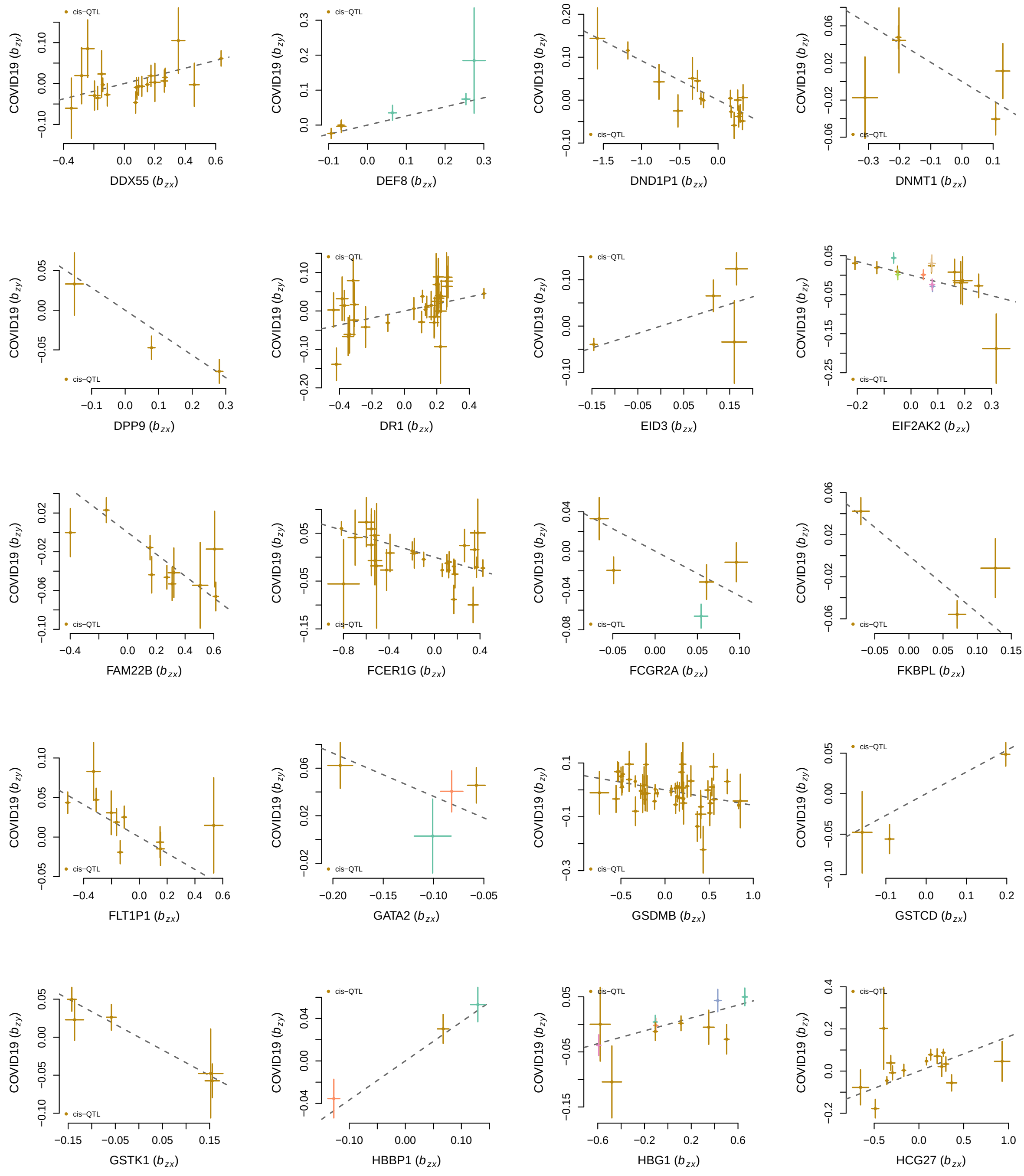

Supplementary Figure 13: Effect size plots for significant RNA expression (eQTLgen) to GenOMICC GSMR (FDR < 0.05; n = 156).

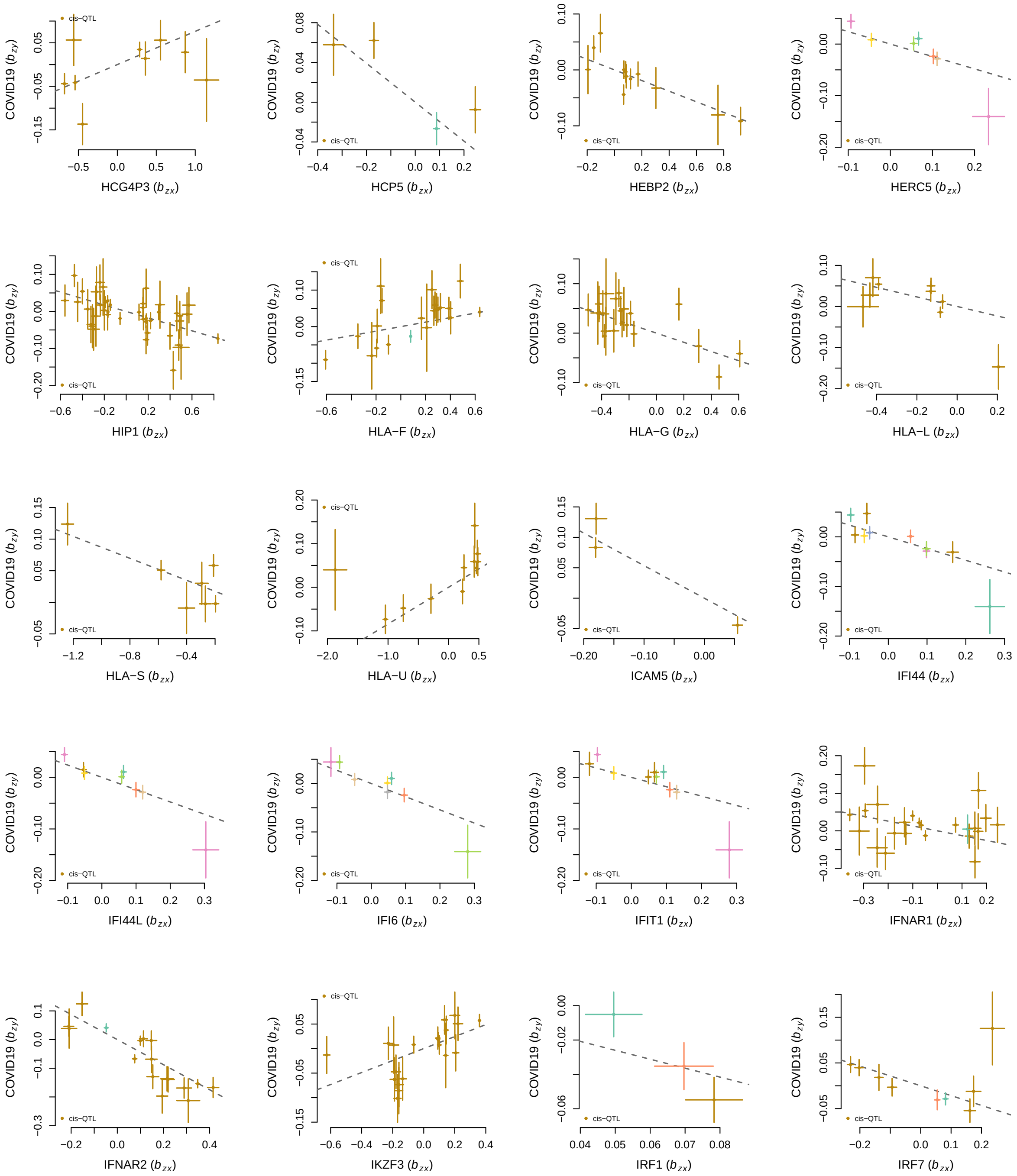

Supplementary Figure 14: Effect size plots for significant RNA expression (eQTLgen) to GenOMICC GSMR (FDR < 0.05; n = 156).

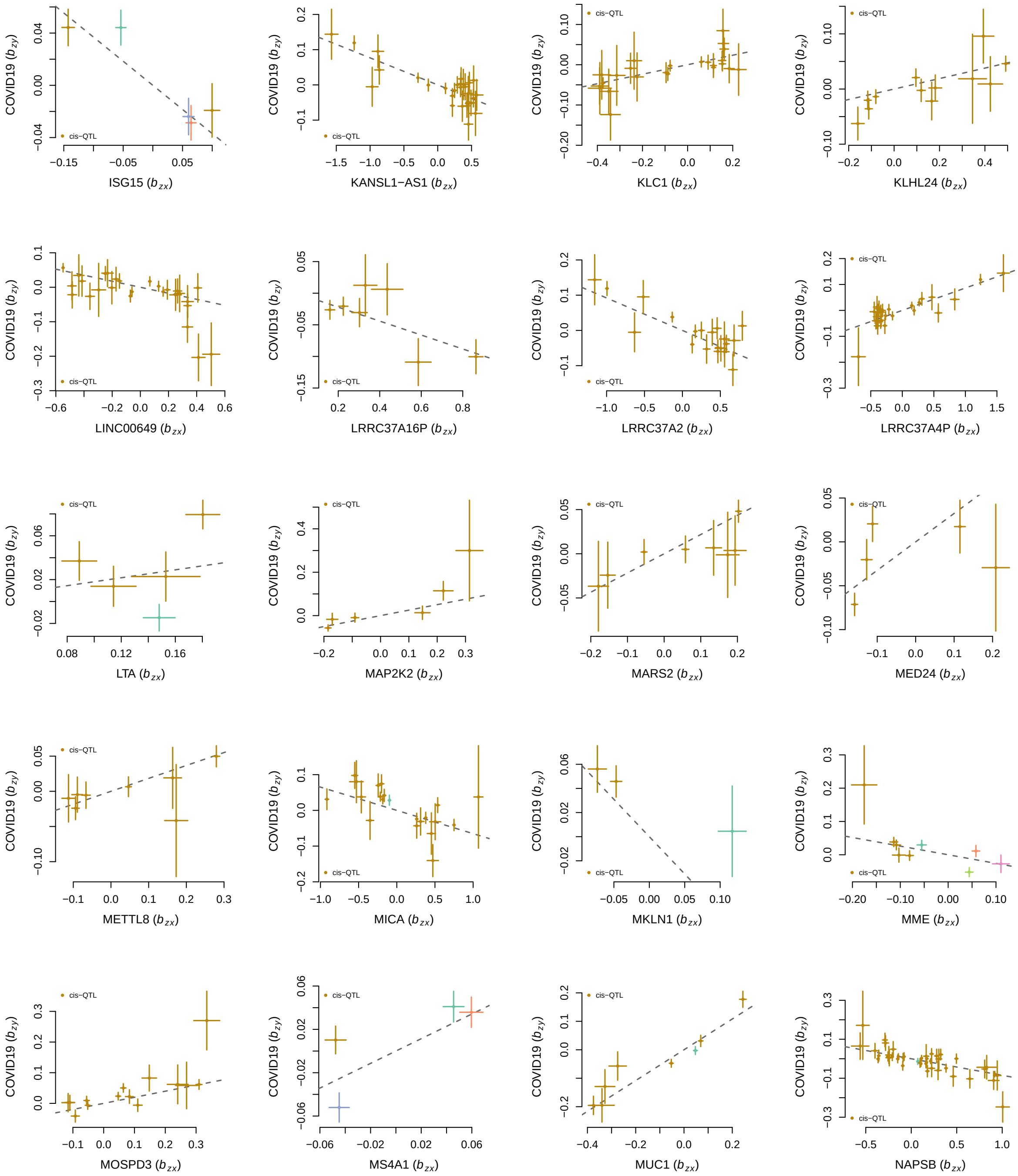

Supplementary Figure 15: Effect size plots for significant RNA expression (eQTLgen) to GenOMICC GSMR (FDR < 0.05; n = 156).

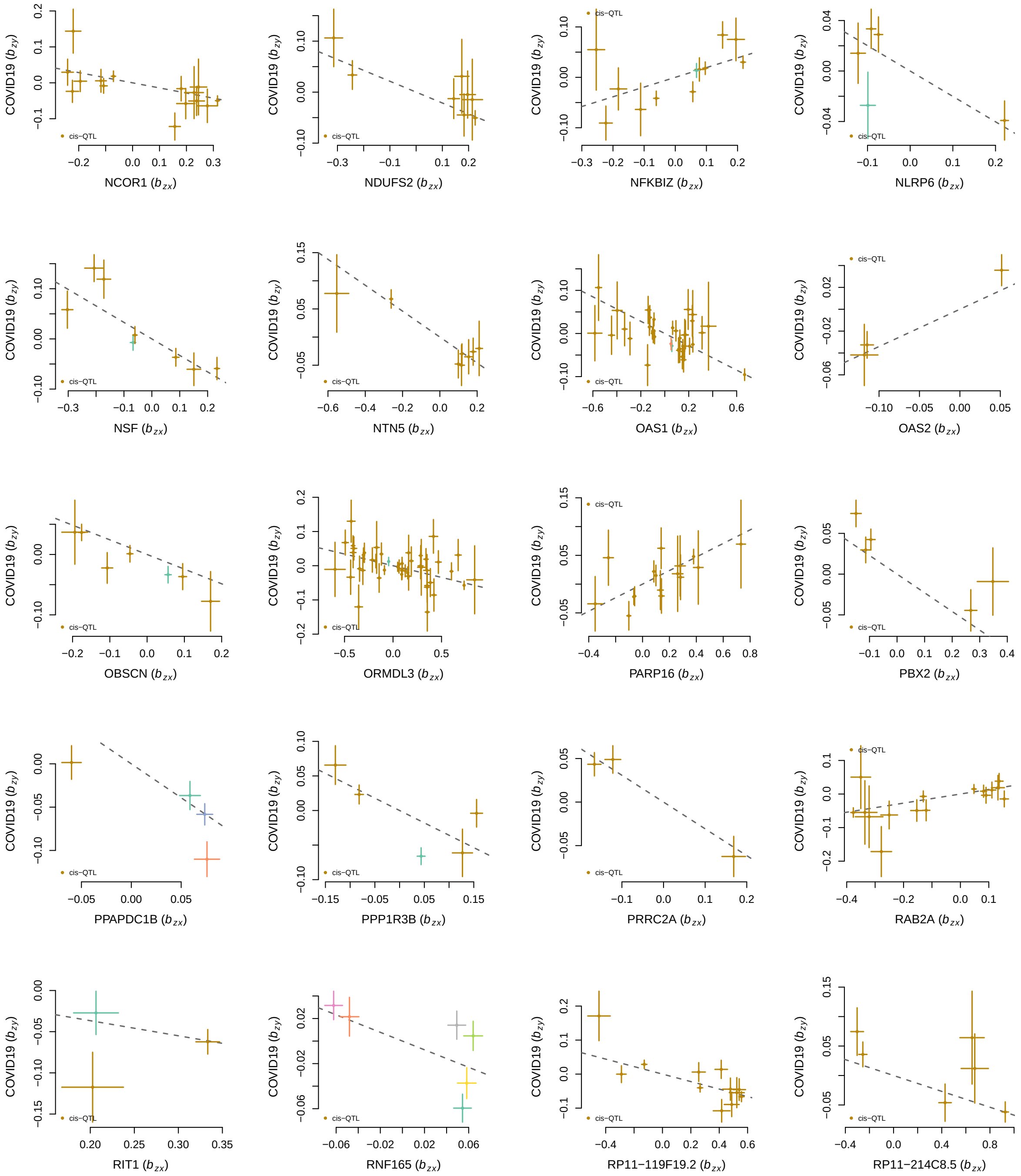

Supplementary Figure 16: Effect size plots for significant RNA expression (eQTLgen) to GenOMICC GSMR (FDR < 0.05; n $= 156$).

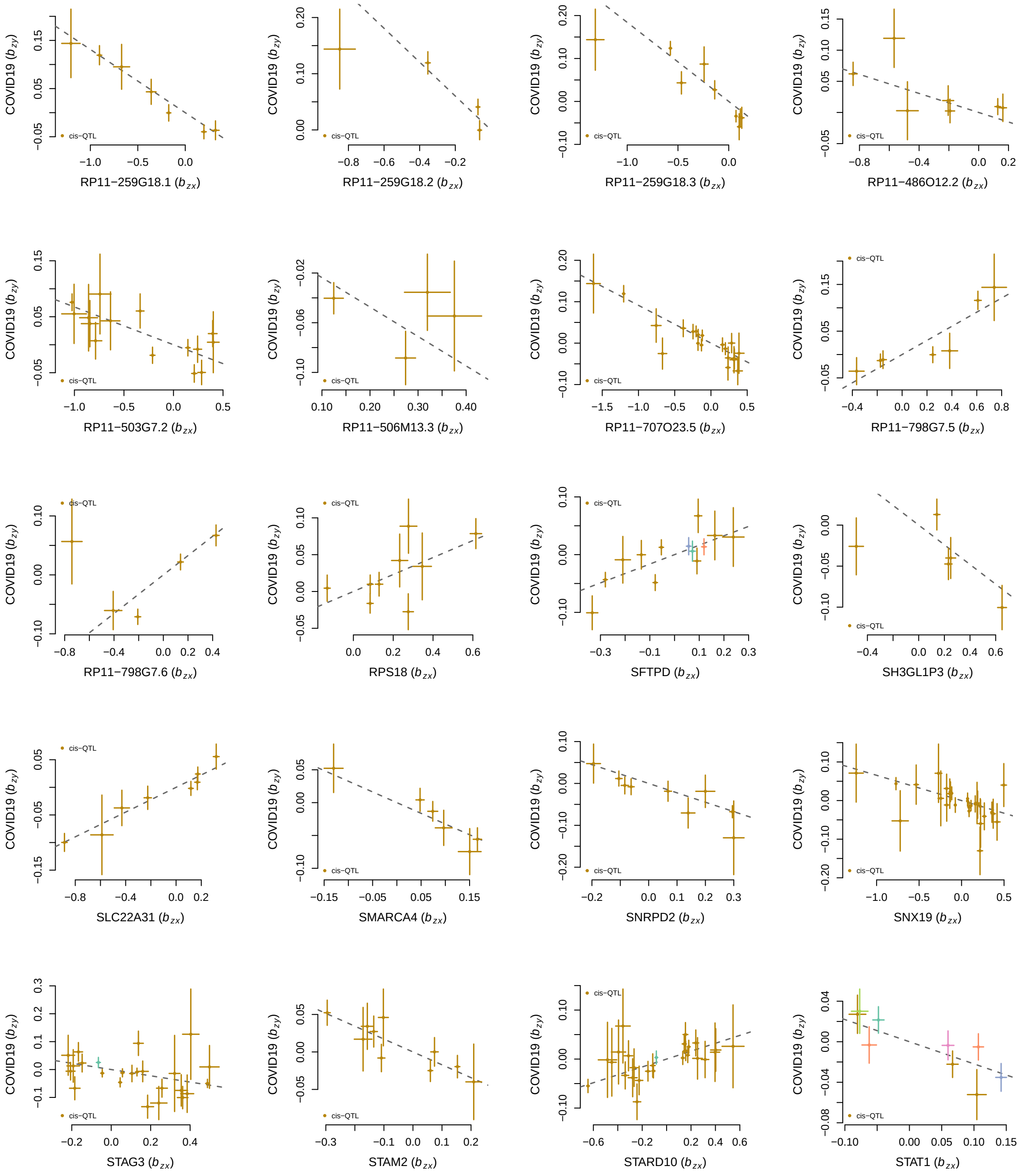

Supplementary Figure 17: Effect size plots for significant RNA expression (eQTLgen) to GenOMICC GSMR (FDR < 0.05; n = 156).

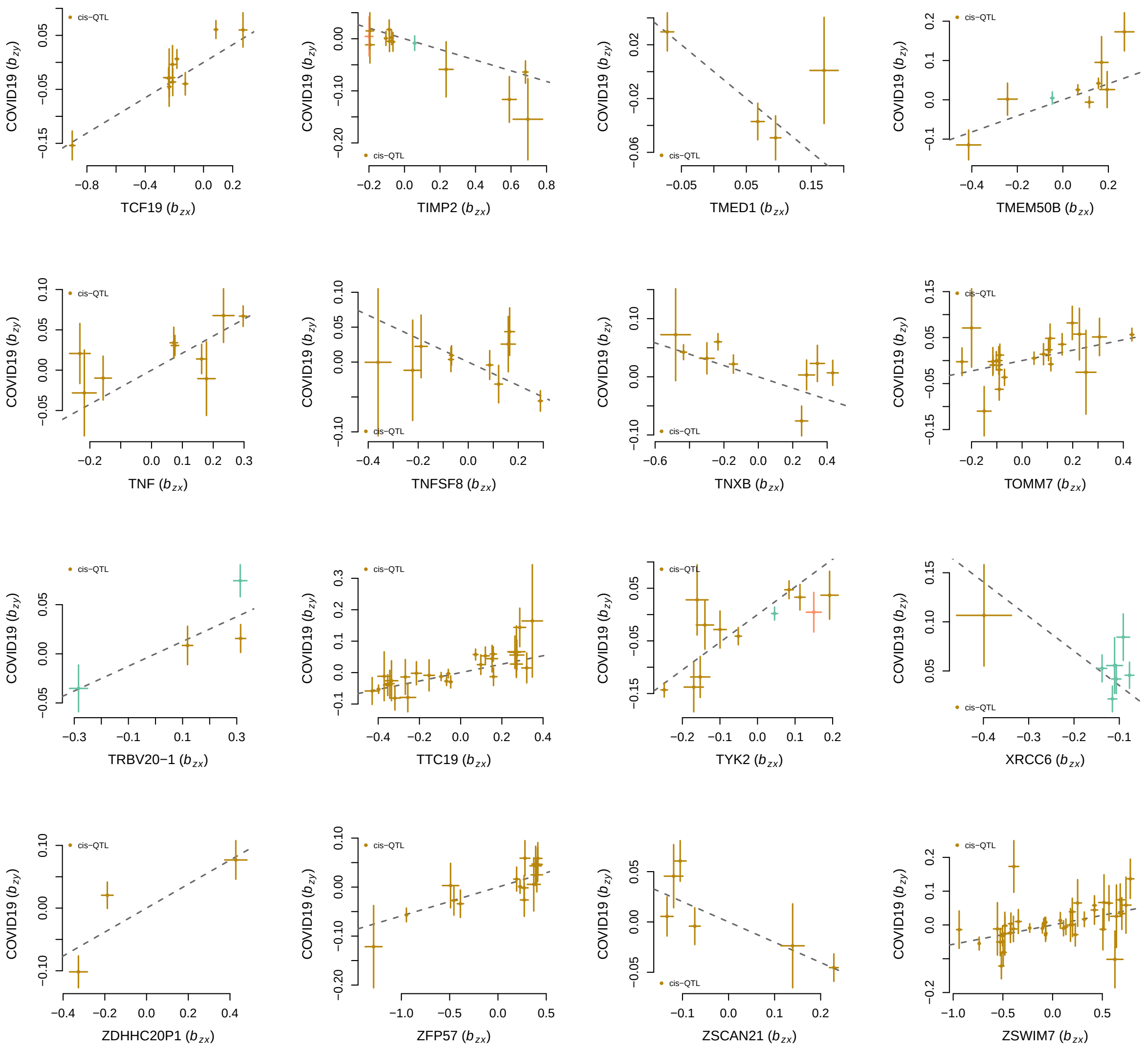

Supplementary Figure 18: Effect size plots for significant RNA expression (eQTLgen) to GenOMICC GSMR (FDR < 0.05; n $=$ 156).

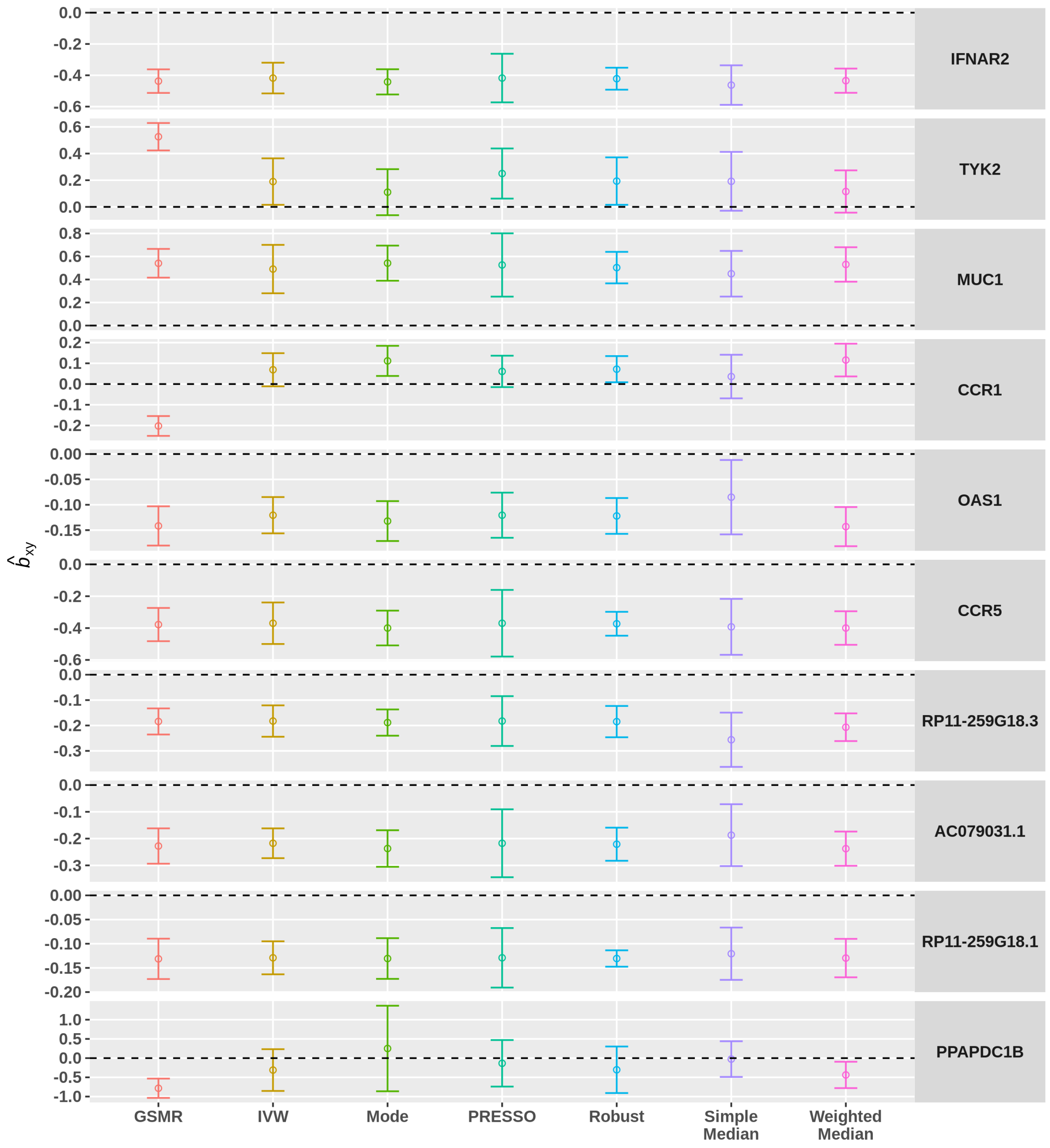

Supplementary Figure 19: Concordance of effect-size estimates using different Mendelian randomisation (MR) methods: GSMR, IVW, Mode, PRESSO, Robust, Simple Median, and Weighted Median. y-axis: effect-size estimates and 95% confidence interval. Significant RNA expression (eQTLgen) to GenOMICC GSMR results displayed (FDR < 0.01; n = 79).

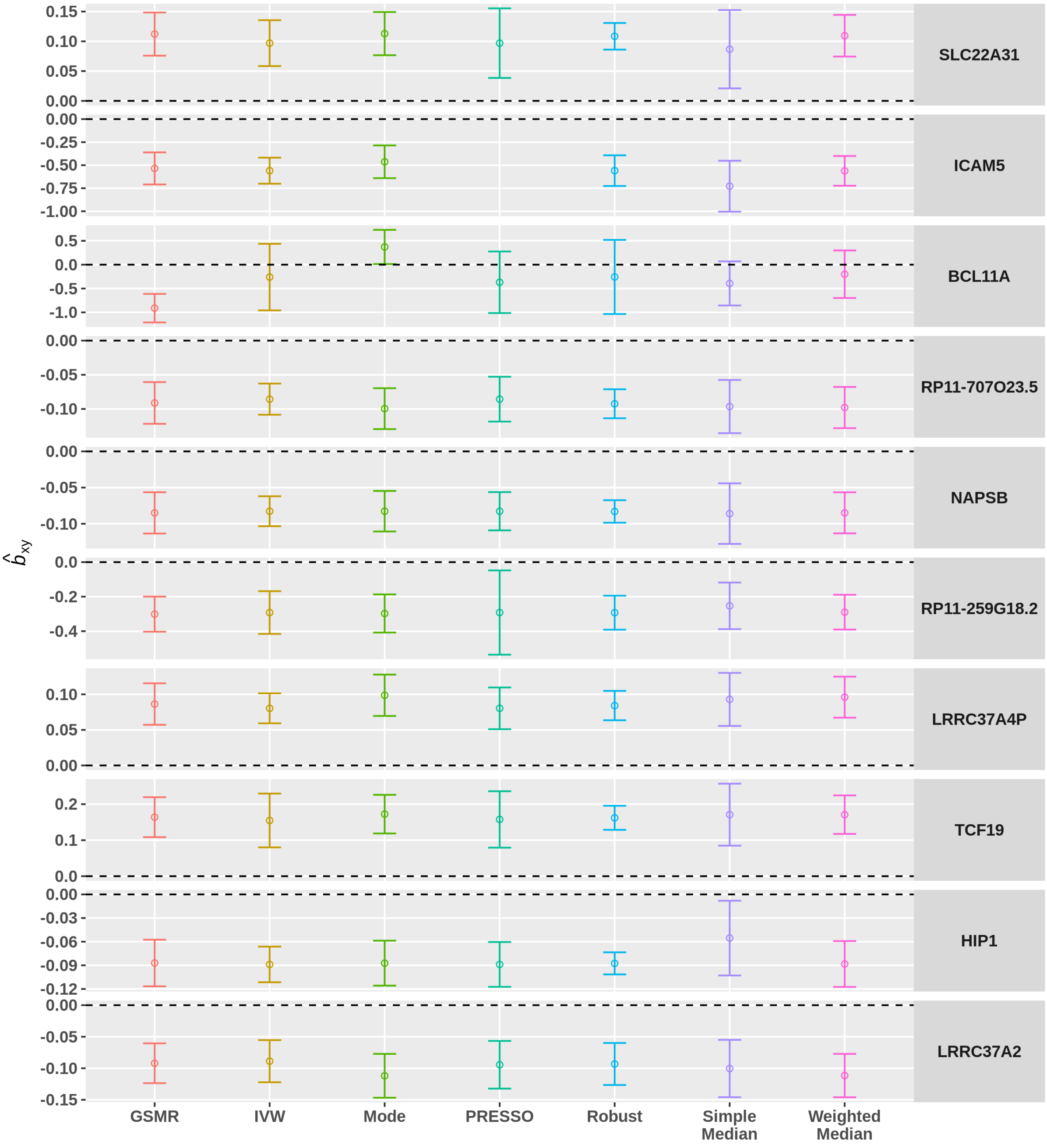

Supplementary Figure 20: Concordance of effect-size estimates using different Mendelian randomisation (MR) methods: GSMR, IVW, Mode, PRESSO, Robust, Simple Median, and Weighted Median. y-axis: effect-size estimates and 95% confidence interval. Significant RNA expression (eQTLgen) to GenOMICC GSMR results displayed (FDR < 0.01; n = 79).

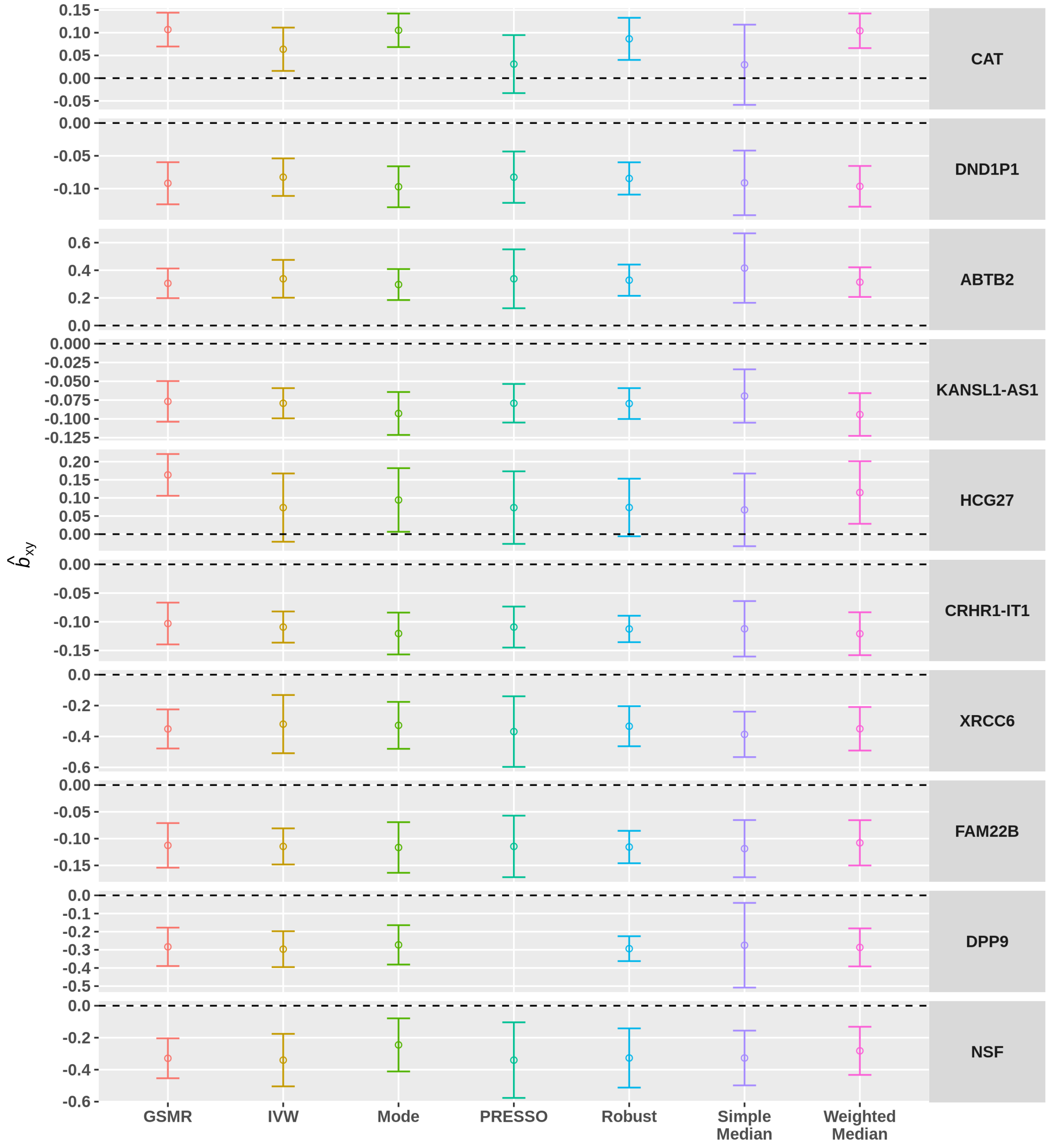

Supplementary Figure 21: Concordance of effect-size estimates using different Mendelian randomisation (MR) methods: GSMR, IVW, Mode, PRESSO, Robust, Simple Median, and Weighted Median. y-axis: effect-size estimates and 95% confidence interval. Significant RNA expression (eQTLgen) to GenOMICC GSMR results displayed (FDR < 0.01; n = 79).

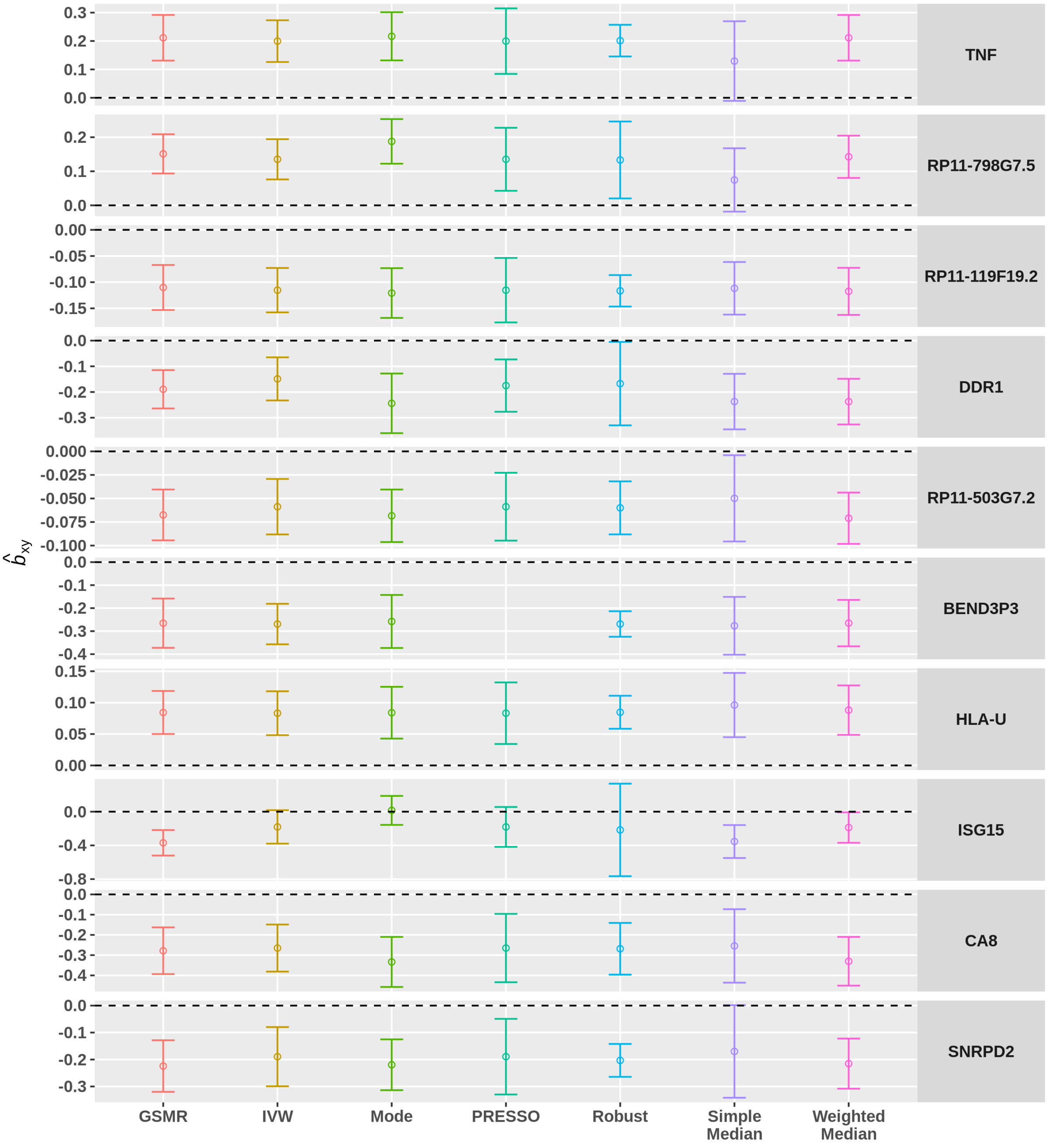

Supplementary Figure 22: Concordance of effect-size estimates using different Mendelian randomisation (MR) methods: GSMR, IVW, Mode, PRESSO, Robust, Simple Median, and Weighted Median. y-axis: effect-size estimates and 95% confidence interval. Significant RNA expression (eQTLgen) to GenOMICC GSMR results displayed (FDR < 0.01; n = 79).

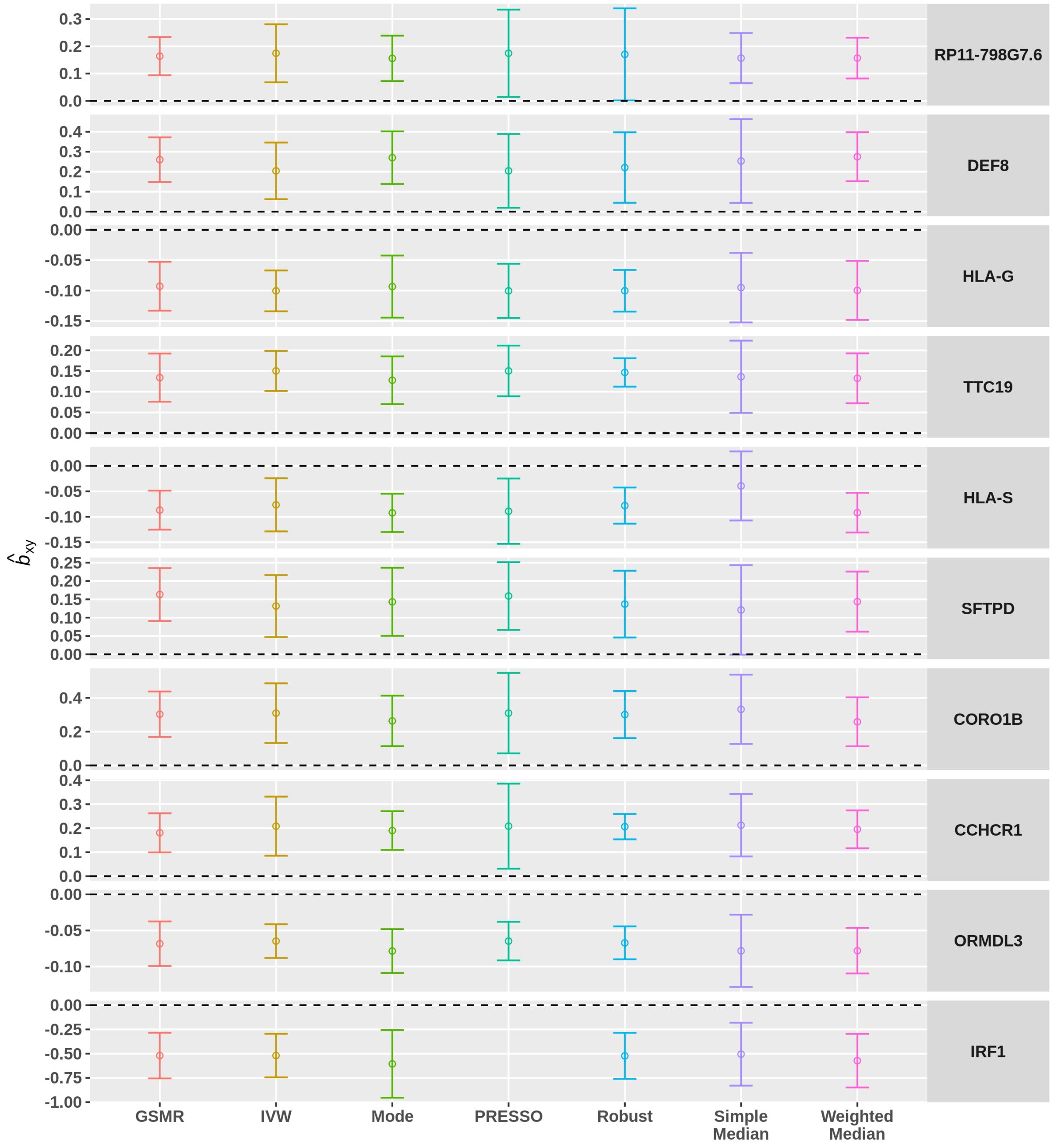

Supplementary Figure 23: Concordance of effect-size estimates using different Mendelian randomisation (MR) methods: GSMR, IVW, Mode, PRESSO, Robust, Simple Median, and Weighted Median. y-axis: effect-size estimates and 95% confidence interval. Significant RNA expression (eQTLgen) to GenOMICC GSMR results displayed (FDR < 0.01; n $= 79$).

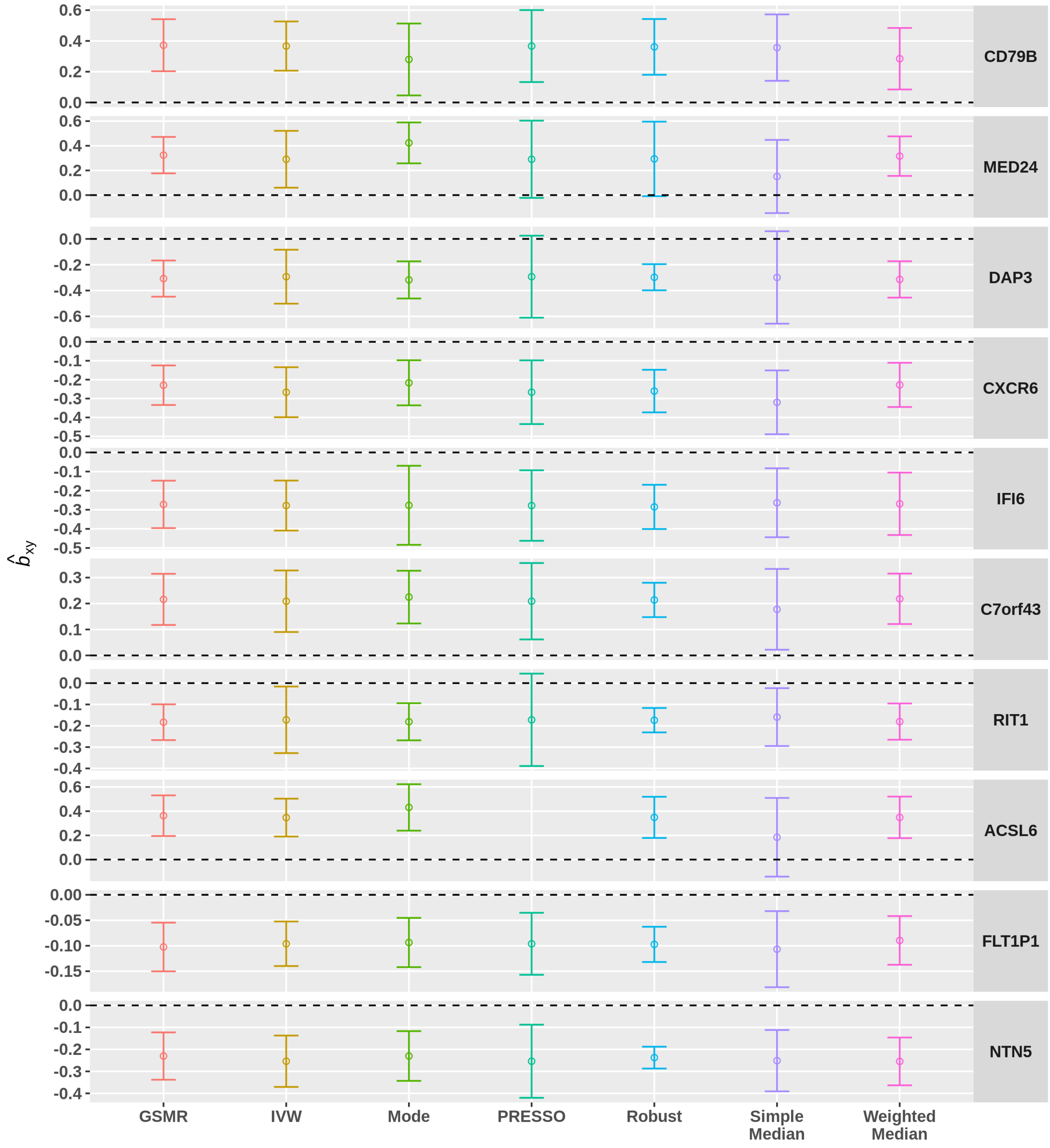

Supplementary Figure 24: Concordance of effect-size estimates using different Mendelian randomisation (MR) methods: GSMR, IVW, Mode, PRESSO, Robust, Simple Median, and Weighted Median. y-axis: effect-size estimates and 95% confidence interval. Significant RNA expression (eQTLgen) to GenOMICC GSMR results displayed (FDR < 0.01; n = 79).

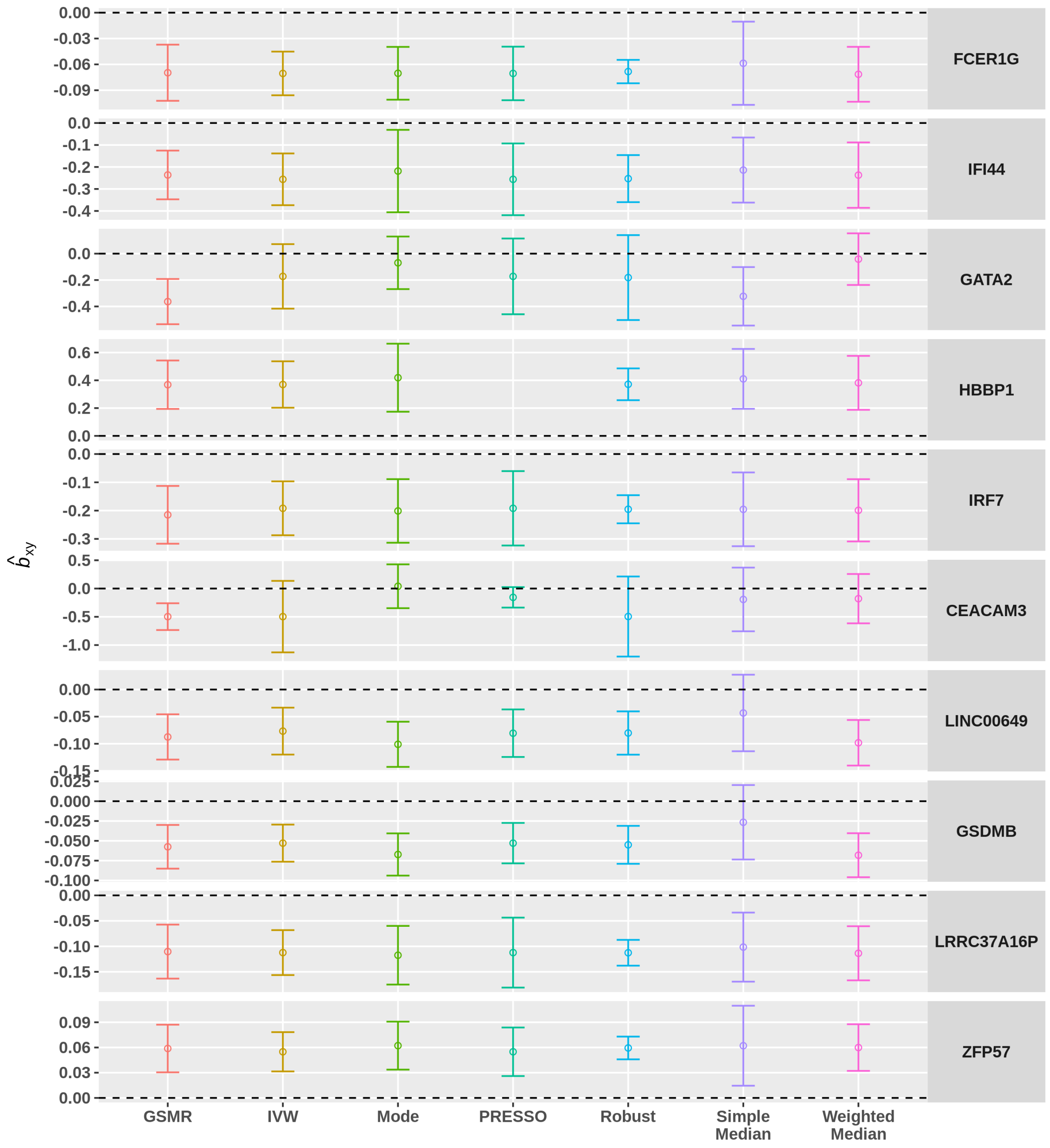

Supplementary Figure 25: Concordance of effect-size estimates using different Mendelian randomisation (MR) methods: GSMR, IVW, Mode, PRESSO, Robust, Simple Median, and Weighted Median. y-axis: effect-size estimates and 95% confidence interval. Significant RNA expression (eQTLgen) to GenOMICC GSMR results displayed (FDR < 0.01; n = 79).

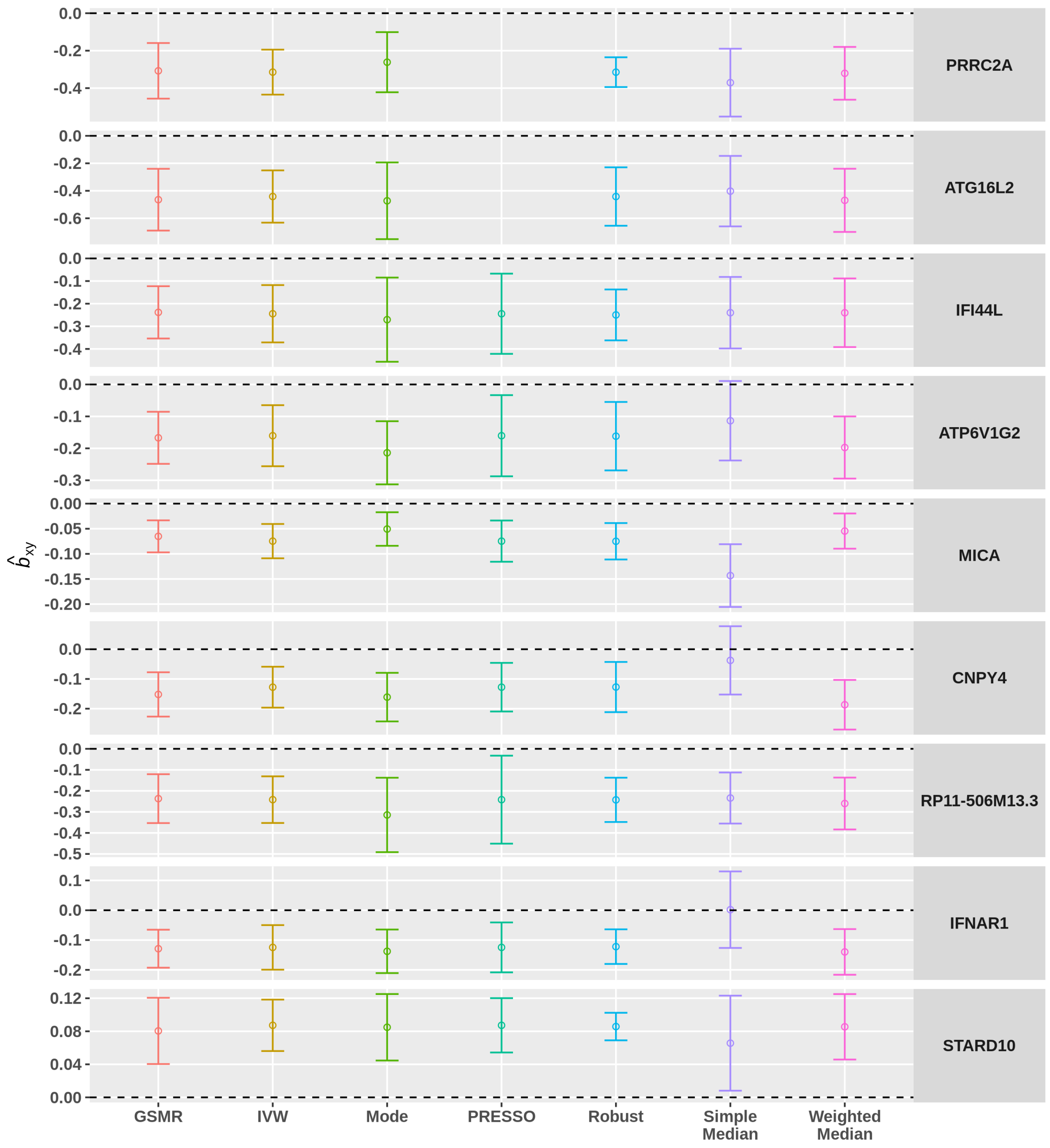

Supplementary Figure 26: Concordance of effect-size estimates using different Mendelian randomisation (MR) methods: GSMR, IVW, Mode, PRESSO, Robust, Simple Median, and Weighted Median. y-axis: effect-size estimates and 95% confidence interval. Significant RNA expression (eQTLgen) to GenOMICC GSMR results displayed (FDR < 0.01; n $= 79$).

10 Fine mapping

We have performed fine-mapping to compute credible sets of variants for the identified loci. In a small number of regions, the finemapping algorithm fails to converge, with quality controls pointing at the lack of a suitable linkage disequilibrium panel as a potential cause. Finemapping using meta analysis summary statistics from heterogenous cohorts, as is the case here, is difficult and remains an open problem with existing finemapping methods recently shown to perform poorly in this context.[9] While we note these limitations, we provide the obtained credible sets to aid any further functional work (Supplementary Figures 27-44, Supplementary Table 5).

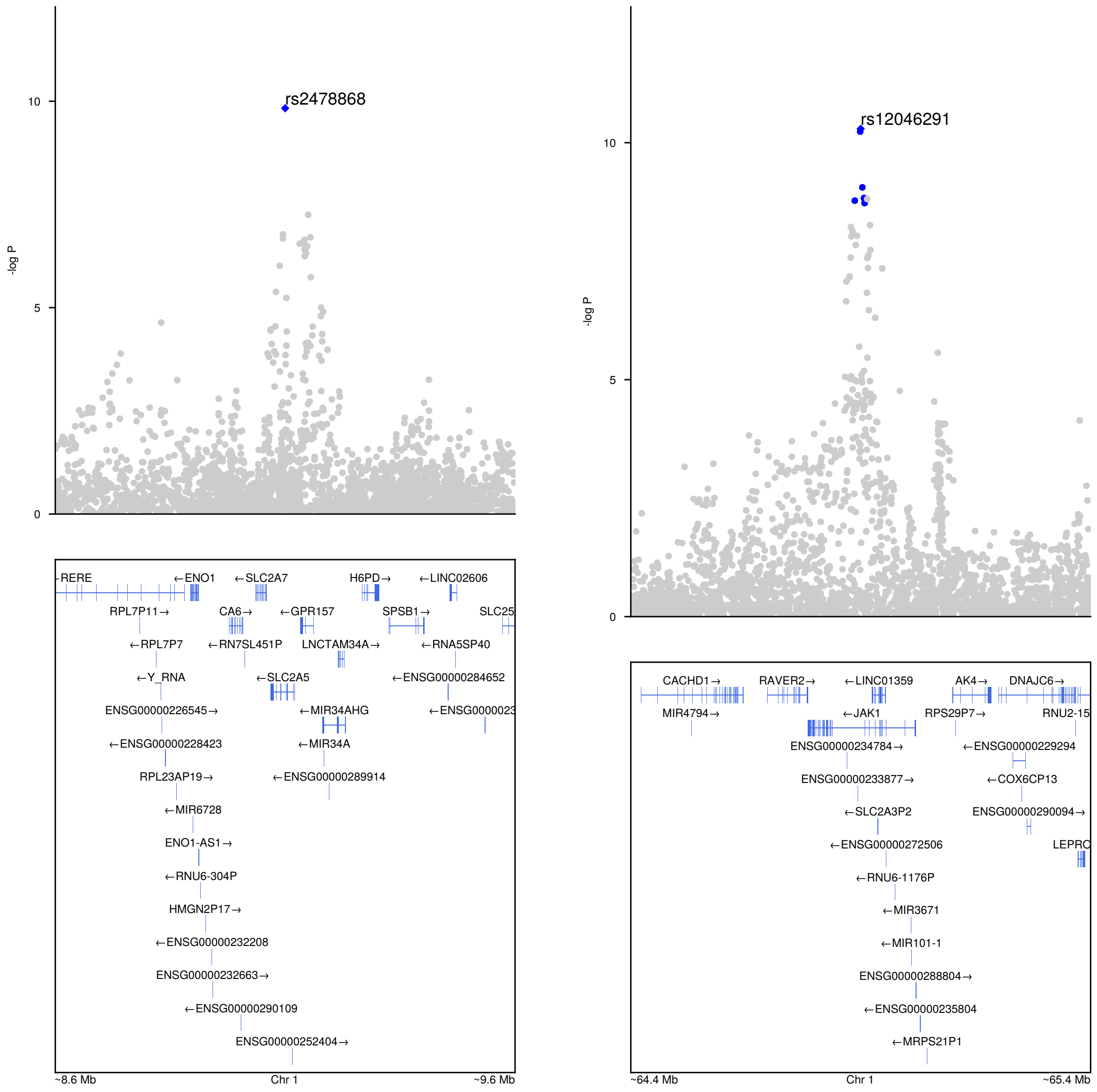

(a) (b)

Supplementary Figure 27: Fine mapping results obtained using SuSiE for regions surrounding lead variants. Lead variants are indicated from conditional analysis are indicated by diamonds. Variants contained in 95% credible sets are colored by set membership. Protein coding genes are shown in the lower panels.

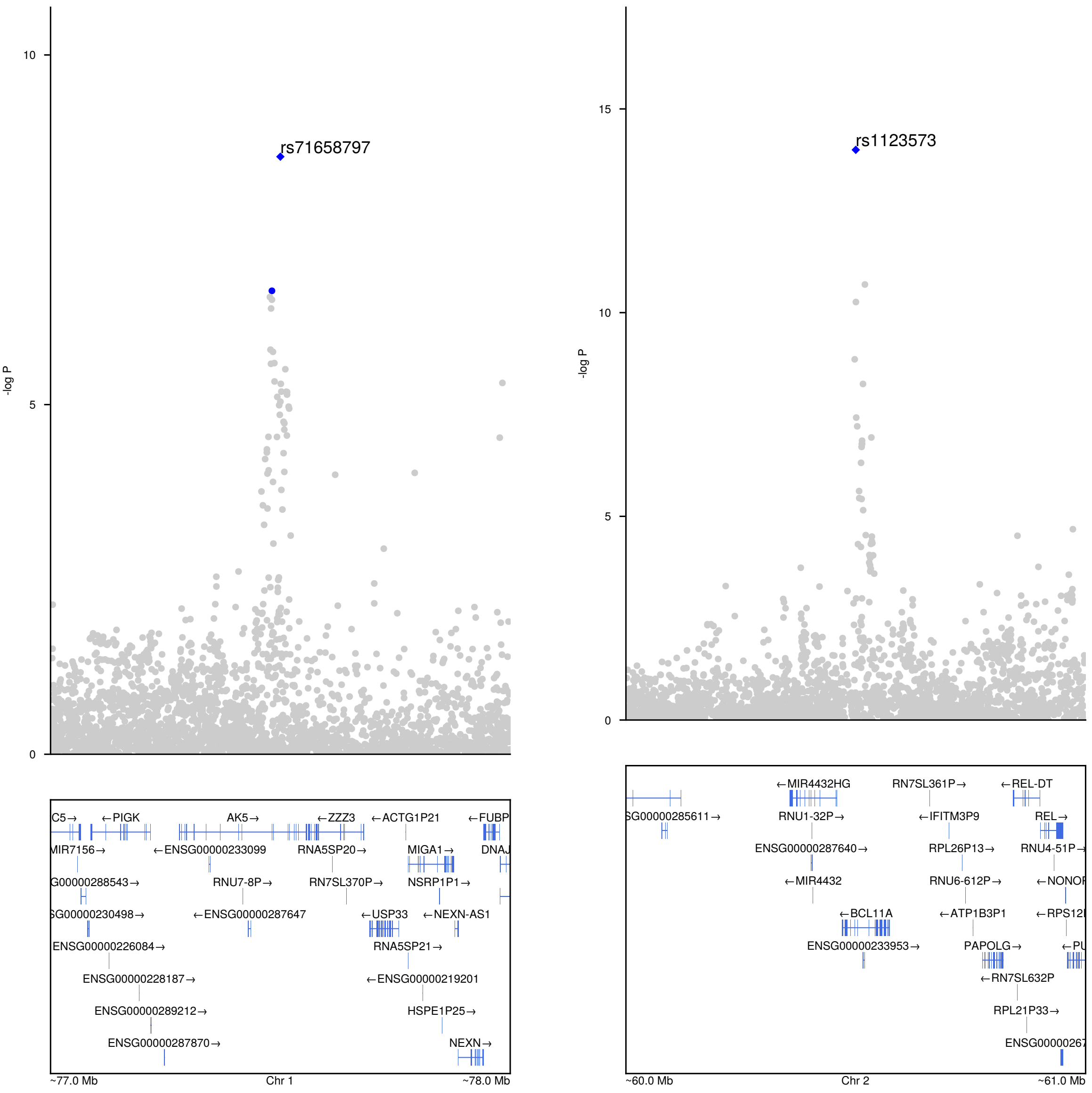

(a) (b)

Supplementary Figure 28: Fine mapping results obtained using SuSiE for regions surrounding lead variants. Lead variants are indicated from conditional analysis are indicated by diamonds. Variants contained in 95% credible sets are colored by set membership. Protein coding genes are shown in the lower panels.

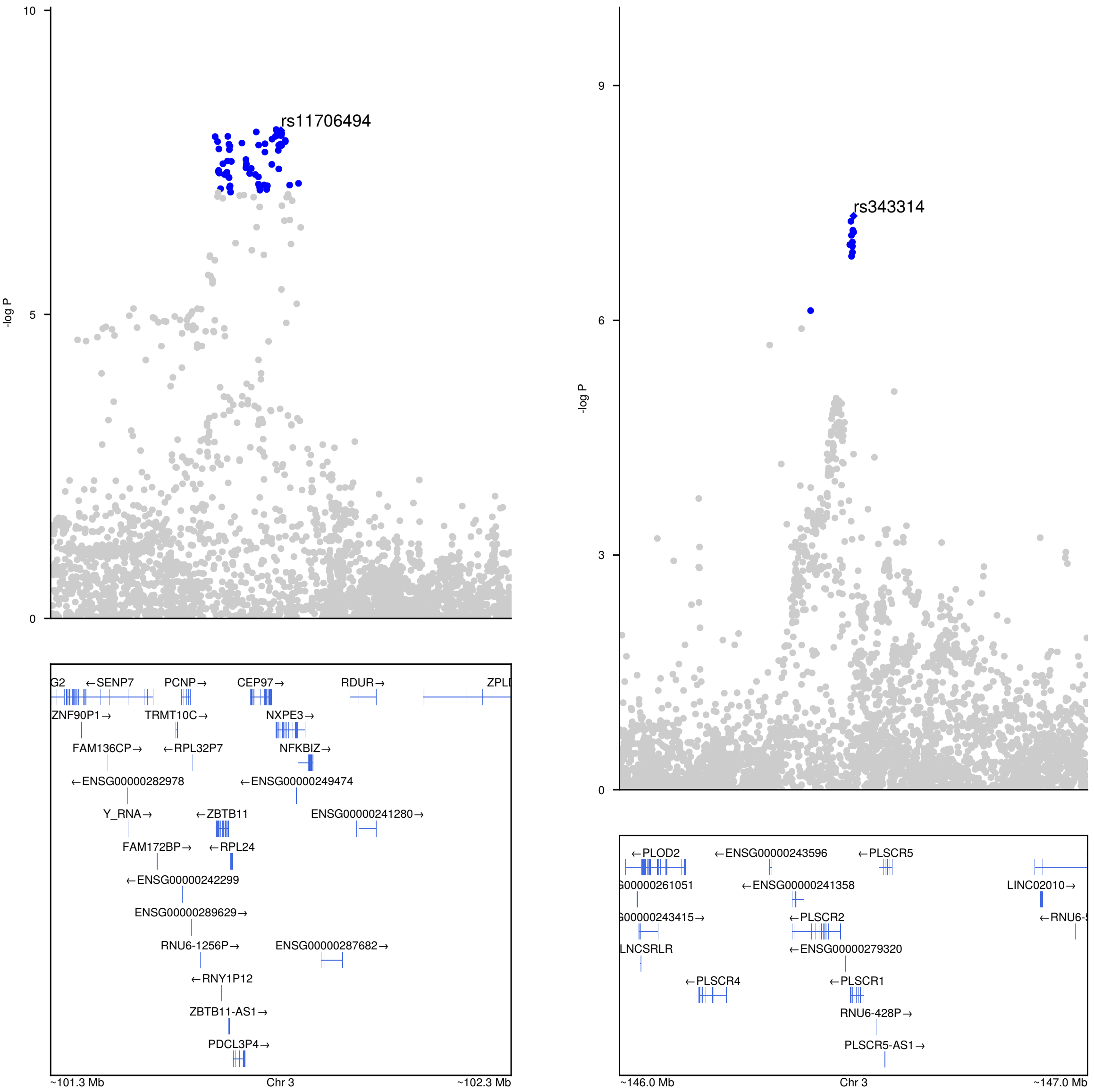

(a) (b)

Supplementary Figure 29: Fine mapping results obtained using SuSiE for regions surrounding lead variants. Lead variants are indicated from conditional analysis are indicated by diamonds. Variants contained in 95% credible sets are colored by set membership. Protein coding genes are shown in the lower panels.

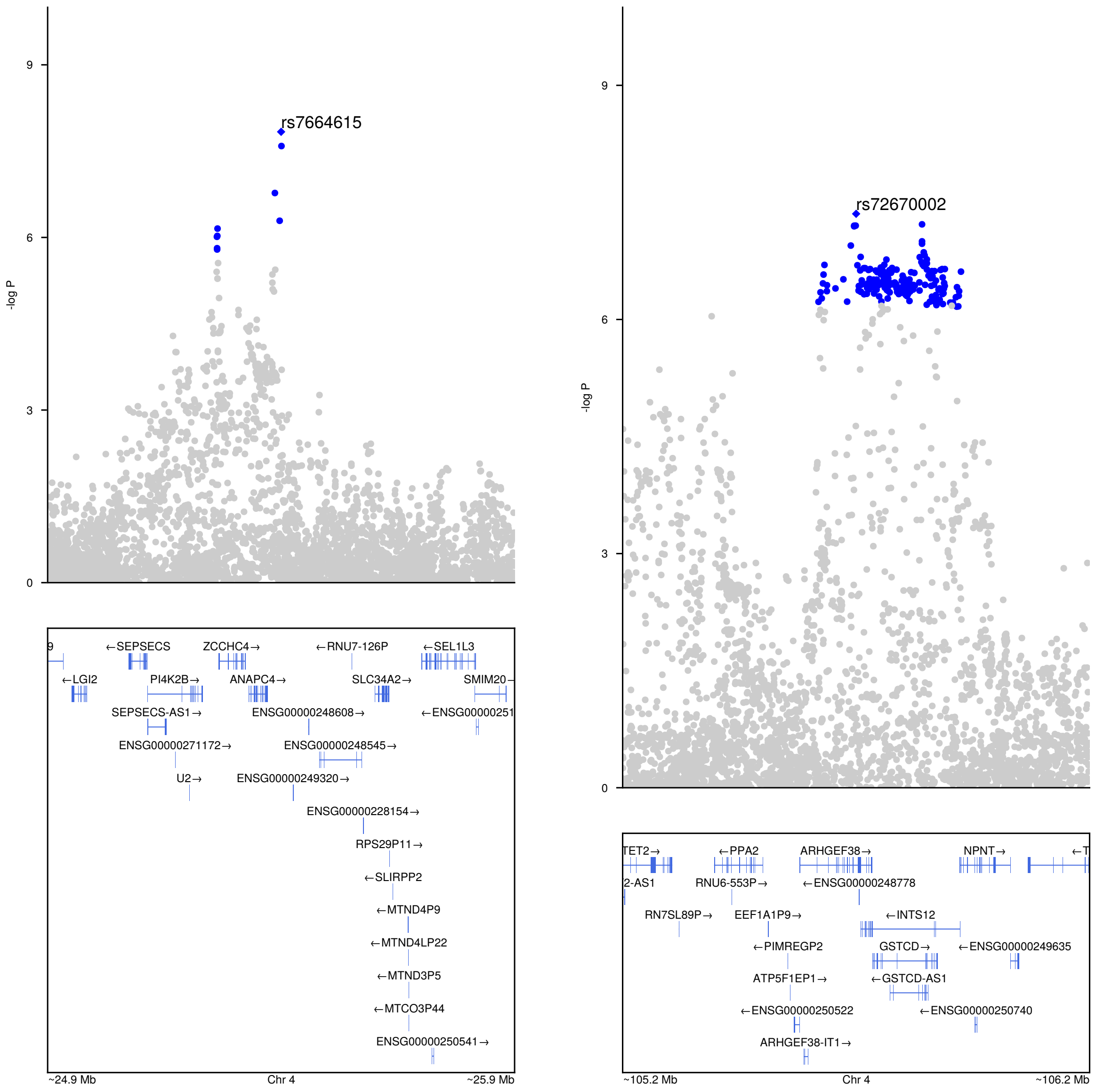

(a) (b)

Supplementary Figure 30: Fine mapping results obtained using SuSiE for regions surrounding lead variants. Lead variants are indicated from conditional analysis are indicated by diamonds. Variants contained in 95% credible sets are colored by set membership. Protein coding genes are shown in the lower panels.

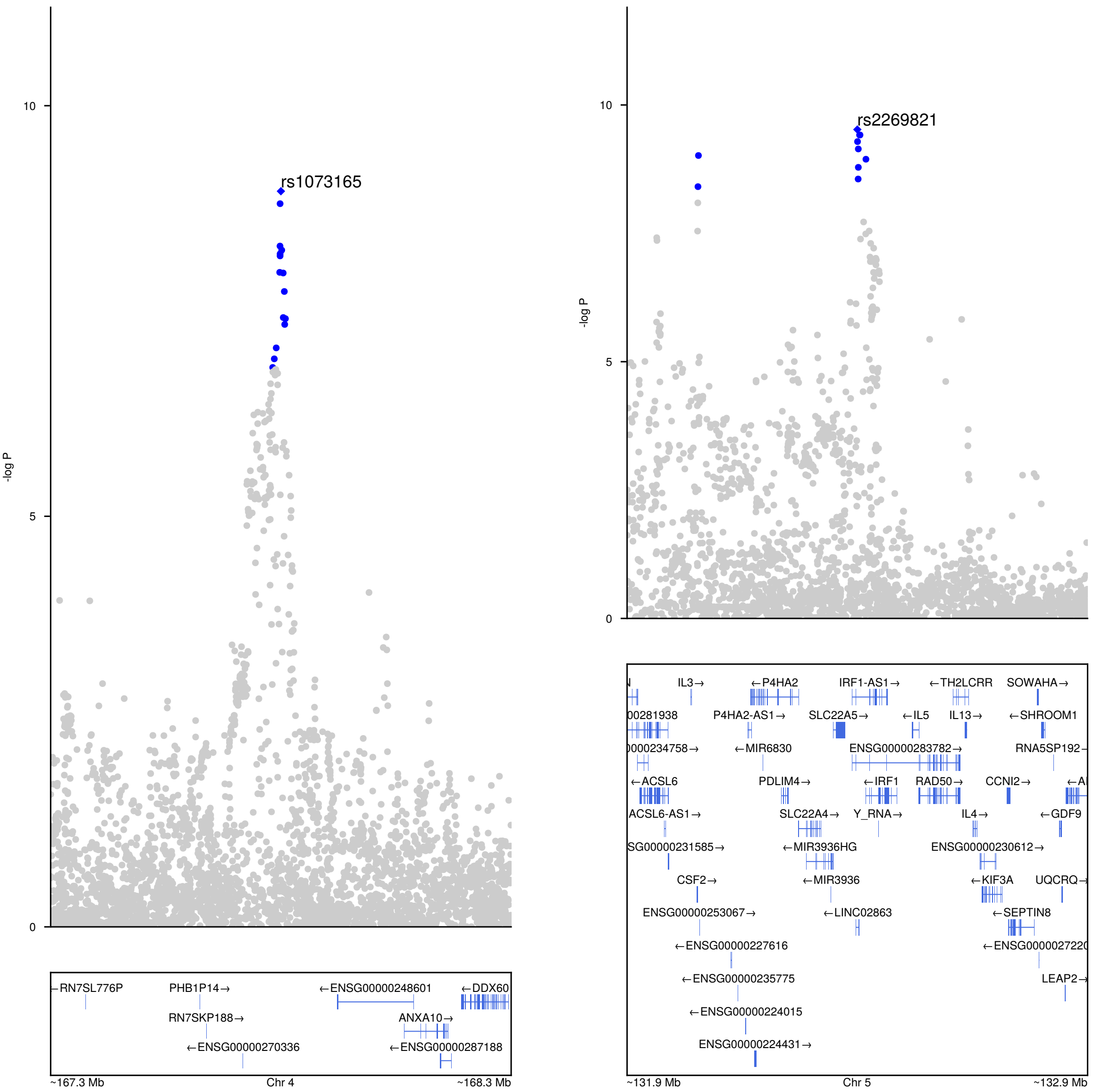

(a) (b)

Supplementary Figure 31: Fine mapping results obtained using SuSiE for regions surrounding lead variants. Lead variants are indicated from conditional analysis are indicated by diamonds. Variants contained in 95% credible sets are colored by set membership. Protein coding genes are shown in the lower panels.

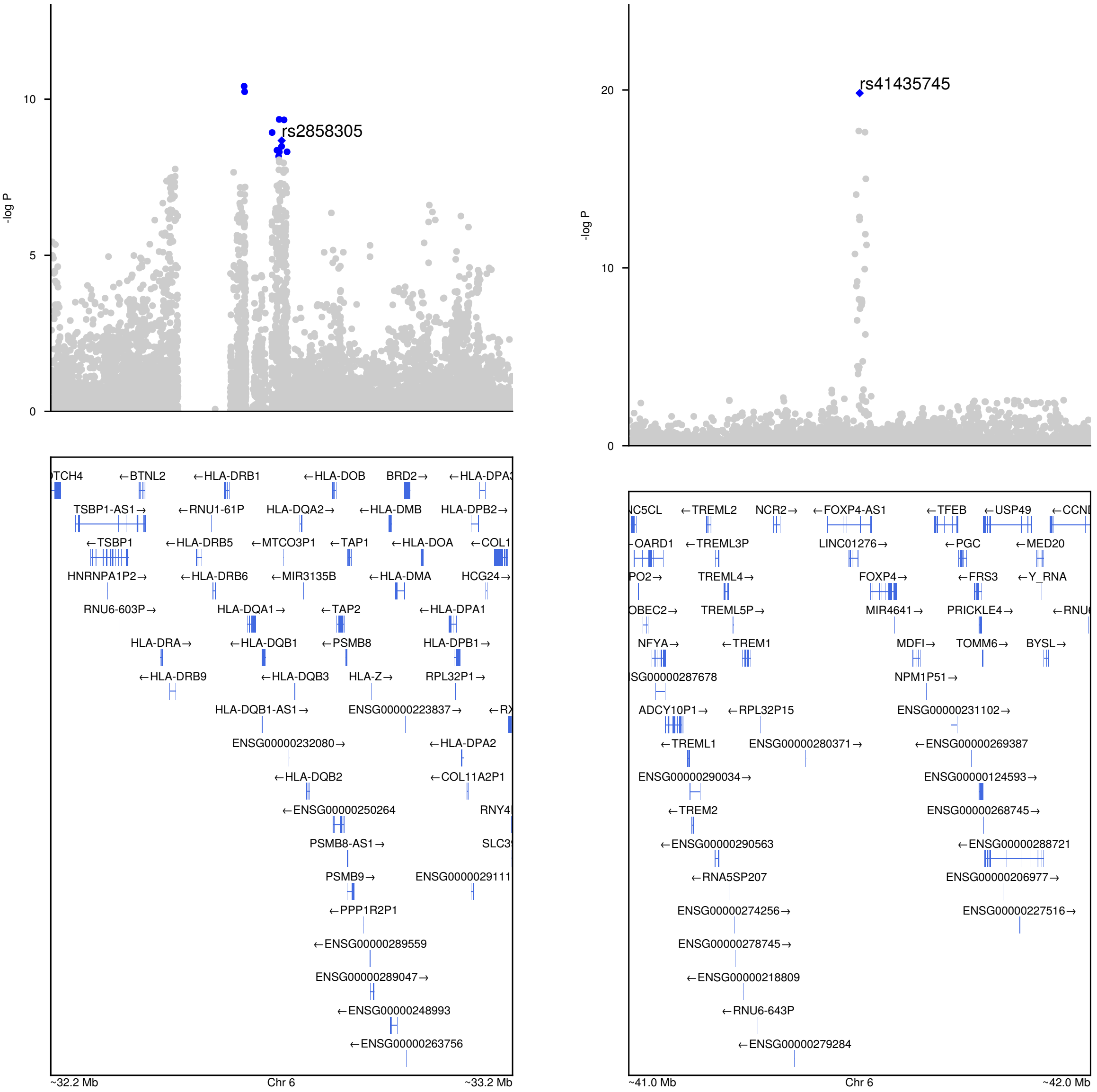

(a) (b)

Supplementary Figure 32: Fine mapping results obtained using SuSiE for regions surrounding lead variants. Lead variants are indicated from conditional analysis are indicated by diamonds. Variants contained in 95% credible sets are colored by set membership. Protein coding genes are shown in the lower panels.

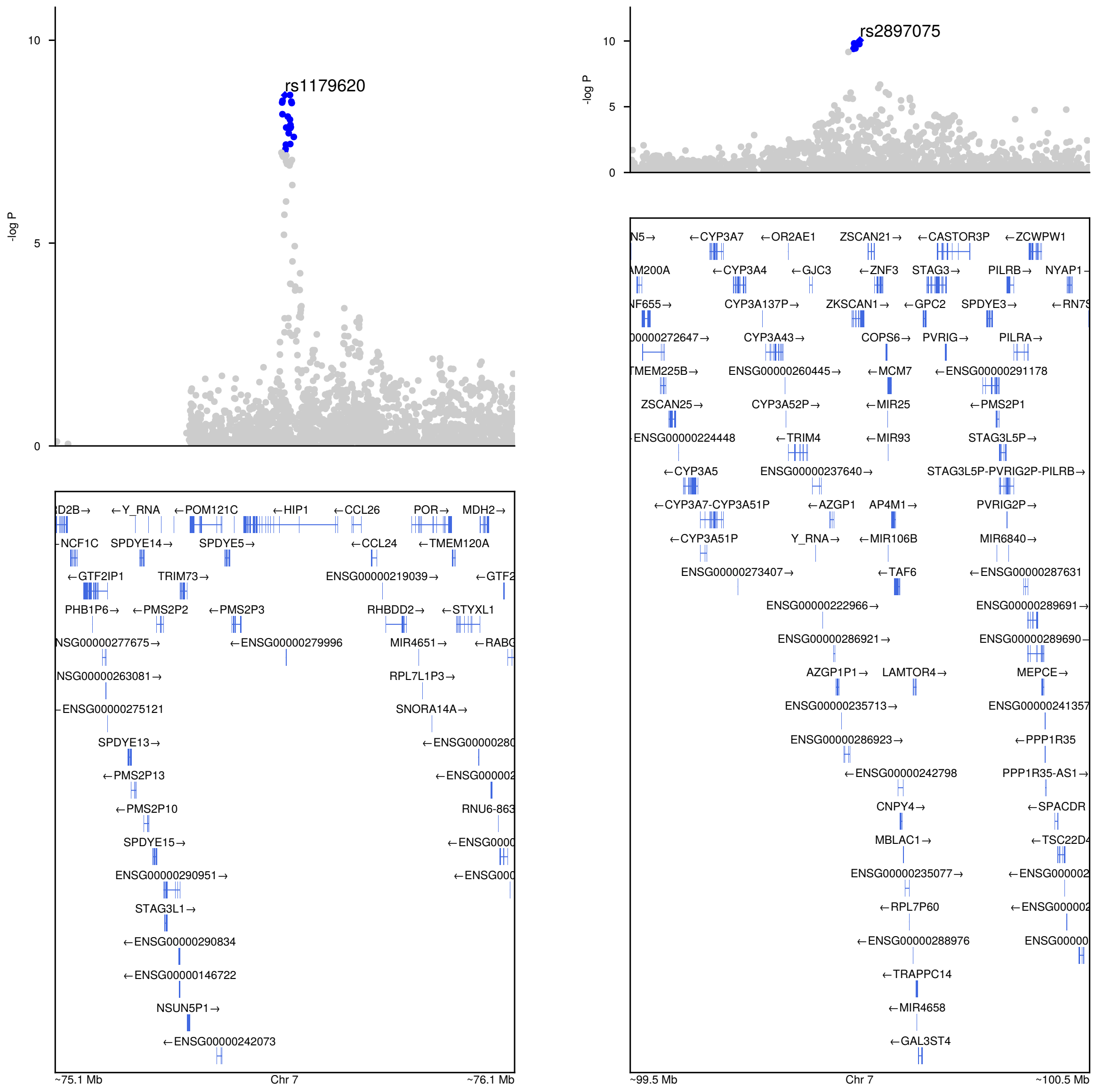

(a)

(b)

Supplementary Figure 33: Fine mapping results obtained using SuSiE for regions surrounding lead variants. Lead variants are indicated from conditional analysis are indicated by diamonds. Variants contained in 95% credible sets are colored by set membership. Protein coding genes are shown in the lower panels.

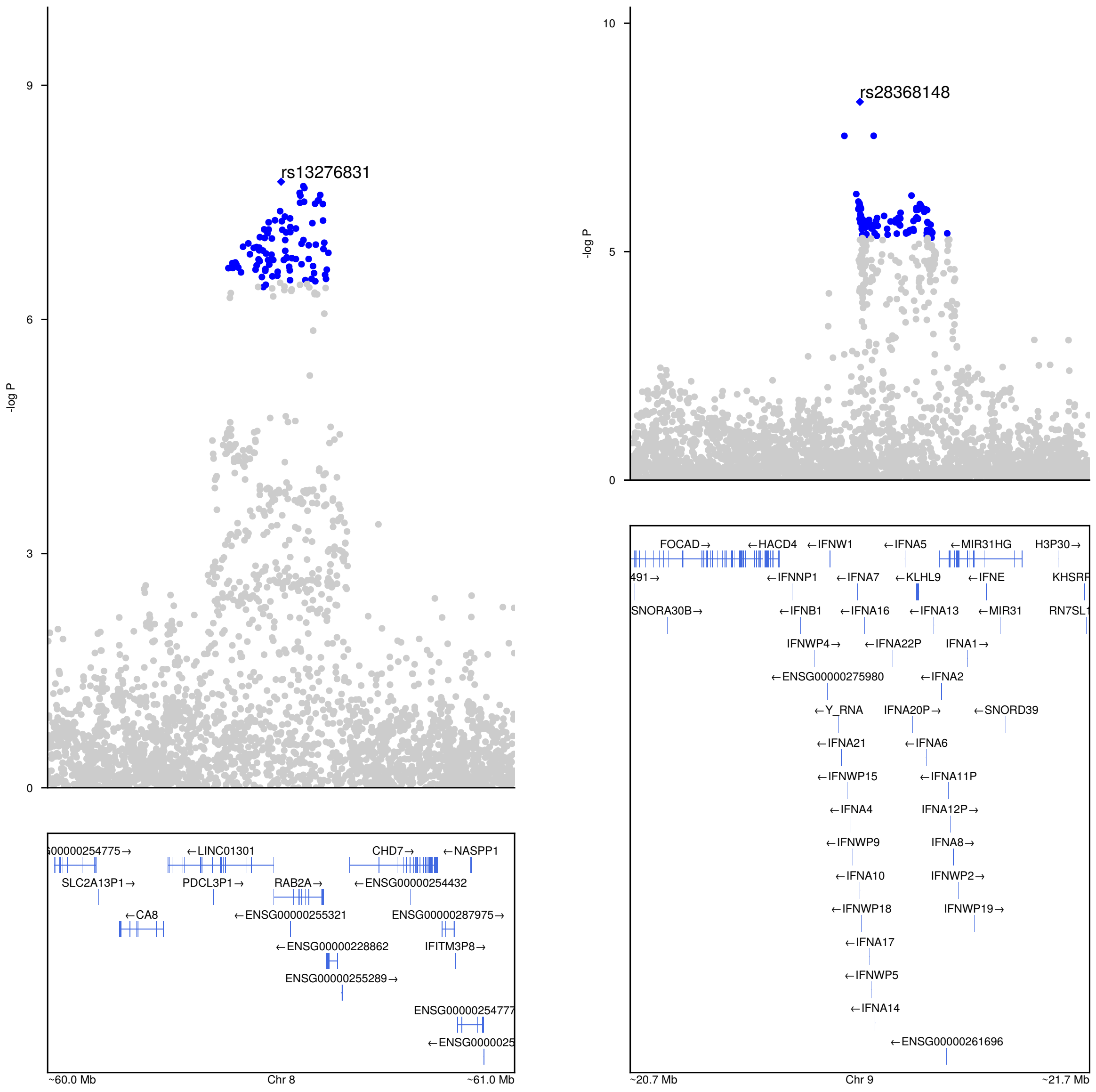

(a) (b)

Supplementary Figure 34: Fine mapping results obtained using SuSiE for regions surrounding lead variants. Lead variants are indicated from conditional analysis are indicated by diamonds. Variants contained in 95% credible sets are colored by set membership. Protein coding genes are shown in the lower panels.

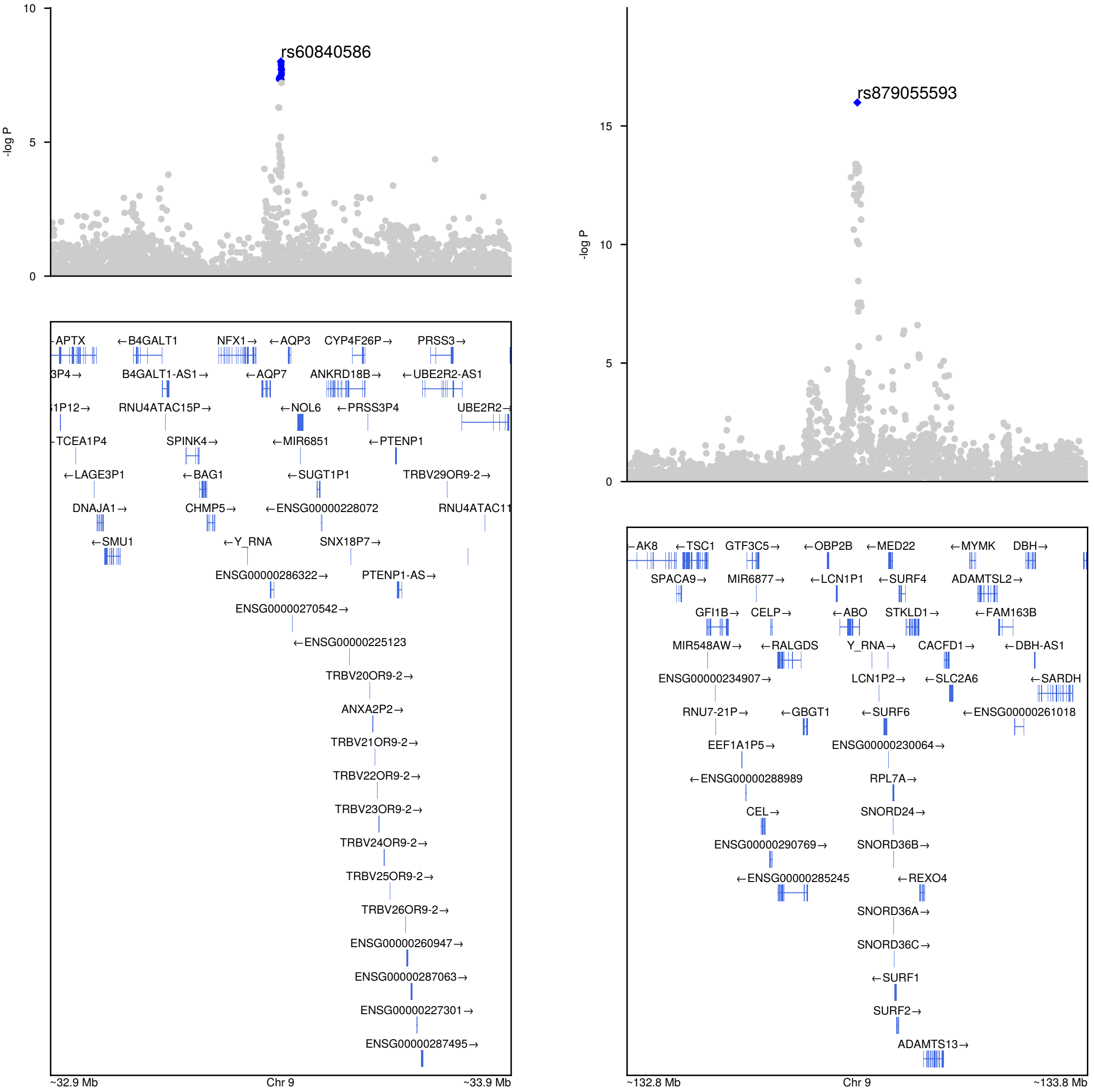

(a) (b)

Supplementary Figure 35: Fine mapping results obtained using SuSiE for regions surrounding lead variants. Lead variants are indicated from conditional analysis are indicated by diamonds. Variants contained in 95% credible sets are colored by set membership. Protein coding genes are shown in the lower panels.

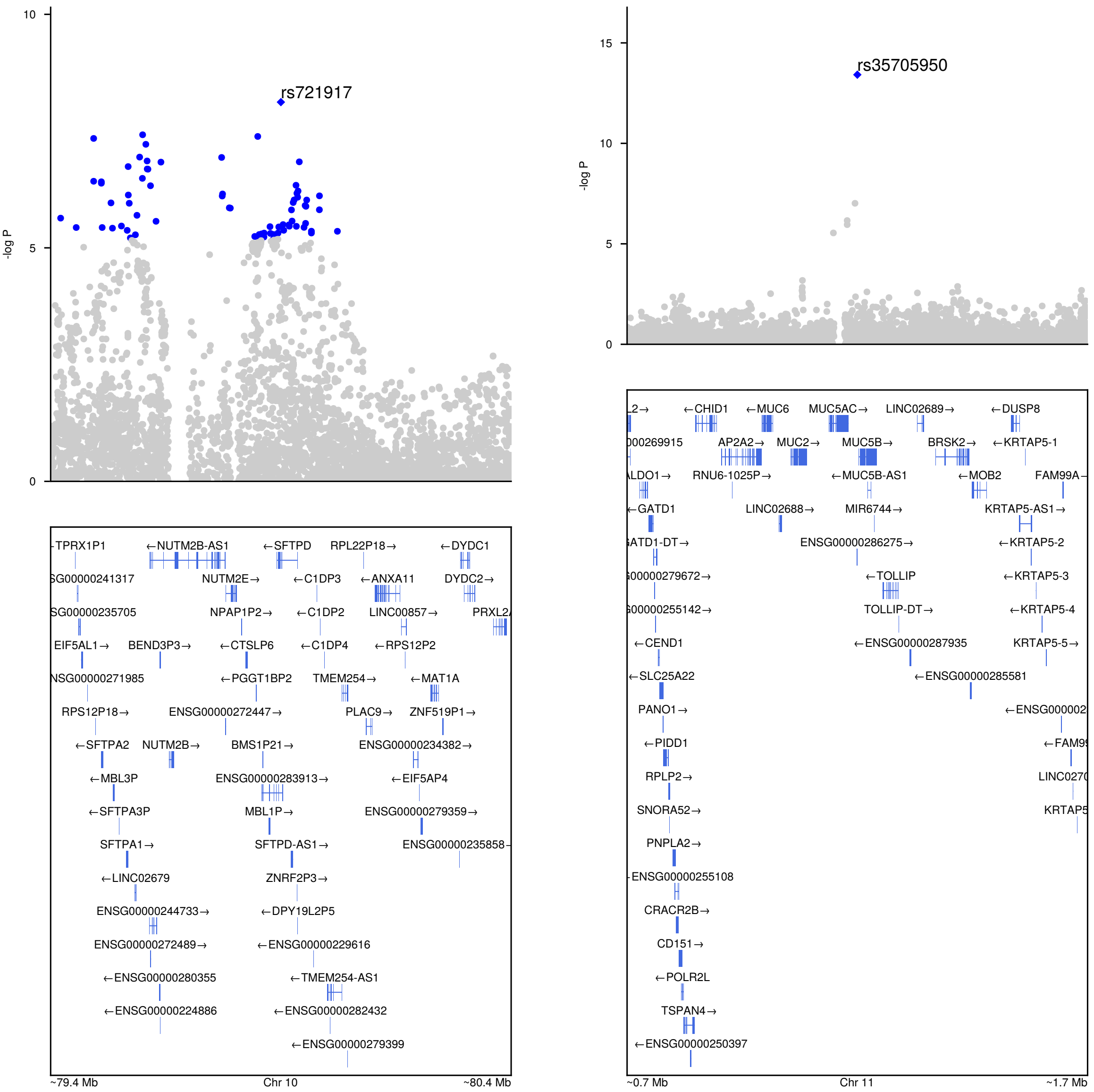

(a) (b)

Supplementary Figure 36: Fine mapping results obtained using SuSiE for regions surrounding lead variants. Lead variants are indicated from conditional analysis are indicated by diamonds. Variants contained in 95% credible sets are colored by set membership. Protein coding genes are shown in the lower panels.

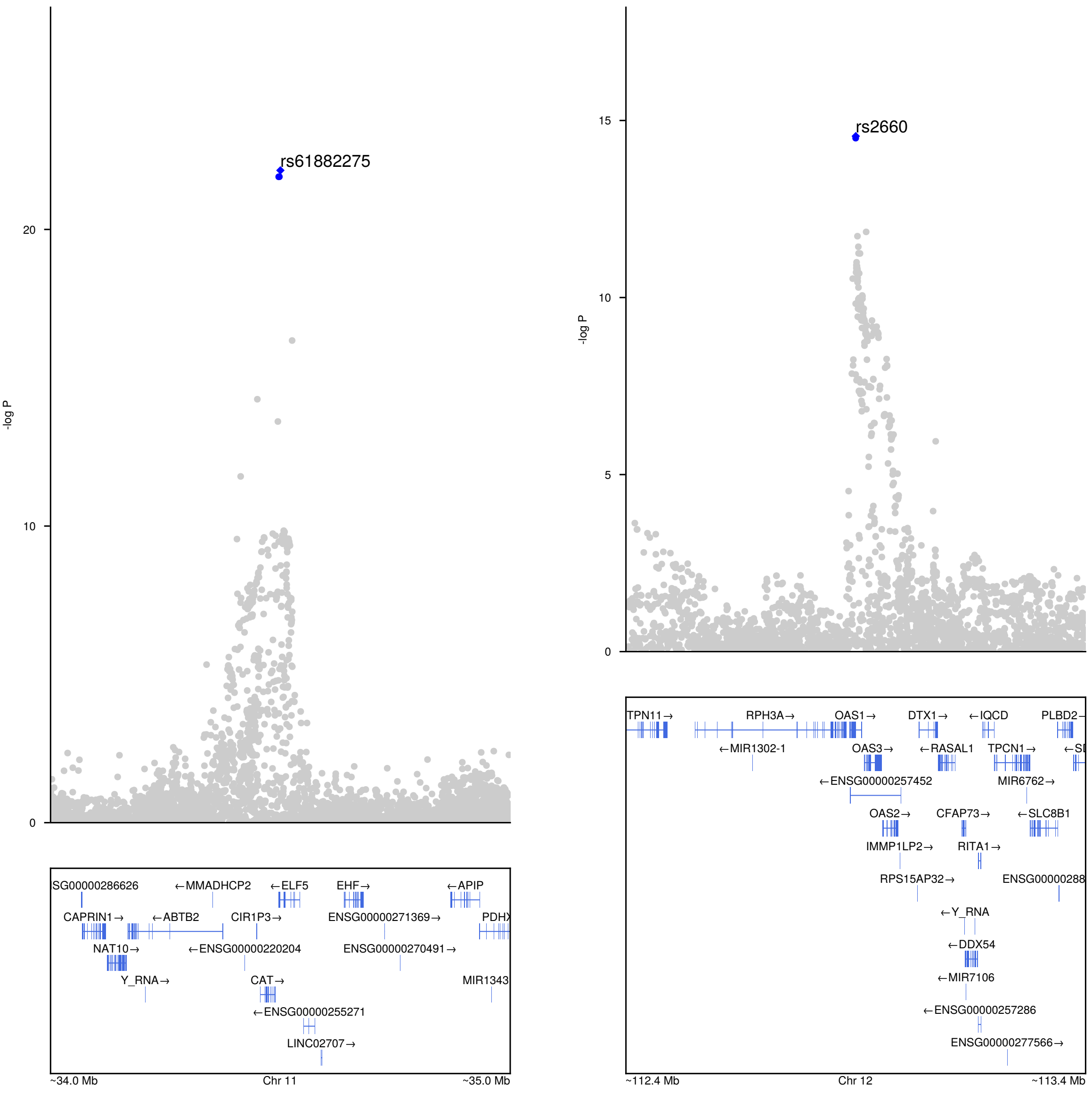

(a) (b)

Supplementary Figure 37: Fine mapping results obtained using SuSiE for regions surrounding lead variants. Lead variants are indicated from conditional analysis are indicated by diamonds. Variants contained in 95% credible sets are colored by set membership. Protein coding genes are shown in the lower panels.

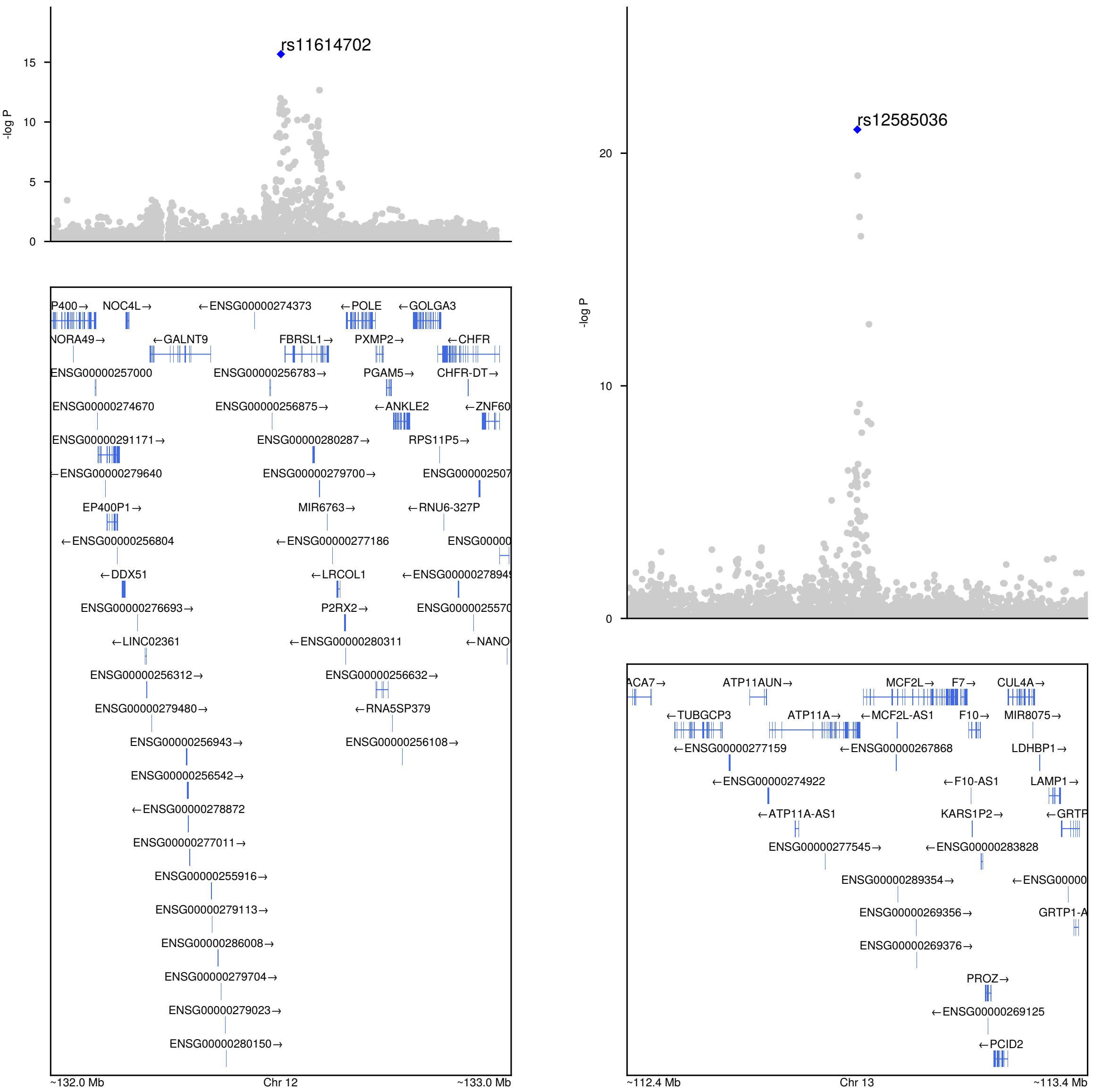

(a) (b)

Supplementary Figure 38: Fine mapping results obtained using SuSiE for regions surrounding lead variants. Lead variants are indicated from conditional analysis are indicated by diamonds. Variants contained in 95% credible sets are colored by set membership. Protein coding genes are shown in the lower panels.

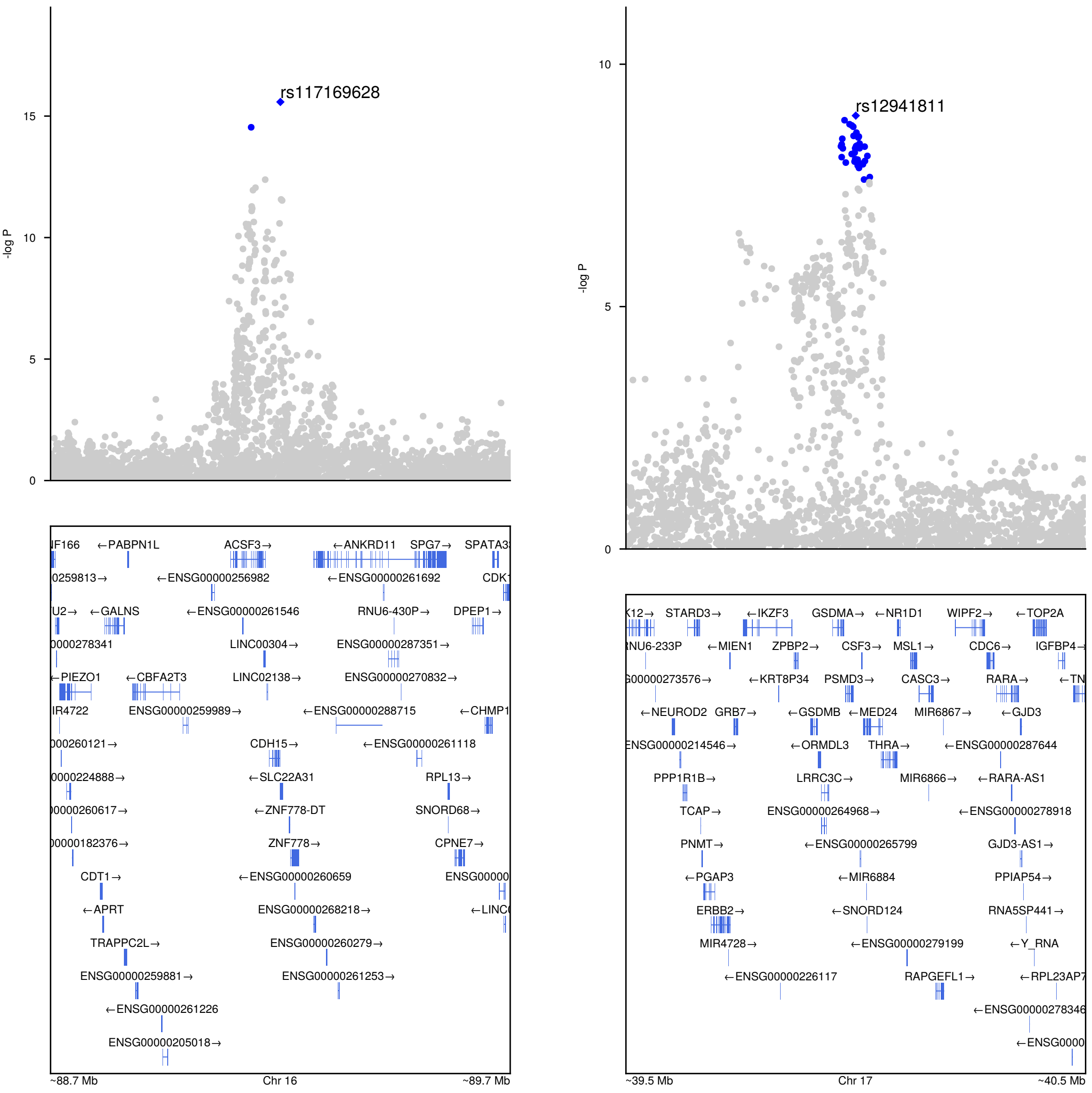

(a) (b)

Supplementary Figure 39: Fine mapping results obtained using SuSiE for regions surrounding lead variants. Lead variants are indicated from conditional analysis are indicated by diamonds. Variants contained in 95% credible sets are colored by set membership. Protein coding genes are shown in the lower panels.

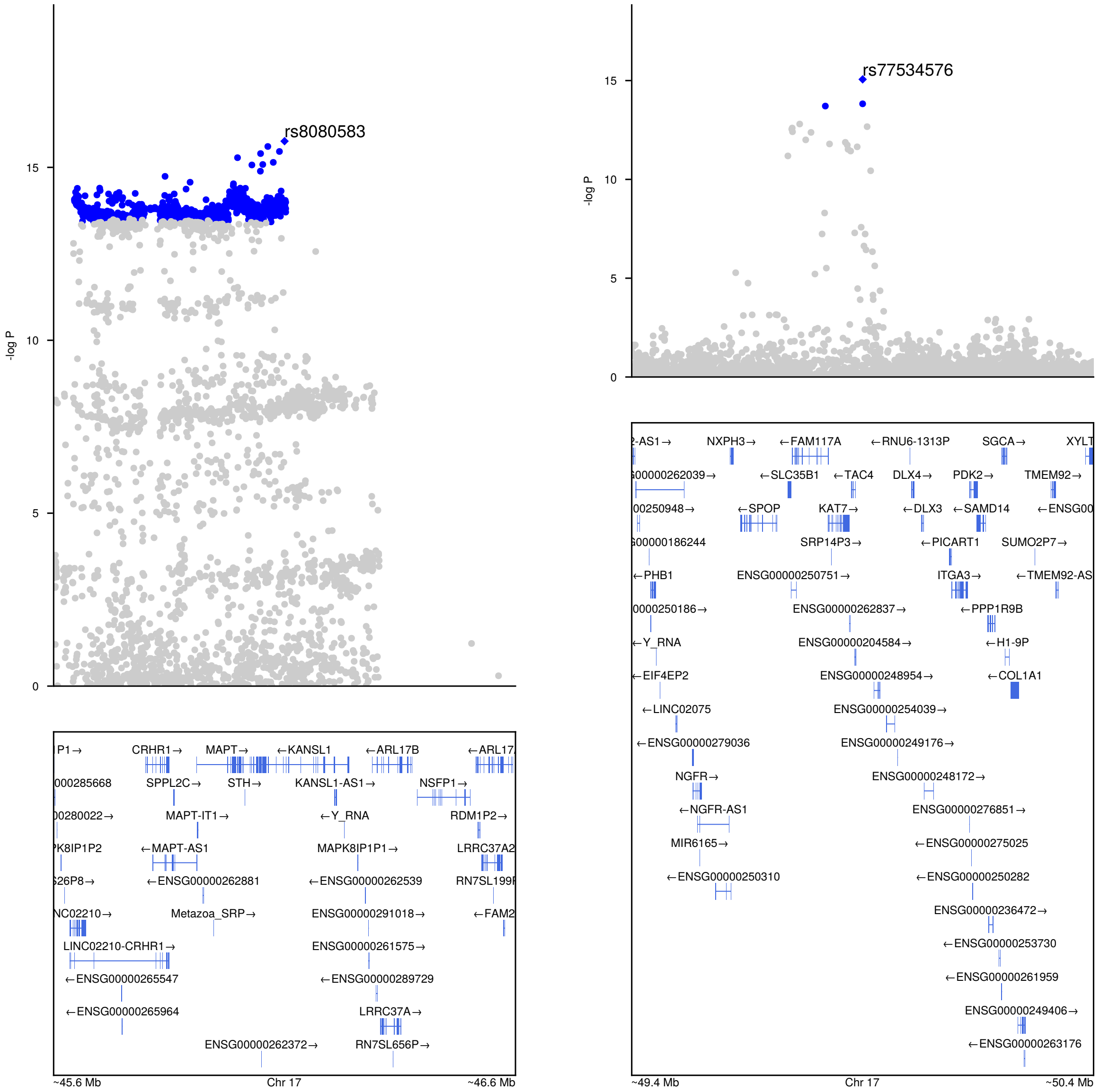

(a) (b)

Supplementary Figure 40: Fine mapping results obtained using SuSiE for regions surrounding lead variants. Lead variants are indicated from conditional analysis are indicated by diamonds. Variants contained in 95% credible sets are colored by set membership. Protein coding genes are shown in the lower panels.

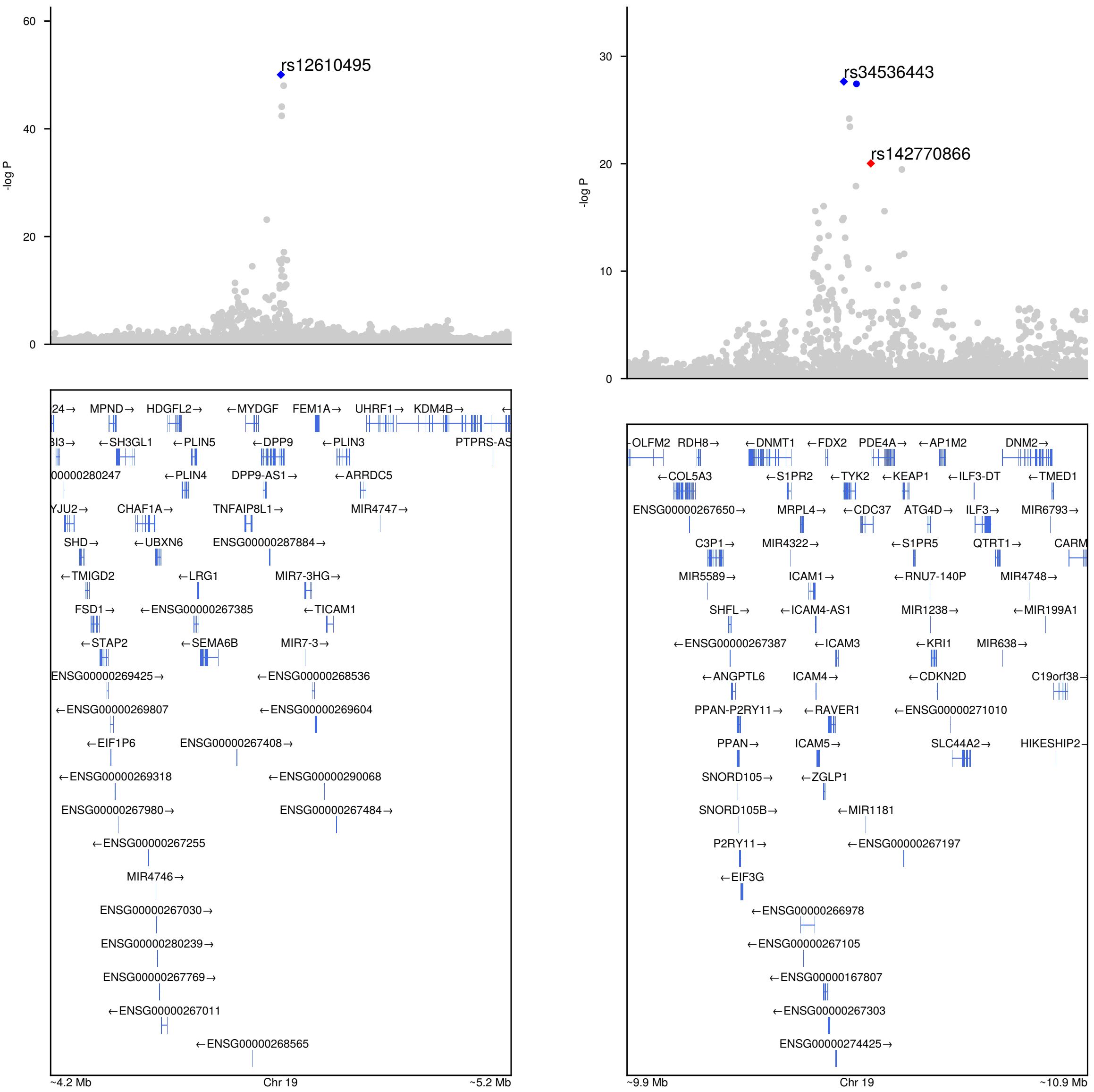

(a) (b)

Supplementary Figure 41: Fine mapping results obtained using SuSiE for regions surrounding lead variants. Lead variants are indicated from conditional analysis are indicated by diamonds. Variants contained in 95% credible sets are colored by set membership. Protein coding genes are shown in the lower panels.

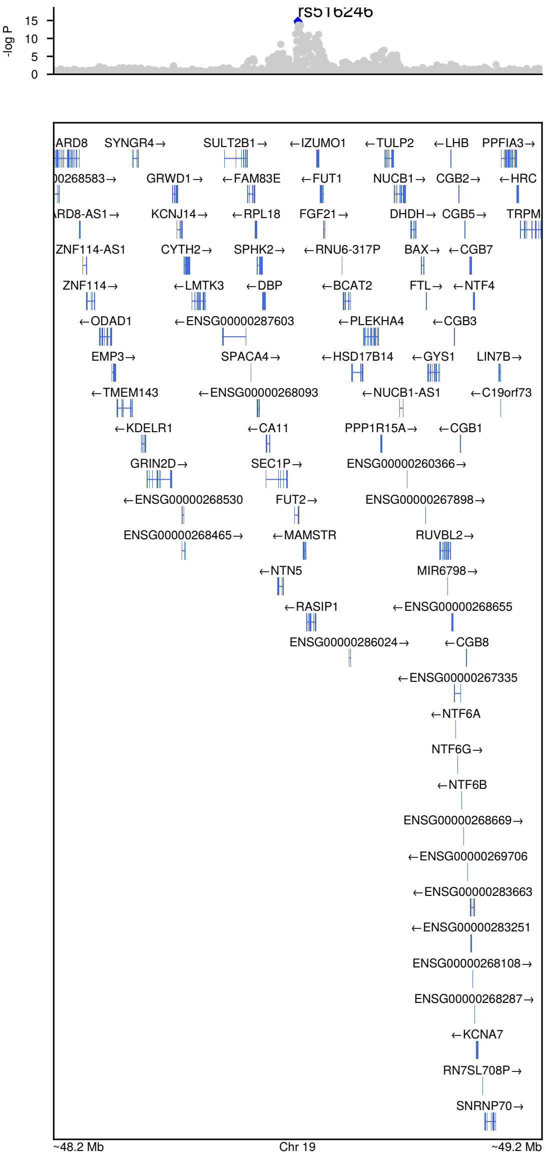

(a)

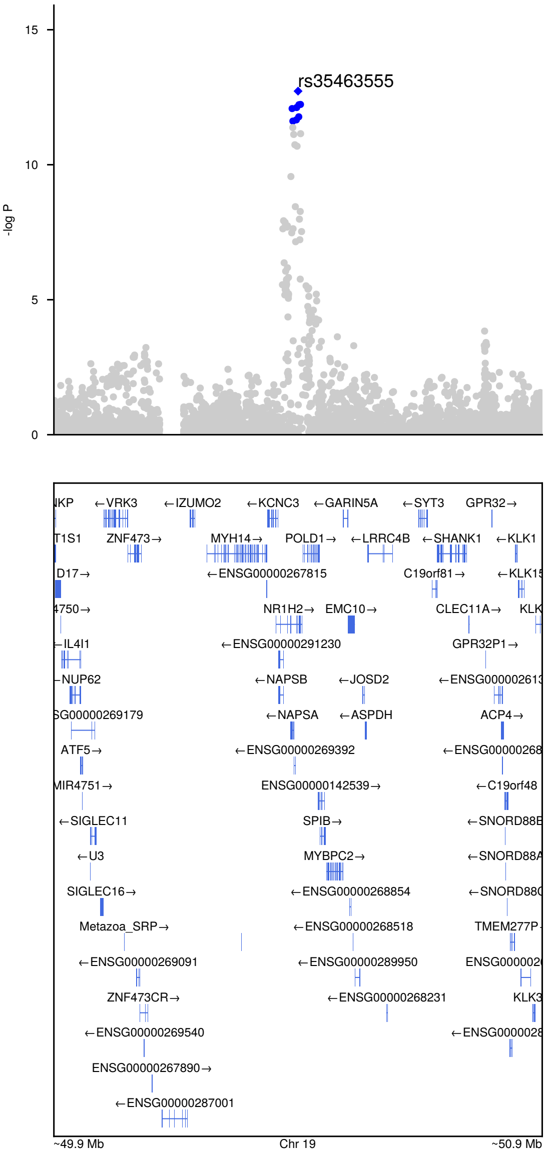

(b)

Supplementary Figure 42: Fine mapping results obtained using SuSiE for regions surrounding lead variants. Lead variants are indicated from conditional analysis are indicated by diamonds. Variants contained in 95% credible sets are colored by set membership. Protein coding genes are shown in the lower panels.

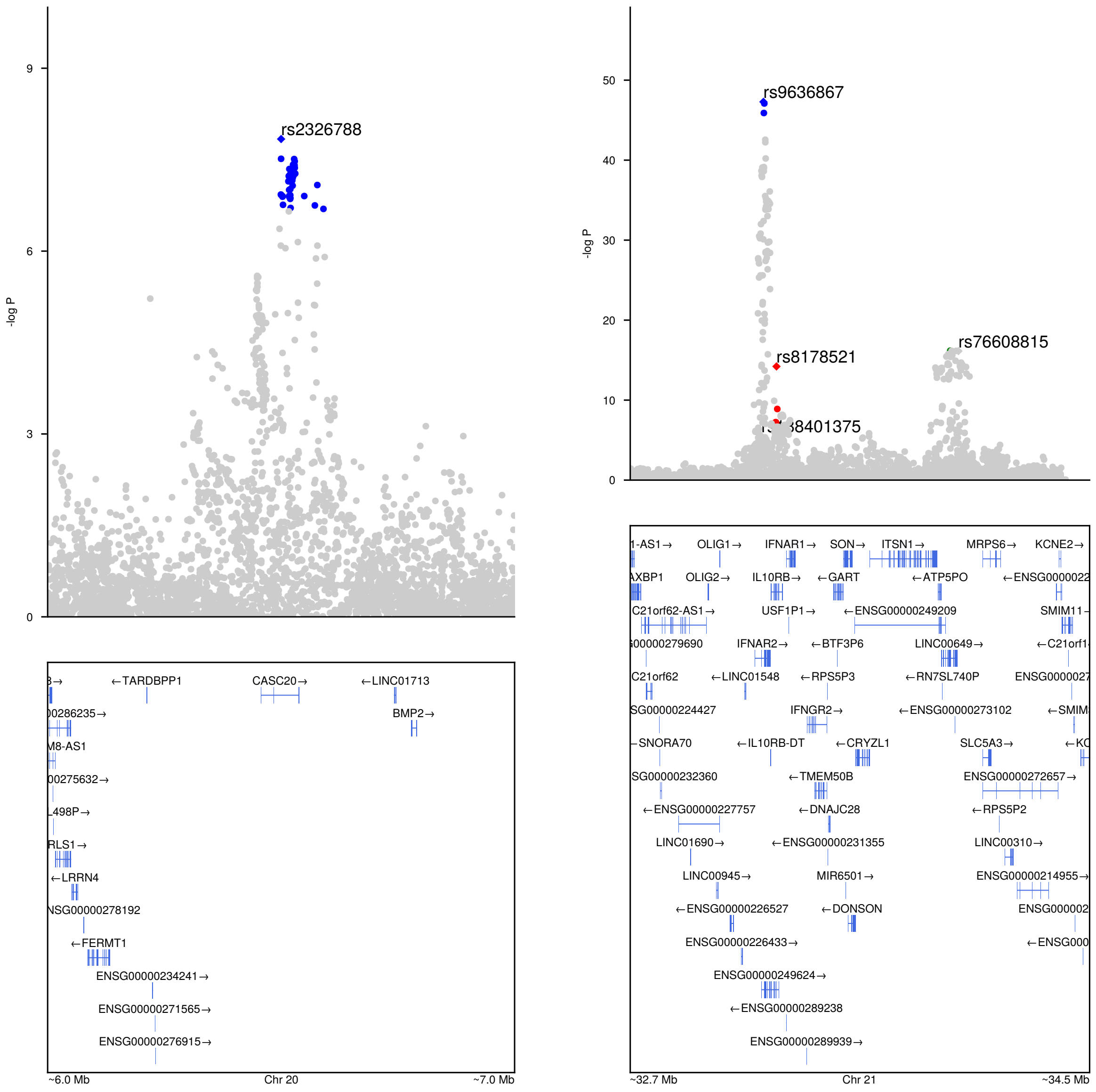

(a) (b)

Supplementary Figure 43: Fine mapping results obtained using SuSiE for regions surrounding lead variants. Lead variants are indicated from conditional analysis are indicated by diamonds. Variants contained in 95% credible sets are colored by set membership. Protein coding genes are shown in the lower panels.

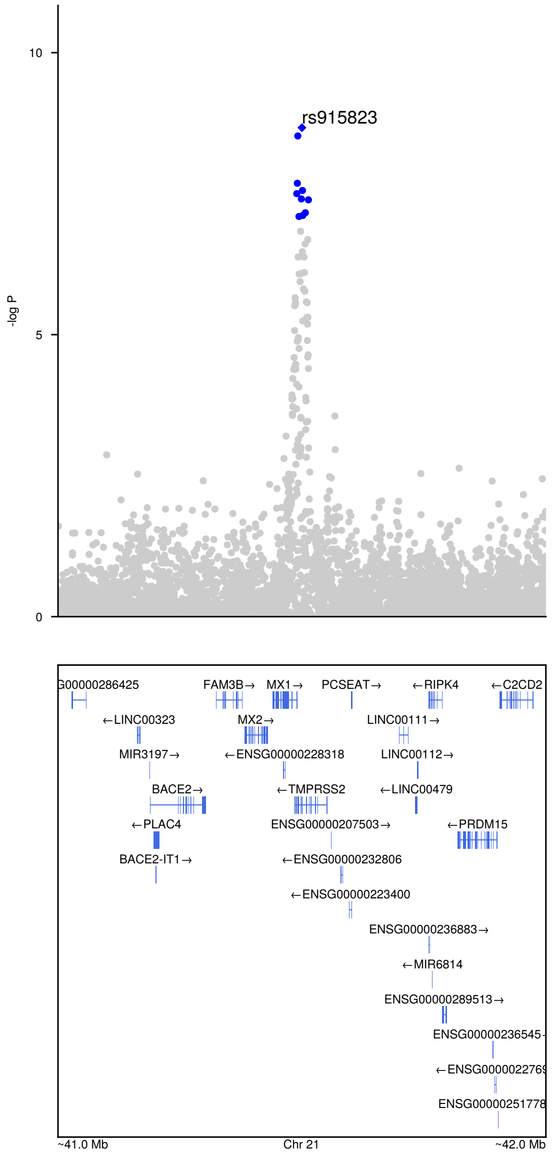

(a)

Supplementary Figure 44: Fine mapping results obtained using SuSiE for regions surrounding lead variants. Lead variants are indicated from conditional analysis are indicated by diamonds. Variants contained in 95% credible sets are colored by set membership. Protein coding genes are shown in the lower panels.

11 Change in allele frequency over time

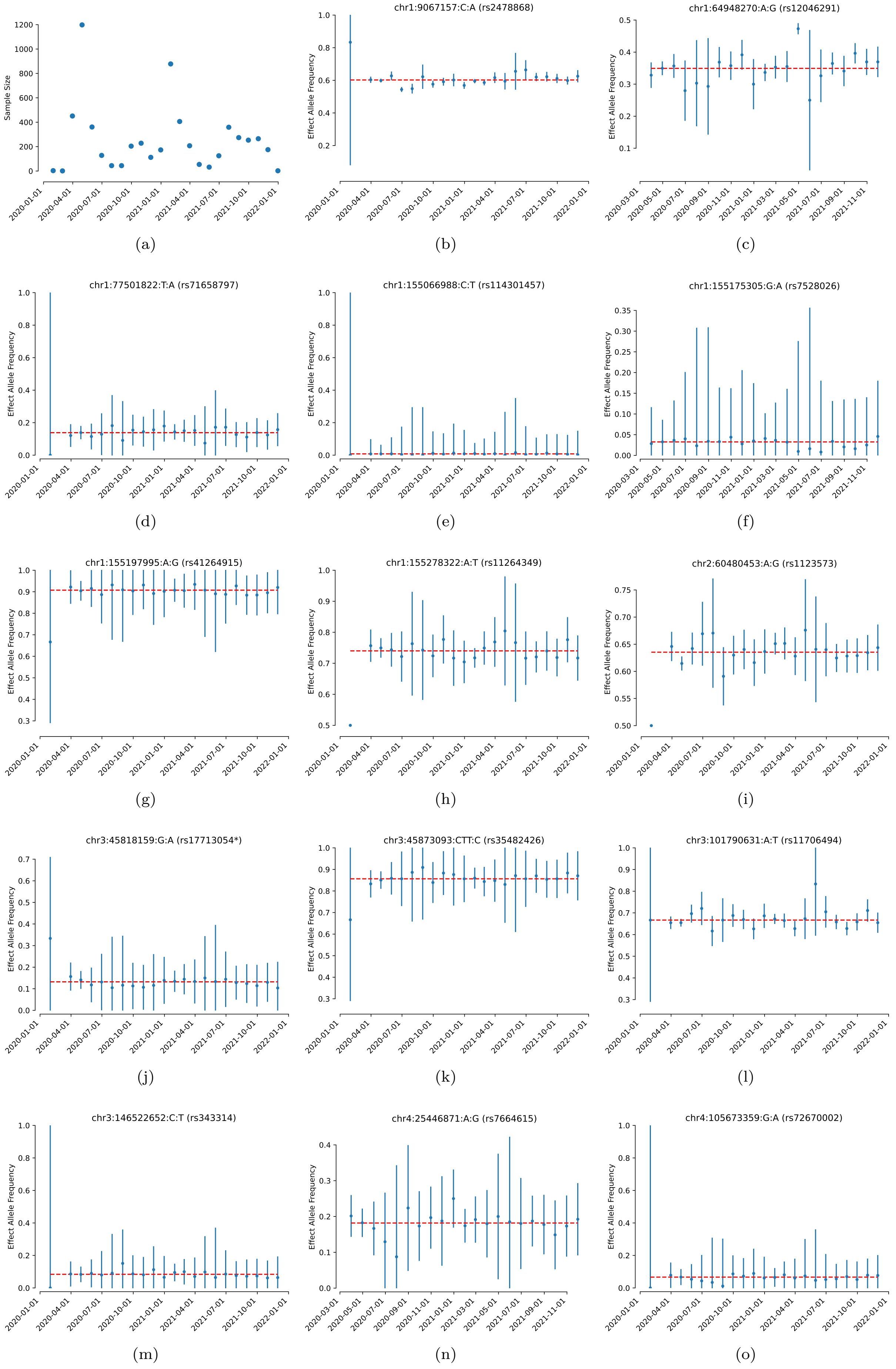

Supplementary Figure 45: Effect allele frequency (EAF +/- 95%CI) in critically-ill group (GenOMICC UK only) by month of for all lead variants. Dashed red line indicates EAF in the whole cohort. Sample sizes for each month are shown in panel (a) above.

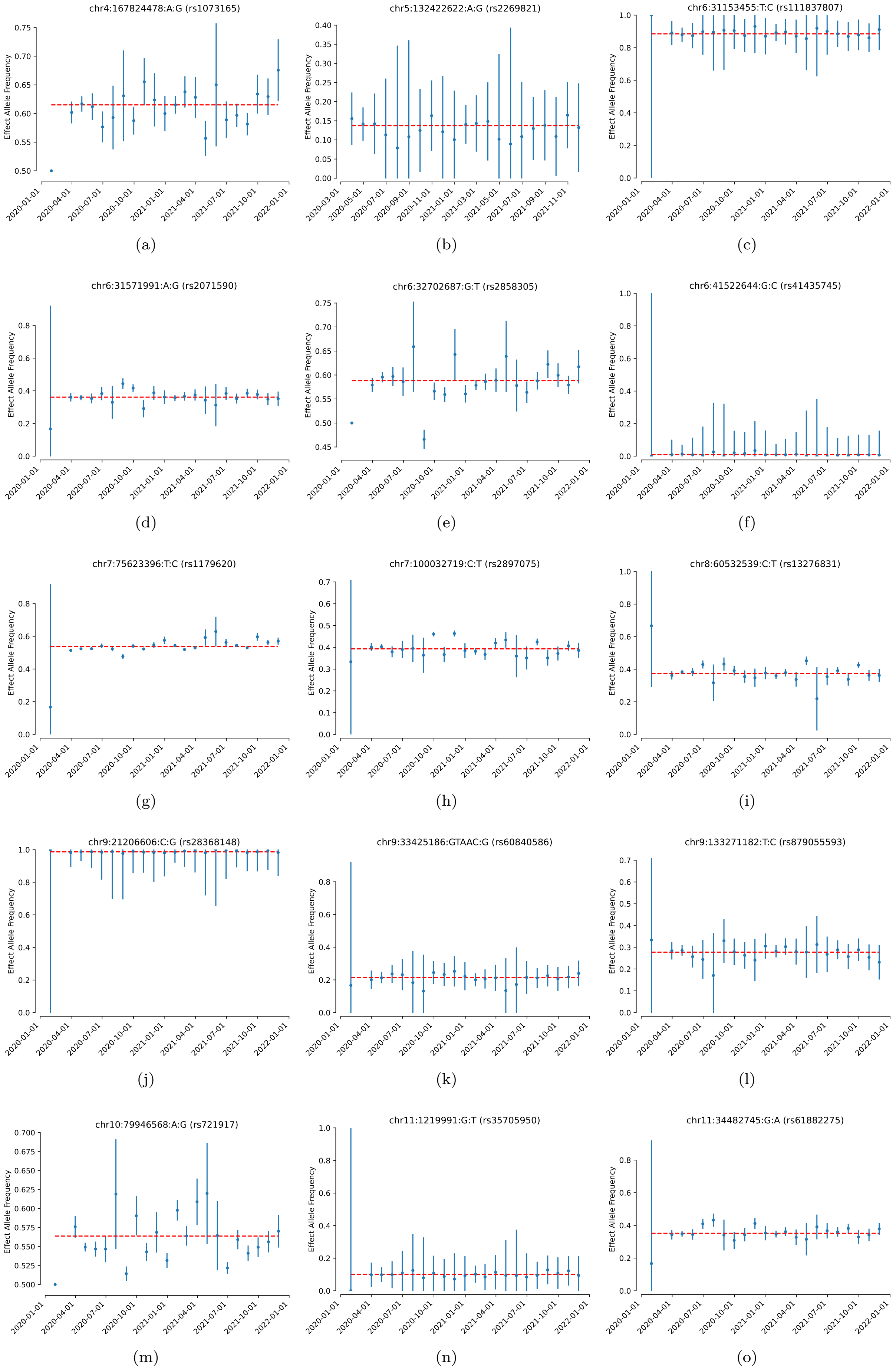

Supplementary Figure 46: Effect allele frequency (EAF +/- 95%CI) in critically-ill group (GenOMICC UK only) by month of for all lead variants. Dashed red line indicates EAF in the whole cohort. Sample sizes for each month are shown in panel (a) above.

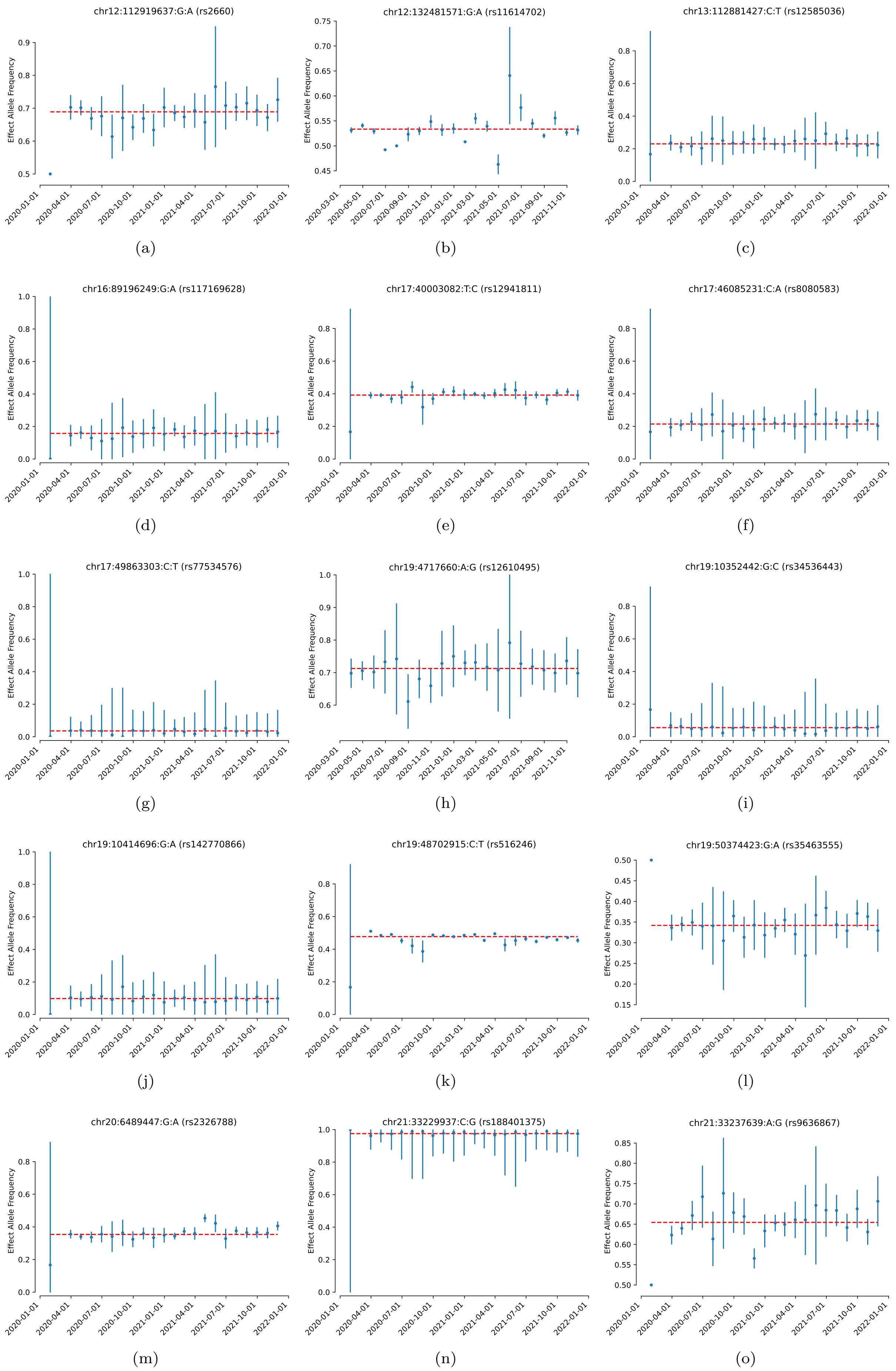

Supplementary Figure 47: Effect allele frequency (EAF +/- 95%CI) in critically-ill group (GenOMICC UK only) by month of for all lead variants. Dashed red line indicates EAF in the whole cohort. Sample sizes for each month are shown in panel (a) above.

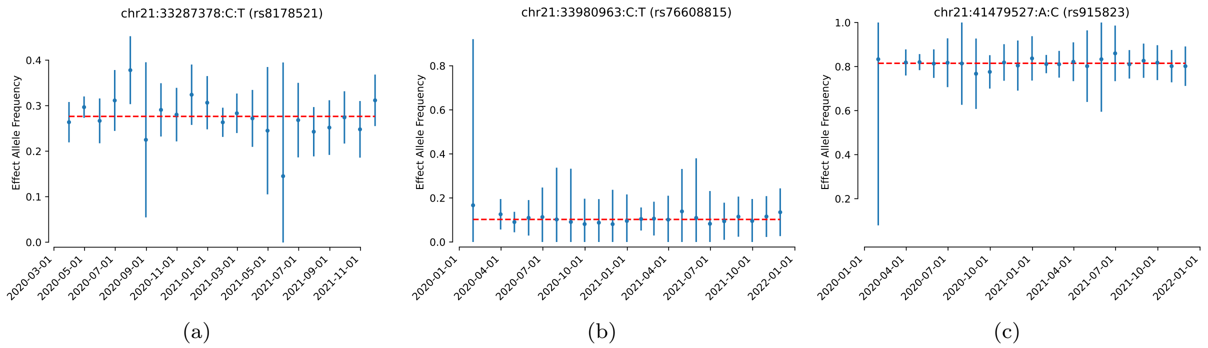

Supplementary Figure 48: Effect allele frequency (EAF +/- 95%CI) in critically-ill group (GenOMICC UK only) by month of for all lead variants. Dashed red line indicates EAF in the whole cohort. Sample sizes for each month are shown in panel (a) above.

12 Contributing Studies

12.1 Ethical approval

All participants, or their representatives where appropriate, gave informed consent. Details of ethical review for each study can be found below.

12.2 GenOMICC and ISARIC4C

Patients were recruited to the GenOMICC (Genetics Of Mortality In Critical Care) study in 224 intensive care units in the UK and Ireland (genomicc.org). All cases had confirmed Covid-19 according to local clinical testing and were deemed, in the view of the treating clinician, to require continuous cardiorespiratory monitoring. In UK practice this kind of monitoring is undertaken in high-dependency or intensive care units. Additional Covid-19 confirmed hospitalised cases were recruited through the International Severe Acute Respiratory Infection Consortium (ISARIC) Coronavirus Clinical Characterisation Consortium (4C).Both studies were approved by the appropriate research ethics committees (Scotland, 15/SS/0110; England, Wales and Northern Ireland, 19/WM/0247). Current and previous versions of the study protocol are available at genomicc.org/protocol. All participants gave informed consent.

Participants were recruited to the mild Covid-19 cohort on the basis of having experienced mild (non-hospitalised) or asymptomatic Covid-19. Participants volunteered to take part in the study via a microsite and were required to self-report the details of a positive Covid-19 test. Volunteers were prioritised for genome sequencing based on demographic matching with the critical Covid-19 cohort considering self-reported ancestry, sex, age and location.

12.2.1 Whole genome sequencing summary statistics

8794 critically ill individuals and 1809 mild Covid-19 controls were sequenced through the GenOMICC study. To increase control numbers, we added general population controls from 100k-genomes cohort. Participants were enrolled in the 100,000 Genomes Project from families with a broad range of rare diseases, cancers and infection by 13 regional NHS Genomic Medicine Centres across England and in Northern Ireland, Scotland and Wales. For this analysis, participants for whom a positive SARS-CoV-2 test had been recorded as of March, 2021 were not included due to uncertainty in the severity of Covid-19 symptoms. Only participants for whom genome sequencing was performed from blood derived DNA were included and participants with haematological malignancies were excluded to avoid potential tumour contamination. DNA extraction, sequencing, ancestry estimation, kinship estimation, principal components calculation and QC pipelines were performed as described in Kousathanas et al.[3]

GWAS was performed using a 2-step logistic mixed model regression approach as implemented in SAIGE v0.44.5 for single variant association analyses with sex, age, age squared , age by sex interaction and 20 principal components as covariates. The principal components were computed separately by predicted genetic ancestry (i.e, EUR-specific, AFR-specific, etc.), to capture subtle structure effects.

12.2.2 Genotype summary statistics

9484 Critically ill cases recruited through the GenOMICC, and 958 additional Covid-19 confirmed hospitalised cases recruited through ISARIC4C were genotyped. DNA extraction, sample QC, genotype QC, kinship estimation, ancestry estimation and imputation were performed with the pipelines described in Pairo-Castineira et al.[6].

After these steps and removing individuals with whole genome sequencing data available[3] there were 3577 unrelated individuals assigned to the European ancestry group, 383 unrelated individuals assigned to the South Asian ancestry group, 259 unrelated individuals assigned to the African ancestry group and 140 individuals assigned to East Asian ancestry group. Individuals from American ancestry were removed from the analysis because there were less than 100. For the critical illness analysis, individuals which did nof fulfill the critical illness criteria (ICU admission, or ventilation) were filtered out. Final numbers for critical illness GWAS are: 3213 individuals from European ancestry, 127 from East Asian ancestry, 353 from South Asian ancestry and 242 from African ancestry.

UK Biobank participants from a subset with no relationships up to third degree were considered as potential controls if they were not identified by the UK Biobank as outliers based on either genotyping missingness rate or heterogeneity, and their sex inferred from the genotypes matched their self-reported sex. After excluding participants who had received PCR tests for Covid-19, based on information downloaded from the UK Biobank on 27/01/2022. For each GenOMICC individual we considered the subset of UK Biobank individuals with matching assigned ancestry group sex, age and BMI distribution as

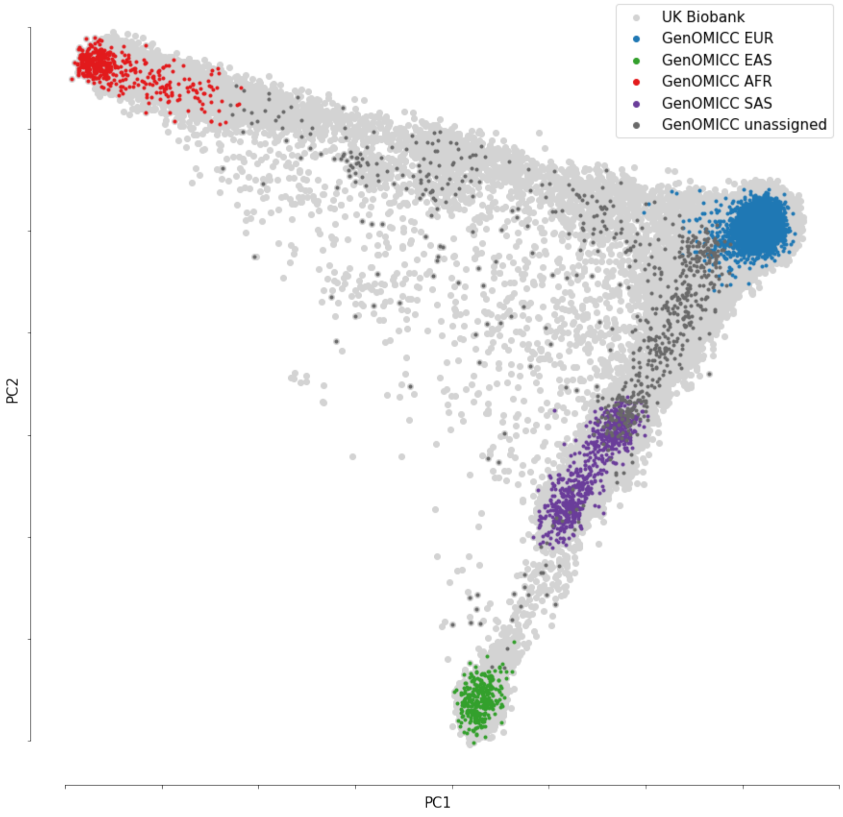

Supplementary Figure 49: Whole cohort PCA. Genomic PCA plot showing the first two principal components for the combination of all UK Biobank and GenOMICC participants. Assigned ancestry group for GenOMICC participants is indicated through colour.

controls. For non-European ancestries we selected the five closest controls, based on euclidean distance in the space of the first six ancestry specific genomic principal components.

Test for association between case–control status and allele dosage was performed by hospitalised and critical illness separately by fitting a logistic regression model using PLINK 2.00 with sex, age, mean-centred age^2, deprivation score decile of residential postcode, and the first 10 ancestry specific genomic principal components as covariates.

GWAS results for individuals from European ancestry were filtered for $MAF > 0.01$, HWE $p-value > 10^{-50}$, genotyping rate > 0.99 and imputation score > 0.9 in both GenOMICC and UK Biobank. To avoid bias for using a different genotyping array and imputation method between cases and controls, MAF for each SNP was compared between UK Biobank and gNOMAD non-Finnish European individuals, and SNPs were removed from following two rules (1)In SNPs with MAF > 0.1 in gnomAD, an absolute difference in MAF of 0.05 between gnomAD and UK Biobank controls; (2) in SNPs with MAF < 0.1 in gnomAD, a difference in MAF of $> 0.25 \times MAF_{gnomad}$ between UK Biobank controls and gnomAD. After this filter, a further LD-check filter, following Kousathanas et al[3] was used to clean the GWAS from false positives. GWAS analyses of individuals of East Asian, South Asian and African ancestries were filtered for variants filtered for a MAF> 0.05 in UK Biobank corresponding to the same ancestry and then for the SNPs that passed quality control in the European GWAS.

12.2.3 BraCovid/GenoMICC Brazil

Covid-19 patients were enrolled after hospitalisation in one of the following tertiary care centers in the metropolitan area of Sao Paulo, Brazil: Instituto do Coração, and Instituto Central do Hospital das Clínicas da Faculdade de Medicina da Universidade de São Paulo. The study was approved by the National Research Ethics Committee (CONEP) and Ethics Committee for the Analysis of Research Projects at HC FMUSP (CAPPesq, 5025/20/054). Non-hospitalised cases were selected by serological studies surveys for previous SARS-CoV-2 infection or SARS-CoV-2 PCR test among health professionals or the general population.After signing an informed consent, a sample of whole-blood already collected for in-hospital biochemical analysis or SARS-CoV-2 serology was used for genomic DNA extraction.DNA extraction, genotyping, imputation and QC pipelines are described in Pereira et al.[1]. Principal components for

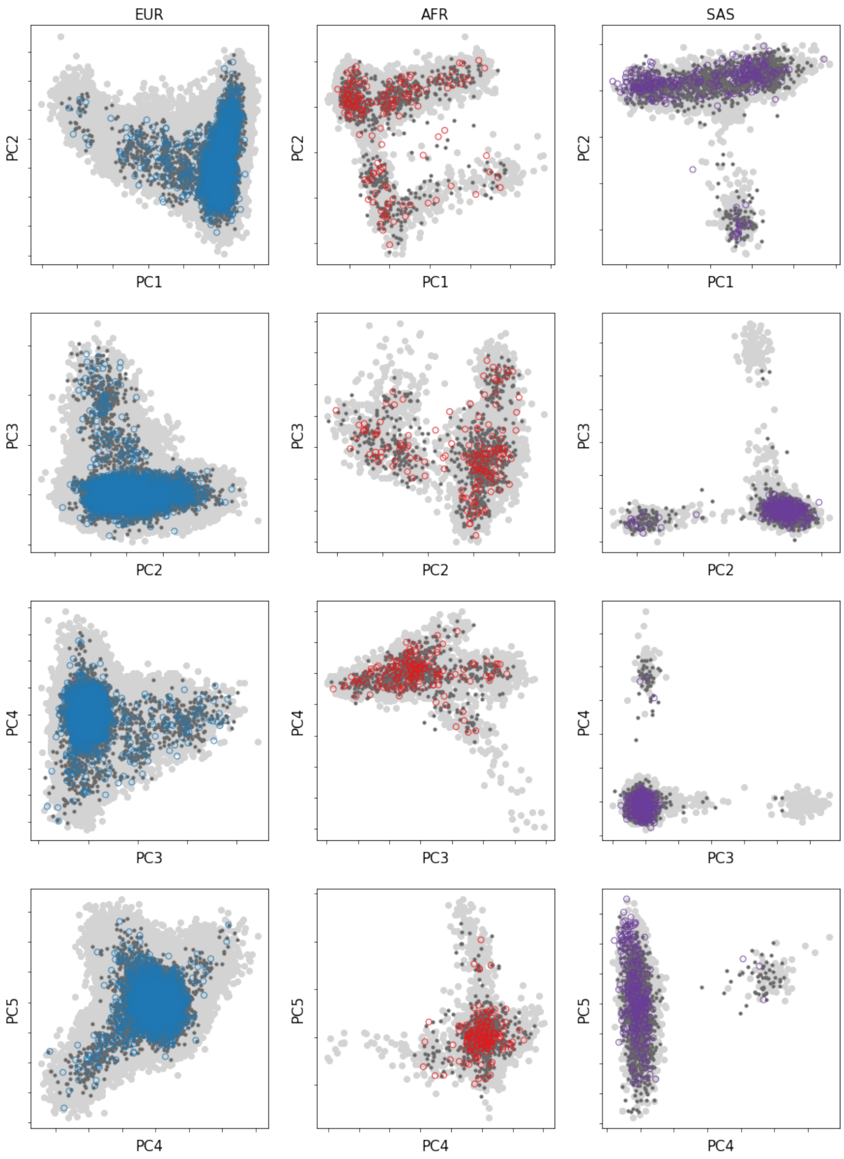

Supplementary Figure 50: Ancestry-specific PCA. Genomic PCA plots showing the distribution of all cases and controls for the first 5 principal components for each ancestry group. Cases are shown as coloured open circles: European (EUR, blue), African (AFR, red), and South Asian (SAS, purple). Controls are dark grey closed circles. UK Biobank population background is shown as light grey closed circles.

cases and controls were calculated with PLINK 2.0. Due to the level of admixture and complex genetic structure present in the Brazilian population, three different ancestry subgroups were defined (European, African, Native American) using k-means clustering. GWAS of Covid-19 hospitalised cases versus Covid-19 positive mild controls was performed with a logistic regression model in PLINK 2.0 including age, sex, array type and four principal components as covariates for each ancestry. A trans-ancestry fixed-effect meta-analysis was calculated using the plink meta-analysis routine[1].

12.2.4 GenOMICC Saudi Arabia

In Saudi Arabia 1036 participants were recruited as part of the GenOMICC Saudi Arabia study. Informed Consent was provided to each participant or their legal guardian (if the participant could not consent) by the corresponding institute. This study was approved the IRB of each participating hospitals, and the IRB at King Abdullah International Medical Research Centre, Ministry of National Guard–Health Affairs, Riyadh, Ministry of Health, and King Fahad Medical City. Participants were diagnosed of Covid-19 through a positive PCR test, and were genotyped using the Affymetrix Saudi Arabia platform. After a manual QC with Axiom suite, sample QC, genotype QC and kinship estimation were performed as in Pairo-Castineira et al.[6]. To infer ancestry, a first step was done projecting the genotype data into 1000 Genomes principal components calculated with GCTA 1.9. Most individuals had admix ancestry according to PCA projection (Supplementary Figure 51), which is consistent with population structure analysis of the region[10]. Outliers belonging to East Asian, South Asian or African ancestries were removed from the analysis because there were not enough numbers to perform an ancestry-specific GWAS. Then we performed a principal component analysis of the remaining individuals using GCTA 1.9(Supplementary Figure 52). Individuals with a deviation more than 2sd from the mean in the 10 first principal components were considered outliers and removed from the dataset. After QC 93 severe cases and 645 controls were used for the GWAS. Analysis was performed as in Pairo-Castineira et al.[6] using PLINK2 and unrelated individuals but in order to account for the admix ancestry of the participants, we included into the model age, sex and first 10 pcs as covariates and an interaction term between the genotype and the first principal component.

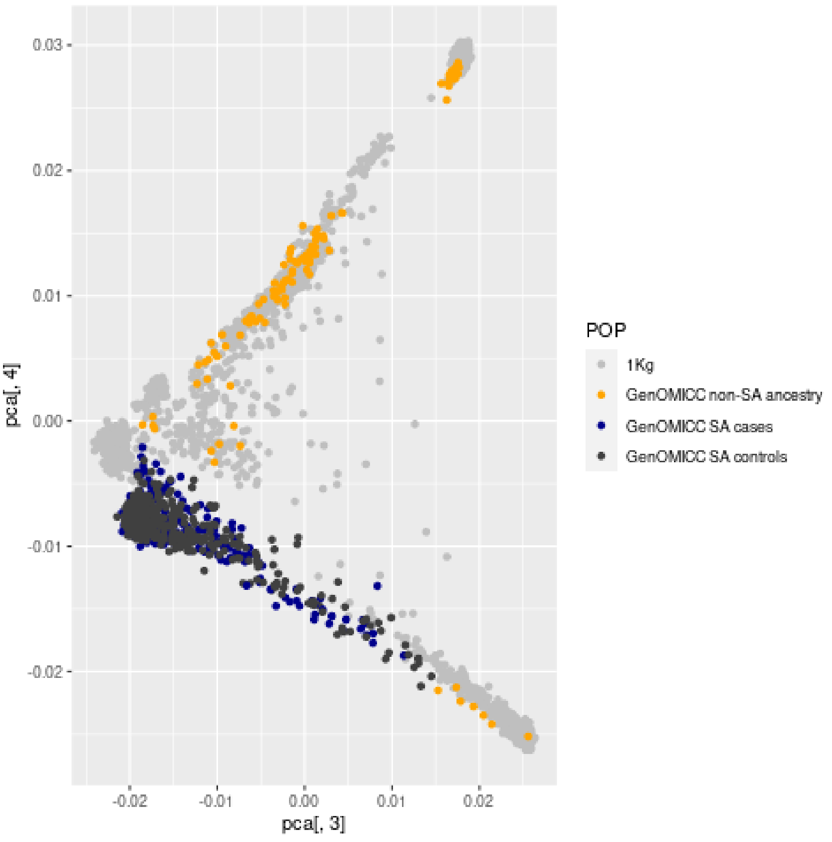

Supplementary Figure 51: Genomic PCA plot showing the first two principal components for the combination of 1000 Genomes project and GenOMICC Saudi Arabia participants. GenOMICC Saudi Arabia cases are coloured in blue, GenOMICC Saudi Arabia controls in dark grey, and GenOMICC Saudi Arabia participants removed for the analysis due to ancestry in orange

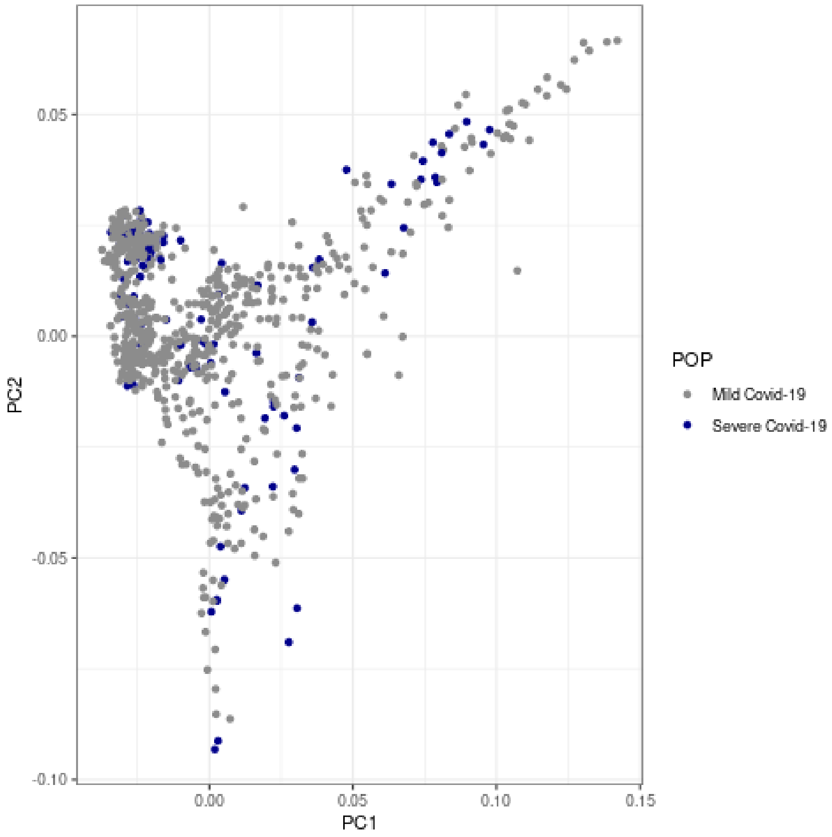

Supplementary Figure 52: Ancestry-specific PCA plot for Saudi Arabian participants. Severe cases are coloured in blue and mild cases used as controls in grey

12.3 SCOURGE

In Spain, 11,939 Covid-19 positive cases were recruited as part of SCOURGE study from 34 centres in 25 cities.[2] Study samples and data were collected by the participating centers, through their respective biobanks after informed consent, with the approval of the respective Ethic and Scientific Committees. The whole project was approved by the Galician Ethical Committee Ref 2020/197. Individuals were diagnosed as Covid-19 positive through a PCR-based test or according to local clinical and laboratory procedures. All cases were classified in a five-level severity scale. Two Spanish sample collections with unknown Covid-19 status were included as general population controls in some analyses: 3,437 samples from the Spanish DNA biobank (https://www.bancoadn.org) and 2,506 samples from the GR@CE consortium. DNA extraction, genotyping, imputation, ancestry estimation, kinship estimation and QC pipelines are described in Cruz et al.[2].

GWAS was performed by fitting a logisitic regression models implemented in SAIGE. For the hospitalised meta-analysis the summary statistics of the hospitalisation vs non-hospitalised (mild cases and population controls) analysis were used. For the critical illness meta-analysis, the summary statistics of severity grade 3 and 4 as defined by SCOURGE[2] vs population controls were used.

12.4 HGI release 6

In total 25 studies contributed to the A2 analysis, with 8779 cases and 1001572 controls and 43 studies contributed to the B2 analysis with 24274 cases and 2061529 controls without including 23andMe data. All studies followed protocols approved by local Institutional Review Boards; All protocols followed local ethics recommendations and informed consent was obtained when required. Meta-analysis of all cohorts was performed with and inverse-weighting variance method as described by the Covid-19 Host Genetics Initiative[11]

In order to account for signal due to sample overlap we performed a mathematical subtraction from HGIv6B2 of the GenOMICC GWAS of European genetic ancestry and the BraCovid analysis, and a mathematical subtraction from HGIv6A2 of the GenOMICC GWAS of European genetic ancestry . Publicly-available HGI data was downloaded from https://www.covid19hg.org/results/r6/. The subtraction was performed using MetaSubtract package (version 1.60) for R (version 4.0.2) after removing

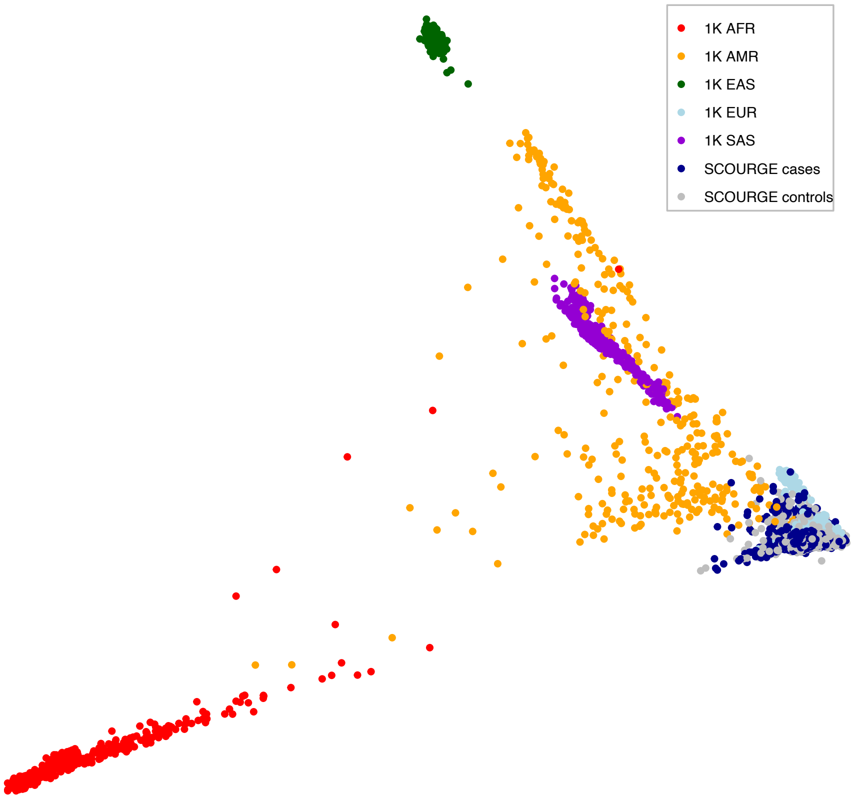

Supplementary Figure 53: Genomic PCA plot showing the first two principal components for the combination of 1000 Genomes project and SCOURGE cases and controls. 1000 Genomes individuals are coloured according to ancestry, SCOURGE cases in blue and controls in grey

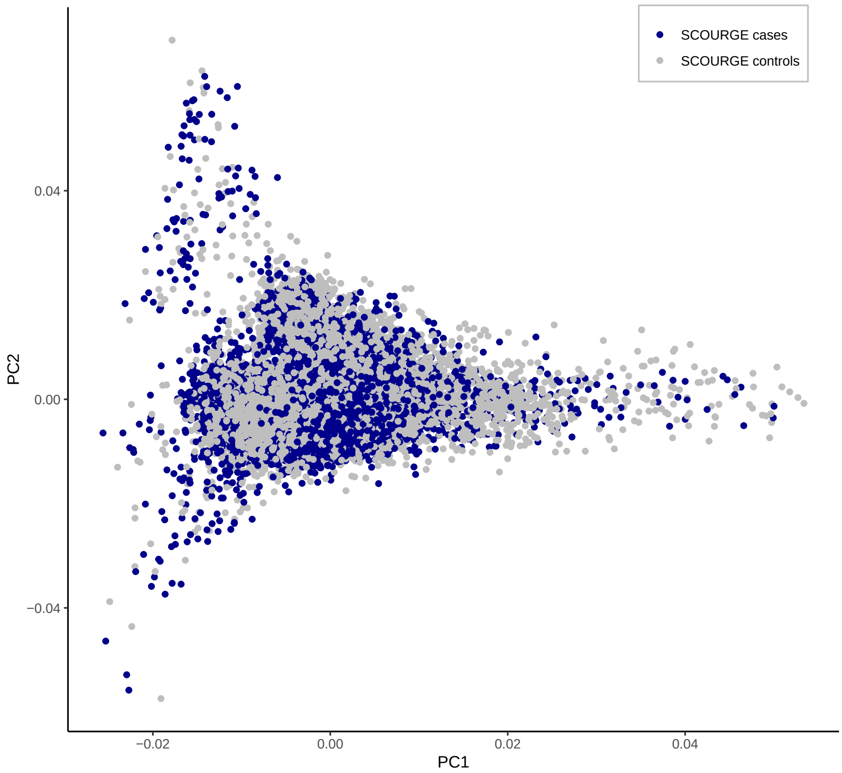

Supplementary Figure 54: Ancestry Specific PCA plot showing the first two principal components for the SCOURGE study. Controls are coloured in grey and cases in blue

variants with the same genomic position and using the lambda.cohorts with genomic inflation calculated on the GenOMICC summary statistics.

12.5 23andMe

Participants were recruited from the customer base of 23andMe, Inc., a personal genetics company. 23andMe, Inc. participants provided informed consent and volunteered to participate in the research online, under a protocol approved by the external AAHRPP-accredited IRB, Ethical & Independent (E&I) Review Services. As of 2022, E&I Review Services is part of Salus IRB (https://www.versiticlinicaltrials.org/salusirb).

Study participation consisted solely of web-based surveys, and different phenotypes were defined for disease severity: pneumonia, hospitalisation and respiratory support. DNA extraction, genotyping, imputation, ancestry estimation, kinship estimation and QC pipelines are described in Shelton et al.[12].

GWAS were performed for each phenotype and ancestry separately using a logistic regression using age, sex, age squared, a age:sex interaction and top ten principal components as covariates, as described in Shelton et al.[12]

For the hospitalisation meta-analysis we used summary statistics from the respiratory support or pneumonia phenotype against population controls GWAS from European, African and Latino ancestries. For the critically-ill meta-analysis we used summary statistics from the respiratory phenotype against population controls GWAS. We filtered variants that passed internal 23andMe ancestry QC[12] and had imputation score > 0.6 and with MAF > 0.005.

13 Contributors

13.1 GenOMICC Investigators

13.1.1 Co-Investigators

J. Kenneth Baillie[1,2,3,11], Colin Begg[27], Sara Clohisey[3], Charles Hinds[20], Peter Horby[21], Julian Knight[8], Lowell Ling[22], David Maslove[14], Danny McAuley[23,24], Johnny Millar[3], Hugh Montgomery[25], Alistair Nichol[15], Peter J.M. Openshaw[12,26], Alexandre C Pereira[48], Chris P Ponting[2], Kathy Rowan[47], Malcolm G Semple[16,17], Manu Shankar-Hari[18], Charlotte Summers[19], Timothy Walsh[11].

13.1.2 Management, Laboratory and Data team

Emma Aitkin[3], Latha Aravindan[49], Ruth Armstrong[3], J. Kenneth Baillie[3,11], Heather Biggs[50], Ceilia Boz[3], Adam Brown[3], Primmy Chikowore[3], Richard Clark[13], Sara Clohisey[3], Audrey Coutts[13], Judy Coyle[3], Louise Cullum[3], Sukamal Das[49], Nicky Day[3], Lorna Donnelly[13], Esther Duncan[3], Angie Fawkes[13], Paul Finernan[3], Max Head Fourman[3], Anita Furlong[50], James Furniss[3], Bernadette Gallagher[3], Tammy Gilchrist[13], Ailsa Golightly[3], Fiona Griffiths[3], Katarzyna Hafezi[13], Debbie Hamilton[3], Ross Hendry[3], Naomi Kearns[3], Andy Law[3], Dawn Law[3], Rachel Law[3], Sarah Law[3], Rebecca Lidstone-Scott[3], Christen Lauder[13], Louise Macgillivray[13], Alan Maclean[13], Hanning Mal[3], Sarah McCafferty[13], Ellie Mcmaster[3], Jen Meikle[3], Shona C Moore[16], Kirstie Morrice[13], Lee Murphy[13], Sheena Murphy[49], Hellen Mybaya[3], Miranda Odam[3], Wilna Oosthuyzen[3], Chenqing Zheng[51], Jiantao Chen[51], Nick Parkinson[3], Trevor Paterson[3], Petra Tucker[50], Katherine Schon[50], Andrew Stenhouse[3], Mihaela Das[49], Maaike Swets[3,52], Helen Szoor-McElhinney[3], Filip Taneski[3], Lance Turtle[16], Tony Wackett[3], Mairi Ward[3], Jane Weaver[3], Nicola Wrobel[13], Marie Zechner[3].

13.1.3 Guys and St Thomas' Hospital, London, UK

Jacqueline Pan[53], Neus Grau[53], Tim Owen Jones[53], Rosario Lim[53], Martina Marotti[53], Christopher Whitton[53], Aneta Bociek[53], Sara Campos[53], Gill Arbane[53], Manu Shankar-Hari[53], Marlies Ostermann[53], Mina Cha[53], Fabiola DAmato[53], Eirini Kosifidou[53], Shelley Lorah[53], Kyma Morera[53].

13.1.4 James Cook University Hospital, Middlesbrough, UK

Laura Brady[54], Keith Hugill[54], Jeremy Henning[54], Stephen Bonner[54], Evie Headlam[54], Jessica Jones[54], Abigail List[54], Joanne Morley[54], Amy Welford[54], Bobette Kamangu[54], Anitha Ratnakumar[54], Abiola Shoremekun[54].

13.1.5 Barts Health NHS Trust, London, UK

Zoe Alldis[55], Raine Astin-Chamberlain[55], Fatima Bibi[55], Jack Biddle[55], Sarah Blow[55], Matthew Bolton[55], Catherine Borra[55], Ruth Bowles[55], Maudrian Burton[55], Yasmin Choudhury[55], Amber Cox[55], Amy Easthope[55], Patrizia Ebano[55], Stavros Fotiadis[55], Jana Gurasashvili[55], Rosslyn Halls[55], Pippa Hartridge[55], Delordson Kallon[55], Jamila Kassam[55], Ivone Lancoma-Malcolm[55], Maninderpal Matharu[55], Peter May[55], Oliver Mitchelmore[55], Tabitha Newman[55], Mital Patel[55], Jane Pheby[55], Irene Pinzuti[55], Zoe Prime[55], Oleksandra Prysyazhna[55], Julian Shiel[55], Melanie Taylor[55], Carey Tierney[55], Olivier Zongo[55], Suzanne Wood[55], Anne Zak[55], David Collier[55].

13.1.6 Royal Stoke University Hospital, Staffordshire, UK

Manuela Mundy[56], Christopher Thompson[56], Lisa Pritchard[56], Minnie Gellamucho[56], David Cartlidge[56], Nageswar Bandla[56], Lucy Bailey[56], Michelle Davies[56], Jane Delaney[56], Leanne Scott[56].

13.1.7 North Middlesex University Hospital NHS trust, London, UK

Marwa Abdelrazik[57], Frater Alasdair[57], David Carter[57], Munzir Elhassan[57], Arunkumar Ganesan[57], Samuel Jenkins[57], Zoe Lamond[57], Dharam Purohit[57], Kumar Rohit[57], Malik Saleem[57], Alanna Wall[57], Kugan Xavier[57], Dhanalaksmi Bakthavatsalam[57], Kirolos Gehad[57], Pakeerathan Gnanapragasam[57], Kapil Jain[57], Swati Jain[57], Abdul Malik[57], Naveen Pappachan[57], Jeronimo Moreno-Cuesta[57], Anne Haldeos[57], Rachel Vincent[57], Maryjane Oziegb[57].

13.1.8 King's College Hospital, London, UK

Anna Cavazza[58], Maeve Cockrell[58], Eleanor Corcoran[58], Maria Depante[58], Clare Finney[58], Ellen Jerome[58], Abigail Knighton[58], Monalisa Nayak[58], Evita Pappa[58], Rohit Saha[58], Sian Saha[58], Andrew Dodd[58], Kevin O'Reilly[58], Mark McPhail[58], Emma Clarey[58], Harriet Noble[58], John Smith[58].

13.1.9 Charing Cross Hospital, St Mary's Hospital and Hammersmith Hospital, London, UK

Phoebe Coghlan[59], Stephen Brett[59], Anthony Gordon[59], Maie Templeton[59], David Antcliffe[59], Dorota Banach[59], Sarah Darnell[59], Ziortza Fernandez[59], Eleanor Jepson[59], Amal Mohammed[59], Roceld Rojo[59], Sonia Sousa Arias[59], Anita Tamang Gurung[59], Jenny Wong[59].

13.1.10 The Royal Liverpool University Hospital, Liverpool, UK

Jaime Fernandez-Roman[60], David O. Hamilton[60], Emily Johnson[60], Brian Johnston[60], Maria Lopez Martinez[60], Suleman Mulla[60], Alicia A.C. Waite[60], Karen Williams[60], Victoria Waugh[60], Ingeborg Welters[60], Jessica Emblem[60], Maria Norris[60], David Shaw[60].

13.1.11 John Radcliffe Hospital, Oxford, UK

Archana Bashyal[61], Sally Beer[61], Paula Hutton[61], Stuart McKechnie[61], Neil Davidson[61], Soya Mathew[61], Grace Readion[61], Jung Ryu[61], Jean Wilson[61].

13.1.12 Addenbrooke's Hospital, Cambridge, UK

Shruti Agrawal[62], Kay Elston[62], Megan Jones[62], Eoghan Meaney[62], Petra Polgarova[62], Muhammad Elbehery[62], Charlotte Summers[62], Esther Daubney[62], Anthony Ng[62], Jocelyn Marshall[62], Nazima Pathan[62], Katerina Stroud[62], Deborah White[62].

13.1.13 Nottingham University Hospital, Nottingham, UK

Angela Andrew[63], Saima Ashraf[63], Amy Clark[63], Martin Dent[63], Margaret Langley[63], Cecilia Peters[63], Lucy Ryan[63], Julia Sampson[63], Shuying Wei[63], Alice Baddeley[63], Megan Meredith[63], Lucy Morris[63], Alexandra Gibbons[63], Lisa McLoughlin[63].

13.1.14 St George's Hospital, London, UK

Carlos Castro Delgado[64], Victoria Clark[64], Deborah Dawson[64], Lijun Ding[64], Georgia Durrant[64], Obiageri Ezeobu[64], Abiola Harrison[64], William James Hurt[64], Rebecca Kanu[64], Ashley Kinch[64], Susannah Leaver[64], Ana Lisboa[64], Jisha Mathew[64], Kamal Patel[64], Romina Pepermans Saluzzio[64], John Rawlins[64], Tinashe Samakomva[64], Nirav Shah[64], Christine Sicat[64], Joana Texeira[64], Joana Gomes De Queiroz[64],

Edna Fernandes Da Gloria[64], Elena Maccacari[64], Nikki Yun[64], Soumendu Manna[64], Sarah Farnell-Ward[64], Maria Maizcordoba[64], Maria Thanasi[64], Hawakin Haji Ali[64].

13.1.15 BHRUT (Barking Havering) - Queens Hospital and King George Hospital, Essex, UK

Janice Hastings[65], Lina Grauslyte[65], Musarat Hussain[65], Bobby Ruge[65], Sam King[65], Tatiana Pogreban[65], Lace Rosaroso[65], Helen Smith[65], Mandeep-Kaur Phull[65], Nikkita Adams[65], George Franke[65], Aparna George[65], Erika Salciute[65], Joanna Wong[65], Karen Dunne[65], Luke Flower[65], Emma Sharland[65], Sukhmani Sra[65].

13.1.16 Royal Infirmary of Edinburgh, Edinburgh, UK

Gillian Andrew[66], Marie Callaghan[66], Lucy Barclay[66], Lucy Marshall[66], Kenneth Baillie[66], Maria Amamio[66], Sophie Birch[66], Kate Briton[66], Sarah Clark[66], Katerine Doverman[66], Dave Hope[66], Corrienne Mcculloch[66], Scott Simpson[66], Jo Singleton[66].

13.1.17 Kingston Hospital, Surrey, UK

Rita Fernandez[67], Meryem Allen[67], David Baptista[67], Rebecca Crowe[67], Jonathan Fox[67], Jacyntha Khera[67], Adam Loveridge[67], India McKenley[67], Eriko Morino[67], Andres Naranjo[67], Denise O'Connor[67], Richard Simms[67], Kathryn Sollesta[67], Andrew Swain[67], Harish Venkatesh[67], Rosie Herdman-Grant[67], Anna Joseph[67].

13.1.18 Queen Alexandra Hospital, Portsmouth, UK

Angela Nown[68], Steve Rose[68], David Pogson[68], Helen Boxall[68], Lutece Brimfield[68], Helen Claridge[68], Zoe Daly[68], Shenu George[68], Andrew Gribbin[68].

13.1.19 Royal Gwent Hospital, Newport, UK

Yusuf Cheema[69], Sean Cutler[69], Owen Richards[69], Anna Roynon-Reed[69], Shiney Cherian[69], Anne Emma Heron[69], Gemma Williams[69], Tamas Szakmany[69], Abby Waters[69], Kim Collins[69], Jill Dunhill[69], Ffion Jones[69], Rebecca Morris[69], Lucy Ship[69], Amy Cardwell[69].

13.1.20 Royal Blackburn Teaching Hospital, Blackburn, UK

Syamlan Ali[70], Ravi Bhatterjee[70], Rachel Bolton[70], Srikanth Chukkambotla[70], Dabheoc Coleman[70], Jack Dalziel[70], Joseph Dykes[70], Christopher Fine[70], Bethan Gay[70], Wendy Goddard[70], Drew Goodchild[70], Rhiannan Harling[70], Muhammad Hijazi[70], Sarah Keith[70], Meherunnisa Khan[70], Roseanna Matt[70], Janet Ryan-Smith[70], Samuel Saad[70], Philippa Springle[70], Jacqueline Thomas[70], Nick Truman[70], Aayesha Kazi[70], Matthew Smith[70], Heather Collier[70], Chloe Davison[70], Stephen Duberley[70], Jeanette Hargreaves[70], Janice Hartley[70], Tahera Patel[70], Ellen Smith[70].

13.1.21 Stepping Hill Hospital, Stockport, UK

Alissa Kent[71], Emma Goodwin[71], Ahmed Zaki[71], Clare Tibke[71], Susan Hopkins[71], Hywel Gerrard[71], Matthew Jackson[71], Sara Bennett[71], Liane Marsh[71], Rebecca Mills[71].

13.1.22 Northumbria Healthcare NHS Foundation Trust, North Shields, UK

Jessica Bell[72], Helen Campbell[72], Angela Dawson[72], Steve Dodds[72], Stacey Duffy[72], Lisa Gallagher[72], Gemma McCafferty[72], Stacey Short[72], Tracy Smith[72], Kirsty Thomas[72], Claire Walker[72], Jessica Reynolds[72], Bryan Yates[72], Hayley McKie[72], Maria Panteli[72], Maria Thompson[72], Gail Waddell[72].

13.1.23 Countess of Chester Hospital, Chester, UK

Sarah De Beger[73], Azmerelda Abraheem[73], Charlie Dunmore[73], Rumanah Girach[73], Rhianna Jones[73], Emily London[73], Imrun Nagra[73], Farah Nasir[73], Hannah Sainsbury[73], Clare Smedley[73], Stephen Brearey[73], Caroline Burchett[73], Kathryn Cawley[73], Maria Faulkner[73], Helen Jeffrey[73], Peter Bamford[73], Firdaus Shaikh[73], Lauren Slack[73], Angela Davies[73].

13.1.24 Pinderfields General Hospital, Wakefield, UK

Hollie Brooke[74], Jose Cebrian Suarez[74], Ruth Charlesworth[74], Karen Hansson[74], John Norris[74], Alice Poole[74], Rajdeep Sandhu[74], Elizabeth Smithson[74], Muthu Thirumaran[74], Veronica Wagstaff[74], Sarah Buckley[74], Brendan Sloan[74], Alastair Rose[74], Amy Major[74], Alexandra Metcalfe[74].

13.1.25 Ninewells Hospital, Dundee, UK

Christine Almaden-Boyle[75], Pauline Austin[75], Susan Chapman[75], Alexandre Eros[75], Louise Cabrelli[75], Stephen Cole[75], Clare Whyte[75], Matt Casey[75].

13.1.26 Croydon University Hospital, Croydon, UK

Vasileios Bafitis[76], George Tsinaslanidis[76], Cassandra George[76], Reena Khade[76], Christopher Black[76], Sundar Raj Ashok[76].

13.1.27 Morriston Hospital, Swansea, UK

Sean Farley[77], Elaine Brinkworth[77], Rachel Harford[77], Carl Murphy[77], Marie Williams[77], Luke Newey[77], Hannah Toghill[77], Sophie Lewis[77], Tabitha Rees[77], Ceri Battle[77], Mark Baker[77], Jenny Travers[77], Karen Chesters[77].

13.1.28 Queen Elizabeth University Hospital, Glasgow, UK

Nicola Baxter[78], Andrew Arnott[78], Gordan McCreath[78], Christopher McParland[78], Laura Rooney[78], Malcolm Sim[78], Steven Henderson[78], Lynn Abel[78], Carol Dalton[78], Sophie Kennedy-Hay[78], Lynn O'Donohoe[78], Megan O'Hare[78], Izabela Orlikowska[78], Natasha Parker[78].

13.1.29 Broomfield Hospital, Chelmsford, UK

Fiona McNeela[79], Amanda Lyle[79], Alistair Hughes[79], Jayachandran Radhakrishnan[79], Sian Gibson[79].

13.1.30 Heartlands Hospital, Birmingham, UK

Hollie Bancroft[80], Mary Bellamy[80], Jacqueline Daglish[80], Salma Kadiri[80], Faye Moore[80], Joanne Rhodes[80], Mirriam Sangombe[80], Zhane Peterkin[80], James Scriven[80], Margaret Carmody[80].

13.1.31 Royal Sussex County Hospital, Brighton, UK

Juliet Cottle[81], Emily Peasgood[81], Laura Ortiz-Ruiz de Gordoa[81], Claire Phillips[81], Denise Skinner[81].

13.1.32 York Hospital, York, UK

Zoe Cinquina[82], Kate Howard[82], Rosie Joy[82], Samantha Roche[82], Isobel Birkinshaw[82], Joseph Carter[82], Jo Ingham[82], Nicola Marshall[82], Harriet Pearson[82], Zoe Scott[82].

13.1.33 Queen Elizabeth Hospital, Birmingham, UK

Jo Dasgin[83], Jaspret Gill[83], Annette Nilsson[83], Amy Bamford[83], Diana Hull[83], James Scriven[83], Nafeesah Ahmadhaider[83], Michelle Bates[83], Christopher McGhee[83].

13.1.34 Royal Glamorgan Hospital, Pontyclun, UK

Hannah Ellis[84], Gwenllian Sera Howe[84], Jayaprakash Singh[84], Natalie Stroud[84], Lisa Roche[84], Ceri Lynch[84], Bethan Deacon[84], Carla Pothecary[84], Justyna Smeaton[84], Kevin Agravante[84].

13.1.35 Barnet Hospital, London, UK

Vinodh Krishnamurthy[85], Cynthia Diaba[85], Lincy John[85], Lai Lim[85], Rajeev Jha[85].

13.1.36 Wythenshawe Hospital, Manchester, UK

Jasmine Egan[86], Timothy Felton[86], Susannah Glasgow[86], Grace Padden[86], Ozerah Choudhr[86], Joanne Bradley-Potts[86], Stuart Moss[86], Saejohn Lingeswaran[86], Peter Alexander[86], Craig Brandwood[86], Sofia Fiouni[86], Luke Ward[86], Schvearn Allen[86], Jane Shaw[86], Christopher Smith[86].

13.1.37 Medway Maritime Hospital, Gillingham, UK

Oluronke Adanini[87], Rebecca Collins[87], Maines Msiska[87], Linda Ofori[87], Nikhil Bhatia[87], Hayley Dolan[87].

13.1.38 Royal Berkshire NHS Foundation Trust, Berkshire, UK

Mark Brunton[88], Jess Caterson[88], Holly Coles[88], Liza Keating[88], Emma Tilney[88], Nicola Jacques[88], Matthew Frise[88], Jennifer Armistead[88], Shauna Bartley[88], Parminder Bhuie[88], Sabi Rai[88], Gabriela Tomkova[88].

13.1.39 Whiston Hospital, Prescot, UK

Sandra Greer[89], Karen Shuker[89], Ascanio Tridente[89].

13.1.40 The Royal Oldham Hospital, Manchester, UK

Emma Dobson[90], Jodie Hunt[90], Redmond Tully[90], Joy Dearden[90], Andrew Drummond[90], Prakash Kamath[90], Emily Bullock[90], Michelle Mulcahy[90], Shelia Munt[90], Grainne O'Connor[90], Jennifer Philbin[90], Chloe Rishton[90], Chloe Scott[90], Sarah Winnard[90].

13.1.41 Chesterfield Royal Hospital Foundation Trust, Chesterfield, UK

Nurkamalia Hasni[91], Rachel Gascoyne[91], Joanne Hawes[91], Kelly Pritchard[91], Lesley Stevenson[91], Amanda Whileman[91], Sarah Beavis[91], Lauren Bishop[91], Cindy Cart[91], Katie Dale[91], Mary Kelly-Baxter[91], Adam Mendelski[91], Emma Moakes[91], Rheanna Smith[91], Jan Woodward[91], Stephanie Wright[91].

13.1.42 Aberdeen Royal Infirmary, Aberdeen, UK

Angela Allan[92], Adriana Botello[92], Jade Liew[92], Jasmine Medhora[92], Erin Trumper[92], Felicity Savage[92], Teresa Scott[92], Marc Place[92], Callum Kaye[92].

13.1.43 Royal Devon and Exeter Hospital, Exeter, UK

Sarah Benyon[93], Suzie Marriott[93], Linda Park[93], Helen Quinn[93], Daisy Skyes[93], Lily Zitter[93], Kizzy Baines[93], Elizabeth Gordon[93], Samantha Keenan[93], Andrew Pitt[93].

13.1.44 Glasgow Royal Infirmary, Glasgow, UK

Katharine Duffy[94], Jane Ireland[94], Gary Semple[94], Lynne Turner[94], Susanne Cathcart[94], Dominic Rimmer[94], Alex Puxty[94], Kathryn Puxty[94], Andrew Hurst[94], Jennifer Miller[94], Susan Speirs[94], Lauren Walker[94].

13.1.45 Blackpool Victoria Hospital, Blackpool, UK

Zena Bradshaw[95], Joanna Brown[95], Sarah Melling[95], Stephen Preston[95], Nicola Slawson[95], Scott Warden[95], Alanna Beasley[95], Emma Stoddard[95], Leonie Benham[95], Jason Cupitt[95], Melanie Caswell[95], Lisa Elawamy[95], Ashleigh Wignall[95].

13.1.46 Southampton General Hospital, Southampton, UK

Belinda Roberts[96], Hannah Golding[96], Samantha Leggett[96], Michelle Male[96], Martyna Marani[96], Kirsty Prager[96], Toran Williams[96], Kim Golder[96], Oliver Jones[96], Rebecca Cusack[96], Clare Bolger[96], Rachel Burnish[96], Michael Carter[96], Susan Jackson[96], Karen Salmon[96], Jonathan Biss[96].

13.1.47 Ashford and St Peter's Hospital, Surrey, UK

Maia Aquino[97], Maria Croft[97], Victoria Frost[97], Ian White[97], Keshnie Govender[97].

13.1.48 Derriford Hospital, Plymouth, UK

Natasha Webb[98], Liana Stapleton[98], Colin Wells[98], Nikitas Nikitas[98], Ana Sanchez - Rodriguez[98], Kayleigh Spencer[98], Bethan Stowe[98].

13.1.49 East Surrey Hospital, Redhill, UK

Yvonne Izzard[99], Michelle Poole[99], Sonja Monnery[99], Sallyanne Trotman[99], Valerie Beech[99], Edward Combes[99], Teishel Joefield[99].

13.1.50 Poole Hospital, Poole, UK

Patrick Covernton[100], Sarah Savage[100], Elizabeth Woodward[100], Julie Camsooksai[100], Henrik Reschreiter[100], Charlotte Barclay[100], Yasmin DeAth[100], Judith Dube[100], Charlotte Humphrey[100], Sarah Jenkins[100], Emma Langridge[100], Rebecca Milne[100], Beverley Wadams[100], Megan Woolcock[100].

13.1.51 Royal Alexandra Hospital, Paisley, UK

Michael Brett[101], Brian Digby[101], Lisa Gemmell[101], James Hornsby[101], Patrick MacGoey[101], Pauline O'Neil[101], Richard Price[101], Radha Sundaram[101], Lynn Abel[101], Natalie Rodden[101], Nicola Thomson[101], Kevin Rooney[101], Susan Currie[101], Natasha Parker[101], Lauren Walker[101], Philip Henderson[101].

13.1.52 St James's University Hospital and Leeds General Infirmary, Leeds, UK

Bethan Ogg[102], Simon Whiteley[102], Liz Wilby[102], Kate Long[102], Shailamma Matthew[102], Sheila Salada[102], Susan Trott[102], Sarah Watts[102], Zoe Friar[102], Abigail Speight[102].

13.1.53 Bedford Hospital, Bedford, UK

Victoria Bastion[103], Humza Chandna[103], Brice Djeugam[103], Muhammad Haseeb[103], Harriet Kent[103], Gamu Lubimbi[103], Sophie Murdoch[103], Alastair Thomas[103], Beena David[103], Rachel Lorusso[103], Ana Vochin[103], Melchizedek Penacerrada[103], Retno Wulandari[103].

13.1.54 Southport and Formby District General Hospital, Ormskirk, UK

Charlotte Heath[104], Srinivas Jakkula[104], Anna Morris[104], Ashar Ahmed[104], Arvind Nune[104], Claire Buttriss[104], Emma Whitaker[104].

13.1.55 The Tunbridge Wells Hospital and Maidstone Hospital, Kent, UK

Miriam Davey[105], David Golden[105], Amy Acklery[105], Fabio Fernandes[105], Bec Seaman[105], Victoria Earl[105].

13.1.56 Queen Elizabeth Hospital, Woolwich, London, UK

Amy Collins[106], Waqas Khaliq[106], Rachel Adam[106], Estefania Treus[106].

13.1.57 North Manchester General Hospital, Manchester, UK

Sarah Holland[107], Jordan Alfonso[107], Bethan Blackledge[107], Michelle Bruce[107], Laura Jayne Durrans[107], Ayaa Eltayeb[107], Jade Harris[107], Samuel Hey[107], Martin Hruska[107], Thomas Lamb[107], Joanne Rothwell[107], Adele Fitzgerald[107], Gabriella Lindergard[107], Helen T-Michael[107], Tracey Duncan[107], Sharon Baxter-Dore[107], Lisa Cooper[107], Claire Fox[107], Jacinta Guerin[107], Tracey Hodgkiss[107], Karen Connolly[107].

13.1.58 Royal Victoria Infirmary, Newcastle Upon Tyne, UK

Paul McAlinden[108], Victoria Bridgett[108], Maggie Fearby[108], A Gulati[108], Helen Hanson[108], Sinead Kelly[108], Louise McCormack[108], Rachel Nixon[108], Philip Robinson[108], Victoria Slater[108], Elaine Stephenson[108], Andrea Webster[108], K Webster[108], Carole Hays[108], Anne Hudson[108], Bijal Patel[108], Ian Clement[108], John Davis[108], Sarah Francis[108], Douglas Jerry[108].

13.1.59 Hull Royal Infirmary, Hull, UK

Caroline Abernathy[109], Louise Foster[109], Andrew Gratrix[109], Llucia Cabral-Ortega[109], Matthew Hines[109], Victoria Martinson[109], Elizabeth Stones[109], Karen Winter[109].

13.1.60 Manchester Royal Infirmary, Manchester, UK

Esther Barrow[110], Katharine Wylie[110], Deborah Baines[110], Katie Birchall[110], Laurel Kolakaluri[110], Richard Clark[110], Anila Sukumaran[110], Craig Brandwood[110], Melanie Barker[110], Deborah Paripoorani[110], Lara Smith[110], Charlotte Taylor[110].

13.1.61 Royal Derby Hospital, Derby, UK

Charlotte Downes[111], Melanie Hayman[111], Katie Riches[111], Priya Daniel[111], Deepak Subramanian[111], Kathleen Holding[111], Mary Hilton[111], Carly Mcdonald[111], Georgina Richardson[111].

13.1.62 Aintree University Hospital, Liverpool, UK

Georgia Halladay[112], Peter Harding[112], Amie Reddy[112], Ian Turner-Bone[112], Laura Wilding[112], Robert Parker[112], Michaela Lloyd[112], Leanne Smith[112], Charlie Kelly[112].

13.1.63 Fairfield General Hospital, Bury, UK

Maria Lazo[113], Alan Neal[113], Olivia Walton[113], Julie Melville[113], Jay Naisbitt[113], Emily Bullock[113], Rosane Joseph[113].

13.1.64 Norfolk and Norwich University hospital (NNUH), Norwich, UK

Sara Callam[114], Lisa Hudig[114], Jocelyn Keshet-Price[114], Katie Stammers[114], Karen Convery[114], Georgina Randell[114], Deirdre Fottrell-gould[114].

13.1.65 Milton Keynes University Hospital, Milton Keynes, UK

Esther Mwaura[115], Sara-Beth Sutherland[115], Richard Stewart[115], Louise Mew[115], Lynn Wren[115].

13.1.66 Good Hope Hospital, Birmingham, UK

Laura Thrasyvoulou[116], Heather Willis[116], James Scriven[116], Bridget Hopkins[116], Daniel Lenton[116], Abigail Roberts[116].

13.1.67 Queen Elizabeth Hospital Gateshead, Gateshead, UK

Maria Bokhari[117], Rachael Lucas[117], Wendy McCormick[117], Jenny Ritzema[117], Vanessa Linnett[117], Amanda Sanderson[117], Helen Wild[117].

13.1.68 Royal Bolton Hospital, Bolton, UK

Rebecca Flanagan[118], Robert Hull[118], Kat Rhead[118], Emma McKenna[118], Gareth Hughes[118], Jennifer Anderson[118], Kelly Jones[118], Scott Latham[118], Heather Riley[118].

13.1.69 Tameside General Hospital, Ashton Under Lyne, UK

Martina Coulding[119], Martyn Clark[119], Jacqueline McCormick[119], Oliver Mercer[119], Darsh Potla[119], Hafiz Rehman[119], Heather Savill[119], Victoria Turner[119], Edward Jude[119], Susan Kilroy[119].

13.1.70 Salford Royal Hospital, Manchester, UK

Elena Apetri[120], Cathrine Basikolo[120], Bethan Blackledge[120], Laura Catlow[120], Matthew Collis[120], Reece Doonan[120], Jade Harris[120], Alice Harvey[120], Karen Knowles[120], Stephanie Lee[120], Diane Lomas[120], Chloe Lyons[120], Liam McMorrow[120], Angiy Michael[120], Jessica Pendlebury[120], Jane Perez[120], Maria Poulaka[120], Nicola Proudfoot[120], Kathryn Slevin[120], Vicky Thomas[120], Danielle Walker[120], Paul Dark[120], Bethan Charles[120], Danielle McLaughlan[120], Melanie Slaughter[120], Dan Horner[120], Kathryn Cawley[120], Tracy Marsden[120].

13.1.71 Great Ormond St Hospital and UCL Great Ormond St Institute of Child Health NIHR Biomedical Research Centre, London, UK

Joyann Andrews[121], Emily Beech[121], Olugbenga Akinkugbe[121], Alasdair Bamford[121], Holly Belfield[121], Gareth A. L. Jones[121], Tara McHugh[121], Hamza Meghari[121], Samiran Ray[121], Ana Luisa Tomas[121], Lauran O'Neill[121], Mark Peters[121], Michael Bell[121], Sarah Benkenstein[121], Catherine Chisholm[121], Charlene Davies[121], Klaudia Kupiec[121], Caroline Payne[121].

13.1.72 Southmead Hospital, Bristol, UK

Joanna Halls[122], Hayley Blakemore[122], Elizabeth Goff[122], Kati Hayes[122], Kerry Smith[122], Deanna Stephens[122], Ruth Worner[122], Borislava Borislavova[122], Beverley Faulkner[122], Matt Thomas[122], Ruth Cookson[122], Emma Gendall[122], Georgina Larman[122], Rebecca Pope[122], Artur Smalira[122].

13.1.73 William Harvey Hospital, Ashford, UK

Victoria Priestley[123], Tracey Cosier[123], Gemma Millen[123], James Rand[123], Natasha Schumacher[123], Roxana Sandhar[123], Heather Weston[123], Neil Richardson[123], Lucy Cooper[123].

13.1.74 Arrowe Park Hospital, Wirral, UK

Cathy Jones[124], Ya-Wen Jessica Huang[124], Reni Jacob[124], Craig Denmade[124], Lewis Mcintyre[124].

13.1.75 Royal Hampshire County Hospital, Hampshire, UK

Dawn Trodd[125], Jane Martin[125], Geoff Watson[125], Emily Bevan[125], Caroline Wreybrown[125].

13.1.76 Bradford Royal Infirmary, Bradford, UK

Shereen Bano[126], Ruth Bellwood[126], Michael Bentley[126], Matt Bromley[126], Lucy Gurr[126], Camilla Ledgard[126], Janet McGowan[126], Kate Pye[126], Kirsten Sellick[126], Amelia Stacey[126], Deborah Warren[126], Brian Wilkinson[126], Louise Akeroyd[126], Huma Shafique[126], James Morgan[126], Susan Shorter[126], Rachel Swinger[126], Emily Waters[126], Tom Lawton[126].

13.1.77 Glan Clwyd Hospital, Bodelwyddan, UK

Elizabeth Allan[127], Kate Darlington[127], Ffyon Davies[127], Llinos Davies[127], Jack Easton[127], Sumit Kumar[127], Richard Lean[127], Callum Mackay[127], Richard Pugh[127], Xinyi Qiu[127], Stephanie Rees[127], Jeremy Scanlon[127], Joanne Lewis[127], Daniel Menzies[127], Annette Bolger[127], Gwyneth Davies[127], Jennifer Davies[127], Esther Garrod[127], Helen Jones[127], Rachel Manley[127], Hannah Williams[127].

13.1.78 Royal Bournemouth Hospital, Bournemouth, UK

Jordan Frankham[128], Sally Pitts[128], Nigel White[128], Debbie Branney[128], Heather Tiller[128].

13.1.79 Bristol Royal Infirmary, Bristol, UK

Georgia Efford[129], Zoe Garland[129], Lisa Grimmer[129], Bethany Gumbrill[129], Rebekah Johnson[129], Katie Sweet[129], Jeremy Bewley[129], Christina Coleman[129], Katie Corcoran[129], Eva Maria Hernandez Morano[129], Rachel Shiel[129], Denise Webster[129], Josephine Bonnici[129], Eleanor Daniel[129], Abbie Dell[129].

13.1.80 University Hospital North Durham, Darlington, UK and Darlington Memorial Hospital, Darlington, UK

Melanie Kent[130], Ami Wilkinson[130], Ellen Brown[130], Andrea Kay[130], Suzanne Campbell[130], Amanda Cowton[130], Mark Birt[130], Vicki Greenaway[130], Kathryn Potts[130], Clare Hutton[130], Andrew Shepperson[130].

13.1.81 Basildon Hospital, Basildon, UK

Miranda Forsey[131], Alice Nicholson[131], Mark Vertue[131], Joanne Riches[131], Agilan Kaliappan[131], Anne Nicholson[131].

13.1.82 University College Hospital, London, UK

Niall MacCallum[132], Eamon Raith[132], Georgia Bercades[132], Ingrid Hass[132], David Brealey[132], Gladys Martir[132], Anna Reyes[132], Deborah Smyth[132], Maria Zapatamartinez[132].

13.1.83 Whittington Hospital, London, UK

Ana Alvaro[133], Champa Jetha[133], Louise Ma[133], Lauren Booker[133], Loreta Mostoles[133], Anezka Pratley[133], Abdelhakim Altabaibeh[133], Chetan Parmar[133], Kayleigh Gilbert[133].

13.1.84 Western General Hospital, Edinburgh, UK

Susie Ferguson[134], Amy Shepherd[134], Sheila Morris[134], Jo Singleton[134], Rosie Baruah[134], Maria Amamio[134], Sophie Birch[134], Kate Briton[134], Sarah Clark[134], Katherine Doverman[134], Lucy Marshall[134], Scott Simpson[134].

13.1.85 Ipswich Hospital, Ipswich, UK

Georgina Lloyd[135], Stephanie Bell[135], Vanessa Rivers[135], Bally Purewal[135].

13.1.86 Hereford County Hospital, Hereford, UK

Kate Hammerton[136], Susan Anderson[136], Janine Birch[136], Emma Collins[136], Ryan Oleary[136].

13.1.87 Sunderland Royal Hospital, Sunderland, UK

Sarah Cornell[137], Jordan Jarmain[137], Kimberley Rogerson[137], Fiona Wakinshaw[137], Lindsey Woods[137], Anthony Rostron[137], Zeynep Elcioglu[137], Alistair Roy[137].

13.1.88 Queens Hospital Burton, Burton-On-Trent, UK

Gillian Bell[138], Holly Dickson[138], Louise Wilcox[138], Amro Katary[138], Katy English[138].

13.1.89 Musgrove Park Hospital, Taunton, UK

Joanne Hutter[139], Corinne Pawley[139], Patricia Doble[139], Charmaine Shovelton[139], Marius Vaida[139], Rebecca Purnell[139], Ashly Thomas[139].

13.1.90 The Royal Papworth Hospital, Cambridge, UK

Lenka Cagova[140], Adama Fofano[140], Helen Holcombe[140], Alice Michael Mitchell[140], Lucy Mwaura[140], KrithivasanÂ Praman[140], Lucie Garnr[140], Sue Mepham[140], Kitty Paques[140], Alain Vuylsteke[140], Jennifer Mackie[140], Carmen Pearn[140], Julie Zamikula[140].

13.1.91 University Hospital Lewisham, London, UK

Mark Birt[141], Estefania Treus Gude[141], Maggie Nyirenda[141], Lisa Capozzi[141], Rosie Reece-Anthony[141], Waqas Khaliq[141], Hazma Noor[141], Alfa Cresia Nilo[141].

13.1.92 The Princess Alexandra Hospital, Harlow, UK

Michelle Grove[142], Amelia Daniel[142], Amy Easthope[142], Joanne Finn[142], Nikki White[142], Rajnish Saha[142], Bibi Badal[142], Karen Ixer[142].

13.1.93 University Hospital of Wales, Cardiff, UK

Donna Duffin[143], Ben Player[143], Helen Hill[143], Jade Cole[143], Jenny Brooks[143], Michelle Davies[143], Rhys Davies[143], Lauren Hunt[143], Emma Thomas[143], Angharad Williams[143].

13.1.94 West Middlesex Hospital, Isleworth, UK

Metod Oblak[144], Mini Thankachen[144], Jamie Irisari[144], Amrinder Sayan[144], Monica Popescu[144].

13.1.95 Royal Albert Edward Infirmary, Wigan, UK

Cheryl Finch[145], Andrew Jamieson[145], Alison Quinn[145], Joshua Cooper[145], Sarah Liderth[145], Natalia Waddington[145].

13.1.96 Stoke Mandeville Hospital, Buckinghamshire, UK

Iona Burn[146], Katarina Manso[146], Ruth Penn[146], Julie Tebbutt[146], Danielle Thornton[146], James Winchester[146], Geraldine Hambrook[146], Pradeep Shanmugasundaram[146].

13.1.97 Royal Lancaster Infirmary, Lancaster, UK

Jayne Craig[147], Kerry Simpson[147], Andrew Higham[147], Louise Sibbett[147].

13.1.98 Basingstoke and North Hampshire Hospital, Basingstoke, UK

Sheila Paine[148], Annabel Reed[148], Jo-Anna Conyngham[148], Mcdonald Mupudzi[148], Rachel Thomas[148], Mary Wright[148], Denise Griffin[148], Richard Partridge[148], Maria Alvarez Corral[148], Nycola Muchenje[148], Mildred Sitonik[148], Caroline Wrey Brown[148].

13.1.99 Worthing Hospital, Worthing, UK and St Richard's Hospital, Chichester, UK

Aaron Butler[149], Linda Folkes[149], Heather Fox[149], Amy Gardner[149], David Helm[149], Gillian Hobden[149], Kirsten King[149], Jordi Margalef[149], Michael Margarson[149], Tim Martindale[149], Emma Meadows[149], Dana Raynard[149], Yvette Thirlwall[149], Yolanda Baird[149], Raquel Gomez[149], Darren Martin[149], Luke Hodgson[149], Clinton Corin[149], Erikka Sidall[149], Densie Szabo[149], Sharon Floyd[149].

13.1.100 The Alexandra Hospital, Redditch and Worcester Royal Hospital, Worcester, UK

Hannah Davies[150], Karen Austin[150], Olivia Kelsall[150], Hannah Wood[150], Hannah Davies[150], Peter Anderson[150], Katie Archer[150], Andrew Burtenshaw[150], Sarah Clayton[150], Naiara Cother[150], Nicholas Cowley[150], Caroline Davis[150], Stephen Digby[150], Alison Durie[150], Alison Harrison[150], Emma Low[150], Michael McAlindon[150], Alex McCurdy[150], Aled Morgan[150], Tobias Rankin[150], Jessica Thrush[150], Helen Tranter[150], Charlie Vigurs[150], Laura Wild[150], Karen Austin[150], Olivia Kelsall[150], Hannah Wood[150].

13.1.101 Royal Cornwall Hospital, Truro, UK

Thomas Cornell[151], Kate Ralph[151], Sarah Bean[151], Karen Burt[151], Michael Spivey[151], Carol Richards[151], Rachel Tedstone[151].

13.1.102 Watford General Hospital, Watford, UK

Siobhain Carmody[152], Xiaobei Zhao[152], Valerie Page[152], Mark Louie Guanco[152], Elvira Hoxha[152], Camilla Zorloni[152].

13.1.103 Macclesfield District General Hospital, Macclesfield, UK

Charlotte Dean[153], Emma Jones[153], Emma Carter[153], Joshua Dunn[153], Thomas Kong[153], Mervin Mahenthran[153], Chris Marsh[153], Maureen Holland[153], Natalie Keenan[153], Mohamed Mahmoud[153], Marc Lyons[153], Joanne Bradley-Potts[153], Helen Wassall[153], Meghan Young[153].

13.1.104 Royal Surrey County Hospital, Guildford, UK

Paul Bradley[154], Dorota Burda[154], Sinead Donlon[154], Lesley Harden[154], Celia Harris[154], Irving Mayangao[154], Rugia Montaser[154], Sheila Mtuwa[154], Charles Piercy[154], Eleanor Smith[154], Sarah Stone[154], Jerik Verula[154], Helen Blackman[154], Cheryl Marriott[154], Natalia Michalak[154], Ben Creagh-Brown[154], Armorel Salberg[154], Naomi Boyer[154], Veronika Pristopan[154].

13.1.105 Rotherham General Hospital, Rotherham, UK

Victoria Maynard[155], Rachel Walker[155], Anil Hormis[155], Dawn Collier[155], Cheryl Graham[155], Vicky Maynard[155], Jake McCormick[155], Jake Warrington[155].

13.1.106 Craigavon Area Hospital, County Armagh, NI

Denise Cosgrove[156], Denise McFarland[156], Judith Ratcliffe[156], Rob Charnock[156].

13.1.107 King's Mill Hospital, Nottingham, UK

Inez Wynter[157], Mandy Gill[157], Jill Kirk[157], Paul Paul[157], Valli Ratnam[157], Sarah Shelton[157].

13.1.108 Dumfries and Galloway Royal Infirmary, Dumfries, UK

Catherine Jardine[158], Alasdair Hay[158], Dewi Williams[158].

13.1.109 Prince Charles Hospital, Merthyr Tydfil, UK

Bethan Deacon[159], Latha Durga[159], Meg Hibbert[159], Gareth Kennard-Holden[159], Chrsitopher Woodford[159], Carla Pothecary[159], Lisa Roche[159], Dariusz Tetla[159], Kevin Agravante[159], Justyna Smeaton[159].

13.1.110 Ysbyty Gwynedd, Bangor, UK

Alicia Price[160], Alice Thomas[160], Chris Thorpe[160], Ellen Knights[160], Donna Ward[160].

13.1.111 Royal Preston Hospital, Preston, UK

Shondipon Laha[161], Mark Verlander[161], Alexandra Williams[161].

13.1.112 The Great Western Hospital, Swindon, UK

Rachel Prout[162], Helen Langton[162], Malcolm Watters[162], Charlotte Hunt[162], Catherine Novis[162].

13.1.113 Lincoln County Hospital, Lincoln, UK

Sarwat Arif[163], Amy Cunningham[163], Claire Hewitt[163], Julia Hindale[163], Karen Jackson-Lawrence[163], Sarah Shepardson[163], Maryanne Wills[163], Susie Butler[163], Silivia Tavares[163], Russell Barber[163], Annette Hilldrith[163], Kelly Hubbard[163].

13.1.114 University Hospital of North Tees, Stockton on Tees, UK

Dawn Egginton[164], Michele Clark[164], Sarah Purvis[164], Simon Sinclair[164], Vicky Collins[164].

13.1.115 Glangwili General Hospital, Camarthen, UK

Bethan Landeg[165], Craig Sell[165], Samantha Coetzee[165], Alistair Gales[165], Igor Otahal[165], Becky Icke[165], Meena Raj[165], Caroline Williams[165], Jill Williams[165], Lucy Hill[165].

13.1.116 Southend University Hospital, Westcliff-on-Sea, UK

Abdul Kayani[166], Bridgett Masunda[166], Prisca Gondo[166], Nigara Atayeva[166].

13.1.117 Lister Hospital, Stevenage, UK

Carina Cruz[167], Natalie Pattison[167].

13.1.118 Diana Princess of Wales Hospital, Grimsby, UK

Caroline Burnett[168], Jonathan Hatton[168], Elaine Heeney[168], Maria Newton[168], Hassan Al-Moasseb[168], Teresa Behan[168], Jasmine Player[168], Rachael Stead[168], Atideb Mitra[168], Kirsty Nauyokas[168].

13.1.119 West Suffolk Hospital, Bury St Edmunds, UK

Sally Humphreys[169], Helen Cockerill[169], Ruth Tampsett[169].

13.1.120 Victoria Hospital, Kirkcaldy, UK

Evgeniya Postovalova[170], Tina Coventry[170], Amanda McGregor[170], Susan Fowler[170], Mike Macmahon[170], Patricia Cochrane[170], Sandra Pirie[170].

13.1.121 Calderdale Royal Hospital, Halifax, UK and Huddersfield Royal Infirmary, Huddersfield, UK

Sarah Hanley[171], Asifa Ali[171], Megan Brady[171], Sam Dale[171], Annalisa Dance[171], Lisa Gledhill[171], Jill Greig[171], Kathryn Hanson[171], Kelly Holdroyd[171], Marie Home[171], Tahira Ishaq[171], Diane Kelly[171], Lear Matapure[171], Deborah Melia[171], Samantha Mellor[171], Ekta Merwaha[171], Tonicha Nortcliffe[171], Lisa Shaw[171], Ryan Shaw[171], Tracy Wood[171], Lee-Ann Bayo[171], Miranda Usher[171], Alison Wilson[171], Ross Kitson[171], Jez Pinnell[171], Matthew Robinson[171], Kaitlin Boltwood[171].

13.1.122 Dorset County Hospital, Dorchester, UK

Jenny Birch[172], Laura Bough[172], Rebecca Tutton[172], Barbara Winter-Goodwin[172], Josie Goodsell[172], Kate Taylor[172], Patricia Williams[172], Sarah Williams[172], Ashleigh Cave[172], James Rees[172].

13.1.123 Russell's Hall Hospital, Dudley, UK

Janet Imeson-Wood[173], Jacqueline Smith[173], Vishal Amin[173], Komala Karthik[173], Rizwana Kausar[173], Elena Anastasescu[173], Karen Reid[173], Vikram Anumakonda[173], Ella Stoddart[173].

13.1.124 Royal United Hospital, Bath, UK

Carrie Demetriou[174], Charlotte Eckbad[174], Lucy Howie[174], Sarah Mitchard[174], Lidia Ramos[174], Katie White[174], Sarah Hierons[174], Fiona Kelly[174], Alfredo Serrano-Ruiz[174], Gabrielle Evans[174].

13.1.125 St Mary's Hospital, Newport, UK

Liz Nicol[175], Joy Wilkins[175], Kim Hulacka[175], Gabor Debreceni[175], Alison Brown[175], Vikki Crickmore[175].

13.1.126 George Eliot Hospital NHS Trust, Nuneaton, UK

Kay Hill[176], Thogulava Kannan[176].

13.1.127 Yeovil Hospital, Yeovil, UK

Zenaida Dagutao[177], Kate Beesley[177], Alison Lewis[177], Jess Perry[177], Sherly Antony[177], Sarah Board[177], Clare Buckley[177], Lucy Pippard[177], Alfonso Tanate[177], Diane Wood[177], Agnieska Kubisz-Pudelko[177], Ayman Gouda[177].

13.1.128 Forth Valley Royal Hospital, Falkirk, UK

Fiona Auld[178], Joanne Donnachie[178], Euan Murdoch[178], Lynn Prentice[178], Nikole Runciman[178], Dhaneesha Senaratne[178], Abigail Short[178], Laura Sweeney[178], Lesley Symon[178], Anne Todd[178], Patricia Turner[178], Erin McCann[178], Dario Salutous[178], Ian Edmond[178], Lesley Whitelaw[178].

13.1.129 Frimley Park Hospital, Surrey, UK

Harish Venkatesh[179], Yvonne Bland[179], Istvan Kajtor[179], Lisa Kavanagh[179], Karen Singler[179], George Linfield-Brown[179].

13.1.130 Chelsea & Westminster NHS Foundation Trust, London, UK

Luke Stephen Prockter Moore[180], Marcela Vizcaychipi[180], Laura Martins[180], Luke Moore[180], Rhian Bull[180], Jaime Carungcong[180].

13.1.131 Queen Elizabeth the Queen Mother Hospital, Margate, UK

Louise Allen[181], Eva Beranova[181], Alicia Knight[181], Carly Price[181], Sorrell Tilbey[181], Sharon Turney[181], Tracy Hazelton[181], Gabriella Tutt[181], Mansi Arora[181], Salah Turki[181], Emily Sinfield[181], Joanne Deery[181], Hazel Ramos[181].

13.1.132 Royal Brompton Hospital, London, UK

Daniele Cristiano[182], Natalie Dormand[182], Zohreh Farzad[182], Mahitha Gummadi[182], Sara Salmi[182], Geraldine Sloane[182], Mathew Varghese[182], Vicky Thwaites[182], Brijesh Patel[182], Liyanage Kamal[182], Anelise Catelan Zborowski[182].

13.1.133 Darent Valley Hospital, Dartford, UK

Ryan Coe[183], Madeleine Anderson[183], Jane Beadle[183], Charlotte Coates[183], Katy Collins[183], Maria Crowley[183], Laura Johnson[183], Laura King[183], Remi Paramsothy[183], Janet Sargeant[183], Pedro Silva[183], Carmel Stuart[183], June Taylor[183], David Tyl[183], Phillipa Wakefield[183], Charlotte Kamundi[183], Olumide Olufuwa[183], Zakaulla Belagodu[183], Anca Gherman[183], Naomi Oakley[183].

13.1.134 University Hospital Crosshouse, Kilmarnock, UK

John Allan[184], Tim Geary[184], Alistair Meikle[184], Peter O'Brien[184], Stephen Wood[184], Andrew Clark[184], Gordon Houston[184].

13.1.135 University Hospital Wishaw, Wishaw, UK

Karen Black[185], Michelle Clarkson[185], Stuart D'Sylva[185], Alan Morrison[185], Kathryn Norman[185], Margaret Taylor[185], Suzanne Clements[185], Catriona Cohrane[185], Nora Gonzalez[185], Dominic Strachan[185], Claire Beith[185], Kirsten Moar[185].

13.1.136 University College Dublin, St Vincent's University Hospital, Dublin, Ireland

Lorna Murphy[186], Michelle Smythe[186], Alistair Nichol[186], Kathy Brickell[186].

13.1.137 The Queen Elizabeth Hospital, King's Lynn, UK

Inthakab Ali Mohamed Ali[187], Karen Beaumont[187], Mohamed Elsaadany[187], Kay Fernandes[187], Sameena Mohamed Ally[187], Harini Rangarajan[187], Varun Sarathy[187], Sivarupan Selvanayagam[187], Dave Vedage[187], Matthew White[187], Zoe Coton[187], Aricsa Joshy[187], Mark Blunt[187], Hollie Curgenven[187].

13.1.138 Walsall Manor Hospital, Walsall, UK

Liam Botfield[188], Catherine Dexter[188], Aditya Kuravi[188], Joanne Butler[188], Robert Chadwick[188], Poonam Ranga[188], Lisa Richardson[188], Emma Virgilio[188], Maddiha Anwer[188], Atul Garg[188], Donna Botfield[188], Xana Marriott[188].

13.1.139 Princess Royal Hospital, Brighton, UK

Keely Stewart[189], Dee Mullan[189], Claire Phillips[189], Jane Gaylard[189], Justyna Nowak[189], Denise Skinner[189].

13.1.140 Barnsley Hospital, Barnsley, UK

Sian Jones[190], Rikki Crawley[190], Abigail Crew[190], Mishell Cunningham[190], Allison Daniels[190], Laura Harrison[190], Susan Hope[190], Nicola Lancaster[190], Jamie Matthews[190], Gemma Wray[190], Alice Nicholson[190], Ken Inweregbu[190], Sarah Cutts[190], Katharine Miller[190].

13.1.141 Warrington General Hospital, Warrington, UK

Ailbhe Brady[191], Rebekah Chan[191], Shane McIvor[191], Helena Prady[191], Bijoy Mathew[191], Jeff Little[191], Tim Furniss[191].

13.1.142 Royal Victoria Hospital, Belfast, NI

Chris Wright[192], Bernadette King[192], Christopher Wasson[192], Aisling O'Neill[192], Christine Turley[192], Peter McGuigan[192], Erin Collins[192], Stephanie Finn[192], Jackie Green[192], Julie McAuley[192], Abitha Nair[192], Charlotte Quinn[192], Suzanne Tauro[192], Kathryn Ward[192], Michael McGinlay[192], Kiran Reddy[192].

13.1.143 Royal Hallamshire Hospital and Northern General Hospital, Sheffield, UK

Norfaizan Ahmad[193], Samantha Anderson[193], Joann Barker[193], Kris Bauchmuller[193], Kathryn Birchall[193], Sarah Bird[193], Kay Cawthron[193], Luke Chetam[193], Joby Cole[193], Ben Donne[193], David Foote[193], Amber Ford[193], Helena Hanratty[193], Kate Harrington[193], Lisa Hesseldon[193], Kay Housley[193], Yvonne Jackson[193], Claire Jarman[193], Faith Kibutu[193], Becky Lenagh[193], Irene Macharia[193], Shamiso Masuko[193], Leanne Milner[193], Helen Newell[193], Lorenza Nwafor[193], Simon Oxspring[193], Patrick Phillips[193], Ajay Raithatha[193], Sarah Rowland-Jones[193], Jacqui Smith[193], Roger Thompson[193], Helen Trower[193], Sara Walker[193], James Watson[193], Matthew Wiles[193], Alison Lye[193], Jayne Willson[193], Gary Mills[193], Sansha Harris[193], Eleanor Hartill[193].

13.1.144 Harefield Hospital, London, UK

Anthony Barron[194], Ciara Collins[194], Sundeep Kaul[194], Claire Nolan[194], Oliver Polgar[194], Claire Prendergast[194], Paula Rogers[194], Rajvinder Shokkar[194], Meriel Woodruff[194], Kanta Mahay[194], Vicky Thwaites[194], Anna Reed[194], Hayley Meyrick[194], Heather Passmore[194], James Farwell[194].

13.1.145 Cumberland Infirmary, Carlisle, UK

Alison Brown[195], Susan O'Connell[195], Jane Gregory[195], Luigi Barberis[195], Rosemary Harper[195], Tim Smith[195], Diane Armstrong[195].

13.1.146 Eastbourne District General Hospital, East Sussex, UK and Conquest Hospital, East Sussex, UK

Angie Bowey[196], Anne Cowley[196], Andrew Corner[196], Judith Highgate[196], Claire Rutherfurd[196], Jo-Anne Taylor[196], Sarah Goodwin[196], Claire Rutherford[196].

13.1.147 Salisbury District Hospital, Salisbury, UK

Beena Eapen[197], Fiona Trim[197], Phil Donnison[197].

13.1.148 Airedale General Hospital, Keighley, UK

Lisa Armstrong[198], Hayley Bates[198], Emma Dooks[198], Fiona Farquhar[198], Amy Kitching[198], Chantal McParland[198], Sophie Packham[198], Brigid Hairsine[198].

13.1.149 Leicester Royal Infirmary, Leicester, UK

Premetie Andreou[199], Dawn Hales[199], Megha Mathews[199], Rekha Patel[199], Peter Barry[199], Neil Flint[199], Jessica Hailstone[199], Navneet Ghuman[199], Bethany Leonard[199], Rachel Lees[199].

13.1.150 Peterborough City Hospital, Peterborough, UK and Hinchingbrooke Hospital, Huntingdon, UK

Deborah Butcher[200], Katy Leng[200], Nicola Butterworth-Cowin[200], Susie O'Sullivan[200].

13.1.151 Colchester General Hospital, Colchester, UK

Alison Ghosh[201], Emma Williams[201].

13.1.152 Princess Royal Hospital, Telford and Royal Shrewsbury Hospital, Shrewsbury, UK

Colene Adams[202], Anita Agasou[202], Tracie Arden[202], Mandy Beekes[202], Amy Bowes[202], Pauline Boyle[202], Heather Button[202], Mandy Carnahan[202], Anne Carter[202], Danielle Childs[202], Jane Gaylard[202], Fran Hurford[202], Yasmin Hussain[202], Ayesha Javaid[202], James Jones[202], Michael Leigh[202], Terry Martin[202], Helen Millward[202], Nichola Motherwell[202], Dee Mullan[202], Julie Newman[202], Rachel Rikunenko[202], Jo Stickley[202], Julie Summers[202], Louise Ting[202], Helen Tivenan[202], Denise Donaldson[202], Nigel Capps[202], Emily Cale[202], Sanal Jose[202], Wendy Osbourne[202], Susie Pajak[202], Jayne Rankin[202], Louise Tonks[202].

13.1.153 University Hospital Monklands, Airdrie, UK

Tracy Baird[203], Margaret Harkins[203], Jim Ruddy[203], Joe West[203].

13.1.154 Wrexham Maelor Hospital, Wrexham, Wales

Joseph Duffield[204], Lewis Mallon[204], Oliver Smith[204], Sara Smuts[204], Andy Campbell[204], Cate Davies[204], Sarah Davies[204], Rachel Hughes[204], Lisa Jobes[204], Victoria Whitehead[204], Clare Watkins[204].

13.1.155 Royal Hospital for Children, Glasgow, UK

Fiona Bowman[27], Barry Milligan[27], Colin Begg[27], Liane McPherson[27].

13.1.156 New Cross Hospital, Wolverhampton, UK

Stella Metherell[205], Nichola Harris[205], Victoria Lake[205], Elizabeth Radford[205], Andy Smallwood[205], Shameer Gopal[205], Katherine Vassell[205].

13.1.157 University Hospital Hairmyres, East Kilbride, UK

Dina Bell[206], Rosalind Boyle[206], Katie Douglas[206], Lynn Glass[206], Liz Lennon[206], Austin Rattray[206], Claire Beith[206], Emma Lee[206].

13.1.158 Warwick Hospital, Warwick, UK

Danielle Jones[207], Penny Parsons[207], Ben Attwood[207], Paul Jefferson[207], Mohan Ranganathan[207], Inderjit Atwal[207], Bridget Campbell[207], Angela Day[207], Camilla Stagg[207].

13.1.159 Sandwell General Hospital and City Hospital, Birmingham, UK

Emma Haynes[208], Cecilia Ahmed[208], Sarah Clamp[208], Julie Colley[208], Risna Haq[208], Anne Hayes[208], Sibet Joseph[208], Zahira Maqsood[208], Samia Hussain[208], Jonathan Hulme[208], Patience Domingos[208], Rita Kumar[208], Manjit Purewal[208], Becky Taylor[208].

13.1.160 Royal Manchester Children's Hospital, Manchester, UK

Lara Bunni[209], Monica Latif[209], Claire Jennings[209], Shilu Jose[209], Rebecca Marshall[209], Aleksandra Metryka[209], Gayathri Subramanian[209].

13.1.161 Gloucestershire Royal Hospital, Gloucester, UK

Adam Burgoyne[210], Susan O'Connell[210], Amanda Tyler[210], Joanne Waldron[210], Paula Hilltout[210], Jayne Evitts[210].

13.1.162 University Hospitals Coventry & Warwickshire NHS Trust, Coventry, UK

Geraldine Ward[211], Pamela Bremmer[211], Carl Hawkins[211], Sophie Jackman[211], Michal Ogorek[211].

13.1.163 Torbay Hospital, Torquay, UK

Kylie Ashby[212], Lorraine Thornton[212], Pauline Mercer[212], Matthew Halkes[212], Adam Revill[212].

13.1.164 Pilgrim Hospital, Lincoln, UK

Bryony Saint[213], Jo Fletcher[213], Kimberley Netherton[213], Manish Chablani[213], Amy Kirkby[213], Amanda Roper[213], Kinga Szymiczek[213].

13.1.165 Prince Philip Hospital, Lianelli, UK

Isobel Sutherland[214], Linda O'Brien[214], Igor Otahal[214], Joanne Connell[214], Kim Davies[214], Tracy Lewis[214], Zohra Omar[214], Emma Perkins[214].

13.1.166 Princess of Wales Hospital, Llantrisant, UK

Lisa Roche[215], Sonia Sathe[215], Ellie Davies[215].

13.1.167 Northampton General Hospital NHS Trust, Northampton, UK

Alex Lyon[216], Isheunesu Mapfunde[216], Charlotte Willis[216], Rachael Hitchcock[216], Kathryn Hall[216], Christopher King[216].

13.1.168 The Christie NHS Foundation Trust, Manchester, UK

Andrew Fagan[217], Roonak Nazari[217], Lucy Worsley[217], Suzanne Allibone[217], Vidya Kasipandian[217], Amit Patel[217], Parisa Cutting[217], Roman Genetu[217], Ainhi Mac[217], Anthony Murphy[217], Sinead Ward[217], Fatima Butt[217].

13.1.169 James Paget University Hospital NHS Trust, Great Yarmouth, UK

Amanda Ayers[218], Wendy Harrison[218], Katherine Mackintosh[218], Julie North[218].

13.1.170 Birmingham Children's Hospital, Birmingham, UK

Lydia Ashton[219], Rehana Bi[219], Samantha Owen[219], Helen Winmill[219], Barney Scholefield[219].

13.1.171 Withybush General Hospital, Pembrokeshire, Wales

Hannah Blowing[220], Erin Williams[220], Michaela Duskova[220], Michelle Edwards[220], Alun Rees[220], Helen Thomas[220], Rachel Hughes[220], Igor Otahal[220], Jolene Brooks[220], Janet Phipps[220], Suzanne Brooks[220].

13.1.172 Northwick Park Hospital, London, UK

Catherine Dennis[221], Vicki Parris[221], Sinduya Srikaran[221], Anisha Sukha[221], Alistair McGregor[221], Gerlynn Tiongson[221].

13.1.173 North Devon District Hospital, Barnstaple, UK

Katie Adams[222], Benedict Andrew[222], Adam Brayne[222], Sasha Carter[222], Louise Findlay[222], Emma Fisher[222], Peter Jackson[222], Duncan Kaye[222], Juliet Parkin[222], Victoria Tuckey[222], Jane Hunt[222], Nicholas Love[222], Lynne van Koutrick[222], Ashley Hanson[222].

13.1.174 Scunthorpe General Hospital, Scunthorpe, UK

Kathy Dent[223], Elizabeth Horsley[223], Sandra Pearson[223], Sue Spencer[223], Dorothy Hutchinson[223], Jasmine Player[223], Dorota Potoczna[223], Muhammad Nauman Akhtar[223], Lisa-Jayne Cottam[223], Kirsty Nauyokas[223], Jack Sanders[223].

13.1.175 Royal Free Hospital, London, UK

Sara Mingo Garcia[224], Glykeria pakou[224], Cynthia Diaba[224], Helder Filipe[224], Lincy John[224], Amitaa Maharajh[224], Mark de Neef[224], Daniel Martin[224], Christine Eastgate[224], Poh Choo Teoh[224].

13.1.176 Raigmore Hospital, Inverness, UK

Fiona Barrett[225], Clare Bradley[225], Avril Donaldson[225], Mairi Mascarenhas[225], Marianne O'Hara[225], Laura Okeefe[225], Noreen Clarke[225], Jonathan Whiteside[225], Rachael Campbell[225], Joanna Matheson[225], Deborah McDonald[225], Donna Patience[225].

13.1.177 West Cumberland Hospital, Whitehaven, UK

Polly Rice[226], Tim Smith[226], Melanie Clapham[226], Rachel Mutch[226], Luigi Barberis[226], Rosemary Harper[226], Hannah Craig[226], Una Poultney[226].

13.1.178 Furness General Hospital, Barrow-in-Furness, UK

Karen Burns[227], Andrew Higham[227].

13.1.179 Liverpool Heart and Chest Hospital, Liverpool, UK

Sophie Twiss[228], Janet Barton[228], Linsha George[228], Clare Harrop[228], Sherly Mathew[228], David Justin Wright[228].

13.1.180 Scarborough General Hospital, Scarborough, UK

Rachel Harrison[229], Jordan Toohie[229], Ben Chandler[229], Alison Turnbull[229], Janine Mallinson[229], Kerry Elliott[229].

13.1.181 Bronglais General Hospital, Aberystwyth, UK

Rebecca Wolf-Roberts[230], Helen Tench[230], Igor Otahal[230], Maria Hobrok[230], Ronda Loosley[230], Heather McGuinness[230], Tanya Sims[230].

13.1.182 Alder Hey Children's Hospital, Liverpool, UK

Deborah Afolabi[231], Kathryn Sian Allison[231], Taya Anderson[231], Rachael Dore[231], Dawn Jones[231], Naomi Rogers[231], Paula Saunderson[231], Jennifer Whitbread[231], Laura O'Malley[231], Laura Rad[231], Daniel Hawcutt[231].

13.1.183 Borders General Hospital, Melrose, UK

Jonathan Aldridge[232], Melanie Tolson[232], Sweyn Garrioch[232].

13.1.184 Leighton Hospital, Cheshire, UK

Joanne Tomlinson[233], Michael Grosdenier[233].

13.1.185 Kent & Canterbury Hospital, Canterbury, UK

David Loader[234], Ritoo Kapoor[234], Gemma Hector[234].

13.1.186 Harrogate and District NHS Foundation Trust, Harrogate, UK

Joslan Scherewode[235], Chunda Sri-Chandana[235], Lorraine Stephenson[235], Sarah Marsh[235].

13.1.187 The Royal Marsden Hospital, London, UK

Arnold Dela Rosa[236], Shaman Jhanji[236], Thomas Bemand[236], Ryan Howle[236], Ravishankar Rao Baikady[236], Benjamin Thomas[236], Ethel Black[236], Kate Tatham[236].

13.1.188 Ealing Hospital, Southall, UK

Sambasivarao Gurram[237], Ekaterina Watson[237], Vicki Parris[237], Sheena Quaid[237], Alistair McGregor[237].

13.1.189 St John's Hospital Livingston, Livingston, UK

Anne Saunderson[238], Rachel O'Brien[238], Sam Moultrie[238], Jen Service[238], Clare Cheyne[238], Miranda Odam[238], Alison Wiliams[238].

13.1.190 Wexham Park Hospital, Slough, UK

Nicky Barnes[239], Peter Csabi[239], Joana Da Rocha[239], Louika Glynou[239].

13.1.191 Sheffield Children's Hospital, Sheffield, UK

Amy Huffenberger[240], Jade Bryant[240], Amy Pickard[240], Nicholas Roe[240], Arianna Bellini[240], Anton Mayer[240], Amy Burrow[240], Natalie Colley[240], Jayne Evans[240], Alex Howlett[240], Zeinab Khalifeh@nhs.net[240].

13.1.192 Homerton University Hospital Foundation NHS Trust, London UK

Jerldine Pryce[241], Claire Gorman[241], Amy Easthope[241], Rebecca Brady[241], Elizabeth Timlick[241], Pierre Antoine[241], Abhinhav Gupta[241].

13.1.193 National Hospital for Neurology and Neurosurgery, London, UK

John Hardy[242], Henry Houlden[242], Eleanor Moncur[242], Arianna Tucci[242], Eamon Raith[242], Ambreen Tariq[242], David Brealey[242].

13.1.194 The Royal Alexandra Children's Hospital, Brighton, UK

Emma Tagliavini[243], Becky Ramsay[243], Katy Fidler[243], Kevin Donnelly[243], Rebecca Hollis[243].

13.1.195 Golden Jubilee National Hospital, Clydebank, UK

Jocelyn Barr[244], Elizabeth Boyd[244], Val Irvine[244], Ben Shelley[244], Julie Buckley[244], Charlene Hamilton[244], Kathryn Valdeavella[244].

[49] NIHR Clinical Research Network (CRN), North West London Core Team, 3rd Floor Administrative Block South, Clock Tower, Hammersmith Hospital, Du Cane Road, London W12 0HS
[50] Cambridge University Hospitals NHS Foundation Trust, Hills Road, Cambridge, CB2 0QQ, UK
[51] Biostatistics Group, State Key Laboratory of Biocontrol, School of Life Sciences, Sun Yat-sen University, Guangzhou, China
[52] Department of Infectious Diseases, Leiden University Medical Center, Leiden, The Netherlands
[53] Guys and St Thomas' Hospital, London, UK
[54] James Cook University Hospital, Middlesbrough, UK
[55] Barts Health NHS Trust, London, UK
[56] Royal Stoke University Hospital, Staffordshire, UK
[57] North Middlesex University Hospital NHS trust, London, UK
[58] King's College Hospital, London, UK
[59] Charing Cross Hospital, St Mary's Hospital and Hammersmith Hospital, London, UK
[60] The Royal Liverpool University Hospital, Liverpool, UK
[61] John Radcliffe Hospital, Oxford, UK
[62] Addenbrooke's Hospital, Cambridge, UK
[63] Nottingham University Hospital, Nottingham, UK
[64] St George's Hospital, London, UK
[65] BHRUT (Barking Havering) - Queens Hospital and King George Hospital, Essex, UK
[66] Royal Infirmary of Edinburgh, Edinburgh, UK
[67] Kingston Hospital, Surrey, UK
[68] Queen Alexandra Hospital, Portsmouth, UK
[69] Royal Gwent Hospital, Newport, UK
[70] Royal Blackburn Teaching Hospital, Blackburn, UK
[71] Stepping Hill Hospital, Stockport, UK
[72] Northumbria Healthcare NHS Foundation Trust, North Shields, UK
[73] Countess of Chester Hospital, Chester, UK
[74] Pinderfields General Hospital, Wakefield, UK

[75] Ninewells Hospital, Dundee, UK
[76] Croydon University Hospital, Croydon, UK
[77] Morriston Hospital, Swansea, UK
[78] Queen Elizabeth University Hospital, Glasgow, UK
[79] Broomfield Hospital, Chelmsford, UK
[80] Heartlands Hospital, Birmingham, UK
[81] Royal Sussex County Hospital, Brighton, UK
[82] York Hospital, York, UK
[83] Queen Elizabeth Hospital, Birmingham, UK
[84] Royal Glamorgan Hospital, Pontyclun, UK
[85] Barnet Hospital, London, UK
[86] Wythenshawe Hospital, Manchester, UK
[87] Medway Maritime Hospital, Gillingham, UK
[88] Royal Berkshire NHS Foundation Trust, Berkshire, UK
[89] Whiston Hospital, Prescot, UK
[90] The Royal Oldham Hospital, Manchester, UK
[91] Chesterfield Royal Hospital Foundation Trust, Chesterfield, UK
[92] Aberdeen Royal Infirmary, Aberdeen, UK
[93] Royal Devon and Exeter Hospital, Exeter, UK
[94] Glasgow Royal Infirmary, Glasgow, UK
[95] Blackpool Victoria Hospital, Blackpool, UK
[96] Southampton General Hospital, Southampton, UK
[97] Ashford and St Peter's Hospital, Surrey, UK
[98] Derriford Hospital, Plymouth, UK
[99] East Surrey Hospital, Redhill, UK
[100] Poole Hospital, Poole, UK
[101] Royal Alexandra Hospital, Paisley, UK
[102] St James's University Hospital and Leeds General Infirmary, Leeds, UK
[103] Bedford Hospital, Bedford, UK
[104] Southport and Formby District General Hospital, Ormskirk, UK
[105] The Tunbridge Wells Hospital and Maidstone Hospital, Kent, UK
[106] Queen Elizabeth Hospital, Woolwich, London, UK
[107] North Manchester General Hospital, Manchester, UK
[108] Royal Victoria Infirmary, Newcastle Upon Tyne, UK
[109] Hull Royal Infirmary, Hull, UK
[110] Manchester Royal Infirmary, Manchester, UK
[111] Royal Derby Hospital, Derby, UK
[112] Aintree University Hospital, Liverpool, UK
[113] Fairfield General Hospital, Bury, UK
[114] Norfolk and Norwich University hospital (NNUH), Norwich, UK
[115] Milton Keynes University Hospital, Milton Keynes, UK
[116] Good Hope Hospital, Birmingham, UK
[117] Queen Elizabeth Hospital Gateshead, Gateshead, UK
[118] Royal Bolton Hospital, Bolton, UK
[119] Tameside General Hospital, Ashton Under Lyne, UK
[120] Salford Royal Hospital, Manchester, UK
[121] Great Ormond St Hospital and UCL Great Ormond St Institute of Child Health NIHR Biomedical Research Centre, London, UK
[122] Southmead Hospital, Bristol, UK
[123] William Harvey Hospital, Ashford, UK
[124] Arrowe Park Hospital, Wirral, UK
[125] Royal Hampshire County Hospital, Hampshire, UK
[126] Bradford Royal Infirmary, Bradford, UK
[127] Glan Clwyd Hospital, Bodelwyddan, UK
[128] Royal Bournemouth Hospital, Bournemouth, UK
[129] Bristol Royal Infirmary, Bristol, UK
[130] University Hospital North Durham, Darlington, UK and Darlington Memorial Hospital, Darlington, UK
[131] Basildon Hospital, Basildon, UK
[132] University College Hospital, London, UK
[133] Whittington Hospital, London, UK
[134] Western General Hospital, Edinburgh, UK
[135] Ipswich Hospital, Ipswich, UK
[136] Hereford County Hospital, Hereford, UK
[137] Sunderland Royal Hospital, Sunderland, UK
[138] Queens Hospital Burton, Burton-On-Trent, UK
[139] Musgrove Park Hospital, Taunton, UK
[140] The Royal Papworth Hospital, Cambridge, UK
[141] University Hospital Lewisham, London, UK
[142] The Princess Alexandra Hospital, Harlow, UK
[143] University Hospital of Wales, Cardiff, UK
[144] West Middlesex Hospital, Isleworth, UK
[145] Royal Albert Edward Infirmary, Wigan, UK
[146] Stoke Mandeville Hospital, Buckinghamshire, UK
[147] Royal Lancaster Infirmary, Lancaster, UK
[148] Basingstoke and North Hampshire Hospital, Basingstoke, UK
[149] Worthing Hospital, Worthing, UK and St Richard's Hospital, Chichester, UK
[150] The Alexandra Hospital, Redditch and Worcester Royal Hospital, Worcester, UK
[151] Royal Cornwall Hospital, Truro, UK
[152] Watford General Hospital, Watford, UK
[153] Macclesfield District General Hospital, Macclesfield, UK
[154] Royal Surrey County Hospital, Guildford, UK
[155] Rotherham General Hospital, Rotherham, UK
[156] Craigavon Area Hospital, County Armagh, NI
[157] King's Mill Hospital, Nottingham, UK
[158] Dumfries and Galloway Royal Infirmary, Dumfries, UK
[159] Prince Charles Hospital, Merthyr Tydfil, UK
[160] Ysbyty Gwynedd, Bangor, UK
[161] Royal Preston Hospital, Preston, UK
[162] The Great Western Hospital, Swindon, UK
[163] Lincoln County Hospital, Lincoln, UK
[164] University Hospital of North Tees, Stockton on Tees, UK
[165] Glangwili General Hospital, Camarthen, UK

[166] Southend University Hospital, Westcliff-on-Sea, UK
[167] Lister Hospital, Stevenage, UK
[168] Diana Princess of Wales Hospital, Grimsby, UK
[169] West Suffolk Hospital, Bury St Edmunds, UK
[170] Victoria Hospital, Kirkcaldy, UK
[171] Calderdale Royal Hospital, Halifax, UK and Huddersfield Royal Infirmary, Huddersfield, UK
[172] Dorset County Hospital, Dorchester, UK
[173] Russell's Hall Hospital, Dudley, UK
[174] Royal United Hospital, Bath, UK
[175] St Mary's Hospital, Newport, UK
[176] George Eliot Hospital NHS Trust, Nuneaton, UK
[177] Yeovil Hospital, Yeovil, UK
[178] Forth Valley Royal Hospital, Falkirk, UK
[179] Frimley Park Hospital, Surrey, UK
[180] Chelsea & Westminster NHS Foundation Trust, London, UK
[181] Queen Elizabeth the Queen Mother Hospital, Margate, UK
[182] Royal Brompton Hospital, London, UK
[183] Darent Valley Hospital, Dartford, UK
[184] University Hospital Crosshouse, Kilmarnock, UK
[185] University Hospital Wishaw, Wishaw, UK
[186] University College Dublin, St Vincent's University Hospital, Dublin, Ireland
[187] The Queen Elizabeth Hospital, King's Lynn, UK
[188] Walsall Manor Hospital, Walsall, UK
[189] Princess Royal Hospital, Brighton, UK
[190] Barnsley Hospital, Barnsley, UK
[191] Warrington General Hospital, Warrington, UK
[192] Royal Victoria Hospital, Belfast, NI
[193] Royal Hallamshire Hospital and Northern General Hospital, Sheffield, UK
[194] Harefield Hospital, London, UK
[195] Cumberland Infirmary, Carlisle, UK
[196] Eastbourne District General Hospital, East Sussex, UK and Conquest Hospital, East Sussex, UK
[197] Salisbury District Hospital, Salisbury, UK
[198] Airedale General Hospital, Keighley, UK
[199] Leicester Royal Infirmary, Leicester, UK
[200] Peterborough City Hospital, Peterborough, UK and Hinchingbrooke Hospital, Huntingdon, UK
[201] Colchester General Hospital, Colchester, UK
[202] Princess Royal Hospital, Telford and Royal Shrewsbury Hospital, Shrewsbury, UK
[203] University Hospital Monklands, Airdrie, UK
[204] Wrexham Maelor Hospital, Wrexham, Wales
[205] New Cross Hospital, Wolverhampton, UK
[206] University Hospital Hairmyres, East Kilbride, UK
[207] Warwick Hospital, Warwick, UK
[208] Sandwell General Hospital and City Hospital, Birmingham, UK
[209] Royal Manchester Children's Hospital, Manchester, UK
[210] Gloucestershire Royal Hospital, Gloucester, UK
[211] University Hospitals Coventry & Warwickshire NHS Trust, Coventry, UK
[212] Torbay Hospital, Torquay, UK
[213] Pilgrim Hospital, Lincoln, UK
[214] Prince Philip Hospital, Lianelli, UK
[215] Princess of Wales Hospital, Llantrisant, UK
[216] Northampton General Hospital NHS Trust, Northampton, UK
[217] The Christie NHS Foundation Trust, Manchester, UK
[218] James Paget University Hospital NHS Trust, Great Yarmouth, UK
[219] Birmingham Children's Hospital, Birmingham, UK
[220] Withybush General Hospital, Pembrokeshire, Wales
[221] Northwick Park Hospital, London, UK
[222] North Devon District Hospital, Barnstaple, UK
[223] Scunthorpe General Hospital, Scunthorpe, UK
[224] Royal Free Hospital, London, UK
[225] Raigmore Hospital, Inverness, UK
[226] West Cumberland Hospital, Whitehaven, UK
[227] Furness General Hospital, Barrow-in-Furness, UK
[228] Liverpool Heart and Chest Hospital, Liverpool, UK
[229] Scarborough General Hospital, Scarborough, UK
[230] Bronglais General Hospital, Aberystwyth, UK
[231] Alder Hey Children's Hospital, Liverpool, UK
[232] Borders General Hospital, Melrose, UK
[233] Leighton Hospital, Cheshire, UK
[234] Kent & Canterbury Hospital, Canterbury, UK
[235] Harrogate and District NHS Foundation Trust, Harrogate, UK
[236] The Royal Marsden Hospital, London, UK
[237] Ealing Hospital, Southall, UK
[238] St John's Hospital Livingston, Livingston, UK
[239] Wexham Park Hospital, Slough, UK
[240] Sheffield Children's Hospital, Sheffield, UK
[241] Homerton University Hospital Foundation NHS Trust, London UK
[242] National Hospital for Neurology and Neurosurgery, London, UK
[243] The Royal Alexandra Children's Hospital, Brighton, UK
[244] Golden Jubilee National Hospital, Clydebank, UK

13.2 SCOURGE Consortium

Javier Abellan[304,305], René Acosta-Isaac[306], Jose María Aguado[307,308,309,310], Carlos Aguilar[311], Sergio Aguilera-Albesa[312,313], Abdolah Ahmadi Sabbagh[314], Jorge Alba[315], Sergiu Albu[316,317,318], Karla A.M. Alcalá-Gallardo[319], Julia Alcoba-Florez[320], Sergio Alcolea Batres[321], Holmes Rafael Algarin-Lara[322,323], Virginia Almadana[324], Julia Almeida[325,326,327,328], Berta Almoguera[37,329], María R. Alonso[330], Nuria Alvarez[330], Yady Álvarez-Benítez[322,323], Felipe Álvarez-Navia[331,332], Rodolfo Alvarez-Sala Walther[321], Álvaro Andreu-Bernabeu[309,333], Maria Rosa Antonijoan[334], Eunate Arana-Arri[335,336], Carlos Aranda[337,338],

Celso Arango[309,333,339], Carolina Araque[340,341], Nathalia K. Araujo[342], Izabel M.T. Araujo[343], Ana C. Arcanjo[344,345,346], Ana Arnaiz[33,34,35], Francisco Arnalich Fernández[347], María J. Arranz[348], José Ramon Arribas Lopez[347], Maria-Jesus Artiga[349], Yubelly Avello-Malaver[350], Carmen Ayuso[37,329], Ana Margarita Baldión-Elorza[350], Belén Ballina Martín[314], Raúl C. Baptista-Rosas[351,352,353], Andrea Barranco-Díaz[323], María Barreda- Sánchez[354,355], Viviana Barrera-Penagos[350], Moncef Belhassen-Garcia[332,356], Enrique Bernal[354], David Bernal-Bello[357], Joao F. Bezerra[358], Marcos A.C. Bezerra[359], Natalia Blanca-López[360], Rafael Blancas[361], Lucía Boix-Palop[362], Alberto Borobia[363], Elsa Bravo[364], María Brion[365,366], Óscar Brochado-Kith[310,367], Ramón Brugada[366,368,369,370], Matilde Bustos[371], Alfonso Cabello[372], Juan J. Caceres-Agra[373], Esther Calbo[374], Enrique J. Calderón[375,376,377], Shirley Camacho[378], Marcela C. Campos[344], Yolanda Cañadas[338], Cristina Carbonell[331,332], Servando Cardona-Huerta[36], Antonio Augusto F. Carioca[379], Maria Sanchez Carpintero[337,338], Carlos Carpio Segura[321], Thássia M.T. Carratto[380], José An-tonio Carrillo-Avila[381], Maria C.C. Carvalho[382], Carlos Casasnovas[37,383,384], Luis Castano[37,335,385,386,387], Carlos F. Castaño[337,338], Jose E. Castelao[388], Aranzazu Castellano Candalija[389], María A. Castillo[378], Francisco C. Ceballos[367], Jessica G. Chaux[341], Walter G. Chaves- Santiago[341,390], Sylena Chiquillo-Gómez[322,323], Marco A. Cid-Lopez[319], Oscar Cienfuegos-Jimenez[36], Rosa Conde-Vicente[391], M. Lour-des Cordero-Lorenzana[392], Dolores Corella[393,394], Almudena Corrales[30,31], Jose L. Cortes-Sanchez[36,395], Marta Corton[37,329], Tatiana X. Costa[396], Raquel Cruz[37,44,45,397], Marina S. Cruz[342], Luisa Cuesta[398], Gabriela C.R. Cunha[399], Gabriela V. da Silva[343], David Dalmau[374,400], Raquel C.S. Dantas-Komatsu[342], M. Teresa Darnaude[401], Raimundo de Andrés[402], Jéssica N.G. de Araújo[403], Carmen de Juan[404], Juan De la Cruz Troca[376,405,406], Carmen de la Horra[377], Ana B. de la Hoz[335], Alba De Martino-Rodríguez[407,408], Julianna Lys de Sousa Alves Neri[409], Victor del Campo-Pérez[410], Juan Delgado-Cuesta[411], Covadonga M. Diaz-Caneja[309,333,339], Anderson Díaz-Pérez[323], Aranzazu Diaz de Bustamante[401], Beatriz Dietl[374], Silvia Diz-de Almeida[37,44], Manoella do Monte Alves[412,413], Elena Domínguez-Garrido[414], Katiusse A. dos Santos[382], Alice M. Duarte[343], Jose Echave-Sustaeta[415], Rocío Eiros[416], César O. Enciso-Olivera[340,341], Gabriela Escudero[417], Pedro Pablo España[418], Gladys Mercedes Estigarribia Sanabria[419], María Carmen Fariñas[33,34,35], Marianne R. Fernandes[420,421], Ramón Fernández[33,422], Lidia Fernandez-Caballero[37,329], Ana Fernández-Cruz[423], María J. Fernandez-Nestosa[424], Uxía Fernández-Robelo[425], Amanda Fernández-Rodríguez[310,367], Marta Fernández-Sampedro[33,34,35], Ruth Fernández-Sánchez[37,329], Tania Fernández-Villa[426], Silvia Fernández Ferrero[314], Yolanda Fernández Martínez[314], Carmen Fernéndez Capitán[389], Patricia Flores-Pérez[427], Vicente Friaza[376,377], Lácides Fuenmayor-Hernández[323], Marta Fuertes Núñez[314], Victoria Fumadó[428], Ignacio Gadea[429], Lidia Gagliardi[337,338], Manuela Gago-Domínguez[45,46], Natalia Gallego[38], Cristina Galoppo[430], Inés García[37,329], Mercedes García[337,338], Leticia García[337,338], Carlos Garcia-Cerrada[304,305,431], Aitor García-de-Vicuña[335,385], Josefina Garcia-García[354], Irene García-García[363], Carmen García-Ibarbia[33,34,35], Andrés C. García-Montero[432], Ana García-Soidán[433], Elisa García-Vázquez[354], María Carmen García Torrejón[305,434], Emiliano Garza-Frias[36], Angela Gentile[430], Belén Gil-Fournier[435], Javier Gómez-Arrue[407,408], Mario Gómez-Duque[341,390], Luis Gómez Carrera[321], María Gómez García[397], Ángela Gómez Sacristán[436], Anna González-Neira[330], Javier González-Peñas[309,333,339], Manuel Gonzalez-Sagrado[391], Beatriz González Álvarez[407,408], Fernan Gonzalez Bernaldo de Quirós[437], Hugo Gonzalo Benito[438], Oscar Gorgojo-Galindo[439], Miguel Górgolas[372], Florencia Guaragna[430], Genilson P. Guegel[440], Beatriz Guillen-Guio[30], Encarna Guillen-Navarro[354,441,442,443], Pablo Guisado-Vasco[415], Juan F. Gutiérrez-Bautista[444], Luz D. Gutierrez-Castañeda[341,445], Sarah Heili-Frades[446], Estefania Hernandez[447], Luis D. Hernandez-Ortega[353,448], Guillermo Hernández-Pérez[331], Rebeca Hernández-Vaquero[449], Cristina Hernández Moro[314], Belen Herraez[330], M. Teresa Herranz[354], María Herrera[337,338], María José Herrero[450,451], Antonio Herrero-Gonzalez[452], Juan P. Horcajada[310,317,453,454,455], Natale Imaz-Ayo[335], Maider Intxausti-Urrutibeaskoa[456], María Íñiguez[457], Rafael H. Jacomo[458], Rubén Jara[354], Perez Maria Jazmin[430], Ángel Jiménez[337,338], Pilar Jiménez[444], Ignacio Jiménez-Alfaro[459], María A. Jimenez-Sousa[310,367], Iolanda Jordan[376,460,461], Rocío Laguna-Goya[462,463], Daniel Laorden[321], María Lasa-Lazaro[462,463], María Claudia Lattig[378,464], Ailen Lauriente[430], Anabel Liger Borja[465], Lucía Llanos[466], Amparo López-Bernús[331,332], Esther Lopez-Garcia[376,405,406,467], Rosario Lopez-Rodriguez[37,329], Miguel A. López-Ruz[468,469,470], Eduardo López Granados[37,471,472], Leonardo Lorente[473], José E. Lozano[474], María Lozano-Espinosa[465], Andre D. Luchessi[475], Ignacio Mahillo[31,476,477], Esther Mancebo[462,463], Carmen Mar[418], Cristina Marcelo Calvo[389], Miguel Marcos[331,332], Alba Marcos-Delgado[478], Alicia Marín Candon[363], Pablo Mariscal Aguilar[321], María M. Martín[479], María Dolores Martín[480], Vicente Martín[376,478], Marta Martin-Fernandez[481], Caridad Martín-López[465], José-Ángel Martín-Oterino[331,332], Laura Martin-Pedraza[360], María Martín-Vicente[367], Amalia Martinez[482], Ricardo Martínez[447], Juan José Martínez[37,384], Silvia Martínez[33,35], Eleno Martínez-Aquino[483], Óscar Martínez-González[361], Iciar Martinez-Lopez[484,485], Oscar Martinez-Nieto[350,464], Pedro Martinez-Paz[438], Angel Martinez-Perez[486], Andrea Martínez-Ramas[37,329], Michel F. Martinez-Resendez[36], Violeta Martínez Robles[314], Laura Marzal[37,329], Juliana F. Mazzeu[487,488,489], Jeane F.P. Medeiros[342], Kelliane A. Medeiros[490,491], Francisco J. Medrano[375,376,377], Xose M. Meijome[492,493], Natalia Mejuto-Montero[494], Ana Méndez-Echevarria[347], Humberto Mendoza Charris[323,364], Eleuterio

Merayo Macías[495], Fátima Mercadillo[496], Arieh R. Mercado-Sesma[353,448], Pablo Minguez[37,329], Antonio J J. Molina[376,478], Elena Molina-Roldán[497], Juan José Montoya[447], Vitor M.S. Moraes[380], Patricia Moreira-Escriche[404], Xenia Morelos-Arnedo[323,364], Antonio Moreno-Docón[354], Junior Moreno-Escalante[323], Victor Moreno Cuerda[304,305], Alberto Moreno Fernández[389], Rubén Morilla[377,498], Patricia Muñoz García[31,309,499], Pablo Neira[430], Julian Nevado[37,38,500], Israel Nieto-Gañán[433], Joana F.R. Nunes[344], Rocio Nuñez- Torres[330], Antònia Obrador-Hevia[501,502], J. Gonzalo Ocejo-Vinyals[33,35], Virginia Olivar[430], Silviene F. Oliveira[487,488,489,503,504], Lorena Ondo[37,329], Alberto Orfao[325,326,327,328], Luis Ortega[505], Eva Ortega-Paino[349], Fernando Ortiz-Flores[33,35], Rocio Ortiz-Lopez[36,506], José A. Oteo[315,457], Harry Pachajoa[507,508], Manuel Pacheco[447], Fredy Javier Pacheco-Miranda[323], Irene Padilla Conejo[314], Sonia Panadero-Fajardo[381], Mara Parellada[309,333,339], Roberto Pariente-Rodríguez[433], Estela Paz-Artal[462,463,509], Germán Peces-Barba[31,510], Miguel S. Pedromingo Kus[511], Celia Perales[429], Patricia Perez[512], César Pérez[513], Gustavo Perez-de-Nanclares[335,385], Felipe Pérez-García[514,515], Patricia Pérez-Matute[457], Alexandra Pérez-Serra[366,368], M. Elena Pérez-Tomás[354], Teresa Perucho[516], Lisbeth A. Pichardo[314], Susana M.T. Pinho[490,517,518], Mel · lina Pinsach-Abuin[366,368], Luz Adriana Pinzón[341,390], Guillermo Pita[330], Francesc Pla-Junca[37,519], Laura Planas-Serra[37,384], Ericka N. Pompa-Mera[520], Gloria L. Porras-Hurtado[447], Aurora Pujol[37,384,521], María Eugenia Quevedo Chávez[322,323], Maria Angeles Quijada[334,522], Inés Quintela[397], Diana Ramirez-Montaño[523], Soraya Ramiro León[435], Pedro Rascado Sedes[449], Delia Recalde[407,408], Emma Recio-Fernández[457], Salvador Resino[310,367], Adriana P. Ribeiro[490,491,518], Carlos S. Rivadeneira-Chamorro[341], Diana Roa-Agudelo[350], Montserrat Robelo Pardo[449], Marilyn Johanna Rodriguez[341], Fernando Rodriguez-Artalejo[376,405,406,467], Marena Rodríguez-Ferrer[323], Carlos Rodriguez-Gallego[32,524], José A. Rodriguez-Garcia[314], María A. Rodriguez-Hernandez[371], Antonio Rodriguez-Nicolas[444], Agustí Rodriguez-Palmero[384,525], Emilio Rodríguez-Ruiz[45,449], Paula A. Rodriguez-Urrego[350], Belén Rodríguez Maya[304], German Ezequiel Rodriguez Novoa[430], Federico Rojo[328,526], Andrea Romero-Coronado[323], Filomeno Rondón García[314], Lidia S. Rosa[527], Antonio Rosales-Castillo[528], Cladelis Rubio[529,530], María Rubio Olivera[337,338], Montserrat Ruiz[37,384], Francisco Ruiz-Cabello[444,469,531], Eva Ruiz-Casares[516], Juan J. Ruiz-Cubillan[33,35], Javier Ruiz-Hornillos[338,532,533], Pablo Ryan[534,535,536], Hector D. Salamanca[340,341], Lorena Salazar-García[378], Giorgina Gabriela Salgueiro Origlia[389], Pedro-Luis Sánchez[332,416], Clara Sánchez-Pablo[416], Olga Sánchez-Pernaute[537], Antonio J. Sánchez López[538], María Concepción Sánchez Prados[321], Javier Sánchez Real[314], Jorge Sánchez Redondo[304,539], Cristina Sancho- Sainz[456], Anna Sangil[362], Arnoldo Santos[513], Ney P.C. Santos[420], Agatha Schlüter[37,384], Sonia Segovia[519,540,541], Alex Serra-Llovich[400], Fernando Sevil Puras[311], Marta Sevilla Porras[37,38], Miguel A. Sicolo[542,543], Vivian N. Silbiger[475], Nayara S. Silva[403], Fabiola T.C. Silva[344], Cristina Silván Fuentes[37], Jordi Solé-Violán[31,544,545], José Manuel Soria[486], Jose V. Sorlí[393,394], Renata R. Sousa[487], Juan Carlos Souto[306], Karla S.C. Souza[382], Vanessa S. Souza[399], John J. Sprockel[341,390], José Javier Suárez-Rama[397], David A. Suarez-Zamora[350], Xiana Taboada-Fraga[494], Eduardo Tamayo[439,546], Alvaro Tamayo-Velasco[547], Juan Carlos Taracido-Fernandez[452], Nathali A.C. Tavares[548], Carlos Tellería[407,408], Jair Antonio Tenorio Castaño[37,38,500], Alejandro Teper[430], Juan Torres-Macho[549], Lilian Torres-Tobar[341], Ronald P. Torres Gutiérrez[511], Jesús Troya[534], Miguel Urioste[496], Juan Valencia-Ramos[550], Agustín Valido[324,551], Juan Pablo Vargas Gallo[552,553], Belén Varón[554], Romero H.T. Vasconcelos[548], Tomas Vega[555], Santiago Velasco-Quirce[556], Valentina Vélez-Santamaría[383,384], Virginia Víctor[337,338], Julia Vidán Estévez[314], Miriam Vieitez-Santiago[33,35], Carlos Vilches[557], Lavinia Villalobos[314], Felipe Villar[510], Judit Villar-Garcia[558,559,560], Cristina Villaverde[37,329], Pablo Villoslada-Blanco[457], Ana Virseda-Berdices[367], Zuleima Yáñez[323], Antonio Zapatero-Gaviria[561], Ruth Zarate[562], Sandra Zazo[526], Miguel López de Heredia[37], Ingrid Mendes[37], Rocío Moreno[37], Esther Sande[37,44,45], Carlos Flores[29,30,31,32], José A. Riancho[33,34,35], Augusto Rojas-Martinez[36], Pablo Lapunzina[37,38,500], Angel Carracedo[37,44,45,46,397].

[304] Hospital Universitario Mostoles, Medicina Interna, Madrid, Spain
[305] Universidad Francisco de Vitoria, Madrid, Spain
[306] Haemostasis and Thrombosis Unit, Hospital de la Santa Creu i Sant Pau, IIB Sant Pau, Barcelona, Spain
[307] Unit of Infectious Diseases, Hospital Universitario 12 de Octubre, Instituto de Investigación Sanitaria Hospital 12 de Octubre (imas12), Madrid, Spain
[308] Spanish Network for Research in Infectious Diseases (REIPI RD16/0016/0002), Instituto de Salud Carlos III, Madrid, Spain
[309] School of Medicine, Universidad Complutense, Madrid, Spain
[310] Centro de Investigación Biomédica en Red de Enfermedades Infecciosas (CIBERINFEC), Instituto de Salud Carlos III, Madrid, Spain
[311] Hospital General Santa Bárbara de Soria, Soria, Spain
[312] Pediatric Neurology Unit, Department of Pediatrics, Navarra Health Service Hospital, Pamplona, Spain
[313] Navarra Health Service, NavarraBioMed Research Group, Pamplona, Spain
[314] Complejo Asistencial Universitario de León, León, Spain
[315] Hospital Universitario San Pedro, Infectious Diseases Department, Logroño, Spain
[316] Fundación Institut Guttmann, Institut Universitari de Neurorehabilitació adscrit a la UAB, Hospital de Neurorehabilitació, Barcelona, Spain
[317] Universitat Autònoma de Barcelona (UAB), Barcelona, Spain
[318] Fundació Institut d'Investigació en Ciències de la Salut Germans Trias i Pujol, Barcelona, Spain
[319] Hospital General de Occidente, Guadalajara, Mexico
[320] Microbiology Unit, Hospital Universitario N. S. de Candelaria, Santa Cruz de Tenerife, Spain
[321] Hospital Universitario La Paz-IDIPAZ, Servicio de Neumología, Madrid, Spain
[322] Camino Universitario Adelita de Char, Mired IPS, Barranquilla, Colombia

[323] Universidad Simón Bolívar, Facultad de Ciencias de la Salud, Barranquilla, Colombia
[324] Hospital Universitario Virgen Macarena, Neumología, Seville, Spain
[325] Departamento de Medicina, Universidad de Salamanca, Salamanca, Spain
[326] Centro de Investigación del Cáncer (IBMCC) Universidad de Salamanca - CSIC, Salamanca, Spain
[327] Biomedical Research Institute of Salamanca (IBSAL) Salamanca, Spain
[328] Centre for Biomedical Network Research on Cancer (CIBERONC), Instituto de Salud Carlos III, Madrid, Spain
[329] Department of Genetics & Genomics, Instituto de Investigación Sanitaria-Fundación Jiménez Díaz University Hospital - Universidad Autónoma de Madrid (IIS-FJD, UAM), Madrid, Spain
[330] Spanish National Cancer Research Centre, Human Genotyping-CEGEN Unit, Madrid, Spain
[331] Hospital Universitario de Salamanca-IBSAL, Servicio de Medicina Interna, Salamanca, Spain
[332] Universidad de Salamanca, Salamanca, Spain
[333] Department of Child and Adolescent Psychiatry, Institute of Psychiatry and Mental Health, Hospital General Universitario Gregorio Marañón (IiSGM), Madrid, Spain
[334] Clinical Pharmacology Service, Hospital de la Santa Creu i Sant Pau, IIB Sant Pau, Barcelona, Spain
[335] Biocruces Bizkai HRI, Barakaldo, Bizkaia, Spain
[336] Cruces University Hospital, Osakidetza, Barakaldo, Bizkaia, Spain
[337] Hospital Infanta Elena, Valdemoro, Madrid, Spain
[338] Instituto de Investigación Sanitaria-Fundación Jiménez Díaz University Hospital - Universidad Autónoma de Madrid (IIS-FJD, UAM), Madrid, Spain
[339] Centre for Biomedical Network Research on Mental Health (CIBERSAM), Instituto de Salud Carlos III, Madrid, Spain
[340] Fundación Hospital Infantil Universitario de San José, Bogotá, Colombia
[341] Fundación Universitaria de Ciencias de la Salud, Bogotá, Colombia
[342] Universidade Federal do Rio Grande do Norte, Programa de Pós-graduação em Ciências da Saúde, Natal, Brazil
[343] Universidade Federal do Rio Grande do Norte, Departamento de Medicina Clínica, Natal, Brazil
[344] Departamento de Genética e Morfologia, Instituto de Ciências Biológicas, Universidade de Brasília, Brasilia, Brazil
[345] Colégio Marista de Brasilia, Brazil
[346] Associação Brasileira de Educação e Cultura, Brazil
[347] Hospital Universitario La Paz-IDIPAZ, Servicio de Medicina Interna, Madrid, Spain
[348] Fundació Docència I Recerca Mutua Terrassa, Barcelona, Spain
[349] Spanish National Cancer Research Center, CNIO Biobank, Madrid, Spain
[350] Fundación Santa Fe de Bogota, Departamento Patologia y Laboratorios, Bogotá, Colombia
[351] Hospital General de Occidente, Zapopan, Jalisco, Mexico
[352] Centro Universitario de Tonalá, Universidad de Guadalajara, Tonalá, Jalisco, Mexico
[353] Centro de Investigación Multidisciplinario en Salud, Universidad de Guadalajara, Guadalajara, Mexico
[354] Instituto Murciano de Investigación Biosanitaria (IMIB-Arrixaca), Murcia, Spain
[355] Universidad Católica San Antonio de Murcia (UCAM), Murcia, Spain
[356] Hospital Universitario de Salamanca-IBSAL, Servicio de Medicina Interna-Unidad de Enfermedades Infecciosas, Salamanca, Spain
[357] Hospital Universitario de Fuenlabrada, Department of Internal Medicine, Madrid, Spain
[358] Escola Tecnica de Saúde, Laboratorio de Vigilancia Molecular Aplicada, Pará, Brazil
[359] Federal University of Pernambuco, Genetics Postgraduate Program, Recife, PE, Brazil
[360] Hospital Universitario Infanta Leonor, Servicio de Alergia, Madrid, Spain
[361] Hospital Universitario del Tajo, Servicio de Medicina Intensiva, Toledo, Spain
[362] Hospital Universitario Mutua Terrassa, Barcelona, Spain
[363] Hospital Universitario La Paz-IDIPAZ, Servicio de Farmacología, Madrid, Spain
[364] Alcaldía de Barranquilla, Secretaría de Salud, Barranquilla, Colombia
[365] Instituto de Investigación Sanitaria de Santiago (IDIS), Xenética Cardiovascular, Santiago de Compostela, Spain
[366] Centre for Biomedical Network Research on Cardiovascular Diseases (CIBERCV), Instituto de Salud Carlos III, Madrid, Spain
[367] Unidad de Infección Viral e Inmunidad, Centro Nacional de Microbiología (CNM), Instituto de Salud Carlos III (ISCIII), Madrid, Spain
[368] Cardiovascular Genetics Center, Institut d'Investigació Biomèdica Girona (IDIBGI), Girona, Spain
[369] Medical Science Department, School of Medicine, University of Girona, Girona, Spain
[370] Hospital Josep Trueta, Cardiology Service, Girona, Spain
[371] Institute of Biomedicine of Seville (IBiS), Consejo Superior de Investigaciones Científicas (CSIC)- University of Seville- Virgen del Rocio University Hospital, Seville, Spain
[372] Division of Infectious Diseases, Instituto de Investigación Sanitaria-Fundación Jiménez Díaz University Hospital - Universidad Autónoma de Madrid (IIS-FJD, UAM), Madrid, Spain
[373] Intensive Care Unit, Hospital Universitario Insular de Gran Canaria, Las Palmas de Gran Canaria, Spain
[374] Hospital Universitario Mutua Terrassa, Terrassa, Spain
[375] Departemento de Medicina, Hospital Universitario Virgen del Rocío,Universidad de Sevilla, Seville, Spain
[376] Centre for Biomedical Network Research on Epidemiology and Public Health (CIBERESP), Instituto de Salud Carlos III, Madrid, Spain
[377] Instituto de Biomedicina de Sevilla, Seville, Spain
[378] Universidad de los Andes, Facultad de Ciencias, Bogotá, Colombia
[379] University of Fortaleza (UNIFOR), Department of Nutrition. Fortaleza, Brazil
[380] Departamento de Química, Faculdade de Filosofia, Ciências e Letras de Ribeirão Preto, Universidade de São Paulo, Brazil
[381] Andalusian Public Health System Biobank, Granada, Spain
[382] Universidade Federal do Rio Grande do Norte, Programa de Pós-Graduação em Ciências Farmacêuticas, Natal, Brazil
[383] Neuromuscular Unit, Neurology Department, Hospital Universitari de Bellvitge, L'Hospitalet de Llobregat (Barcelona), Spain
[384] Bellvitge Biomedical Research Institute (IDIBELL), Neurometabolic Diseases Laboratory, L'Hospitalet de Llobregat, Spain
[385] Osakidetza, Cruces University Hospital, Barakaldo, Bizkaia, Spain
[386] Centre for Biomedical Network Research on Diabetes and Metabolic Associated Diseases (CIBERDEM), Instituto de Salud Carlos III, Madrid, Spain
[387] University of Pais Vasco, UPV/EHU, Bizkaia, Spain
[388] Oncology and Genetics Unit, Instituto de Investigacion Sanitaria Galicia Sur, Xerencia de Xestion Integrada de Vigo-Servizo Galego de Saúde, Vigo, Spain
[389] Hospital Universitario La Paz, Hospital Carlos III, Madrid, Spain
[390] Hospital de San José, Sociedad de Cirugía de Bogota, Bogotá, Colombia
[391] Hospital Universitario Río Hortega, Valladolid, Spain
[392] Servicio de Medicina intensiva, Complejo Hospitalario Universitario de A Coruña (CHUAC), Sistema Galego de Saúde (SERGAS), A Coruña, Spain
[393] Valencia University, Preventive Medicine Department, Valencia, Spain
[394] Centre for Biomedical Network Research on Physiopatology of Obesity and Nutrition (CIBEROBN), Instituto de Salud Carlos III, Madrid, Spain
[395] Otto von Guericke University, Departament of Microgravity and Translational Regenerative Medicine, Magdeburg, Germany
[396] Maternidade Escola Janário Cicco, Natal, Brazil
[397] Centro Nacional de Genotipado (CEGEN), Universidade de Santiago de Compostela, Santiago de Compostela, Spain
[398] Institute of Psychiatry and Mental Health, Hospital General Universitario Gregorio Marañón (IiSGM), Madrid, Spain
[399] Programa de Pós Graduação em Ciências da Saúde, Faculdade de Medicina, Universidade de Brasília, Brasilia, Brazil
[400] Fundació Docència I Recerca Mutua Terrassa, Terrassa, Spain
[401] Hospital Universitario Mostoles, Unidad de Genética, Madrid, Spain
[402] Internal Medicine Department, Instituto de Investigación Sanitaria-Fundación Jiménez Díaz University Hospital - Universidad

Autónoma de Madrid (IIS-FJD, UAM), Madrid, Spain
[403] Universidade Federal do Rio Grande do Norte, Pós-graduação em Biotecnologia - Rede de Biotecnologia do Nordeste (Renorbio), Natal, Brazil
[404] Hospital Universitario Severo Ochoa, Servicio de Medicina Interna, Madrid, Spain
[405] Department of Preventive Medicine and Public Health, School of Medicine, Universidad Autónoma de Madrid, Madrid, Spain
[406] IdiPaz (Instituto de Investigación Sanitaria Hospital Universitario La Paz), Madrid, Spain
[407] Instituto Aragonés de Ciencias de la Salud (IACS), Zaragoza, Spain
[408] Instituto Investigación Sanitaria Aragón (IIS-Aragon), Zaragoza, Spain
[409] Universidade Federal do Rio Grande do Norte, Programa de Pós Graduação em Nutrição, Natal, Brazil
[410] Preventive Medicine Department, Instituto de Investigacion Sanitaria Galicia Sur, Xerencia de Xestion Integrada de Vigo-Servizo Galego de Saúde, Vigo, Spain
[411] Hospital Universitario Virgen del Rocío, Servicio de Medicina Interna, Seville, Spain
[412] Universidade Federal do Rio Grande do Norte, Departamento de Infectologia, Natal, Brazil
[413] Hospital de Doenças Infecciosas Giselda Trigueiro, Rio Grande do Norte, Natal, Brazil
[414] Unidad Diagnóstico Molecular. Fundación Rioja Salud, La Rioja, Spain
[415] Hospital Universitario Quironsalud Madrid, Madrid, Spain
[416] Hospital Universitario de Salamanca-IBSAL, Servicio de Cardiología, Salamanca, Spain
[417] Hospital Universitario Puerta de Hierro, Servicio de Medicina Interna, Majadahonda, Spain
[418] Biocruces Bizkaia Health Research Institute, Galdakao University Hospital, Osakidetza, Bizkaia, Spain
[419] Instituto Regional de Investigación en Salud-Universidad Nacional de Caaguazú, Caaguazú, Paraguay
[420] Universidade Federal do Pará, Núcleo de Pesquisas em Oncologia, Belém, Pará, Brazil
[421] Hospital Ophir Loyola, Departamento de Ensino e Pesquisa, Belém, Pará, Brazil
[422] Fundación Asilo San Jose, Santander, Spain
[423] Unidad de Enfermedades Infecciosas, Servicio de Medicina Interna, Hospital Universitario Puerta de Hierro, Instituto de Investigación Sanitaria Puerta de Hierro - Segovia de Arana, Madrid, Spain
[424] Universidad Nacional de Asunción, Facultad de Politécnica, Paraguay
[425] Urgencias Hospitalarias, Complejo Hospitalario Universitario de A Coruña (CHUAC), Sistema Galego de Saúde (SERGAS), A Coruña, Spain
[426] Grupo de Investigación en Interacciones Gen-Ambiente y Salud (GIIGAS) - Instituto de Biomedicina (IBIOMED), Universidad de León, León, Spain
[427] Hospital Universitario Niño Jesús, Pediatrics Department, Madrid, Spain
[428] Unitat de Malalties Infeccioses i Importades, Servei de Pediatría, Infectious and Imported Diseases, Pediatric Unit, Hospital Universitari Sant Joan de Deú, Barcelona, Spain
[429] Microbiology Department, Instituto de Investigación Sanitaria-Fundación Jiménez Díaz University Hospital - Universidad Autónoma de Madrid (IIS-FJD, UAM), Madrid, Spain
[430] Hospital de Niños Ricardo Gutierrez, Buenos Aires, Argentina
[431] Centre for Biomedical Network Research on Rare Diseases (CIBERER), Instituto de Salud Carlos III, Madrid, Spain

Universidad Francisco de Vitoria, Madrid,Spain
[432] University of Salamanca, Biomedical Research Institute of Salamanca (IBSAL), Salamanca, Spain
[433] Department of Immunology, IRYCIS, Hospital Universitario Ramón y Cajal, Madrid, Spain
[434] Hospital Infanta Elena, Servicio de Medicina Intensiva, Valdemoro, Madrid, Spain
[435] Hospital Universitario de Getafe, Servicio de Genética, Madrid, Spain
[436] Pneumology Department, Hospital General Universitario Gregorio Marañón (iiSGM), Madrid, Spain
[437] Ministerio de Salud Ciudad de Buenos Aires, Buenos Aires, Argentina
[438] Hospital Clinico Universitario de Valladolid, Unidad de Apoyo a la Investigación, Valladolid, Spain
[439] Universidad de Valladolid, Departamento de Cirugía, Valladolid, Spain
[440] Secretaria Municipal de Saude de Apodi, Natal, Brazil
[441] Sección Genética Médica - Servicio de Pediatría, Hospital Clínico Universitario Virgen de la Arrixaca, Servicio Murciano de Salud, Murcia, Spain
[442] Departamento Cirugía, Pediatría, Obstetricia y Ginecología, Facultad de Medicina, Universidad de Murcia (UMU), Murcia, Spain
[443] Grupo Clínico Vinculado, Centre for Biomedical Network Research on Rare Diseases (CIBERER), Instituto de Salud Carlos III, Madrid, Spain
[444] Hospital Universitario Virgen de las Nieves, Servicio de Análisis Clínicos e Inmunología, Granada, Spain
[445] Hospital Universitario Centro Dermatológico Federico Lleras Acosta, Bogotá, Colombia
[446] Intermediate Respiratory Care Unit, Department of Pneumology, Instituto de Investigación Sanitaria-Fundación Jiménez Díaz University Hospital - Universidad Autónoma de Madrid (IIS-FJD, UAM), Madrid, Spain
[447] Clinica Comfamiliar Risaralda, Pereira, Colombia
[448] Centro Universitario de Tonalá, Universidad de Guadalajara, Guadalajara, Mexico
[449] Unidad de Cuidados Intensivos, Hospital Clínico Universitario de Santiago (CHUS), Sistema Galego de Saúde (SERGAS), Santiago de Compostela, Spain
[450] IIS La Fe, Plataforma de Farmacogenética, Valencia, Spain
[451] Universidad de Valencia, Departamento de Farmacología, Valencia, Spain
[452] Data Analysis Department, Instituto de Investigación Sanitaria-Fundación Jiménez Díaz University Hospital - Universidad Autónoma de Madrid (IIS-FJD, UAM), Madrid, Spain
[453] Hospital del Mar, Infectious Diseases Service, Barcelona, Spain
[454] Institut Hospital del Mar d'Investigacions Mèdiques (IMIM), Barcelona, Spain
[455] CEXS-Universitat Pompeu Fabra, Spanish Network for Research in Infectious Diseases (REIPI), Barcelona, Spain
[456] Biocruces Bizkaia Health Research Institute, Basurto University Hospital, Osakidetza, Bizkaia, Spain
[457] Infectious Diseases, Microbiota and Metabolism Unit, Center for Biomedical Research of La Rioja (CIBIR), Logroño, Spain
[458] Sabin Medicina Diagnóstica, Brazil
[459] Opthalmology Department, Instituto de Investigación Sanitaria-Fundación Jiménez Díaz University Hospital - Universidad Autónoma de Madrid (IIS-FJD, UAM), Madrid, Spain
[460] Hospital Sant Joan de Deu,Pediatric Critical Care Unit, Barcelona, Spain
[461] Paediatric Intensive Care Unit, Agrupación Hospitalaria Clínic-Sant Joan de Déu, Esplugues de Llobregat, Barcelona, Spain
[462] Hospital Universitario 12 de Octubre, Department of Immunology, Madrid, Spain
[463] Instituto de Investigación Sanitaria Hospital 12 de Octubre (imas12), Transplant Immunology and Immunodeficiencies Group, Madrid, Spain
[464] SIGEN Alianza Universidad de los Andes - Fundación Santa Fe de Bogotá, Bogotá, Colombia
[465] Hospital General de Segovia, Medicina Intensiva, Segovia, Spain
[466] Clinical Trials Unit, Instituto de Investigación Sanitaria-Fundación Jiménez Díaz University Hospital - Universidad Autónoma de Madrid (IIS-FJD, UAM), Madrid, Spain
[467] IMDEA-Food Institute, CEI UAM+CSIC, Madrid, Spain
[468] Hospital Universitario Virgen de las Nieves, Servicio de Enfermedades Infecciosas, Granada, Spain
[469] Instituto de Investigación Biosanitaria de Granada (ibs GRANADA), Granada, Spain
[470] Universidad de Granada, Departamento de Medicina, Granada, Spain
[471] Hospital Universitario La Paz-IDIPAZ, Servicio de Inmunología, Madrid, Spain
[472] La Paz Institute for Health Research (IdiPAZ), Lymphocyte Pathophysiology in Immunodeficiencies Group, Madrid, Spain
[473] Intensive Care Unit, Hospital Universitario de Canarias, La Laguna, Spain
[474] Dirección General de Salud Pública, Consejería de Sanidad, Junta de Castilla y León, Valladolid, Spain
[475] Universidade Federal do Rio Grande do Norte, Departamento de Analises Clinicas e Toxicologicas, Natal, Brazil

[476] Fundación Jiménez Díaz, Epidemiology, Madrid, Spain
[477] Universidad Autónoma de Madrid, Department of Medicine, Madrid, Spain
[478] Instituto de Biomedicina (IBIOMED), Universidad de León, León, Spain
[479] Intensive Care Unit, Hospital Universitario N. S. de Candelaria, Santa Cruz de Tenerife, Spain
[480] Preventive Medicine Department, Instituto de Investigación Sanitaria-Fundación Jiménez Díaz University Hospital - Universidad Autónoma de Madrid (IIS-FJD, UAM), Madrid, Spain
[481] Universidad de Valladolid, Departamento de Medicina, Valladolid, Spain
[482] Hospital Universitario Infanta Leonor, Servicio de Medicina Intensiva, Madrid, Spain
[483] Servicio de Medicina Interna, Sanatorio Franchin, Buenos Aires, Argentina
[484] Unidad de Genética y Genómica Islas Baleares, Islas Baleares, Spain
[485] Hospital Universitario Son Espases, Unidad de Diagnóstico Molecular y Genética Clínica, Islas Baleares, Spain
[486] Genomics of Complex Diseases Unit, Research Institute of Hospital de la Santa Creu i Sant Pau, IIB Sant Pau, Barcelona, Spain
[487] Faculdade de Medicina, Universidade de Brasília, Brasilia, Brazil
[488] Programa de Pós-Graduação em Ciências Médicas, Universidade de Brasília, Brasilia, Brazil
[489] Programa de Pós-Graduação em Ciências da Saúde, Universidade de Brasília, Brasilia, Brazil
[490] Hospital das Forças Armadas, Brazil
[491] Exército Brasileiro, Brazil
[492] Hospital El Bierzo, Gerencia de Asistencia Sanitaria del Bierzo (GASBI), Gerencia Regional de Salud (SACYL), Ponferrada, Spain
[493] Grupo INVESTEN, Instituto de Salud Carlos III, Madrid, Spain
[494] Unidad de Cuidados Intensivos, Complejo Universitario de A Coruña (CHUAC), Sistema Galego de Saúde (SERGAS), A Coruña, Spain
[495] Hospital El Bierzo, Unidad Cuidados Intensivos, León, Spain
[496] Spanish National Cancer Research Centre, Familial Cancer Clinical Unit, Madrid, Spain
[497] Instituto de Investigación Sanitaria San Carlos (IdISSC), Hospital Clínico San Carlos (HCSC), Madrid, Spain
[498] Universidad de Sevilla, Departamento de Enfermería, Seville, Spain
[499] Hospital General Universitario Gregorio Marañón (IiSGM), Madrid, Spain
[500] ERN-ITHACA-European Reference Network
[501] Unidad de Genética y Genómica Islas Baleares, Unidad de Diagnóstico Molecular y Genética Clínica, Hospital Universitario Son Espases, Islas Baleares, Spain
[502] Instituto de Investigación Sanitaria Islas Baleares (IdISBa), Islas Baleares, Spain
[503] Programa de Pós-Graduação em Biologia Animal, Universidade de Brasília, Brasília, Brazil
[504] Programa de Pós-Graduação Profissional em Ensino de Biologia, Universidade de Brasília, Brasília, Brazil
[505] Anatomía Patológica, Instituto de Investigación Sanitaria San Carlos (IdISSC), Hospital Clínico San Carlos (HCSC), Madrid, Spain
[506] Tecnológico de Monterrey, Monterrey, Mexico
[507] Centro de Investigación en Anomalías Congénitas y Enfermedades Raras (CIACER), Universidad Icesi
[508] Departamento de Genetica, Fundación Valle del Lili
[509] Universidad Complutense de Madrid, Department of Immunology, Ophthalmology and ENT, Madrid, Spain
[510] Department of Neumology, Instituto de Investigación Sanitaria-Fundación Jiménez Díaz University Hospital - Universidad Autónoma de Madrid (IIS-FJD, UAM), Madrid, Spain
[511] Hospital Nuestra Señora de Sonsoles, Ávila, Spain
[512] Inditex, A Coruña, Spain
[513] Intensive Care Department, Instituto de Investigación Sanitaria-Fundación Jiménez Díaz University Hospital - Universidad Autónoma de Madrid (IIS-FJD, UAM), Madrid, Spain
[514] Hospital Universitario Príncipe de Asturias, Servicio de Microbiología Clínica, Madrid, Spain
[515] Universidad de Alcalá de Henares, Departamento de Biomedicina y Biotecnología, Facultad de Medicina y Ciencias de la Salud, Madrid, Spain
[516] GENYCA, Madrid, Spain
[517] Marinha do Brasil, Brazil
[518] Universidade de Brasília, Brasilia, Brazil
[519] Neuromuscular Diseases Unit, Department of Neurology, Hospital de la Santa Creu i Sant Pau, Universitat Autònoma de Barcelona, Barcelona, Spain
[520] Instituto Mexicano del Seguro Social (IMSS), Centro Médico Nacional Siglo XXI, Unidad de Investigación Médica en Enfermedades Infecciosas y Parasitarias, Mexico City, Mexico
[521] Catalan Institution of Research and Advanced Studies (ICREA), Barcelona, Spain
[522] Drug Research Centre, Institut d'Investigació Biomèdica Sant Pau, IIB-Sant Pau, Barcelona, Spain
[523] Departamento de Genetica, Clinica imbanaco
[524] Department of Immunology, Hospital Universitario de Gran Canaria Dr. Negrín, Las Palmas de Gran Canaria, Spain
[525] University Hospital Germans Trias i Pujol, Pediatrics Department, Badalona, Spain
[526] Department of Pathology, Biobank, Instituto de Investigación Sanitaria-Fundación Jiménez Díaz University Hospital - Universidad Autónoma de Madrid (IIS-FJD, UAM), Madrid, Spain
[527] Faculdade de Ciências da Saúde, Universidade de Brasília, Brasilia, Brazil
[528] Hospital Universitario Virgen de las Nieves, Servicio de Medicina Interna, Granada, Spain
[529] Fundación Universitaria de Ciencias de la Salud, Grupo de Ciencias Básicas en Salud (CBS), Bogotá, Colombia
[530] Sociedad de Cirugía de Bogotá, Hospital de San José, Bogotá, Colombia
[531] Universidad de Granada, Departamento Bioquímica, Biología Molecular e Inmunología III, Granada, Spain
[532] Hospital Infanta Elena, Allergy Unit, Valdemoro, Madrid, Spain
[533] Faculty of Medicine, Universidad Francisco de Vitoria, Madrid, Spain
[534] Hospital Universitario Infanta Leonor, Madrid, Spain
[535] Complutense University of Madrid, Madrid, Spain
[536] Gregorio Marañón Health Research Institute (IiSGM), Madrid, Spain
[537] Reumathology Service, Instituto de Investigación Sanitaria-Fundación Jiménez Díaz University Hospital - Universidad Autónoma de Madrid (IIS-FJD, UAM), Madrid, Spain
[538] Biobank, Puerta de Hierro-Segovia de Arana Health Research Institute, Madrid, Spain
[539] Universidad Rey Juan Carlos, Madrid, Spain
[540] The John Walton Muscular Dystrophy Research Centre, Newcastle University and Newcastle Hospitals NHS Foundation Trust, Newcastle upon Tyne, UK
[541] Neuromuscular Unit, Neuropediatrics Department, Institut de Recerca Sant Joan de Déu, Hospital Sant Joan de Déu, Spain
[542] Casa de Saúde São Lucas, Natal, Brazil
[543] Hospital Rio Grande, Rio Grande do Norte, Natal, Brazil
[544] Intensive Care Unit, Hospital Universitario de Gran Canaria Dr. Negrín, Las Palmas de Gran Canaria, Spain
[545] Universidad Fernando Pessoa Canarias, Las Palmas de Gran Canaria, Spain
[546] Hospital Clinico Universitario de Valladolid, Servicio de Anestesiologia y Reanimación, Valladolid, Spain
[547] Hospital Clinico Universitario de Valladolid, Servicio de Hematologia y Hemoterapia, Valladolid, Spain
[548] Hospital Universitario Lauro Wanderley, Brazil
[549] Hospital Universitario Infanta Leonor, Servicio de Medicina Interna, Madrid, Spain
[550] University Hospital of Burgos, Burgos, Spain
[551] Universidad de Sevilla, Seville, Spain
[552] Fundación Santa Fe de Bogota, Instituto de servicios medicos de Emergencia y trauma, Bogotá, Colombia
[553] Universidad de los Andes, Bogotá, Colombia

[554] Quironprevención, A Coruña, Spain
[555] Junta de Castilla y León, Consejería de Sanidad, Valladolid, Spain
[556] Gerencia Atención Primaria de Burgos, Burgos, Spain
[557] Immunogenetics-Histocompatibility group, Servicio de Inmunología, Instituto de Investigación Sanitaria Puerta de Hierro - Segovia de Arana, Madrid, Spain
[558] Hospital del Mar, Department of Infectious Diseases, Barcelona, Spain
[559] IMIM (Hospital del Mar Medical Research Institute, Institut Hospital del Mar d'Investigacions Mediques), Barcelona, Spain
[560] Universitat Autònoma de Barcelona, Department of Medicine, Spain
[561] Consejería de Sanidad, Comunidad de Madrid, Madrid, Spain
[562] Centro para el Desarrollo de la Investigación Científica, Asunción, Paraguay

13.3 23andMe Investigators

Janie F. Shelton*[1]*, *Anjali J. Shastri*[1], Chelsea Ye[1], Catherine H. Weldon[1], Teresa Filshtein-Sonmez[1], Daniella Coker[1], Antony Symons[1], Jorge Esparza-Gordillo[2], Stella Aslibekyan[1], Adam Auton[1]

*These authors contributed equally to this work. 1. 23andMe Inc., 223 N Mathilda Ave, Sunnyvale, CA 94086 2. Human genetics - R&D, GSK Medicines Research Centre, Target Sciences-R&D, Stevenage, UK

13.3.1 23andMe Contributors

We thank the 23andMe research participants who made this study possible. We would also like to thank Altovise Ewing, Aaron Petrakovitz, Anne Park, Anne Silk, Aushawna Collins, Becky Macintosh, Carolyn Kao, Courtney Ball, Christine Pai, David Hinds, Devyn Parry, Elo Ratcliff, Emily Bullis, Eric Hall, Farwa Alam, Jacquie Haggarty, Jess Christenson, Jim Lawrence, Jimmy Chau, Josie Shaw, Joe Cackler, Karl Heilbron, Katelyn Kukar, Katie Watson, Marianna Frendo, Olivia Valenti, Ryan Workman, Rachel Lopatin, Robert Bell, Rose Eckert, Sam Rodgers, Sarah Rys, Shawna Averbeck, Shirin Fuller, Vanessa Lane, and Yunxuan Jiang for contributions and insights.

We also thank the 23andMe Research Team: Barry Hicks, Chao Tian, Devika Dhamija, Elizabeth Babalola, Elizabeth S. Noblin, Ethan M. Jewett, G. David Poznik, Gabriel Cuellar Partida, Jared O'Connell, Jingchunzi Shi, Joanna L. Mountain, Joyce Y. Tung, Katarzyna Bryc, Karen E. Huber, Keng-Han Lin, Kimberly F. McManus, Kipper Fletez-Brant, Marie K. Luff, Matthew H. McIntyre, Maya Lowe, Meghan E. Moreno, Peter Wilton, Pierre Fontanillas, Priyanka Nandakumar, Sahar V. Mozaffari, Sarah L. Elson, Sayantan Das, Steven J. Micheletti, Suyash Shringarpure, Vinh Tran, Wei Wang, Will Freyman, and Xin Wang.

Members of the 23andMe COVID-19 Team are: Adam Auton, Adrian Chubb, Alison Fitch, Alison Kung, Amanda Altman, Andy Kill, Anjali Shastri, Catherine Weldon, Chelsea Ye, Daniella Coker, Janie Shelton, Jason Tan, Jeff Pollard, Jennifer McCreight, Jess Bielenberg, John Matthews, Johnny Lee, Lindsey Tran, Michelle Agee, Monica Royce, Nate Tang, Pooja Gandhi, Raffaello d'Amore, Ruth Tennen, Scott Dvorak, Scott Hadly, Stella Aslibekyan, Sungmin Park, Taylor Morrow, Teresa Filshtein Sonmez, Trung Le, and Yiwen Zheng.

13.4 ISARIC4C Investigators

13.4.1 Co-Investigators

J Kenneth Baillie[1,2,3,11], Peter JM Openshaw[12,26], Malcolm G Semple[16,17], Beatrice Alex[245], Petros Andrikopoulos[246,247], Benjamin Bach[245], Wendy S Barclay[248], Debby Bogaert[18], Meera Chand[249], Kanta Chechi[246,250], Graham S Cooke[251], Ana da Silva Filipe[252], Thushan de Silva[253], Annemarie B Docherty[11,254], Gonçalo dos Santos Correia[255,256], Marc-Emmanuel Dumas[246,247,257,258], Jake Dunning[12,259], Tom Fletcher[260], Christopher A Green[261], William Greenhalf[262], Julian Griffin[246], Rishi K Gupta[263], Ewen M Harrison[254], Antonia YW Ho[252,264], Karl Holden[265], Peter W Horby[21], Samreen Ijaz[266], Say Khoo[267], Paul Klenerman[268,269], Andrew Law[3], Matthew Lewis[255,256], Sonia Liggi[246], Wei Shen Lim[270], Lynn Maslen[255,256], Alexander J Mentzer[271,272], Laura Merson[273], Alison M Meynert[2], Shona C Moore[274], Mahdad Noursadeghi[275], Michael Olanipekun[246,247], Anthonia Osagie[246,247], Massimo Palmarini[252], Carlo Palmieri[276,277], William A Paxton[274,278], Georgios Pollakis[274,278], Nicholas Price[279,280], Andrew Rambaut[281], David L Robertson[252], Clark D Russell[18], Vanessa Sancho-Shimizu[282], Caroline Sands[255,256], Janet T Scott[252,283], Louise Sigfrid[273], Tom Solomon[16,284], Shiranee Sriskandan[251,285], David Stuart[8], Charlotte Summers[19], Olivia V Swann[286], Zoltan Takats[246,287], Panteleimon Takis[255,256], Richard S Tedder[288,289,290], AA Roger Thompson[291], Emma C Thomson[252], Ryan S Thwaites[12], Lance CW Turtle[16,292], Maria Zambon[259], Gail Carson[273].

13.4.2 Data analysis team

Thomas M Drake[254], Cameron J Fairfield[254], Stephen R Knight[254], Kenneth A Mclean[254], Derek Murphy[254], Lisa Norman[254], Riinu Pius[254], Catherine A Shaw[254].

13.4.3 Data architecture team

Marie Connor[293], Jo Dalton[293], Carrol Gamble[293], Michelle Girvan[293], Sophie Halpin[293], Janet Harrison[293], Clare Jackson[293], Laura Marsh[293], Stephanie Roberts[293], Egle Saviciute[293], Sara Clohisey[3], Ross Hendry[3], Susan Knight[295], Eva Lahnsteiner[295], Andrew Law[3], Gary Leeming[296], Lucy Norris[297], James Scott-Brown[245], Sarah Tait[295], Murray Wham[2].

13.4.4 Data analysis and management team

James Lee[294], Daniel Plotkin[294], Seán Keating[11].

13.4.5 Project administration team

Cara Donegan[301], Rebecca G Spencer[301].

13.4.6 Project management team

Chloe Donohue[293], Fiona Griffiths[3], Hayley Hardwick[16], Wilna Oosthuyzen[3].

[245] School of Informatics, University of Edinburgh, Edinburgh, UK
[246] Section of Biomolecular Medicine, Division of Systems Medicine, Department of Metabolism, Digestion and Reproduction, Sir Alexander Fleming Building, Exhibition Rd, London SW7 2AZ, UK
[247] Section of Genomic and Environmental Medicine, Respiratory Division, National Heart and Lung Institute, Guy Scadding building, Dovehouse St, London SW3 3LY, UK
[248] Section of Molecular Virology, Imperial College London, London, UK
[249] Antimicrobial Resistance and Hospital Acquired Infection Department, Public Health England, London, UK
[250] Department of Epidemiology and Biostatistics, School of Public Health, Faculty of Medicine, Imperial College London, 2 Norfolk St, W2 1PG, UK
[251] Department of Infectious Disease, Imperial College London, London, UK
[252] MRC-University of Glasgow Centre for Virus Research, 464 Bearsden Road, Glasgow, UK
[253] The Florey Institute for Host-Pathogen Interactions, Department of Infection, Immunity and Cardiovascular Disease, University of Sheffield, Sheffield, UK
[254] Centre for Medical Informatics, The Usher Institute, University of Edinburgh, Edinburgh, UK
[255] National Phenome Centre, Department of Metabolism, Digestion and Reproduction, Imperial College London, London W12 0NN, United Kingdom
[256] Section of Bioanalytical Chemistry, Department of Metabolism, Digestion and Reproduction, Imperial College London, London SW7 2AZ, United Kingdom
[257] European Genomic Institute for Diabetes, CNRS UMR 8199, INSERM UMR 1283, Institut Pasteur de Lille, Lille University Hospital, University of Lille, 59045 Lille, France
[258] McGill University and Genome Quebec Innovation Centre, 740 Doctor Penfield Avenue, Montréal, QC, H3A 0G1, Canada
[259] National Infection Service, Public Health England, London, UK
[260] Liverpool School of Tropical Medicine, Liverpool, UK
[261] Institute of Microbiology and Infection, University of Birmingham, Birmingham, UK
[262] Department of Molecular and Clinical Cancer Medicine, University of Liverpool, Liverpool, UK
[263] Institute for Global Health, University College London, London, UK
[264] Department of Infectious Diseases, Queen Elizabeth University Hospital, Glasgow, UK
[265] University of Liverpool
[266] Virology Reference Department, National Infection Service, Public Health England, Colindale Avenue, London, United Kingdom
[267] Department of Pharmacology, University of Liverpool, Liverpool, UK
[268] Nuffield Department of Medicine, Peter Medawar Building for Pathogen Research, University of Oxford, UK
[269] Translational Gastroenterology Unit, Nuffield Department of Medicine, University of Oxford, UK
[270] Nottingham University Hospitals NHS Trust: Nottingham
[271] Nuffield Department of Medicine, John Radcliffe Hospital, Oxford, United Kingdom
[272] Department of Microbiology/Infectious Diseases, Oxford University Hospitals NHS Foundation Trust, John Radcliffe Hospital, Oxford, United Kingdom
[273] ISARIC Global Support Centre, Centre for Tropical Medicine and Global Health, Nuffield Department of Medicine, University of Oxford, Oxford, UK
[274] Institute of Infection, Veterinary and Ecological Sciences, University of Liverpool, Liverpool, UK
[275] Division of Infection and Immunity, University College London, UK
[276] Molecular and Clinical Cancer Medicine, Institute of Systems, Molecular and Integrative Biology, University of Liverpool
[277] Clatterbridge Cancer Centre NHS Foundation Trust, Liverpool, L7 8YA
[278] NIHR Health Protection Research Unit in Emerging and Zoonotic Infections, Liverpool, UK
[279] Centre for Clinical Infection and Diagnostics Research, Department of Infectious Diseases, School of Immunology and Microbial Sciences, King's College London, London, UK
[280] Department of Infectious Diseases, Guy's and St Thomas' NHS Foundation Trust, London, UK
[281] Institute of Evolutionary Biology, University of Edinburgh, Edinburgh, UK
[282] Department of Pediatrics and Virology, St Mary's Medical School Bldg, Imperial College London, London, UK
[283] NHS Greater Glasgow & Clyde, Glasgow, UK
[284] Walton Centre NHS Foundation Trust, Liverpool, UK
[285] MRC Centre for Molecular Bacteriology and Infection, Imperial College London, London,
[286] Department of Child Life and Health, University of Edinburgh, Edinburgh, UK
[287] National Phenome Centre, Division of Systems Medicine, Department of Metabolism, Digestion and Reproduction, Imperial College London, London W12 0NN, UK
[288] Blood Borne Virus Unit, Virus Reference Department, National Infection Service, Public Health England, London, UK
[289] Transfusion Microbiology, National Health Service Blood and Transplant, London, UK
[290] Department of Medicine, Imperial College London, London, UK
[291] Department of Infection, Immunity and Cardiovascular Disease, University of Sheffield, Sheffield, UK
[292] Tropical & Infectious Disease Unit, Royal Liverpool University Hospital, Liverpool, UK
[293] Liverpool Clinical Trials Centre, University of Liverpool, Liverpool, UK

[294] ISARIC, Global Support Centre, COVID-19 Clinical Research Resources, Epidemic diseases Research Group, Oxford (ERGO), University of Oxford
[295] Public Health Scotland
[296] Centre for Health Informatics, Division of Informatics, Imaging and Data Science, School of Health Sciences, Faculty of Biology, Medicine and Health, University of Manchester, Manchester Academic Health Science Centre, Manchester, UK
[297] EPCC, University of Edinburgh, Edinburgh, UK
[301] Institute of Infection, Veterinary and Ecological Sciences, Faculty of Health and Life Sciences, University of Liverpool, Liverpool, UK

13.4.6.1 Local Principal Investigators Kayode Adeniji, Daniel Agranoff, Ken Agwuh, Dhiraj Ail, Erin L. Aldera, Ana Alegria, Sam Allen, Brian Angus, Abdul Ashish, Dougal Atkinson, Shahedal Bari, Gavin Barlow, Stella Barnass, Nicholas Barrett, Christopher Bassford, Sneha Basude, David Baxter, Michael Beadsworth, Jolanta Bernatoniene, John Berridge, Colin Berry, Nicola Best, Pieter Bothma, Robin Brittain-Long, Naomi Bulteel, Tom Burden, Andrew Burtenshaw, Vikki Caruth, David Chadwick, David Chadwick, Duncan Chambler, Nigel Chee, Jenny Child, Srikanth Chukkambotla, Tom Clark, Paul Collini, Catherine Cosgrove, Jason Cupitt, Maria-Teresa Cutino-Moguel, Paul Dark, Chris Dawson, Samir Dervisevic, Phil Donnison, Sam Douthwaite, Andrew Drummond, Ingrid DuRand, Ahilanadan Dushianthan, Tristan Dyer, Cariad Evans, Chi Eziefula, Chrisopher Fegan, Adam Finn, Duncan Fullerton, Sanjeev Garg, Sanjeev Garg, Atul Garg, Effrossyni Gkrania-Klotsas, Jo Godden, Arthur Goldsmith, Clive Graham, Tassos Grammatikopoulos[69,70], Elaine Hardy, Stuart Hartshorn, Daniel Harvey, Peter Havalda, Daniel B Hawcutt, Maria Hobrok, Luke Hodgson, Anil Hormis, Joanne Howard, Michael Jacobs, Susan Jain, Paul Jennings, Agilan Kaliappan, Vidya Kasipandian, Stephen Kegg, Michael Kelsey, Jason Kendall, Caroline Kerrison, Ian Kerslake, Oliver Koch, Gouri Koduri, George Koshy, Shondipon Laha, Steven Laird, Susan Larkin, Tamas Leiner, Patrick Lillie, James Limb, Vanessa Linnett, Jeff Little, Mark Lyttle, Michael MacMahon, Emily MacNaughton, Ravish Mankregod, Huw Masson, Elijah Matovu, Katherine McCullough, Ruth McEwen, Manjula Meda, Gary Mills, Jane Minton, Kavya Mohandas, Quen Mok, James Moon, Elinoor Moore, Patrick Morgan, Craig Morris, Katherine Mortimore, Samuel Moses, Mbiye Mpenge, Rohinton Mulla, Michael Murphy, Thapas Nagarajan, Megan Nagel, Mark Nelson, Lillian Norris, Matthew K. O'Shea, Marlies Ostermann, Igor Otahal, Mark Pais, Carlo Palmieri[31,71], Selva Panchatsharam, Danai Papakonstantinou, Padmasayee Papineni, Hassan Paraiso, Brij Patel, Natalie Pattison, Justin Pepperell, Mark Peters, Mandeep Phull, Stefania Pintus, Tim Planche, Frank Post, David Price, Rachel Prout, Nikolas Rae, Henrik Reschreiter, Tim Reynolds, Neil Richardson, Mark Roberts, Devender Roberts, Alistair Rose, Guy Rousseau, Bobby Ruge, Brendan Ryan, Taranprit Saluja, Sarah Cole, Matthias L Schmid, Aarti Shah, Manu Shankar-Hari, Prad Shanmuga, Anil Sharma, Anna Shawcross, Jagtur Singh Pooni, Jeremy Sizer, Richard Smith, Catherine Snelson, Nick Spittle, Nikki Staines, Tom Stambach, Richard Stewart, Pradeep Subudhi, Tamas Szakmany, Kate Tatham, Jo Thomas, Chris Thompson, Robert Thompson, Ascanio Tridente, Darell Tupper-Carey, Mary Twagira, Nick Vallotton, Rama Vancheeswaran, Rachel Vincent, Lisa Vincent-Smith, Shico Visuvanathan, Alan Vuylsteke, Sam Waddy, Rachel Wake, Andrew Walden, Ingeborg Welters, Tony Whitehouse, Paul Whittaker, Ashley Whittington, Meme Wijesinghe, Martin Williams, Lawrence Wilson, Stephen Winchester, Martin Wiselka, Adam Wolverson, Daniel G Wootton, Andrew Workman, Bryan Yates, Peter Young.

[69] Paediatric Liver, GI & Nutrition Centre and MowatLabs, King's College Hospital, London, UK
[70] Institute of Liver Studies, King's College London, London, UK
[31] University of Liverpool
[71] Clatterbridge Cancer Centre NHS Foundation Trust

13.4.6.2 Outbreak Laboratory Staff and Volunteers

13.4.6.2.1 Outbreak Laboratory Staff and Volunteers Katie A. Ahmed.

Jane A Armstrong, Milton Ashworth, Innocent G Asiimwe, Siddharth Bakshi, Samantha L Barlow, Laura Booth, Benjamin Brennan, Katie Bullock, Nicola Carlucci, Emily Cass, Benjamin WA Catterall, Jordan J Clark, Emily A Clarke, Sarah Cole, Louise Cooper, Helen Cox, Christopher Davis, Oslem Dincarslan, Alejandra Doce Carracedo, Chris Dunn, Philip Dyer, Angela Elliott, Anthony Evans, Lorna Finch, Lewis WS Fisher, Lisa Flaherty, Terry Foster, Isabel Garcia-Dorival, Philip Gunning, Catherine Hartley, Anthony Holmes, Rebecca L Jensen, Christopher B Jones, Trevor R Jones, Shadia Khandaker, Katharine King, Robyn T. Kiy, Chrysa Koukorava, Annette Lake, Suzannah Lant, Diane Latawiec, Lara Lavelle-Langham, Daniella Lefteri, Lauren Lett, Lucia A Livoti, Maria Mancini, Hannah Massey, Nicole Maziere, Sarah McDonald, Laurence McEvoy, John McLauchlan, Soeren Metelmann, Nahida S Miah, Joanna Middleton, Joyce Mitchell, Shona C Moore, Ellen G Murphy, Rebekah Penrice-Randal, Jack Pilgrim, Tessa Prince, Will Reynolds, P. Matthew Ridley, Debby Sales, Victoria E Shaw, Rebecca K Shears, Benjamin Small, Krishanthi S Subramaniam, Agnieska Szemiel, Aislynn Taggart, Jolanta Tanianis-Hughes, Jordan Thomas, Erwan Trochu, Libby van Tonder, Eve Wilcock, J. Eunice Zhang.

References

1. Pereira, A. C. *et al.* Genetic risk factors and COVID-19 severity in Brazil: results from BRA-COVID study. *Human Molecular Genetics* **31**, 3021–3031 (2022).
2. Cruz, R. *et al.* Novel genes and sex differences in COVID-19 severity. *Human Molecular Genetics* **31**, 3789–3806 (2022).
3. Kousathanas, A. *et al.* Whole-genome sequencing reveals host factors underlying critical COVID-19. *Nature* **607**, 97–103 (2022).
4. Niemi, M. E. K. *et al.* Mapping the human genetic architecture of COVID-19. *Nature* **600**, 472–477 (2021).
5. Ellinghaus, D. *et al.* Genomewide association study of severe covid-19 with respiratory failure. *The New England journal of medicine* **383**, 1522–1534 (2020).
6. Pairo-Castineira, E. *et al.* Genetic mechanisms of critical illness in COVID-19. *Nature* **591**, 92–98 (2021).
7. Pathak, G. A. *et al.* A first update on mapping the human genetic architecture of COVID-19. *Nature* **608**, E1–E10 (2022).
8. Degenhardt, F. *et al.* Detailed stratified GWAS analysis for severe COVID-19 in four european populations. *Human molecular genetics* **31**, 3945–3966 (2022).
9. Kanai, M. *et al.* Meta-analysis fine-mapping is often miscalibrated at single-variant resolution. *Cell Genomics* **0**, (2022).
10. Mineta, K. A. G., Katsuhiko AND Goto. Population structure of indigenous inhabitants of arabia. *PLOS Genetics* **17**, 1–18 (2021).
11. COVID-19 Host Genetics Initiative. Mapping the human genetic architecture of COVID-19. *Nature* (2021) doi:10.1038/s41586-021-03767-x.
12. Shelton, J. F. *et al.* Trans-ancestry analysis reveals genetic and nongenetic associations with COVID-19 susceptibility and severity. *Nature Genetics* **53**, 801–808 (2021).
