# Supplementary material for: GWAS and meta-analysis identifies 49 genetic variants underlying critical COVID-19
Source: Nature. 2023 May 17;617(7962):764–8. doi: 10.1038/s41586-023-06034-3 (PMC10208981; doi:10.1038/s41586-023-06034-3)
Supplement: Supplementary file 2 — Reporting Summary [file 41586_2023_6034_MOESM2_ESM.pdf]

nature portfolio

Corresponding author(s): J. Kenneth Baillie

Last updated by author(s): Feb 22, 2023

Reporting Summary

Nature Portfolio wishes to improve the reproducibility of the work that we publish. This form provides structure for consistency and transparency in reporting. For further information on Nature Portfolio policies, see our Editorial Policies and the Editorial Policy Checklist.

Statistics

For all statistical analyses, confirm that the following items are present in the figure legend, table legend, main text, or Methods section.

n/a	Confirmed	
☐	☒	The exact sample size (*n*) for each experimental group/condition, given as a discrete number and unit of measurement
☐	☒	A statement on whether measurements were taken from distinct samples or whether the same sample was measured repeatedly
☐	☒	The statistical test(s) used AND whether they are one- or two-sided<br>*Only common tests should be described solely by name; describe more complex techniques in the Methods section.*
☐	☒	A description of all covariates tested
☐	☒	A description of any assumptions or corrections, such as tests of normality and adjustment for multiple comparisons
☐	☒	A full description of the statistical parameters including central tendency (e.g. means) or other basic estimates (e.g. regression coefficient) AND variation (e.g. standard deviation) or associated estimates of uncertainty (e.g. confidence intervals)
☐	☒	For null hypothesis testing, the test statistic (e.g. *F*, *t*, *r*) with confidence intervals, effect sizes, degrees of freedom and *P* value noted<br>*Give P values as exact values whenever suitable.*
☒	☐	For Bayesian analysis, information on the choice of priors and Markov chain Monte Carlo settings
☒	☐	For hierarchical and complex designs, identification of the appropriate level for tests and full reporting of outcomes
☐	☒	Estimates of effect sizes (e.g. Cohen's *d*, Pearson's *r*), indicating how they were calculated

Our web collection on statistics for biologists contains articles on many of the points above.

Software and code

Policy information about availability of computer code

Data collection	Illumina i-scan platform, GenomeStudio Analysis software v2.0.3, GSAMD-24v3-0-EA_20034606_A1.bpm manifest and cluster file provided by manufacturer for GenOMICC UK genotyping. Affymetrix Saub1 chip (Saudi Arabia) and Axiom Analysis suite 5.1.1.1 manifest and cluster file provided by manufacturer for GenOMICC Saudi Arabia. Other datasets were collected previously and Summary statistics used. In order to detect eQTLs, untreated primary monocytes were prepared from 174 healthy individuals of Northern European (British) ancestry recruited via the Oxford biobank. Poly-A RNA was paired-end 100bp sequenced in the Oxford Genome Centre using Illumina Hiseq-4000 machines (median = 47,735,438 reads per sample). Genotyping was performed with Illumina HumanOmniExpress with coverage of 733,202 separate markers.
Data analysis	GenomeStudio v2.03, Plink 1.9, Plink 2.0, King 2.1, R v3.6.3, python v3.7, GATK 4.0, USC liftover, GCTA v1.92 ,REGENIE v3.1.2, metal (2018-08-28), BCFtools 1.9, QCtools 1.3, FlashPCA2, admixture,1.3.0 SMR/HEIDI v1.03, MetaXcan v0.6.5, MiniMac4 v1.0, MetaSubtract package v1.60, Rv4.1.0, FUSION (commit e1ba5f7), python v3.10, HISAT2, bamtools, picard v.1.105, verifyBAMID, samtools, SHAPEIT2, PBWT, vcftools v.0.1.12b, HTseq, Deseq2.

For manuscripts utilizing custom algorithms or software that are central to the research but not yet described in published literature, software must be made available to editors and reviewers. We strongly encourage code deposition in a community repository (e.g. GitHub). See the Nature Portfolio guidelines for submitting code & software for further information.

Data

Policy information about availability of data

All manuscripts must include a data availability statement. This statement should provide the following information, where applicable:

- Accession codes, unique identifiers, or web links for publicly available datasets
- A description of any restrictions on data availability
- For clinical datasets or third party data, please ensure that the statement adheres to our policy

Downloadable summary data are available through the GenOMICC data site https://genomicc.org/data. Summary statistics will be available without including 23andme summary statistics, except for the 10,000 most significant hits which will have full summary statistics available. The full GWAS summary statistics for the 23andMe discovery data set will be made available through 23andMe to qualified researchers under an agreement with 23andMe that protects the privacy of the 23andMe participants. Please visit https://research.23andMe.com/dataset-access/ for more information and to apply to access the data.

All individual-level genotype and whole genome sequence data (for both academic and commercial uses) can be accessed through the UKRI/HDR UK Outbreak Data Analysis Platform https://odap.ac.uk. A restricted dataset for a subset of GenOMICC participants is also available through the Genomics England data service.

Monocyte RNA-seq data is available under the title “Monocyte gene expression data” within the Oxford University Research Archives. DOI: 10.5287/ora-ko7q2nq66 (http://dx.doi.org/10.5287/ora-ko7q2nq66)

Human research participants

Policy information about studies involving human research participants and Sex and Gender in Research.

Reporting on sex and gender	Sex was asked in the study, and then confirmed by genotype. When there was a discordance between self-reported sex and genotyped sex the sample was removed from the study.<br>Sex-specific analysis were performed for both males and females but did not give any significant results<br>Overall the study included 45472 cases from which ~40% are female. As data comes from summary statistics from other analysis, it has not been possible to calculate the exact number of females and males.
Population characteristics	Cases have tested positive for Covid-19 and needed hospitalisation or ICU admission. Controls come from different sources, people which experienced mild (non-hospitalised) Covid-19 or population controls from different Biobanks. In GenOMICC Brazil, mild cases were selected from serological studies of SARS-COV2 infection and PCR test results among health professionals and the general population. In GenOMICC Saudi Arabia, mild controls were selected after a positive PCR test. SCOURGE population controls were extracted from Spanish DNA Biobank and the GR@CE consortium. Participants in 23and me analysis provided informed consent and answered surveys online according to 23andme human subjects research protocol.<br>Untreated primary monocytes were prepared from 174 healthy individuals from British ancestry via the Oxford Biobank
Recruitment	Cases were recruited by different studies in hospitals. All participants gave informed consent. Mild controls were recruited on the basis of having experienced mild or assymptomatic Covid-19.<br>For GenOMICC UK and ISARIC4C population controls were used from UK Biobank (project 788), or 100,000 genomes from genomics england. SCOURGE project used controls from Spanish DNA Biobank and the GR@CE consortium
Ethics oversight	GenOMICC Scotland: Scotland A Research Ethics Committee 15/SS/0110.<br>GenOMICC England/Wales/Northern Ireland Coventry and Warwickshire Research Ethics Committtee 19/WM/0247.<br>GenOMICC Brazil (BraCovid) National Research Ethics Committee (CONEP) and Ethics Committee for the Analysis of Research Projects at HC FMUSP (CAPPesq) 5025/20/054.<br>GenOMICC Saudi Arabia IRB at King Abdullah International Medical Research Center.<br>ISARIC4C England/Wales/Northern Ireland South Central Oxford C Research Ethics Committee 13/SC/0149.<br>ISARIC4C Scotland Scotland A Research Ethics Committee 20/SS/0028.<br>SCOURGE Galician Ethical Committee 2020/197.<br>23andme Ethical and Independent Review Services \url{http://www.eandireview.com}.<br>Covid-19 HGI Multiple ethics committees (https://www.covid19hg.org/).<br>Oxford biobank approved by South Central - Oxford C Research Ethics Committee, reference 18/SC/0588

Note that full information on the approval of the study protocol must also be provided in the manuscript.

Field-specific reporting

Please select the one below that is the best fit for your research. If you are not sure, read the appropriate sections before making your selection.

☒ Life sciences ☐ Behavioural & social sciences ☐ Ecological, evolutionary & environmental sciences

For a reference copy of the document with all sections, see nature.com/documents/nr-reporting-summary-flat.pdf

Life sciences study design

All studies must disclose on these points even when the disclosure is negative.

Sample size	cases 45472, controls 2929541
Data exclusions	no exclusions
Replication	As we meta-analysed all public data available for Covid-19, to verify replicability of the findings we performed a heterogeneity test between studies, using a Cochran's Q-test
Randomization	Not relevant to the study
Blinding	Not relevant to the study

Reporting for specific materials, systems and methods

We require information from authors about some types of materials, experimental systems and methods used in many studies. Here, indicate whether each material, system or method listed is relevant to your study. If you are not sure if a list item applies to your research, read the appropriate section before selecting a response.

Materials & experimental systems

n/a	Involved in the study	
☒	☐	Antibodies
☒	☐	Eukaryotic cell lines
☒	☐	Palaeontology and archaeology
☒	☐	Animals and other organisms
☒	☐	Clinical data
☒	☐	Dual use research of concern

Methods

n/a	Involved in the study	
☒	☐	ChIP-seq
☒	☐	Flow cytometry
☒	☐	MRI-based neuroimaging
